# Discovery and description of novel phage genomes from urban microbiomes sampled by the MetaSUB consortium

**DOI:** 10.1038/s41598-024-58226-0

**Published:** 2024-04-04

**Authors:** Vinicius S. Flores, Deyvid E. Amgarten, Bruno Koshin Vázquez Iha, Krista A. Ryon, David Danko, Braden T. Tierney, Christopher Mason, Aline Maria da Silva, João Carlos Setubal

**Affiliations:** 1 Departamento de Bioquímica, Instituto de Química, Universidade de São Paulo, São Paulo, 05508-000 Brazil; 2 Present Address: Hospital Israelita Albert Einstein, São Paulo, Brazil; 3 Weill Cornell Medicine, New York, NY USA; 4 Biotia, New York, NY USA; 5 Harvard Medical School, Cambridge, MA USA

**Keywords:** Metagenomics, Genome informatics, Bacteriophages

## Abstract

Bacteriophages are recognized as the most abundant members of microbiomes and have therefore a profound impact on microbial communities through the interactions with their bacterial hosts. The International Metagenomics and Metadesign of Subways and Urban Biomes Consortium (MetaSUB) has sampled mass-transit systems in 60 cities over 3 years using metagenomics, throwing light into these hitherto largely unexplored urban environments. MetaSUB focused primarily on the bacterial community. In this work, we explored MetaSUB metagenomic data in order to recover and analyze bacteriophage genomes. We recovered and analyzed 1714 phage genomes with size at least 40 kbp, from the class *Caudoviricetes*, the vast majority of which (80%) are novel. The recovered genomes were predicted to belong to temperate (69%) and lytic (31%) phages. Thirty-three of these genomes have more than 200 kbp, and one of them reaches 572 kbp, placing it among the largest phage genomes ever found. In general, the phages tended to be site-specific or nearly so, but 194 genomes could be identified in every city from which phage genomes were retrieved. We predicted hosts for 48% of the phages and observed general agreement between phage abundance and the respective bacterial host abundance, which include the most common nosocomial multidrug-resistant pathogens. A small fraction of the phage genomes are carriers of antibiotic resistance genes, and such genomes tended to be particularly abundant in the sites where they were found. We also detected CRISPR-Cas systems in five phage genomes. This study expands the previously reported MetaSUB results and is a contribution to the knowledge about phage diversity, global distribution, and phage genome content.

## Introduction

Viruses that infect bacteria (bacteriophages, or phages for short) are the most abundant and diverse entities in the biosphere and have been found in every explored biome^1^. Phages require a host to reproduce, and their abundance and distribution in general follow the abundance and distribution of their specific hosts^2,3^. Through the intrinsic linkage between parasite and host, phages profoundly impact microbial communities, by shaping abundance, population dynamics, physiology, metabolism, and evolutionary trajectories of their bacterial hosts^3–6^.

Phage genetic material is composed of either DNA or RNA, which may be double-stranded or single-stranded. The majority of the phages isolated so far have dsDNA genomes packaged into an icosahedral capsid connected to a tail, belonging to the class *Caudoviricetes*^1,7^. Most of the dsDNA phages contain genomes smaller than 200 kbp (on average 40–50 kbp)^8,9^, but phage genomes with sizes that are at least 200 kbp (jumbo phages) or at least 500 kbp (megaphages) have been described^1,10–12^. Phage genomes exhibit remarkable diversity and complex evolutionary relationships due the pervasive mosaicism^13,14^, despite the conserved structural similarities of some viral proteins, such as the major capsid and DNA packaging motor proteins^1,15^.

Phage infection modes comprise a continuum that spans antagonistic to beneficial interactions, ranging from infections by obligately lytic phages to persistent lysogenic infections by temperate phages^5^. Lytic phages hijack the cell molecular machinery, shuts down host defense mechanisms, replicate its genome, produce new virus particles, and destroy its host cell to release virion progeny. Some phages present a non-bactericidal infection mode, where virions are produced and continuously released (chronic infection). Temperate phages can undergo either virion-productive or lysogenic cycles. When in the lysogenic cycle, temperate phages integrate their genomes into the host chromosome or plasmid and replicate along with their host cells, and as such are denoted as prophages. Diverse factors related to the virus itself, the host cell and the environmental conditions can influence both the establishment of persistent lysogeny and the induction of a typical lytic cycle in temperate phages^4,6^.

Along with the genes necessary for virus propagation, the phage genome may also encode toxins, virulence factors, antibiotic resistance genes (ARGs), and auxiliary metabolic genes (AMGs) that, among other functions, can reprogram bacterial metabolism^1,16^. Moreover, phage genomes encode many genes of unknown function, opening an opportunity for exploring medical, agricultural, and industrial biotechnologies^17–19^. The research on phage genomes has been of substantial importance to improve phage-based biocontrol approaches to tackle multidrug-resistant pathogens by means of phage genome engineering or phage-encoded enzymes^20–25^.

Culture-independent approaches such as metagenomics and metatranscriptomics are unveiling the huge diversity, abundance, and function of phages in various microbiomes such as terrestrial, wastewater urban systems, and marine environments^26–30^, honey-bee gut^31^ and human samples^32–34^. Indeed, the growth of viral sequence catalogues like IMG/VR^35^ and the Gut Phage Database^32^ is noteworthy. Yet, it remains challenging to connect a phage with its bacterial host solely based on genome sequence information^36^.

The International Metagenomics and Metadesign of Subways and Urban Biomes Consortium (MetaSUB) aims to produce an extensive exploration and characterization of urban microbiomes^37,38^. An atlas of 4,728 metagenomic samples from mass-transit systems in 60 cities over 3 years was shown to include 1,302 metagenome assembled genomes from bacteria, 2 from archaea and 16,584 from viruses (UviGs, uncultivated viral genomes), corresponding to 11,614 viral species, of which 94.1% did not match any viral sequence present in IMG/VR^38^. To further explore the remarkable viral diversity predicted in global urban microbiomes we present a catalog of highly curated bacteriophage genomes recovered from the MetaSUB metagenomic samples. We focused primarily on phages from the class *Caudoviricetes* with genomes at least 40 kbp long.

## Results

### Recovered phage genomes

**Figure 1:**
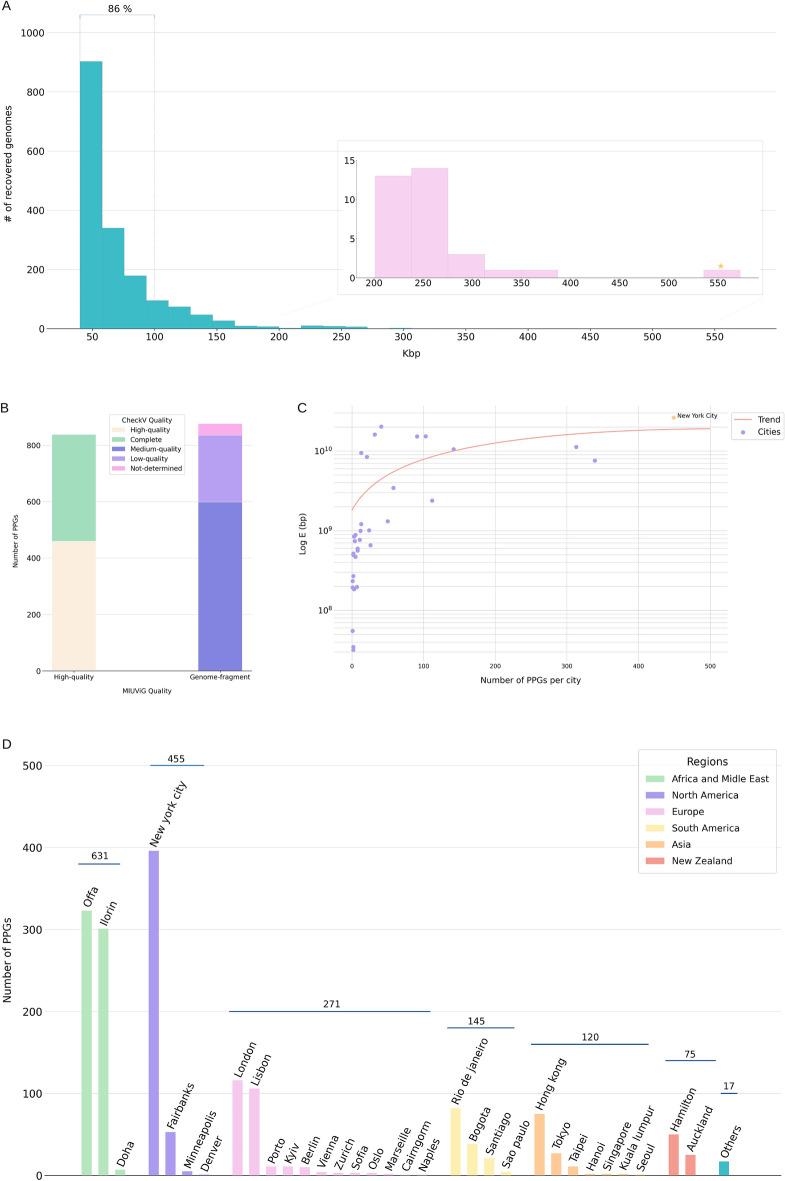
Quality assessment and distribution of PPGs. (**A**) Distribution of PPGs per size in kbp. The distribution of huge PPGs appears enlarged. The yellow star indicates the huge PPG with 572 kbp. (**B**) Completeness of PPGs. (**C**) Rarefaction curve of PPGs. The curve was built using the total number of base pairs in the contigs assembled for each sample of each city. (**D**) Number of PPGs per city and region.

From 3836 MetaSub samples with assembled contigs, 1558 presented at least one contig satisfying our length filters (size greater than or equal to 40 kbp). A total of 94,418 contigs from these samples were submitted to MARVEL^39^ for phage genome prediction, followed by a dereplication step. This process resulted in 1714 contigs, each predicted to be a Putative Phage Genome (PPG) (Supplementary Table S1). Contig lengths ranged from 40,000 bp to 572,750 bp, most of them (86%) ranging from 40 to 100 kbp (Fig. 1A). The vast majority (84%) of PPGs was classified as Complete, High-quality or Medium-quality by CheckV (Fig. 1B). Two-hundred seventy-five PPGs (16%) presented some type of terminal repeat, which is an indication that the genome is complete^33^.

Of the 60 cities sampled in the MetaSUB project, 33 yielded at least one PPG. New York City was the city with the most PPGs (Fig. 1D), and saturation in the number of possible phage genomes that can be recovered may have been reached for this location (Fig. 1C), given the filters applied (see Methods) and the sample collection and sequencing strategies used^38^.

We found that 33 PPGs fit into the jumbo (≥ 200 kbp) or mega (≥ 500 kbp) bacteriophage category (Fig. 1A). More than half of these huge (jumbo + mega) PPGs came from samples collected in the Nigerian cities Offa and Ilorin (Supplementary Fig. S1A). The largest PPG is MSP0001 (572 kbp). Sample H75CGCCXY_SL263641 (New York City) provided by far the most reads for this PPG (204,721 reads), but three other samples (HKC32ALXX_SL254707: Berlin; HMC2KCCXY_SL336564 and HNHKFCCXY_SL345930: Hong Kong) provided more than a thousand reads.

### Phage genomes novelty analysis

Using vContact2 and the Millard phage genome reference database (MillardDB) to build a protein-coding-gene-sharing network^40^, we obtained 225 clusters containing about 33.5% of PPGs (Supplementary Figures S2, S3 and S4). The remaining PPGs (66.5%) were not clustered (48.5%) or were singletons (18%). Ninety-eight clusters have at least one phage from the MillardDB. The presence of a reference phage genome with a taxonomic classification in a cluster allows classification of phages in that cluster. However, most of the MillardDB reference phages do not have a family yet assigned, thus 87 clusters were unassigned at this taxonomic level. Nevertheless, 11 clusters did have reference phages assigned to a family, as defined by the International Committee of Taxonomy of Viruses (ICTV)^7^. In this way, four clusters were assigned to *Peduoviridae* and three clusters were assigned to *Autographiviridae*, in addition to four other clusters assigned each to another specific family (Supplementary Fig. S2). While 76 clusters included reference phages with no genus assigned, 12 clusters included reference phages classified to the same genus and 10 clusters included reference phages from different genera (Supplementary Fig. S3).

A total of 127 clusters did not contain reference genomes from the MillardDB. These clusters may represent novel phage genera or families (Supplementary Fig. S4). However, 46 of them are composed of nodes with a small internal edge weight (less than 100), which means that these clusters may not be robust. Thus, to avoid an overestimated number of possible new phage genera, we applied the Viral Clustering (VC) method^32^ to all PPGs, generating 16 clusters. Five clusters were the same as those found by vContact2, and three had partial overlap (Supplementary Fig. S4). These eight clusters contain 18 PPGs and no reference phage genome. The number 18 is thus our best estimate for a lower bound on the number of novel PPGs at the genus or family level in the MetaSUB samples analyzed here using a clustering approach.

Regarding the 33 huge PPGs, 26 were not clustered by vContact2 and two out of four clusters with huge PPGs are composed of PPGs only (Supplementary Fig. S1C).

In terms of nucleotide comparisons, 4.5% of PPGs aligned to at least one genome in the four reference databases used (Table 1). Comparisons using amino acid sequences resulted in 20.3% of PPGs having a significant alignment (Table 1). This is evidence that at least 80% of the PPGs are novel phage genomes. None of the huge PPGs had significant hits.

**Table 1:** Alignments between the PPGs and phage genomes of reference datasets. The PPGs were aligned by Average Nucleotide Identity (ANI/ FastANI) and Average Aminoacid Identity (AAI/ CompareM) using four different databases. Gray-shaded rows present results of ANI alignments and unshaded rows show results of AAI alignments.

Datasets	# aligned PPGs	# aligned distinct PPGs (%)	# distinct subject sequence that were aligned
NCBI Virus RefSeq	38	9 (0.52%)	37
	179	52 (3.03%)	159
IMG/ VR	280	56 (3.26%)	224
	4715	292 (17.03%)	1902
MillardDB	259	23 (1.34%)	232
	951	105 (6.12%)	813
NCBI GenBank	13	6 (0.35%)	12
	128	72 (3.26%)	105
Total	590	77 (4.50%)	505
	5973	348 (20.30%)	2979

### PPG host prediction

**Figure 2:**
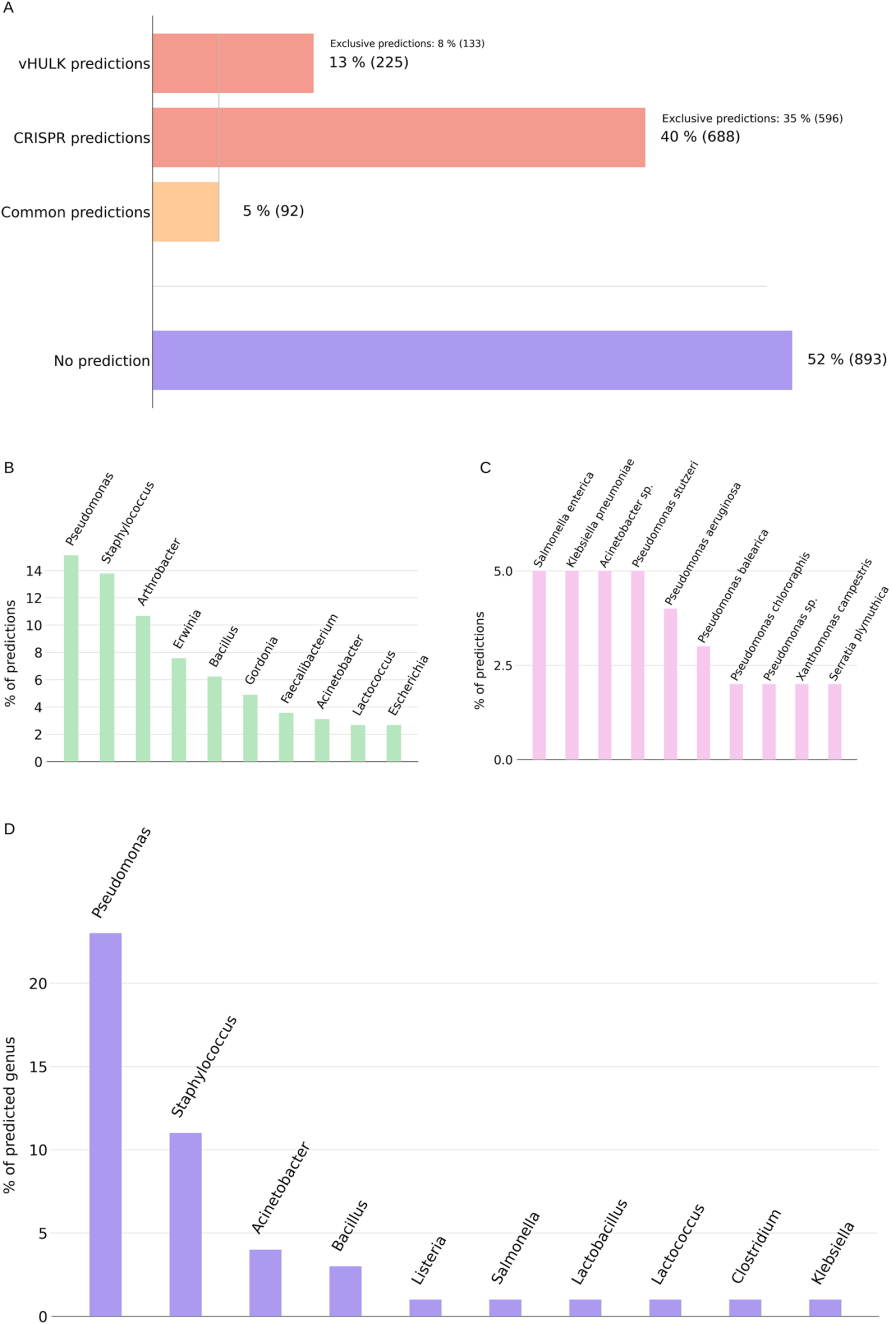
PPG host prediction, at the genus level. (**A**) Distribution of host prediction by method. (**B**) Top 10 host predictions by vHULK. (**C**) Top 10 host prediction by CRISPR alignments. (**D**) Distribution of shared predictions by vHULK and CRISPR-Cas alignments.

The 1,714 PPGs were submitted to host prediction using vHULK^41^, which resulted in 13% predictions to the targets in vHULK models (Fig. 2A). *Pseudomonas* and *Staphylococcus* were the two genera with most predictions (Fig. 2B). vHULK predicted that 43 of the PPGs have a putative host that is a member of the ESKAPE group (***Enterococcus faecium***, ***Staphylococcus aureus***, ***Klebsiella pneumoniae***, ***Acinetobacter baumanni***, ***Pseudomonas aeruginosa*** and ***Enterobacter*** spp.)^42^ , which includes the most common nosocomial multidrug-resistant pathogens (Supplementary Table S1). Moreover 11 PPGs predicted to have an ESKAPE bacterium as host were clustered by vContact2 with high edge weight with reference phages whose identified host genus is in the ESKAPE group (Supplementary Fig. S5), with some PPGs predicted to have more than one bacterial genus as host.

The PPGs were also queried for similarity against spacers of the CRISPR-Cas database^43^. Six hundred eighty-eight PPGs (40%) were linked to a host using CRISPR-Cas alignments (Fig. 2A). The predictions were sparsely distributed among putative hosts (Fig. 2C). In terms of genus predictions, only 92 PPGs (5%) had the same prediction by both approaches, with *Pseudomonas* being the most frequent host genus (Fig. 2D).

In what follows, we use host predictions obtained from vHULK or CRISPR-Cas or both (Supplementary Tables S3, S4, and S5). Conflicting host predictions for a given PPG by these two methods were not considered.

We identified putative hosts for the majority of the huge PPGs. Most of the predictions give *Erwinia* as putative host genus (Supplementary Fig. S1B) according to vHULK (Supplementary Table S1). There were no shared predictions between the CRISPR-Cas approach and vHULK for huge PPGs.

Out of the 75 most common bacterial species reported in MetaSUB microbiomes^38^, 20 appear as a predicted host for the PPGs (Supplementary Table S6). A total of 228 PPGs (13.3%) were assigned with a putative host reported in the list of 75 most common species, with *Salmonella enterica* (14%), *Pseudomonas aeruginosa* (13.6%) and *Klebsiella pneumoniae* (13.5%) being the top three that were predicted as hosts for PPGs (Supplementary Table S6).

When we take into account city-specific MetaSUB data, we observed agreement between the abundance of the 20 bacterial species in the top 75 list for which there is at least one PPG with that species as host and that PPG abundance in that city (Supplementary Fig. S6). We highlight the cities of Minneapolis and Kuala Lumpur, in which *Staphylococcus epidermidis* and *P. aeruginosa* were the most abundant bacteria, respectively. PPGs predicted to have *S. epidermidis* and *P. aeruginosa* as hosts were also the most abundant (27.2% and 38.3%, respectively) in those cities (Supplementary Fig. S6).

### Phage genome content analysis

**Figure 3:**
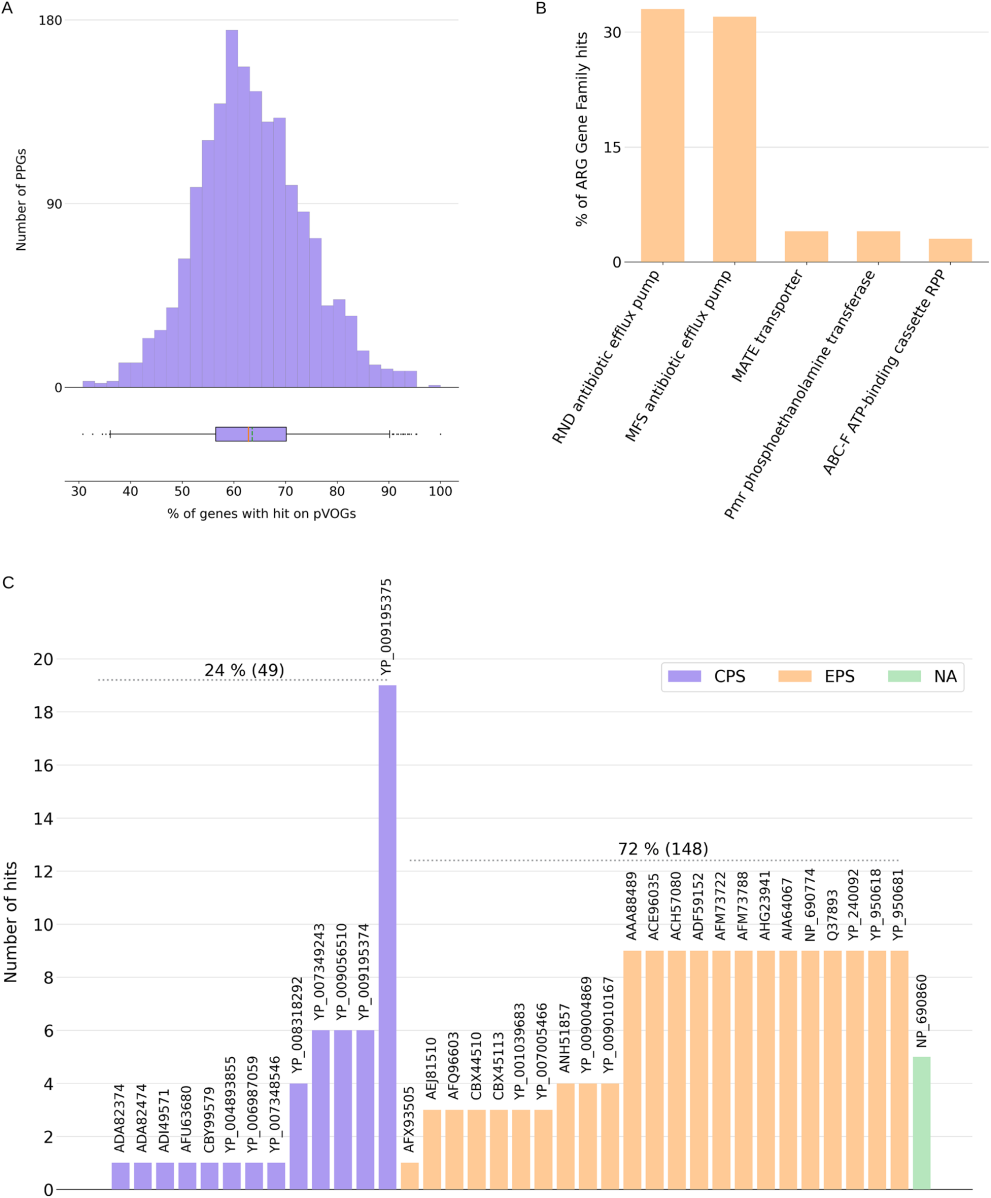
Functional exploration of PPGs. (**A**) Distribution of known phage protein families from pVOGs in PPG predicted proteins. The green and red lines in the whisker plot mark the median and mean values, respectively. (**B**) Top five antimicrobial resistance genes gene (ARG) families found in PPGs. Significant hits were grouped by ARG family. All hits grouped by ARG family can be found in Supplementary Table S3. (**C**) Putative depolymerases found in 45 PPGs. Capsular polysaccharides depolymerases bars are colored in yellow, exopolysaccharides depolymerases bars are colored in orange, and non-identified matches are colored in red. The accession numbers of aligned depolymerases are shown above each bar.

PPGs were investigated regarding the presence of genes encoding known families of phage proteins. Searches against the pVOGs database of phage protein HMM profiles^44^ showed that, on average, 63% of proteins encoded by a PPG presented at least one significant match. Values ranged from 30 to 100% depending on the phage genome considered (Fig. 3A).

We identified 52 PPGs (2.7%) carrying putative ARGs. More than half of these putative ARGs are related to Multi Drug Resistance (MDR) efflux pumps from families of RND and MFS antibiotic efflux pumps (Fig. 3B), which have been reported as commonly found in MetaSUB samples^38^ and are commonly found in bacterial genomes but do not necessarily result in a resistant phenotype^45^.

A total of 201 putative depolymerase genes were identified in 45 PPGs (2.3%). The hits were grouped by the bacterial polysaccharide targets, showing that the majority matched depolymerases for exopolysaccharide (EPS) followed by capsular polysaccharides (CPS). No matches for lipopolysaccharides (LPS) depolymerases were found (Fig. 3C).

We searched for the presence of CRISPR-Cas systems in the PPGs and found them in nine PPGs (Supplementary Table S7). In some PPGs, only CRISPR arrays (spacers) could be detected. Supplementary Fig. S7 shows the five PPGs for which both Cas clusters and CRISPR arrays were detected.

### Lifestyle prediction

Nearly 42% of PPGs were classified as prophages by both CheckV^46^ and VIBRANT^47^ (Table 2). About 69% of PPGs are predicted to have a temperate lifestyle as reported by VIBRANT, and 68.6% of PPGs were predicted with the same lifestyle by Bacphlip^48^ (Table 2). Grouping PPGs by their predicted lifestyle for each city, we found a similar pattern. Thirty-one out of the 33 cities showed that the lysogenic lifestyle is the most common strategy predicted for the PPGs (Supplementary Fig. S8). The two exceptions were the cities Taipei and Marseille, in which the lytic lifestyle is more frequent.

**Table 2:** Putative prophages in the PPGs and their lifestyles as predicted by CheckV and VIBRANT. The lifestyle classification was carried out as described in the Methods Section. (*) VIBRANT did not classify 114 PPGs, while the other tools classified all PPGs.

	CheckV	VIBRANT *	Bacphlip
Prophage	55%	44%	–
Unique	12%	0.9%	–
Intersection	42%	–	
Lifestyles	Temperate	69%	68.6%
	Lytic	31%	31.4%

### Distribution and abundance of PPGs among sites

The mean number of PPGs detected per sample was 730 ± 272. The vast majority of PPGs in a sample has very small relative abundance (less than 0.1%), but in 357 samples (50.6%) there were PPGs with more than 10% relative abundance. The two largest such fractions (98% for MSP0350 and 89% for MSP1223) were found in samples HMCMJCCXY_SL335787 and HMCMJCCXY_SL335821, both from Fairbanks (AK), USA, respectively (Supplementary Table S8).

There is a weak correlation between the number and relative abundances of PPGs and the amount of sequencing data per site in terms of DNA base-pairs in contigs (Fig. 1C).

**Figure 4:**
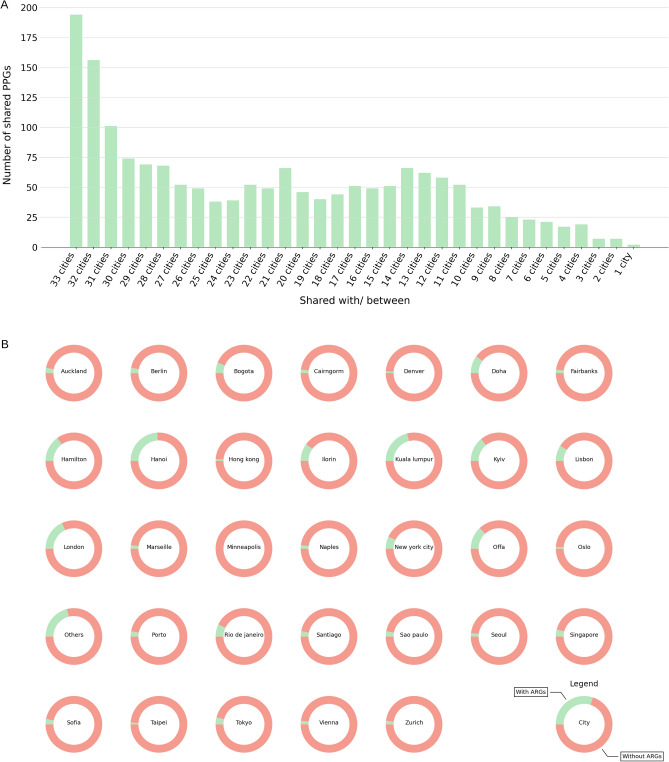
PPG cosmopolitanism and abundance per predicted host. (**A**) Number of PPGs shared by cities. (**B**) Overall abundance of PPGs carrying ARGs vs non-carriers per city.

Of the 1714 PPGs identified, 194 (11.3%) were detected in each of the 33 cities that yielded PPGs (Fig. 4A). Except for two PPGs (MSP1222 and MSP1696, found only in Hong Kong), all others were found in at least two cities.

We compared cities based on the presence and relative abundance of the PPGs, seeking to cluster cities using 12 different combinations between affinity and linkage criteria. Briefly, this means that cities sharing the same PPGs with similar relative abundances will tend to cluster together. Offa and Ilorin (both in Nigeria) formed a consistent cluster in all 12 combinations. The next most frequent clustering was New York-Rio de Janeiro, Denver-Oslo-Zürich, and Tokyo-Bogota, which clustered in nine combinations (Supplementary Fig. S9).

As mentioned above, the number of PPGs which were identified as ARG carriers was relatively small. However, we observed that for some cities, such as Kuala Lumpur and Hanoi, the ARG carriers represent more than 15% of the total abundance of PPGs (Fig. 4B; Supplementary Fig. S10). In addition, for some cities the mean abundance of ARG carriers is statistically greater when compared with the mean abundance of non-ARG carriers. In the general analysis of MetaSUB samples, Hanoi, Offa, and Ilorin samples were reported to have a large number of ARGs^38^; in our analysis, these cities also present a mean abundance of ARG carriers greater than non-ARG carriers, and this difference is statistically significant (Supplementary Table S9).

### The largest PPG

The largest PPG we found (MSP0001) is 572 kbp long, which means it is among the largest megaphages found to date^12^. It is predicted to have a lytic lifestyle, was found in 30 cities, and its host is predicted to be a member of the *Agrobacterium* genus (vHULK prediction).

## Discussion

In the first reported results of the MetaSUB project^38^, 16,584 uncultivated viral genomes (UViGs) were assembled, in addition to many other results concerning bacterial and archaeal genomes. However, Danko et al.^38^ presented only a very broad analysis of these UviGs. Among the reported findings are: the viral clusters based on taxonomy are weakly cosmopolitan, meaning that the majority of cluster members are found at or near one location; of the 11,614 predicted viral species for the 16,584 UviGs, 94.1% did not match any viral sequence in IMG/VR at the species level; and a host was predicted for 3,979 viral species (34,3%).

Here we present results from the same set of samples analyzed by the MetaSUB consortium^38^, focusing on the genomes of bacteriophages (PPGs) from the class *Caudoviricetes*, and analyzing and interpreting these PPGs in much deeper fashion than Danko et al.^38^ did with the UViGs. Out of the 1714 PPGs, nearly half (48,9%) have a high level of completeness. This result contrasts with what has been reported in some recent studies where only a small fraction of recovered phage genomes has a substantial degree of completeness [less than 30% in^32^; less than 8% in^33^]. This high level of completeness is likely due to the minimum length requirement (40 kb) of our processing pipeline, which aimed to capture a high proportion of complete genomes.

Most (80%) of the 1714 PPGs obtained can be considered novel, including all large (≥ 200 kbp) PPGs. In addition, our results suggest that these 1714 PPGs contain at least eight new phage genera based on Viral Clustering method^32^ or up to 127 new genera based on vContact2^40^ and the Millard phage genome reference database. Therefore, the dataset presented here can be considered a sizable contribution for the knowledge of phage diversity in urban environments. This result is consistent with the degree of novelty for viral genomes reported by Danko et al.^38^, which was 94.1%.

Most of the PPGs were site-specific or nearly so. Nevertheless, 194 PPGs (11.3%) were detected in all sites sampled. This result is consistent with the weakly cosmopolitanism of UViGs reported by Danko et al.^38^. We analyzed these 194 PPGs in search of shared traits (such as ARG content, lifestyle, predicted host), but we did not find any pattern. Most of these ubiquitous PPGs were predicted to be prophage/temperate (150; 77%) and to be novel (152; 80%).

Most samples presented a large PPG diversity, but there were exceptions, most notably the dominating PPGs MSP0350 and MSP1223 in samples from Fairbanks. The annotation of these PPGs did not reveal anything that suggested a reason for their dominance.

We identified putative hosts for 48% of the PPGs. We observed that the hosts most frequently predicted are part of the 75 most common bacterial species reported by Danko et al.^38^. This result is evidence that the phage host predictions here reported are in general reliable. Three hundred twenty-five PPGs were predicted to have as host a member of the ESKAPE group^42^. This suggests that the urban environments where these phages were found are a good source of phages that could be screened as potential weapons against the multidrug resistant bacteria that are members of that group. Moreover, some PPGs were predicted to have more than one bacterial genus as host, indicating that these phages may be generalists in the sampled environment. These results are in line with the suggestion that phage host interactions in urban and natural environments might be broad and span bacterial genera^29,49–51^.

The majority of PPGs were predicted to have a lysogenic lifestyle. While there may be a bias in this result caused by the strategy used to collect and sequence the genetic material^38^, grouping the PPGs by predicted lifestyle strategy for each city showed that lysogenic bacteriophages are more frequent than lytic ones. This result suggests that lysogeny may be the preferred strategy of bacteriophages in the urban environments that were sampled. The relative frequency of lytic and lysogenic lifestyles seems to vary across ecosystems, but the ecological factors that influence this balance are a matter of debate^52^. For instance, lysogeny is prevalent in gut microbiota^53^ and in pre-bloom of polar marine environments^54^.

Only a relatively small number of PPGs carry antibiotic resistance genes (ARGs), supporting the hypothesis that phages rarely encode these genes and probably are not, in general, a major reservoir of ARGs^55^. However, we verified that the ARG carriers are among the most abundant PPGs in the cities where they are present. This is evidence that phages could be important agents of ARG dissemination in specific situations, as has been experimentally demonstrated in the viromes of retail food sources^56^ and according to seasons in a river ecosystem^57^.

We also investigated the presence of genes coding for depolymerases in the PPGs, since such enzymes allow phages to degrade the bacterial barriers consisting of polysaccharides and play crucial roles during tailed-phage infections^22^. Similar to the ARG result, only a small number of PPGs encode such genes. Among them, we found PPGs containing depolymerases that target bacterial capsules, a result that may be of significance in the context of ESKAPE pathogens, such as capsular strains of *Klebsiella pneumoniae*^58^.

We detected the presence of a CRISPR-Cas system in five PPGs, all of them with evidence level 3 or 4. The presence of CRISPR-Cas systems in phages has been reported before^8^. Phage CRISPR–Cas systems are used by phages to hijack host biosynthesis machinery^8^, though other functions are hypothesized^59^. The finding of such systems in phages is relevant because every new CRISPR-Cas system can potentially contain enzymes with properties that may improve the efficiency of genome editing in the laboratory^60^.

Overall, our results provide a richer description of the previously reported phage diversity and global distribution in urban environments of the MetaSUB samples, with a focus on the class *Caudoviricetes*. We found that the vast majority of PPGs (80%) are novel and the predicted bacterial hosts for these phages encompass the most common nosocomial multidrug-resistant pathogens. We believe that the PPG set here presented is a valuable resource for future studies to explore phage-derived depolymerases as new antibacterial therapies^24,25^ and might be useful for phage genome engineering to improve phage-based biocontrol approaches to tackle multidrug-resistant pathogens^20,21^.

## Methods

### Phage genome recovery

**Figure 5:**
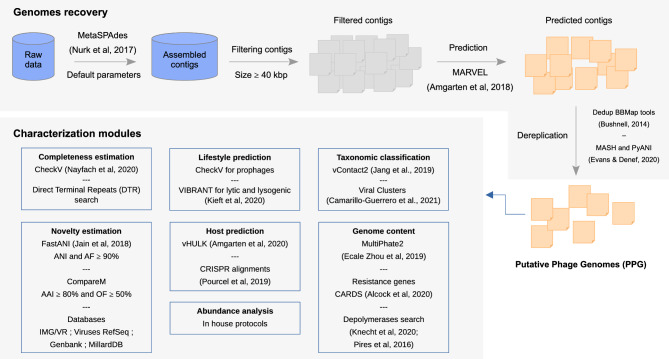
Pipeline applied to the MetaSUB contigs. Details of the pipeline steps and tools are described in the Methods section.

Raw FASTQ data from MetaSUB samples^38^ were individually assembled with metaSPAdes (v3.10.1)^61^. Because our goal was to maximize the number of complete phage genomes to be recovered, we used 40 kbp as the minimum required length of contigs. This threshold is based on^9^, which shows that 40 kbp is a minimum length for complete *Caudoviricetes* (tailed phages) genomes. The set of contigs satisfying this requirement was submitted to MARVEL^39^, using default parameters. All contigs predicted as phages were analyzed for redundancy (full size identical match); contigs entirely contained inside larger ones were also removed, using the tool dedupe from BBMap tools (parameter values s = 3 and e = 2; other parameters were default)^62^. An additional dereplication step was carried out using MASH and PyANI^63^. Initially we clustered all phage contigs with MASH using as threshold 99%. Then, within each cluster we aligned the contigs using PyANI with the flag ANIm. Finally, only the contig with the greatest size in each cluster was selected. The pipeline used for recovering PPGs is summarized in Fig. 5.

### Identification of direct terminal repeats (DTR) and completeness evaluation

Direct terminal repeats (DTR) were identified in contigs by searching exact matches longer than 30 bp at both ends of contigs. Completeness was assessed by CheckV^46^, a tool for assessing overall quality of viral metagenomic uncultivated virus using the MIUViG standards^64^. Default parameters were used.

### Bacteriophages lifestyle predictions

CheckV^46^ and VIBRANT^47^ were used to determine which PPGs could be classified as prophages. For the VIBRANT prophage classifier, all predictions with the suffix ‘_fragment’ were considered as putative prophages. Lifestyle of each PPG was predicted with the VIBRANT phages lifestyle predictor and Bacphlip^48^. All programs mentioned were used with default parameters.

### Clustering approaches for taxonomy evaluation

Taxonomic assignment of the PPGs was performed using vContact2 v0.11.3^40^ and Viral Cluster (VC)^32^. The amino acid sequences of genes predicted by the MultiPhate2 pipeline^65^ were submitted to vContact2 with default parameters and using as reference the Millard phage genome reference database^66^ (MillardDB; January 2023). After clustering, singleton (i.e. unclustered) genomes were excluded from the analysis. The PPGs clustered with reference phage genomes were considered to share the same family and genus of the cluster members that are reference phages. To cluster the PPGs with the VC method, we performed an all-against-all nucleotide alignment (blastn^67^) of PPGs. The following criteria were applied to alignments in order to select blastn hits for further processing: e-value ≤ 10^–5^, ≥ 90% nucleotide identity, and ≥ 75% coverage of the larger sequence against the smaller sequence. PPGs that resulted in hits according to the criteria above were clustered using the Markov Clustering algorithm (MCL) algorithm^68^ as implemented in Python3 (3.8.6). PPGs within a cluster were considered to share the same genus. Then, the clusters of vContact2 composed only by PPGs were compared with the VC clusters, and clusters with at least partial overlap of PPGs (at least one shared PPG) were analyzed with Cytoscape^69^.

### PPG similarity search

The PPGs were aligned with reference phages from different databases to assess the proportion of novel genomes. The comparisons were carried out between the 1,714 PPGs and the following databases: IMG/ VR portion of complete and High-quality phages (February 2023), RefSeq phage genomes of NCBI Virus (February 2023), Genbank phage genomes with more than 2 kbp length (February 2023) and MillardDB (February 2023;^66^). Table 3 reports the number of genomes used in the comparisons. Then each PPG was aligned using Average Nucleotide Identity (ANI) and Average Aminoacid Identity (AAI) with the tools FastANI and CompareM, respectively. For ANI alignments we evaluated a hit as significant when ANI and alignment fraction (AF) were ≥ 90%. AAI alignments with mean AAI greater than 80% and more than 50% of orthologous fraction were considered relevant. In addition, for the huge PPGs we carried out nucleotide alignments (blastn) between them and the jumbo and mega phages from the NCBI Virus database. An E-value of ≤ 10^–5^ was used as threshold.

**Table 3:** Number of phage genome sequences retrieved from the public repositories. NCBI Virus RefSeq (Huge Phages) sequences were dereplicated using dedupe from BBTools^62^.

Repository	Number of Phage Genomes
IMG/ VR	4509
NCBI Virus RefSeq	4679
NCBI Virus RefSeq (Huge phages)	657
NCBI GenBank	20,804
Millard lab database	24,519

### Genome content exploration

In a preliminary stage, we used Prokka^70^ to annotate contigs, followed by amino acid sequence alignments of the predicted genes against the pVOGs database of phage protein HMM profiles^44^ using hmmscan^71^ (default parameters). Hits were considered significant if the e-value was ≤ 10^–5^.

In a second stage, we decided to switch the PPG annotation process to the MultiPhate2 pipeline^65^, which uses Prodigal^72^, Glimmer^73^ and Phanotate^74^ as gene callers. Based on this new annotation, amino acid sequences of predicted open reading frames were aligned with blastp^75^ against the CARD database^76^. Hits with ≥ 80% identity and ≥ 85% alignment coverage were considered as significant.

We made a custom database of depolymerases using curated and classified sequences^20,22^. Then, the ORFs predicted with the MultiPhate2 pipeline^65^ were aligned against this custom database using blastp^75^. Hits that had ≥ 30% identity, ≥ 80% coverage and e-value ≤ 10^–5^ were considered significant. Then, the aligned ORFs were classified as putative depolymerases for Capsular polysaccharide (CPS), Exopolysaccharide (EPS) or Lipopolysaccharide (LPS) based on hits that passed the selection criteria.

CRISPR-Cas systems were searched with the CRISPRCas-Finder tool^77^.

### Host prediction

Host prediction was performed for PPGs using two different methods: CRISPR spacers linking^43^ and machine learning prediction using vHULK^41^. vHULK was run with default parameters. Predictions had to obey the following thresholds: score ≥ 0.3, entropy ≤ 2 and energy ≤ 5. Moreover, only predictions in which the putative host genus and putative species genus agreed were accepted. When multiple hosts were predicted for a given phage, only the one with the highest score was considered. PPGs were also queried against the complete database of CRISPR spacers (downloaded from CRISPRdb^43^ on June, 2020) using NCBI blastn toolkit^67^. Cutoffs for significant hits were e-value ≤ 0.001 and mismatches ≤ 2. Significant hits to a phage genome were considered a link between phage and host.

### Abundance quantification and exploration

The relative abundance for each PPG in a city was normalized based only on reads from that city that aligned to PPGs. When a city had more than one sample, the relative abundance was normalized by the total count of reads aligned to PPGs from all samples from that city. To explore the abundance of PPGs given the putative hosts we grouped the PPGs by their predicted hosts, then for each city the abundances of PPGs sharing the same host were added up. This process was carried out for both vHULK predictions (genus and species predictions) and CRISPR-Cas predictions, except when there were conflicting predictions for a given PPG by both methods. The same grouping strategy was applied for lifestyle predictions and ARG carriers. For ARG carriers we carried out Welch's t-test before checking for normality using the Shapiro–Wilk test. Also, a permutation test was applied because of the unequal size of ARG carriers and non-ARG carriers. We re-sampled 1000 times collecting the same number of ARG carriers in the group of non-ARG carriers for each city in each round; the mean abundance of these re-sampled groups was calculated and compared with the mean abundance of ARG carriers of each city. A p-value threshold of ≤ 0.05 was used for all the statistical tests applied.

The clustering of cities based on the presence and relative abundance of PPGs was carried out using an agglomerative cluster algorithm with 12 different combinations between affinity and linkage criteria. We used the agglomerative clustering algorithm implementation of the scikit-learn package version 1.2.2^78^. Three affinity metrics (Euclidean, L1, Manhattan and Cosine) were combined with four linkage criteria (Complete, Average and Single) to cluster the cities. That approach was chosen since the analysis of different combinations of affinity and linkage criteria has been reported to be more reliable^79^.

### Supplementary Information

Supplementary Information.

## Data Availability

All Putative Phage Genomes (PPGs) listed in Supplementary Table S1 are publicly available in GenBank. Their accession numbers can be found in Supplementary Table S10.

## Abbreviations

AMG: Auxiliary metabolic gene
ARG: Antibiotic resistance gene
CRISPR-Cas: Clustered regularly interspaced short palindromic repeats CRISPR-associated protein
MetaSUB: The international metagenomics and metadesign of subways and urban biomes
PPG: Putative phage genome
pVOG: Prokaryotic virus orthologous groups
