# Publisher Correction: Discovery and description of novel phage genomes from urban microbiomes sampled by the MetaSUB consortium

**DOI:** 10.1038/s41598-024-60777-1

**Published:** 2024-05-10

**Authors:** Vinicius S. Flores, Deyvid E. Amgarten, Bruno Koshin Vázquez Iha, Krista A. Ryon, David Danko, Braden T. Tierney, Christopher Mason, Aline Maria da Silva, João Carlos Setubal

**Affiliations:** 1 Departamento de Bioquímica, Instituto de Química, Universidade de São Paulo, São Paulo, 05508-000 Brazil; 2 Weill Cornell Medicine, New York, NY USA; 3 Biotia, New York, NY USA; 4 Harvard Medical School, Cambridge, MA USA

Correction to: *Scientific Reports* 10.1038/s41598-024-58226-0, published online 04 April 2024

The original version of this article contained an error.

In the original version of this Article, an incorrect version of the Supplementary Information file was mistakenly typeset.

The incorrect Supplementary Information file appears below.

The Supplementary Information file now accompanies the original Article.

### Supplementary Information

Supplementary Information.

