# Supplementary material for: Discovery and description of novel phage genomes from urban microbiomes sampled by the MetaSUB consortium
Source: Sci Rep. 2024 Apr 4;14:7913. doi: 10.1038/s41598-024-58226-0 (PMC10994904; doi:10.1038/s41598-024-58226-0)
Supplement: Supplementary file 1 — Supplementary Information. [file 41598_2024_58226_MOESM1_ESM.pdf]

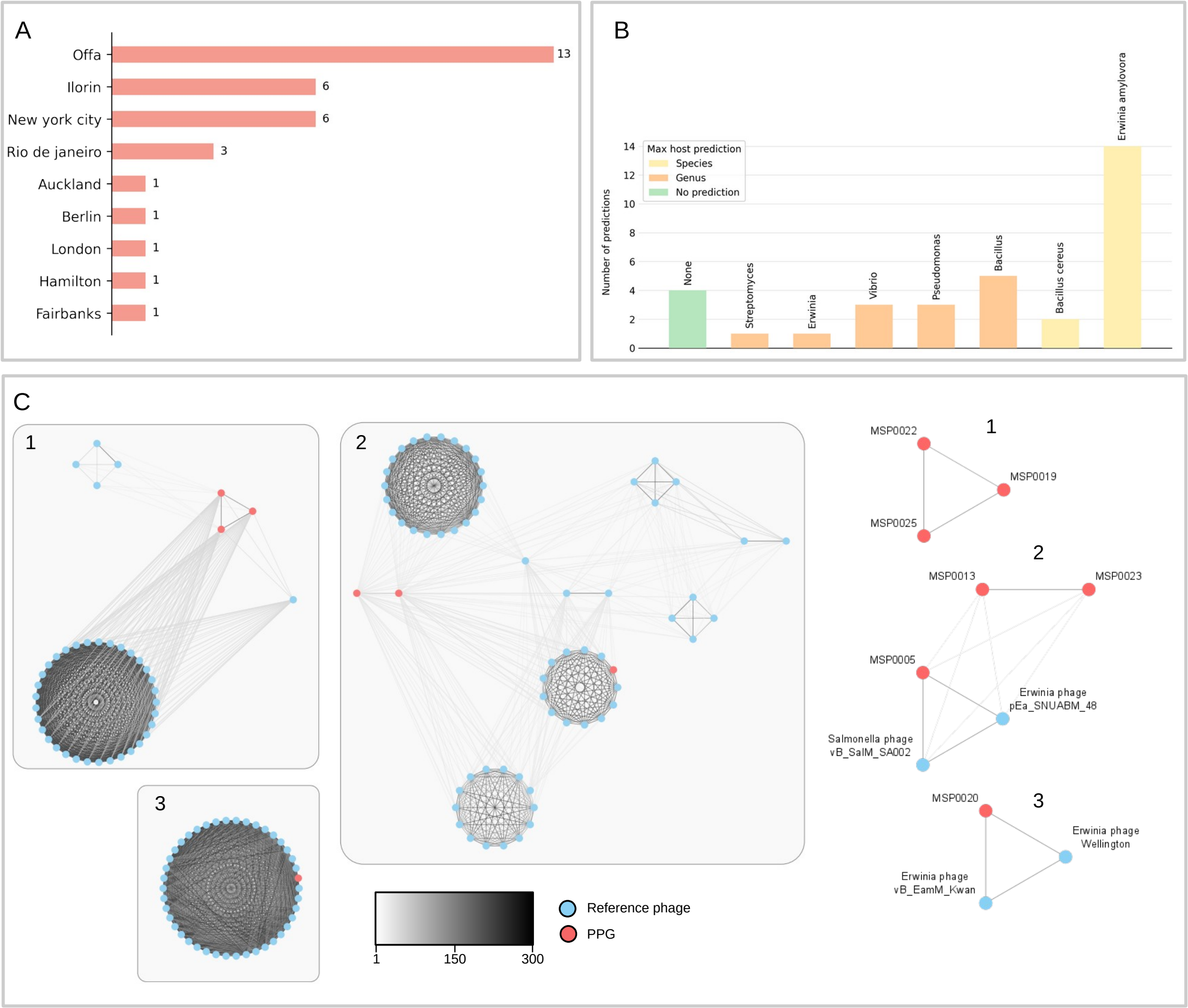

Supplementary Figure 1. Exploration of huge PPGs recovered from MetaSUB samples. (A) Distribution of huge PPGs through cities. (B) Distribution of putative hosts predicted by vHULK for the recovered huge PPGs. (C) vContact2 clustering of huge PPGs and NCBI Virus huge bacteriophages. On the left is shown a representation of all clusters. On the right are shown the parts of the clusters that include the huge PPGs.

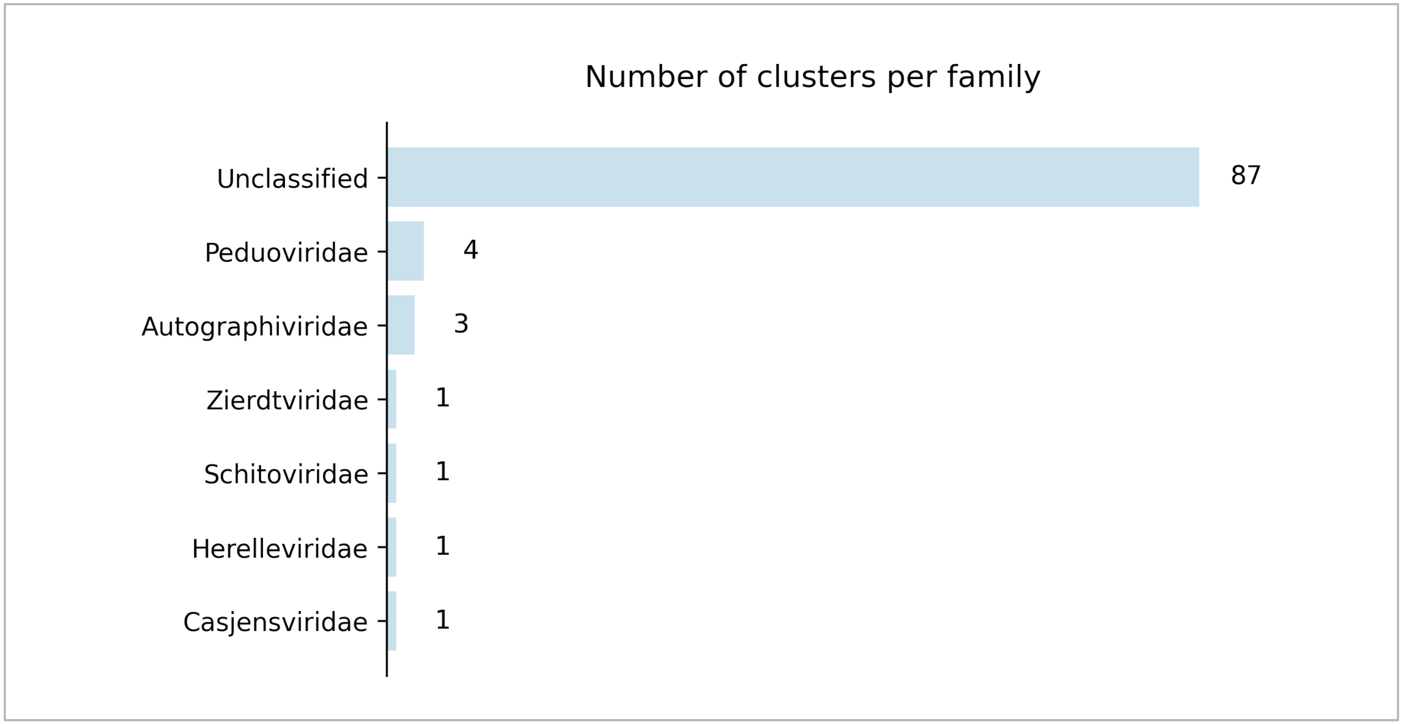

Supplementary Figure S2. Number of clusters per family. Number of clusters assigned with a family level classification. Most reference phages have no family level classification.

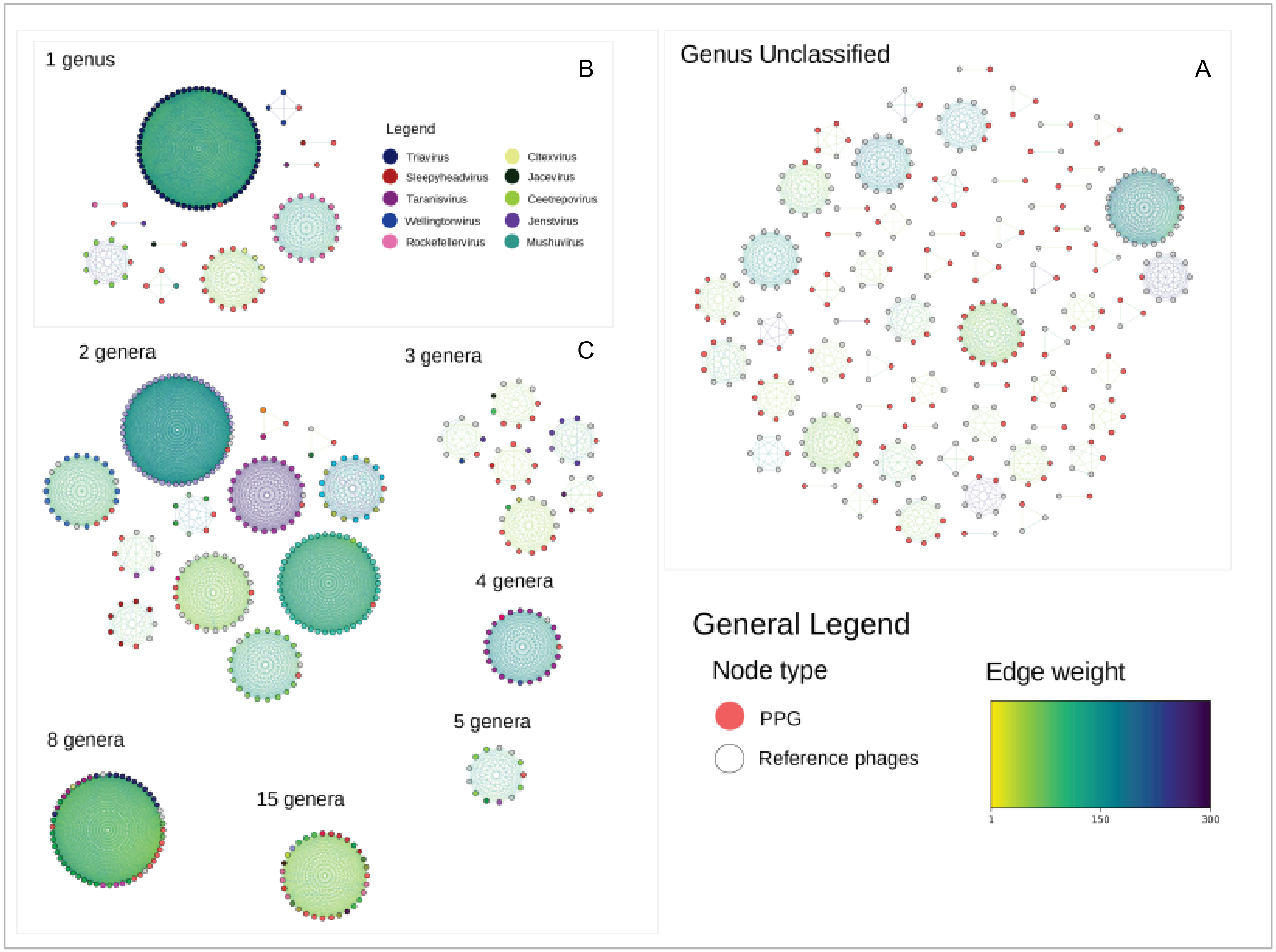

Supplementary Figure S3. PPGs clustered with vContact2 reference phages. A total of 207 recovered genomes were clustered in 98 clusters with vContact2 reference phages from Millard database (Jan. 2023). Each node is a phage genome, in which red nodes are PPGs and the colored nodes are the reference phages. A color gradient shows edge intensity between nodes. (A) Clusters with referencce phages with unclassified genus. (B) Clusters with reference phages of the same genus. (C) Clusters with reference phages from different genera.

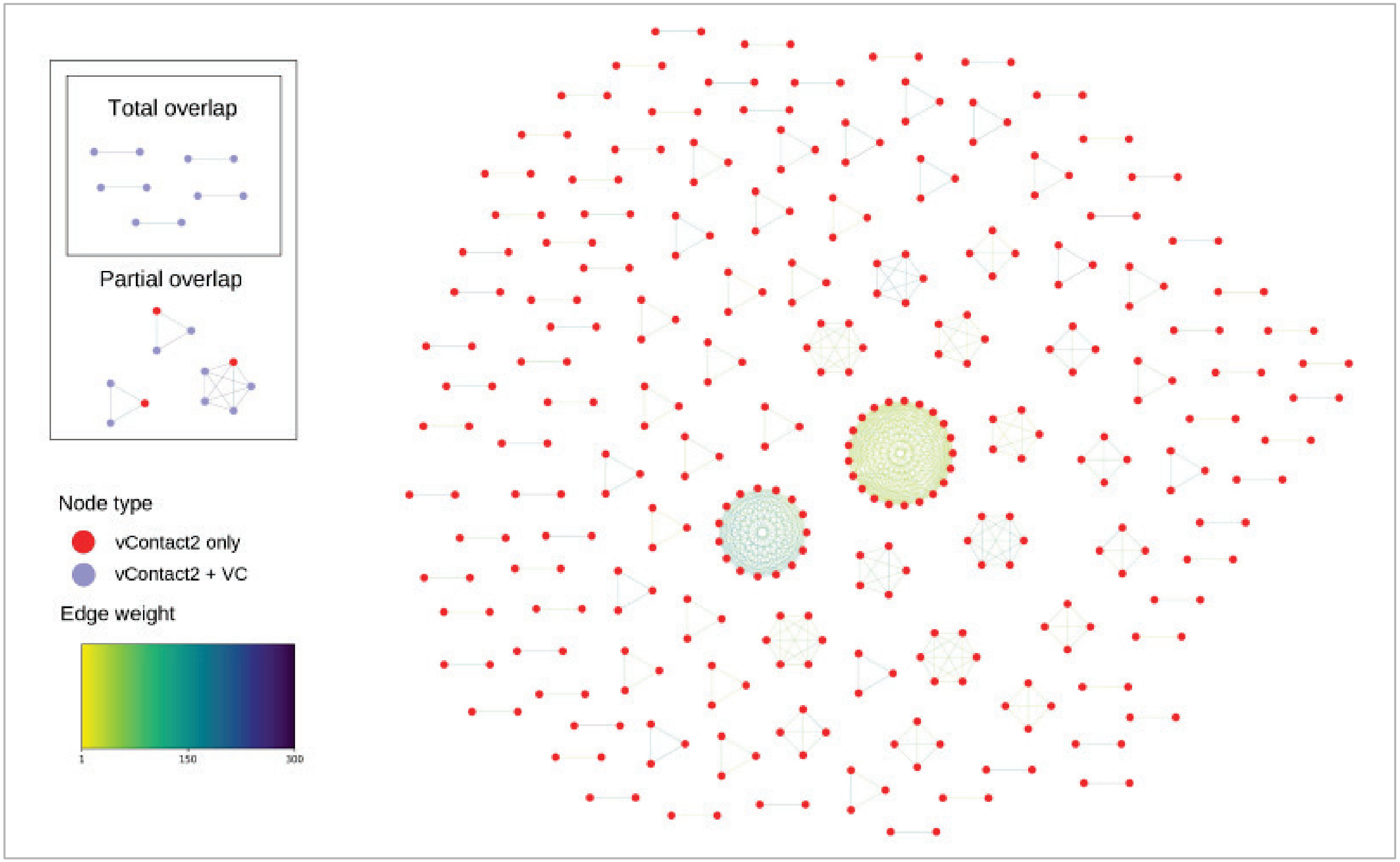

Supplementary Figure S4. Clusters with total, partial or none overlap between vContact2 and VC methodology. All recovered PPGs were clustered using vContact2 and VC methodology. The clusters with complete or partial overlap between vContact2 and VC methodology were presented in the left panel. When a cluster shares a partial overlap between the approaches, the nodes in this partial overlap portion span a high vContact2 edge weight.

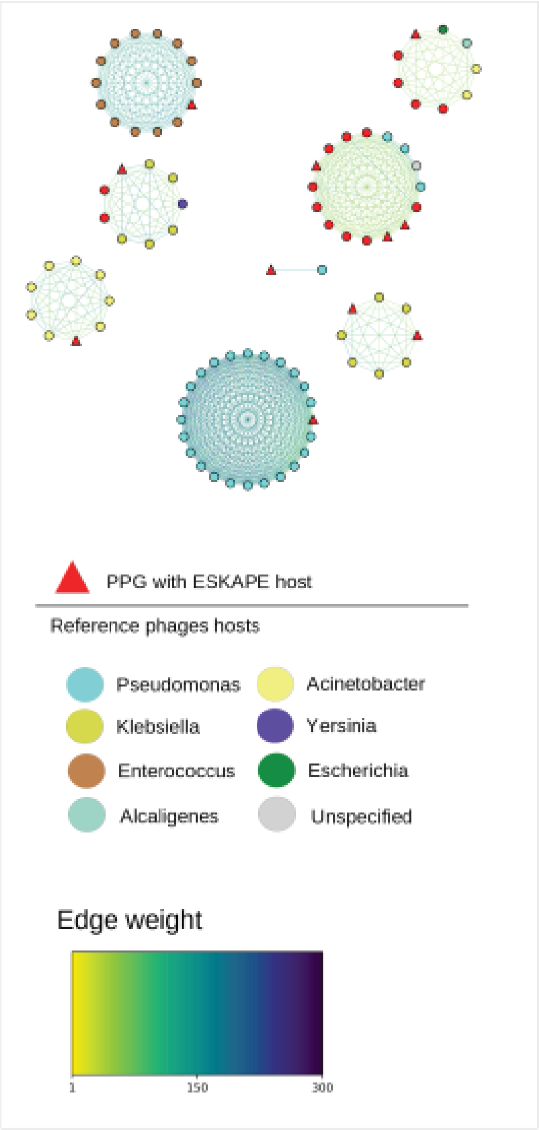

Supplementary Figure S5. Clusters with the 11 PPGs assigned hosts from ESKAPE group. All recovered PPGs were clustered using vContact2 then filtered for clusters whose hosts assigned for reference phages are in the ESKAPE group.

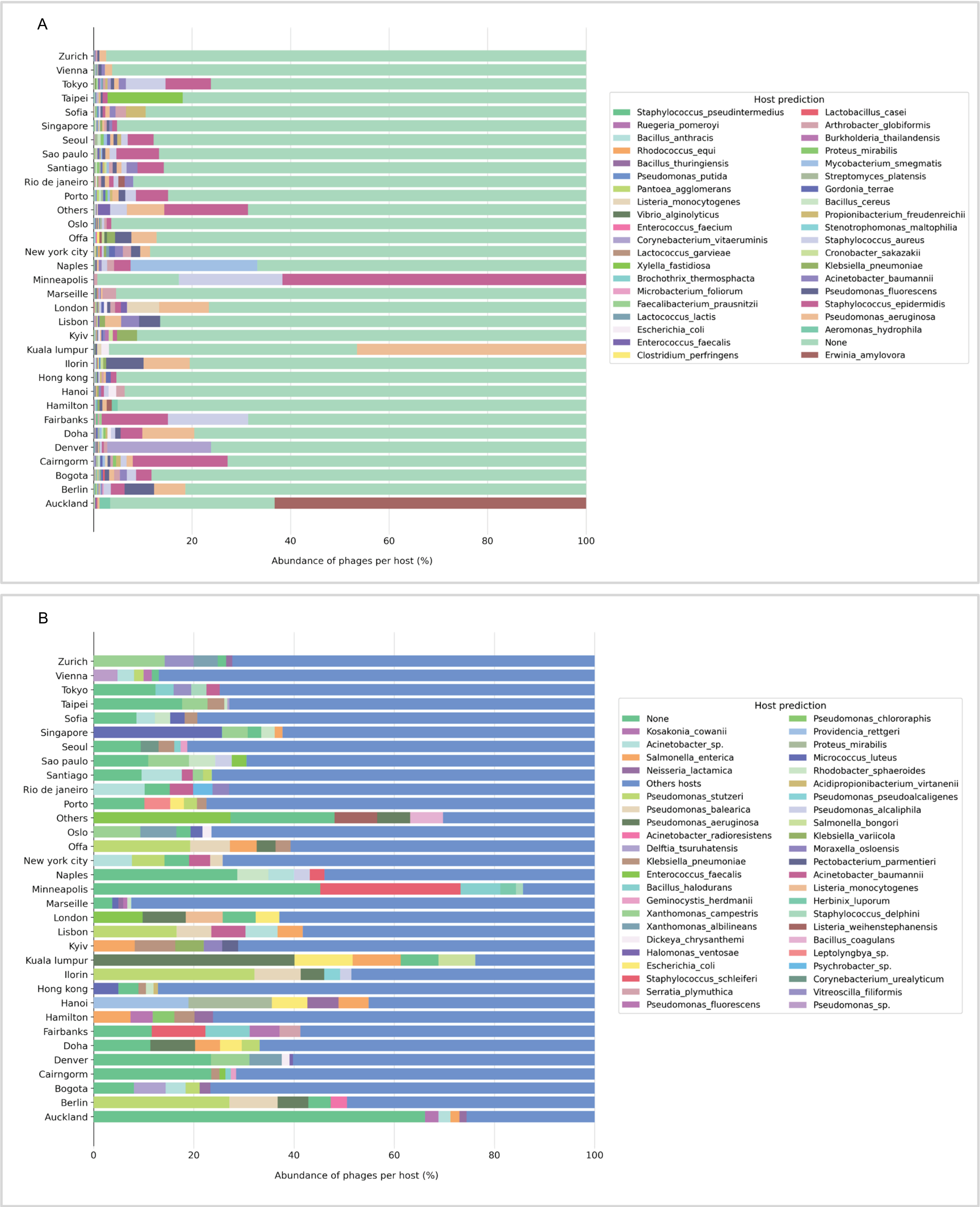

Supplementary Figure S6. Abundance of PPGs per host. (A) Abundance of PPGs per host species predicted by vHULK. (B) Abundance of PPGs per host species predicted by CRISPR-Cas alignments

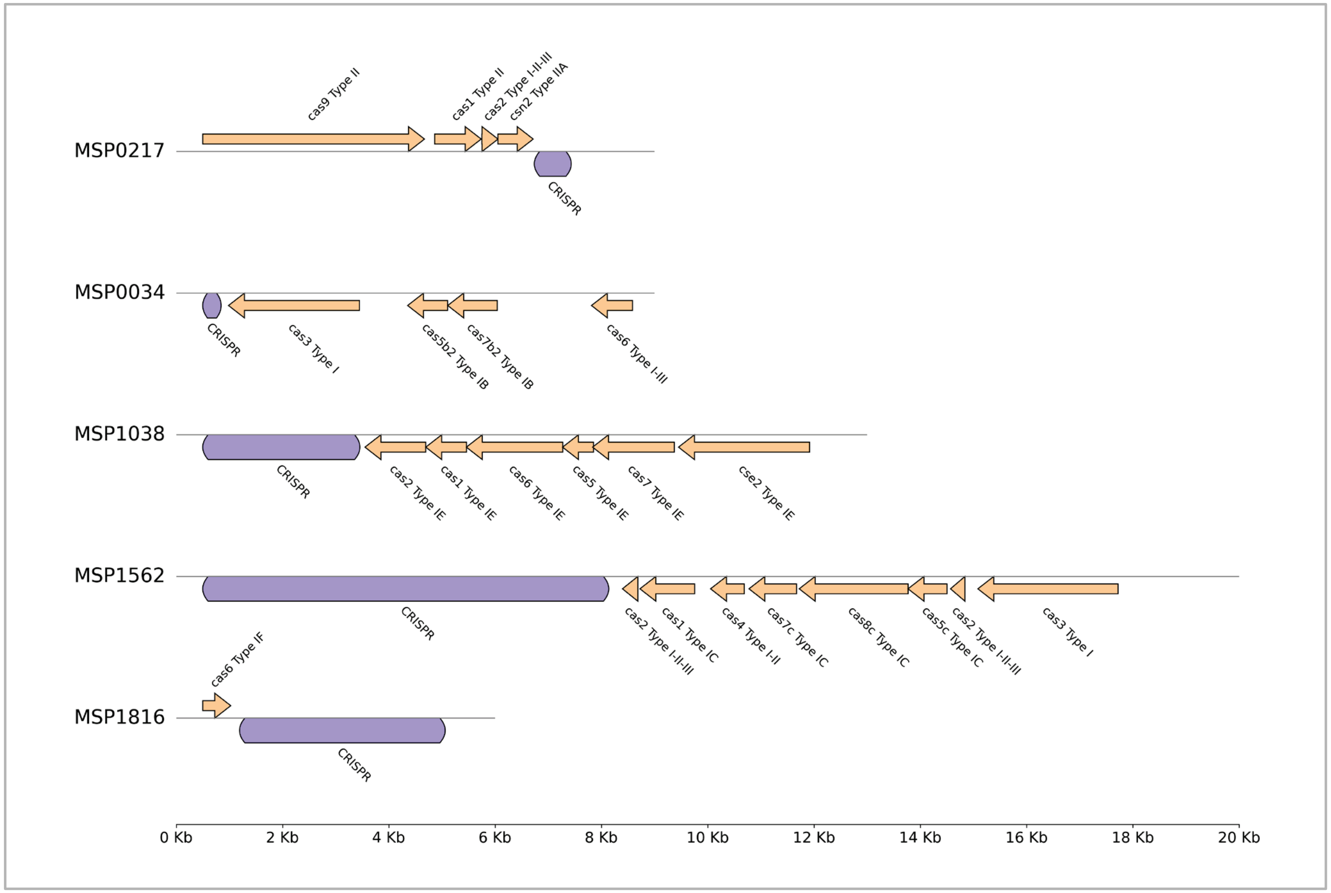

Supplementary Figure S7. CRISPR-Cas system found in PPGs. CRISPR-Cas spacers are represented with a rounded box colored in purple. Each arrow indicates a Cas gene.

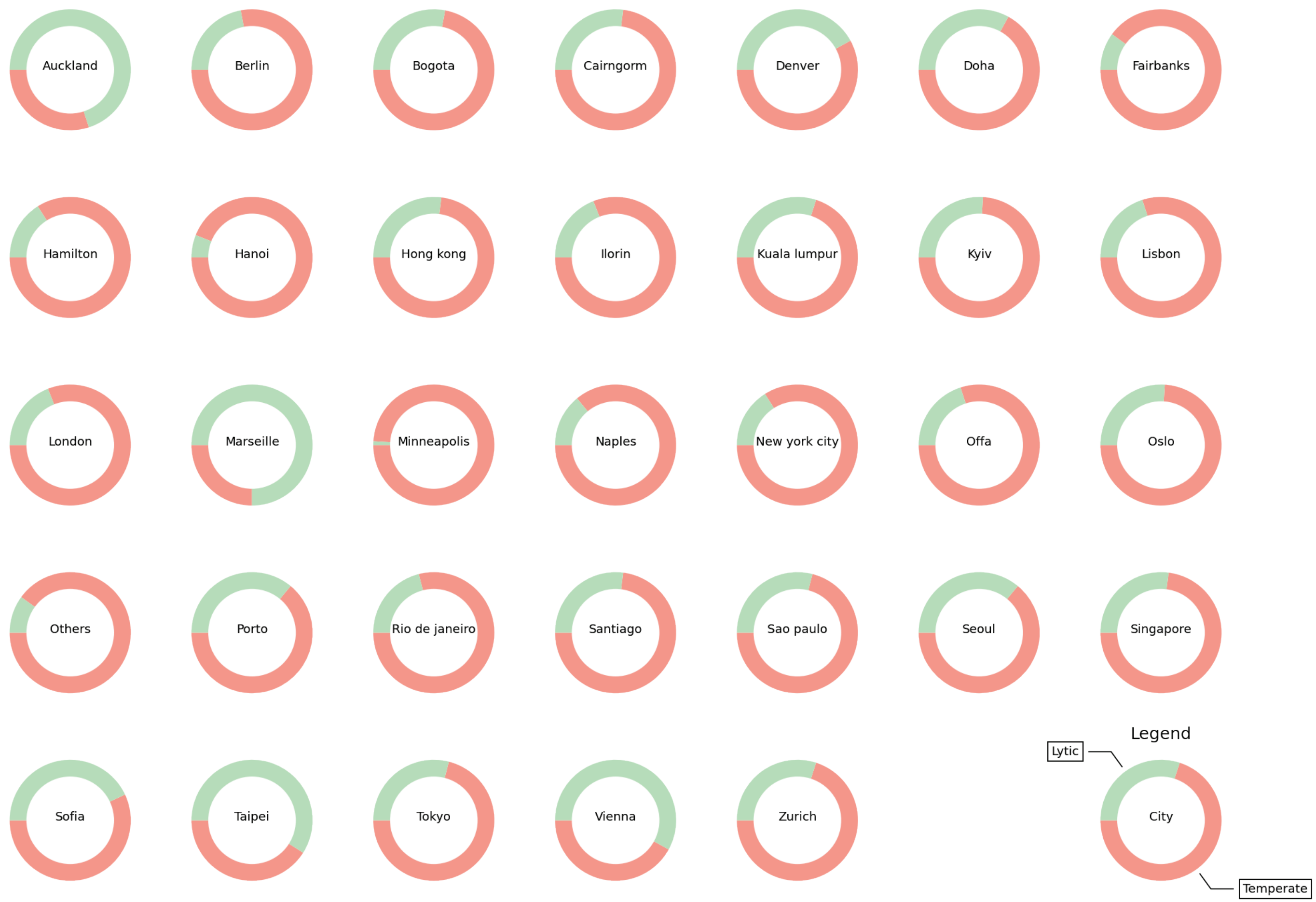

Supplementary Figure S8. Abundance of PPGs grouped by lifestyles for each city. The lifestyle predictions and abundance quantifications were carried out as described in Material and Methods. For this analysis only Bacphlip predictions were used, despite the predictions of VIBRANT and CheckV.

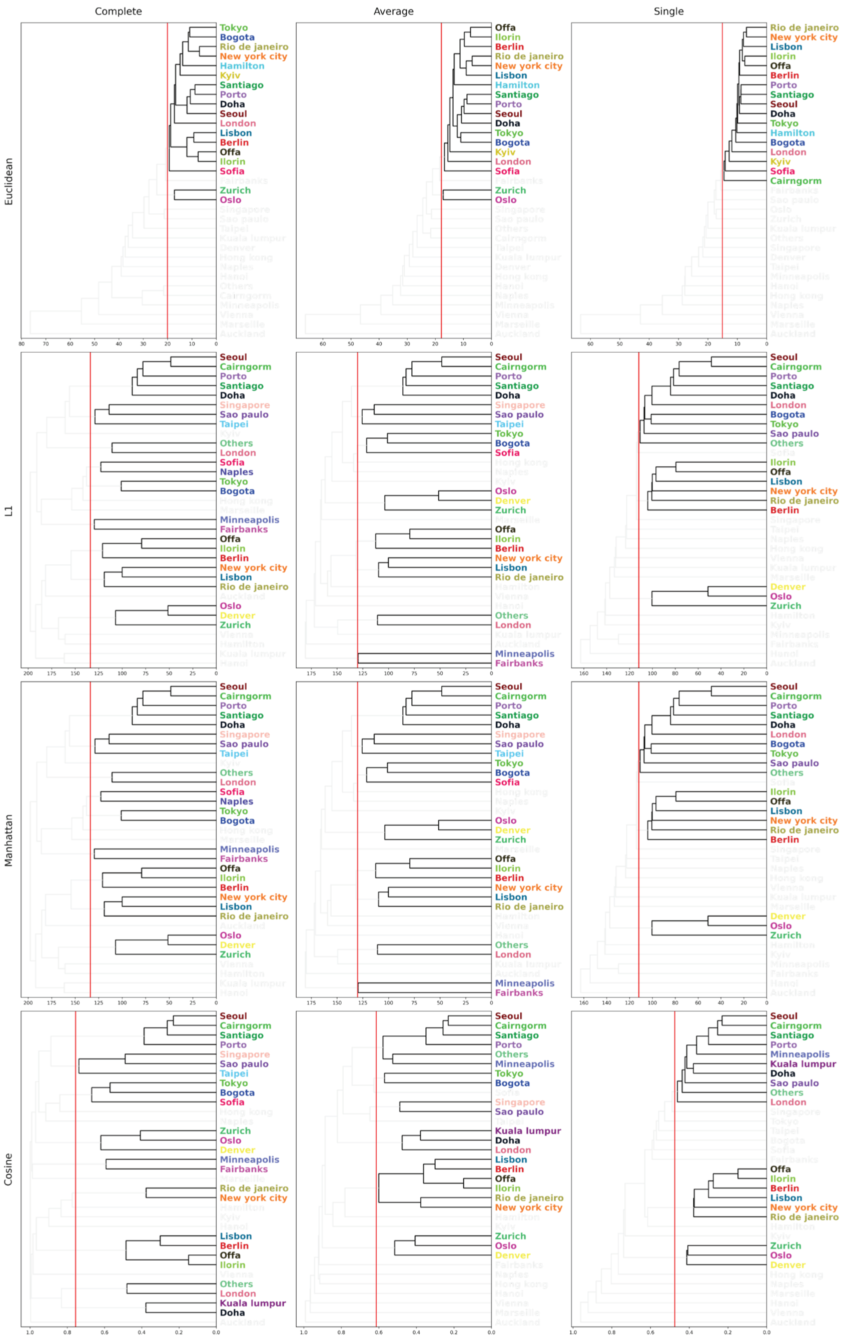

Supplementary Figure S9. Clustering cities based on bacteriophage abundances. Four clustering algorithms (y-axis) with three different metrics (x-axis) were applied for grouping the sampled cities based on PPGs abundances quantification. The red line marks the median value for each metric used per algorithm. The clusters with values above the median were highlighted.

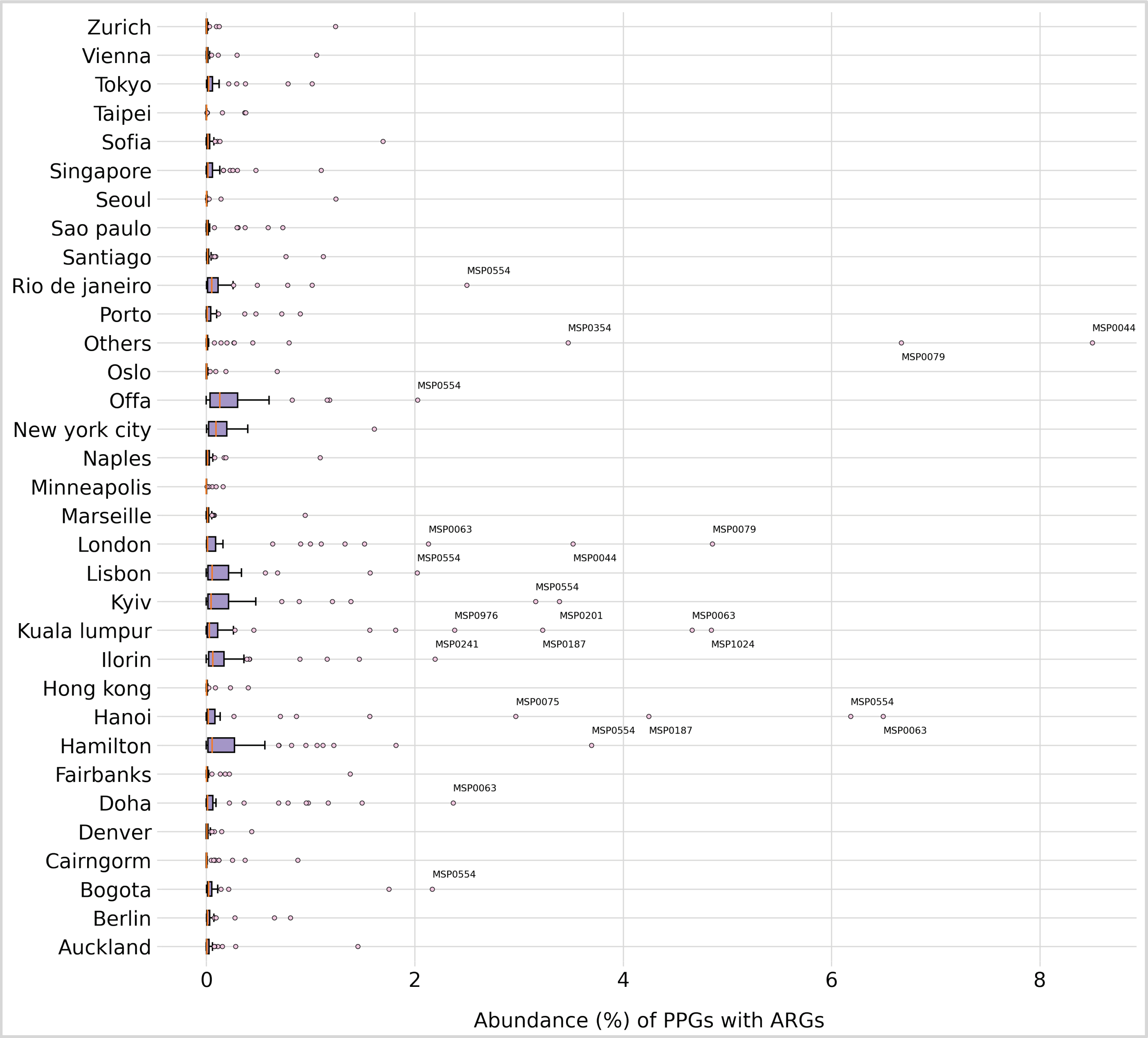

Supplementary Figure S10. Abundances of PPGs identified as carriers of antibiotic resistance genes (ARGs). Abundance distribution of PPGs identified with ARGs per city. The PPGs with more than 2 % of abundance were highlighted by showing their names.

Supplementary Table S1. Summary of PPGs features.

PPG	Phage (Sample name + unique sequential id)	Size (bp)	DTR	Provirus	host_vHULK_genus	host_vHULK_species	host_CRISPR	Lifestyle (Bacphlip)	Novelty	Cosmopolitanism (# cities appeared)	75_most_common
MSP0001	haib17CEM4890_H75CGCCXY_SL263641.1	572750	yes	No	Agrobacterium	None		virulent/lytic	Yes	30.0	0.0
MSP0002	haib17CEM4890_HMCMJCCXY_SL335807.1	376117	no	No				virulent/lytic	Yes	21.0	0.0
MSP0003	haib17CEM4890_H7KYMCCXY_SL273060.1	336762	yes	No			Streptococcus sp. I-G2	prophage/temperate/lysogenic	No	33.0	0.0
MSP0004	haib17CEM4890_H2NYMCCXY_SL254829.21	311039	no	Yes				prophage/temperate/lysogenic	Yes	13.0	0.0
MSP0005	haib17CEM4890_H75CGCCXY_SL263663.3	304729	yes	No	Erwinia	Erwinia_amylovora		virulent/lytic	Yes	17.0	0.0
MSP0006	haib17CEM4890_H2NYMCCXY_SL254846.1	290355	no	No	Vibrio	Vibrio_alginolyticus		virulent/lytic	Yes	12.0	0.0
MSP0007	haib17CEM4890_H2NYMCCXY_SL254843.29	271836	yes	No	Erwinia	None		virulent/lytic	Yes	10.0	0.0
MSP0008	haib17CEM5106_HCY5HCCXY_SL270937.9	269557	yes	No	Erwinia	Erwinia_amylovora		virulent/lytic	Yes	14.0	0.0
MSP0010	haib17CEM4890_H2NYMCCXY_SL254818.11	263436	no	No				virulent/lytic	Yes	22.0	0.0
MSP0011	haib17CEM4890_H2NYMCCXY_SL254830.31	261774	yes	No	Erwinia	None		virulent/lytic	Yes	11.0	0.0
MSP0012	haib17CEM4890_H7KYMCCXY_SL273056.1	260670	no	No	Microbacterium	Microbacterium_foliorum		virulent/lytic	Yes	20.0	0.0
MSP0013	haib17CEM4890_H2NYMCCXY_SL254772.24	258704	yes	No	Erwinia	None		virulent/lytic	Yes	21.0	0.0
MSP0014	haib17CEM4890_H2NYMCCXY_SL254830.32	255896	yes	No	Erwinia	None		virulent/lytic	Yes	18.0	0.0
MSP0015	haib17CEM4890_H2NYMCCXY_SL254814.4	252585	yes	No	Erwinia	Erwinia_amylovora		virulent/lytic	Yes	17.0	0.0
MSP0016	haib17DB4959_H3MGVCCXY_SL259882.6	250778	yes	No				prophage/temperate/lysogenic	Yes	14.0	0.0
MSP0017	haib17CEM4890_H2NYMCCXY_SL254855.29	250179	yes	No	Erwinia	Erwinia_amylovora		virulent/lytic	Yes	20.0	0.0
MSP0018	haib17CEM4890_H2NYMCCXY_SL254855.31	249065	no	Yes				prophage/temperate/lysogenic	Yes	14.0	0.0
MSP0019	haib17KIU4866_H7HJMCCXY_SL272745.11	243203	yes	No	Dickeya	None		virulent/lytic	Yes	20.0	0.0
MSP0020	haib17CEM4890_H2NYMCCXY_SL254826.5	242627	yes	No	Erwinia	Erwinia_amylovora		virulent/lytic	Yes	9.0	0.0
MSP0022	haib17CEM4890_H2NYMCCXY_SL254818.16	241516	yes	No	Dickeya	None		virulent/lytic	Yes	20.0	0.0
MSP0023	haib17CEM4890_H2NYMCCXY_SL254807.13	237329	yes	No	Erwinia	None		virulent/lytic	Yes	6.0	0.0
MSP0024	haib17CEM4890_H2NYMCCXY_SL254861.1	231242	yes	No				virulent/lytic	Yes	6.0	0.0
MSP0025	haib17KIU4866_H7HJMCCXY_SL272745.13	230175	no	No	Dickeya	None		virulent/lytic	Yes	17.0	0.0
MSP0026	haib17CEM5106_HCV72CCXY_SL269793.22	226068	no	Yes	Klebsiella	Klebsiella_pneumoniae	Salmonella enterica subsp. enterica serovar Waycross	prophage/temperate/lysogenic	No	33.0	0.0
MSP0027	haib17KIU4866_H7HJMCCXY_SL272761.13	223601	no	Yes				prophage/temperate/lysogenic	Yes	16.0	0.0
MSP0028	haib17CEM4890_H2NYMCCXY_SL254830.39	222386	yes	No	Vibrio	Vibrio_alginolyticus		virulent/lytic	Yes	19.0	0.0
MSP0029	haib17KIU4866_H7HJMCCXY_SL272759.1	220809	yes	No				virulent/lytic	Yes	25.0	0.0
MSP0030	haib17CEM4890_H75CGCCXY_SL263638.11	220684	no	No	Erwinia	Erwinia_amylovora		virulent/lytic	Yes	12.0	0.0
MSP0031	haib17CEM5106_HCY5JCCXY_SL269588.9	220558	no	Yes			Lactobacillus curvatus	prophage/temperate/lysogenic	Yes	29.0	0.0
MSP0032	haib17CEM4890_H2NYMCCXY_SL254854.24	219620	yes	No				virulent/lytic	Yes	23.0	0.0
MSP0033	haib17KIU4866_H7HJMCCXY_SL272745.15	219144	no	No	Dickeya	None		virulent/lytic	Yes	14.0	0.0
MSP0034	haib17CEM4890_H2NYMCCXY_SL254841.29	212281	no	No			Clostridium botulinum	prophage/temperate/lysogenic	Yes	11.0	0.0
MSP0035	haib17KIU4866_H7HJMCCXY_SL272803.21	200990	no	Yes				prophage/temperate/lysogenic	Yes	18.0	0.0
MSP0036	haib17CEM5106_HCY5HCCXY_SL270996.37	199672	no	Yes	Listeria	Listeria_monocytogenes	Listeria monocytogenes	prophage/temperate/lysogenic	No	22.0	1.0
MSP0037	haib17KIU4866_H7HJMCCXY_SL272769.1	189859	no	No				virulent/lytic	Yes	17.0	0.0
MSP0038	haib17CEM4890_HKC32ALXX_SL254693.38	188252	no	Yes			Caldanaerobacter subterraneus subsp. tengcongensis MB4	prophage/temperate/lysogenic	Yes	29.0	0.0
MSP0040	haib17KIU4866_H7HJMCCXY_SL272783.26	185019	no	No				prophage/temperate/lysogenic	Yes	12.0	0.0
MSP0041	haib17CEM5106_HCV72CCXY_SL269765.38	184925	no	Yes	Gordonia	Gordonia_terrae	Rhodococcus sp. ABRD24	prophage/temperate/lysogenic	Yes	28.0	0.0
MSP0042	haib17CEM5106_HCV72CCXY_SL269730.36	184803	no	Yes			Sporosarcina sp. PTS2304	prophage/temperate/lysogenic	Yes	26.0	0.0
MSP0043	haib17CEM5106_HCY5HCCXY_SL270918.20	184773	no	Yes			Rhodobacter sphaeroides	prophage/temperate/lysogenic	Yes	32.0	0.0
MSP0044	haib17CEM5106_HCY5JCCXY_SL269534.32	181507	no	Yes			Listeria weihenstephanensis	prophage/temperate/lysogenic	No	28.0	0.0
MSP0045	haib17CEM4890_H75CGCCXY_SL263688.45	180388	no	Yes			Salmonella enterica subsp. enterica serovar Wandsworth str.	prophage/temperate/lysogenic	Yes	33.0	1.0
MSP0046	haib17KIU4866_H7HJMCCXY_SL272773.32	177259	no	Yes			Leclercia adecarboxylata	prophage/temperate/lysogenic	No	33.0	0.0
MSP0047	haib17CEM4890_H2NYMCCXY_SL254782.56	175560	no	Yes			Klebsiella pneumoniae subsp. pneumoniae	prophage/temperate/lysogenic	Yes	33.0	1.0
MSP0048	haib17KIU4866_H7HJMCCXY_SL272774.2	173561	no	No	Erwinia	Erwinia_amylovora		virulent/lytic	Yes	12.0	0.0
MSP0049	haib17CEM4890_HKC32ALXX_SL254712.8	171178	no	Yes			Delftia tsuruhatensis	prophage/temperate/lysogenic	Yes	33.0	0.0
MSP0050	haib17CEM4890_H2NYMCCXY_SL254770.20	169080	no	Yes				prophage/temperate/lysogenic	Yes	16.0	0.0
MSP0051	haib17CEM4890_HKC32ALXX_SL254706.35	167836	yes	Yes			Hungateiclostridium thermocellum DSM 1313	prophage/temperate/lysogenic	Yes	9.0	0.0
MSP0052	haib17CEM4890_H2NYMCCXY_SL254826.16	167518	yes	No			Enterococcus durans	prophage/temperate/lysogenic	Yes	6.0	0.0
MSP0054	haib17CEM4890_H2NYMCCXY_SL254852.30	160372	no	Yes	Faecalibacterium	Faecalibacterium_prausnitzii		prophage/temperate/lysogenic	Yes	14.0	0.0
MSP0055	haib17CEM4890_H2NYMCCXY_SL254772.76	160259	no	No				prophage/temperate/lysogenic	Yes	31.0	0.0
MSP0056	haib17CEM4890_H75CGCCXY_SL263690.12	160172	no	Yes			Pseudomonas sp. CMR5c	prophage/temperate/lysogenic	Yes	33.0	0.0
MSP0057	haib17CEM4890_H2NYMCCXY_SL254829.54	159868	yes	No			Clostridium botulinum H04402 065	prophage/temperate/lysogenic	Yes	9.0	0.0
MSP0058	haib17CEM5106_HCVMTCCXY_SL269599.30	159093	no	Yes			Pseudomonas stutzeri	prophage/temperate/lysogenic	Yes	33.0	1.0
MSP0059	haib17CEM4890_HKC32ALXX_SL254764.81	158674	no	Yes				prophage/temperate/lysogenic	Yes	17.0	0.0
MSP0060	haib17KIU4866_H7HJMCCXY_SL272774.3	158408	no	No	Bacillus	Bacillus_thuringiensis	Paenibacillus crassostreae	prophage/temperate/lysogenic	Yes	7.0	0.0
MSP0061	haib17CEM4890_H2NYMCCXY_SL254819.69	158203	no	Yes			Pseudomonas balearica DSM 6083	prophage/temperate/lysogenic	Yes	33.0	1.0
MSP0062	haib17KIU4866_HMCMJCCXY_SL335947.29	157562	no	Yes	Pseudomonas	Pseudomonas_aeruginosa	Pseudomonas aeruginosa 19BR	prophage/temperate/lysogenic	Yes	33.0	1.0
MSP0063	haib17CEM4890_HKC32ALXX_SL254726.5	155965	no	Yes			Escherichia coli	prophage/temperate/lysogenic	No	33.0	1.0
MSP0064	haib17CEM5106_HCCGHCCXY_SL270210.1	155131	no	No	Arthrobacter	Arthrobacter_globiformis		virulent/lytic	Yes	27.0	0.0
MSP0065	haib17CEM4890_H2NYMCCXY_SL254852.32	153533	no	Yes	Staphylococcus	Staphylococcus_epidermidis	Staphylococcus schleiferi	prophage/temperate/lysogenic	No	32.0	0.0
MSP0066	haib17CEM4890_H2NYMCCXY_SL254770.25	152746	yes	No				virulent/lytic	Yes	12.0	0.0
MSP0067	haib17CEM4890_HKC32ALXX_SL254705.65	152455	no	Yes			Dickeya dianthicola IPO 980	prophage/temperate/lysogenic	No	32.0	0.0
MSP0068	haib17CEM4890_H2NYMCCXY_SL254782.72	152226	yes	No				prophage/temperate/lysogenic	Yes	8.0	0.0
MSP0069	haib17CEM4890_H2NYMCCXY_SL254782.73	151871	no	Yes				prophage/temperate/lysogenic	Yes	13.0	0.0

Table S1

MSP0070	haib18CEM5453_HMGN5CCXY_SL344676.1	151238	no	No			Klebsiella pneumoniae	prophage/temperate/lysogenic	Yes	32.0	1.0
MSP0071	haib17CEM4890_H2NYMCCXY_SL254772.86	151055	yes	Yes				prophage/temperate/lysogenic	Yes	6.0	0.0
MSP0072	haib17CEM4890_H2NYMCCXY_SL254818.48	150270	yes	No	Enterococcus	Enterococcus_faecium	Bacillus thuringiensis serovar indiana	virulent/lytic	Yes	12.0	0.0
MSP0073	haib17KIU4866_H7HJMCCXY_SL272745.24	149315	no	Yes			Kosakonia cowanii JCM 10956 = DSM 18146	virulent/lytic	Yes	30.0	0.0
MSP0074	haib18CEM5453_HMC2KCCXY_SL336590.2	148901	no	Yes				prophage/temperate/lysogenic	Yes	33.0	0.0
MSP0075	haib17KIU4866_H7HJMCCXY_SL272789.11	148646	no	No				prophage/temperate/lysogenic	Yes	33.0	0.0
MSP0076	haib17KIU4866_H7HJMCCXY_SL272771.12	148555	no	Yes			Pluralibacter gergoviae	prophage/temperate/lysogenic	Yes	31.0	0.0
MSP0077	haib17KIU4866_HMCMJCCXY_SL335937.1	148399	no	No				virulent/lytic	Yes	17.0	0.0
MSP0078	haib17KIU4866_H7HJMCCXY_SL272816.29	147623	no	Yes	Pseudomonas	Pseudomonas_fluorescens	Pseudomonas stutzeri	prophage/temperate/lysogenic	Yes	33.0	0.0
MSP0079	haib17CEM5106_HCY5HCCXY_SL270996.63	147423	no	Yes			Enterococcus faecalis DENG1	prophage/temperate/lysogenic	No	33.0	1.0
MSP0081	haib17CEM4890_H75CGCCXY_SL263637.8	146953	no	Yes				prophage/temperate/lysogenic	Yes	18.0	0.0
MSP0082	haib17CEM4890_H75CGCCXY_SL263678.34	146358	no	Yes			Salmonella enterica subsp. diarizonae	prophage/temperate/lysogenic	No	33.0	1.0
MSP0083	haib17CEM4890_H7KYMCCXY_SL273067.9	146067	no	Yes	Staphylococcus	Staphylococcus_epidermidis	Staphylococcus schleiferi	prophage/temperate/lysogenic	No	32.0	0.0
MSP0084	haib18CEM5526_HMGTJCCXY_SL342622.32	145959	no	Yes	Pseudomonas	Pseudomonas_aeruginosa	Pseudomonas aeruginosa DK2	prophage/temperate/lysogenic	No	33.0	1.0
MSP0085	haib17CEM5106_HCVMTCCXY_SL269548.16	145192	no	Yes	Brochothrix	Brochothrix_thermosphacta		prophage/temperate/lysogenic	No	21.0	0.0
MSP0086	haib17CEM4890_HMCMJCCXY_SL335783.12	145159	no	Yes	Pseudomonas	Pseudomonas_aeruginosa		prophage/temperate/lysogenic	Yes	30.0	1.0
MSP0088	haib17KIU4866_H7HJMCCXY_SL272800.21	143585	no	Yes				prophage/temperate/lysogenic	Yes	18.0	0.0
MSP0089	haib18CEM5453_HMGW3CCXY_SL342291.36	142407	no	Yes	Pseudomonas	Pseudomonas_aeruginosa	Pseudomonas aeruginosa 19BR	virulent/lytic	No	33.0	1.0
MSP0090	haib17CEM4890_H75CGCCXY_SL263716.15	142223	no	Yes			Escherichia coli	virulent/lytic	Yes	33.0	1.0
MSP0091	haib17CEM4890_H2NYMCCXY_SL254783.86	140295	no	Yes			Aminipila sp. JN-18	prophage/temperate/lysogenic	Yes	6.0	0.0
MSP0094	haib17CEM4890_HMCMJCCXY_SL335842.1	139326	no	No				virulent/lytic	Yes	20.0	0.0
MSP0095	haib17CEM4890_H2NYMCCXY_SL254783.89	139259	yes	No				virulent/lytic	Yes	11.0	0.0
MSP0097	haib17CEM4890_H2NYMCCXY_SL254850.7	139153	yes	No				virulent/lytic	Yes	12.0	0.0
MSP0101	haib17KIU4866_H7HJMCCXY_SL272779.9	138912	yes	No				prophage/temperate/lysogenic	Yes	5.0	0.0
MSP0102	haib17KIU4866_HMCMJCCXY_SL335938.25	138906	no	Yes			Atlantibacter hermannii	virulent/lytic	Yes	30.0	0.0
MSP0103	haib17CEM5106_HCV72CCXY_SL269799.6	138771	no	Yes	Staphylococcus	Staphylococcus_aureus		prophage/temperate/lysogenic	Yes	33.0	1.0
MSP0104	haib17CEM4890_H2NYMCCXY_SL254818.56	138238	yes	No				virulent/lytic	Yes	11.0	0.0
MSP0105	haib17CEM5106_HCY5JCCXY_SL269600.29	137996	no	Yes				prophage/temperate/lysogenic	Yes	18.0	0.0
MSP0106	haib17CEM4890_H2NYMCCXY_SL254798.1	137625	no	Yes				prophage/temperate/lysogenic	Yes	21.0	0.0
MSP0107	haib17CEM4890_HKC32ALXX_SL254681.55	137404	no	Yes				prophage/temperate/lysogenic	Yes	15.0	0.0
MSP0108	haib17DB4959_H3MGVCCXY_SL259974.12	136599	no	Yes				prophage/temperate/lysogenic	No	25.0	0.0
MSP0109	haib17CEM4890_HKC32ALXX_SL254699.1	136299	yes	No				prophage/temperate/lysogenic	Yes	27.0	0.0
MSP0110	haib17KIU4866_H7HJMCCXY_SL272828.8	135769	no	Yes			Pseudomonas stutzeri A1501	prophage/temperate/lysogenic	Yes	32.0	1.0
MSP0111	haib17DB4959_H3MGVCCXY_SL259966.25	135755	no	Yes			Herbinix luporum	prophage/temperate/lysogenic	Yes	33.0	0.0
MSP0112	haib17CEM5106_HCY5HCCXY_SL270986.38	135611	no	Yes			Bacillus coagulans	prophage/temperate/lysogenic	Yes	30.0	0.0
MSP0113	haib17CEM5106_HCV72CCXY_SL269757.20	135563	no	Yes				prophage/temperate/lysogenic	Yes	19.0	0.0
MSP0114	haib17CEM4890_H2NYMCCXY_SL254784.95	135398	no	No	Bacillus	Bacillus_cereus		virulent/lytic	Yes	4.0	0.0
MSP0115	haib17CEM4890_H75CGCCXY_SL263650.25	134934	no	Yes	Pseudomonas	Pseudomonas_aeruginosa		virulent/lytic	Yes	33.0	1.0
MSP0116	haib17CEM5106_HCY5HCCXY_SL270996.74	134712	no	Yes	Pseudomonas	Pseudomonas_aeruginosa	Pseudomonas aeruginosa	prophage/temperate/lysogenic	No	33.0	1.0
MSP0119	haib17KIU4866_HMCMJCCXY_SL335949.27	134327	no	Yes			Salmonella enterica subsp. enterica	prophage/temperate/lysogenic	No	33.0	1.0
MSP0120	haib17CEM4890_HKC32ALXX_SL254705.85	133614	no	Yes				prophage/temperate/lysogenic	Yes	17.0	0.0
MSP0121	haib17CEM4890_H2NYMCCXY_SL254783.96	133385	yes	No			Clostridium cochlearium	prophage/temperate/lysogenic	Yes	7.0	0.0
MSP0122	haib17CEM5106_HCV72CCXY_SL269805.49	132785	no	Yes				prophage/temperate/lysogenic	No	27.0	0.0
MSP0123	haib17CEM5106_HCVMTCCXY_SL269573.43	132662	no	Yes				prophage/temperate/lysogenic	No	32.0	0.0
MSP0124	haib17CEM4890_H2NYMCCXY_SL254807.44	132091	yes	No				prophage/temperate/lysogenic	Yes	10.0	0.0
MSP0125	haib18CEM5453_HMC2KCCXY_SL336564.1	131891	yes	No				virulent/lytic	Yes	8.0	0.0
MSP0127	haib17CEM4890_H75CGCCXY_SL263638.22	131844	no	No				virulent/lytic	Yes	16.0	0.0
MSP0128	haib17CEM4890_H2NYMCCXY_SL254795.82	131593	no	Yes			Achromobacter denitrificans	virulent/lytic	Yes	32.0	0.0
MSP0129	haib17DB4959_H3MGVCCXY_SL259882.13	131518	yes	No			Shewanella sp. FDAARGOS_354	virulent/lytic	Yes	6.0	0.0
MSP0130	haib17CEM4890_H75CGCCXY_SL263640.49	131481	no	Yes	Pseudomonas	None	Pseudomonas chlororaphis subsp. aurantiaca	prophage/temperate/lysogenic	Yes	27.0	0.0
MSP0131	haib17CEM4890_H75CGCCXY_SL263638.23	131465	no	Yes	Aeromonas	Aeromonas_hydrophila		virulent/lytic	Yes	33.0	0.0
MSP0132	haib17CEM4890_H2NYMCCXY_SL254770.38	131384	no	Yes			Salmonella enterica subsp. enterica serovar Weltevreden	prophage/temperate/lysogenic	Yes	33.0	1.0
MSP0133	haib17CEM4890_H2NYMCCXY_SL254790.8	130998	no	Yes			Psychrobacter sp. YP14	prophage/temperate/lysogenic	Yes	29.0	0.0
MSP0134	haib17CEM4890_H2NYMCCXY_SL254818.62	130714	no	Yes			Clostridioides difficile	prophage/temperate/lysogenic	Yes	13.0	0.0
MSP0135	haib17CEM5106_HCV72CCXY_SL269755.24	130400	no	Yes			Pseudomonas stutzeri A1501	prophage/temperate/lysogenic	Yes	33.0	1.0
MSP0136	haib17CEM4890_H75CGCCXY_SL263678.43	130023	no	Yes			Rahnella aquatilis CIP 78.65 = ATCC 33071	prophage/temperate/lysogenic	Yes	33.0	0.0
MSP0137	haib17CEM5106_HCVMTCCXY_SL269538.60	129813	no	Yes				prophage/temperate/lysogenic	Yes	29.0	0.0
MSP0139	haib17KIU4866_H7HJMCCXY_SL272768.5	128931	no	No				virulent/lytic	Yes	6.0	0.0
MSP0140	haib17CEM5106_HCV72CCXY_SL269786.105	128187	no	Yes				prophage/temperate/lysogenic	No	33.0	0.0
MSP0141	haib17KIU4866_HMCMJCCXY_SL335946.4	127916	no	Yes			Citrobacter koseri	prophage/temperate/lysogenic	Yes	32.0	0.0
MSP0142	haib17CEM4890_HKC32ALXX_SL254757.1	127779	no	No				prophage/temperate/lysogenic	Yes	33.0	0.0
MSP0143	haib17CEM5106_HCY5JCCXY_SL269611.24	127461	no	Yes				prophage/temperate/lysogenic	Yes	11.0	0.0
MSP0144	haib17CEM4890_HMCMJCCXY_SL335843.14	127414	no	No				virulent/lytic	Yes	17.0	0.0
MSP0145	haib17CEM4890_H2NYMCCXY_SL254770.41	127134	no	No				virulent/lytic	Yes	14.0	0.0
MSP0146	haib17CEM5106_HCV72CCXY_SL269718.42	127053	no	Yes	Pseudomonas	None	Xanthomonas campestris pv. raphani 756C	prophage/temperate/lysogenic	Yes	32.0	0.0
MSP0147	haib17CEM4890_H2NYMCCXY_SL254783.101	127014	yes	No				prophage/temperate/lysogenic	Yes	8.0	0.0
MSP0148	haib17CEM4890_H2NYMCCXY_SL254795.92	126300	no	Yes				prophage/temperate/lysogenic	Yes	13.0	0.0
MSP0149	haib17CEM4890_H2NYMCCXY_SL254818.72	125820	no	No				virulent/lytic	Yes	20.0	0.0

Table S1

MSP0150	haib17CEM4890_HKC32ALXX_SL254764.110	125751	no	Yes	Pseudomonas	Pseudomonas_fluorescens	Pseudomonas stutzeri	prophage/temperate/lysogenic	Yes	33.0	0.0
MSP0151	haib17CEM5106_HCY5JCCXY_SL269596.64	125477	no	Yes				prophage/temperate/lysogenic	No	25.0	0.0
MSP0152	haib17CEM4890_H2NYMCCXY_SL254769.80	125361	no	Yes				prophage/temperate/lysogenic	Yes	16.0	0.0
MSP0153	haib17CEM4890_H2NYMCCXY_SL254818.74	125262	no	Yes				prophage/temperate/lysogenic	Yes	12.0	0.0
MSP0154	haib17CEM4890_H75CGCCXY_SL263641.45	124776	no	Yes			Pragia fontium	prophage/temperate/lysogenic	Yes	31.0	0.0
MSP0155	haib17CEM5106_HCY5JCCXY_SL269527.32	124748	no	Yes			Streptococcus thermophilus	prophage/temperate/lysogenic	No	31.0	0.0
MSP0156	haib17CEM4890_H75CGCCXY_SL263687.28	124697	no	Yes			Salmonella enterica subsp. enterica	prophage/temperate/lysogenic	Yes	33.0	1.0
MSP0157	haib17CEM4890_HKC32ALXX_SL254694.29	123937	no	Yes				prophage/temperate/lysogenic	Yes	15.0	0.0
MSP0158	haib17CEM4890_H75CGCCXY_SL263652.51	123732	no	Yes			Kosakonia cowanii JCM 10956 = DSM 18146	prophage/temperate/lysogenic	Yes	33.0	0.0
MSP0159	haib17CEM4890_H2NYMCCXY_SL254858.7	123665	no	Yes			Serratia plymuthica AS9	prophage/temperate/lysogenic	No	32.0	0.0
MSP0160	haib17CEM5106_HCY5HCCXY_SL270906.19	123355	no	Yes			Lactococcus piscium	prophage/temperate/lysogenic	No	31.0	0.0
MSP0161	haib17CEM4890_H75CGCCXY_SL263689.41	123256	no	Yes			Pseudomonas parafulva	prophage/temperate/lysogenic	Yes	33.0	0.0
MSP0162	haib17CEM4890_H75CGCCXY_SL263652.54	122447	no	Yes				prophage/temperate/lysogenic	Yes	31.0	0.0
MSP0163	haib17KIU4866_H7HJMCCXY_SL272828.11	122357	no	Yes			Pseudomonas aeruginosa	prophage/temperate/lysogenic	Yes	33.0	1.0
MSP0164	haib17CEM4890_H2NYMCCXY_SL254831.108	122016	no	Yes				prophage/temperate/lysogenic	Yes	14.0	0.0
MSP0165	haib17CEM4890_H75CGCCXY_SL263712.54	121950	no	Yes			Salmonella enterica subsp. enterica serovar Kentucky	prophage/temperate/lysogenic	No	29.0	1.0
MSP0166	haib17CEM4890_H2NYMCCXY_SL254855.101	121781	no	Yes				prophage/temperate/lysogenic	Yes	15.0	0.0
MSP0167	haib17CEM4890_H75CGCCXY_SL263637.13	121631	no	Yes				prophage/temperate/lysogenic	Yes	17.0	0.0
MSP0168	haib17KIU4866_H7HJMCCXY_SL272776.39	121447	no	Yes			Pantoea vagans	prophage/temperate/lysogenic	No	32.0	0.0
MSP0170	haib17CEM4890_H2NYMCCXY_SL254784.125	121137	yes	No			Clostridioides difficile	prophage/temperate/lysogenic	Yes	5.0	0.0
MSP0169	haib17CEM4890_H2NYMCCXY_SL254788.33	121137	no	Yes			Azotobacter vinelandii DJ	prophage/temperate/lysogenic	Yes	32.0	0.0
MSP0171	haib17CEM5106_HCCGHCCXY_SL270510.132	121034	no	Yes	Salmonella	None	Salmonella bongori N268-08	prophage/temperate/lysogenic	No	33.0	0.0
MSP0172	haib17CEM4890_H75CGCCXY_SL263647.37	121024	yes	Yes			Salmonella enterica subsp. enterica serovar Senftenberg	prophage/temperate/lysogenic	Yes	32.0	1.0
MSP0173	haib17KIU4866_H7HJMCCXY_SL272744.7	120955	no	No				virulent/lytic	Yes	6.0	0.0
MSP0174	haib17CEM4890_H2NYMCCXY_SL254784.126	120876	no	Yes			Kosakonia cowanii JCM 10956 = DSM 18146	prophage/temperate/lysogenic	No	32.0	0.0
MSP0176	haib17CEM4890_H2NYMCCXY_SL254769.84	120548	yes	No				prophage/temperate/lysogenic	Yes	5.0	0.0
MSP0177	haib17CEM4890_H2NYMCCXY_SL254831.114	120463	no	Yes				prophage/temperate/lysogenic	Yes	15.0	0.0
MSP0178	haib17CEM4890_H2NYMCCXY_SL254841.67	120131	no	Yes			Acinetobacter radioresistens	prophage/temperate/lysogenic	Yes	32.0	0.0
MSP0179	haib17CEM5106_HCVMTCCXY_SL269617.43	119640	no	Yes			Lactobacillus paracasei	prophage/temperate/lysogenic	Yes	33.0	0.0
MSP0180	haib17CEM4890_H2NYMCCXY_SL254817.76	119531	no	Yes				prophage/temperate/lysogenic	Yes	14.0	0.0
MSP0181	haib17KIU4866_H7HJMCCXY_SL272801.1	119202	no	No	Erwinia	Erwinia_amylovora		virulent/lytic	Yes	11.0	0.0
MSP0182	haib17CEM4890_H2NYMCCXY_SL254852.43	118815	no	Yes				prophage/temperate/lysogenic	Yes	9.0	0.0
MSP0183	haib17CEM4890_H2NYMCCXY_SL254815.8	118206	no	Yes				prophage/temperate/lysogenic	Yes	33.0	0.0
MSP0184	haib17CEM4890_H2NYMCCXY_SL254772.116	117835	no	Yes				prophage/temperate/lysogenic	Yes	12.0	0.0
MSP0185	haib17CEM4890_HKC32ALXX_SL254705.97	117679	yes	Yes				prophage/temperate/lysogenic	Yes	7.0	0.0
MSP0186	haib17DB4959_H3MGVCCXY_SL259989.9	117558	yes	Yes	Staphylococcus	Staphylococcus_epidermidis		prophage/temperate/lysogenic	Yes	33.0	1.0
MSP0187	haib17CEM4890_H2NYMCCXY_SL254771.85	117534	no	Yes			Salmonella enterica subsp. enterica serovar Sanjuan	prophage/temperate/lysogenic	No	33.0	1.0
MSP0188	haib17CEM4890_H2NYMCCXY_SL254854.60	117383	no	Yes			Serratia plymuthica AS9	prophage/temperate/lysogenic	Yes	33.0	0.0
MSP0189	haib17CEM4890_H2NYMCCXY_SL254782.100	116734	no	No			Clostridioides difficile	prophage/temperate/lysogenic	Yes	9.0	0.0
MSP0190	haib17CEM4890_H2NYMCCXY_SL254806.85	116364	no	No				virulent/lytic	Yes	23.0	0.0
MSP0192	haib17CEM4890_H2NYMCCXY_SL254832.6	115970	no	Yes	Pseudomonas	Pseudomonas_aeruginosa	Pseudomonas aeruginosa	prophage/temperate/lysogenic	No	33.0	1.0
MSP0193	haib17CEM4890_HKC32ALXX_SL254699.3	114688	yes	Yes	Arthrobacter	Arthrobacter_globiformis		virulent/lytic	Yes	13.0	0.0
MSP0195	haib17CEM4890_H2NYMCCXY_SL254784.136	114443	yes	No				prophage/temperate/lysogenic	Yes	10.0	0.0
MSP0196	haib17CEM5106_HCY5HCCXY_SL270934.46	114386	no	Yes	Lactobacillus	None		prophage/temperate/lysogenic	Yes	26.0	0.0
MSP0197	haib17CEM5106_HCV72CCXY_SL269738.27	114373	no	Yes				prophage/temperate/lysogenic	Yes	32.0	0.0
MSP0198	haib17CEM5106_HCVMTCCXY_SL269598.40	114173	no	Yes				prophage/temperate/lysogenic	Yes	28.0	0.0
MSP0199	haib17CEM4890_HKC32ALXX_SL254716.25	113914	no	No				prophage/temperate/lysogenic	Yes	10.0	0.0
MSP0200	haib17KIU4866_H7HJMCCXY_SL272788.30	113713	no	Yes			Methylomicrobium album BG8	prophage/temperate/lysogenic	No	33.0	0.0
MSP0201	haib17CEM4890_HKC32ALXX_SL254680.87	113469	no	Yes			Salmonella enterica subsp. enterica	prophage/temperate/lysogenic	No	33.0	1.0
MSP0202	haib17CEM5106_HCV72CCXY_SL269748.102	113314	no	Yes			Pseudomonas sp. CMR12a	prophage/temperate/lysogenic	Yes	33.0	0.0
MSP0203	haib17CEM4890_H2NYMCCXY_SL254839.17	113215	no	Yes			Pseudomonas citronellolis	prophage/temperate/lysogenic	Yes	33.0	0.0
MSP0204	haib17CEM4890_H2NYMCCXY_SL254830.108	113038	no	Yes				prophage/temperate/lysogenic	No	19.0	0.0
MSP0205	haib17CEM5106_HCY5HCCXY_SL270942.24	112968	no	Yes			Pseudomonas chlororaphis subsp. aurantiaca	prophage/temperate/lysogenic	Yes	33.0	0.0
MSP0206	haib17DB4959_H3MGVCCXY_SL259966.35	112610	no	Yes	Staphylococcus	Staphylococcus_epidermidis	Staphylococcus schleiferi	prophage/temperate/lysogenic	No	31.0	0.0
MSP0207	haib17KIU4866_H7HJMCCXY_SL272759.5	112344	no	Yes				prophage/temperate/lysogenic	No	16.0	0.0
MSP0208	haib17CEM4890_H2NYMCCXY_SL254836.28	111872	no	Yes				prophage/temperate/lysogenic	Yes	15.0	0.0
MSP0209	haib17CEM4890_H2NYMCCXY_SL254772.125	111737	no	Yes				prophage/temperate/lysogenic	Yes	12.0	0.0
MSP0210	haib17KIU4866_H7HJMCCXY_SL272827.21	111628	no	Yes				prophage/temperate/lysogenic	Yes	30.0	0.0
MSP0211	haib18CEM5453_HNHKFCCXY_SL345930.1	111560	no	Yes				virulent/lytic	Yes	27.0	0.0
MSP0212	haib18CEM5453_HMC2KCCXY_SL336804.1	111523	no	No				virulent/lytic	Yes	32.0	0.0
MSP0213	haib17CEM5106_HCV72CCXY_SL269749.73	111409	no	Yes			Pseudomonas parafulva	prophage/temperate/lysogenic	Yes	29.0	0.0
MSP0214	haib17KIU4866_H7HJMCCXY_SL272759.6	111304	no	No				prophage/temperate/lysogenic	Yes	33.0	0.0
MSP0215	haib17KIU4866_HMCMJCCXY_SL335948.19	111078	no	Yes			Neisseria subflava	prophage/temperate/lysogenic	No	32.0	0.0
MSP0216	haib17KIU4866_H7HJMCCXY_SL272752.13	111053	no	No				virulent/lytic	Yes	21.0	0.0
MSP0217	haib17CEM5106_HCY5JCCXY_SL269588.30	110983	no	Yes				prophage/temperate/lysogenic	Yes	27.0	0.0
MSP0218	haib17KIU4866_H7HJMCCXY_SL272796.65	110740	no	Yes			Klebsiella oxytoca	prophage/temperate/lysogenic	No	33.0	0.0
MSP0219	haib17CEM5106_HCV72CCXY_SL269751.33	110618	no	Yes	Pseudomonas	Pseudomonas_aeruginosa	Pseudomonas stutzeri A1501	prophage/temperate/lysogenic	Yes	33.0	0.0
MSP0220	haib17CEM5106_HCV72CCXY_SL269751.34	110486	yes	No			Salmonella enterica subsp. diarizonae	prophage/temperate/lysogenic	No	23.0	1.0

MSP0221	haib17CEM5106_HCV72CCXY_SL269758.40	110343	no	Yes			Psychrobacter sp. P11G5	prophage/temperate/lysogenic	Yes	29.0	0.0
MSP0222	haib17CEM4890_HKC32ALXX_SL254728.134	110200	no	Yes				prophage/temperate/lysogenic	Yes	11.0	0.0
MSP0223	haib17CEM4890_H2NYMCCXY_SL254830.112	110188	no	Yes				prophage/temperate/lysogenic	Yes	15.0	0.0
MSP0224	haib17CEM4890_HKC32ALXX_SL254711.17	110088	no	Yes	Lactobacillus	Lactobacillus_casei	Lactobacillus sp. CBA3606	prophage/temperate/lysogenic	No	23.0	0.0
MSP0225	haib17CEM5106_HCY5HCCXY_SL270959.75	110083	no	No			Bacillus safensis FO-36b	prophage/temperate/lysogenic	Yes	18.0	0.0
MSP0226	haib17CEM4890_H75CGCCXY_SL263638.25	109959	no	No	Erwinia	Erwinia_amylovora		virulent/lytic	Yes	14.0	0.0
MSP0227	haib17CEM4890_H75CGCCXY_SL263652.67	109704	no	Yes			Pectobacterium brasiliense	prophage/temperate/lysogenic	Yes	33.0	0.0
MSP0228	haib17CEM4890_HKC32ALXX_SL254680.91	109495	no	Yes			Hungateiclostridium saccincola	prophage/temperate/lysogenic	Yes	17.0	0.0
MSP0229	haib17CEM4890_H75CGCCXY_SL263647.42	109127	no	Yes			Pseudomonas aeruginosa	prophage/temperate/lysogenic	Yes	32.0	1.0
MSP0230	haib17CEM4890_HKC32ALXX_SL254742.102	108710	no	No				prophage/temperate/lysogenic	Yes	13.0	0.0
MSP0232	haib17CEM4890_H2NYMCCXY_SL254794.214	107755	no	Yes				prophage/temperate/lysogenic	Yes	14.0	0.0
MSP0233	haib17CEM4890_H75CGCCXY_SL263712.63	107422	no	Yes			Kosakonia cowanii JCM 10956 = DSM 18146	prophage/temperate/lysogenic	Yes	33.0	0.0
MSP0234	haib17CEM5106_HCVMTCCXY_SL269605.24	107298	no	Yes				prophage/temperate/lysogenic	Yes	31.0	0.0
MSP0235	haib17CEM4890_H75CGCCXY_SL263651.38	106991	no	Yes			Klebsiella pneumoniae	prophage/temperate/lysogenic	Yes	27.0	1.0
MSP0236	haib17DB4959_H3MGVCCXY_SL259923.18	106819	no	Yes	Staphylococcus	Staphylococcus_epidermidis		prophage/temperate/lysogenic	No	31.0	1.0
MSP0237	haib17CEM4890_H2NYMCCXY_SL254846.16	106548	no	Yes			Salmonella enterica subsp. enterica serovar Waycross	prophage/temperate/lysogenic	No	29.0	1.0
MSP0238	haib17CEM5106_HCY5JCCXY_SL269596.75	105700	no	Yes				virulent/lytic	Yes	17.0	0.0
MSP0239	haib17KIU4866_H7HJMCCXY_SL272744.12	105263	no	No	Erwinia	None		virulent/lytic	Yes	17.0	0.0
MSP0240	haib17CEM5106_HCVMTCCXY_SL269610.51	105094	no	Yes	Gordonia	Gordonia_terrae		prophage/temperate/lysogenic	Yes	30.0	0.0
MSP0241	haib17CEM5106_HCV72CCXY_SL269719.44	104857	no	Yes	Pseudomonas	Pseudomonas_aeruginosa	Pseudomonas stutzeri	prophage/temperate/lysogenic	Yes	33.0	0.0
MSP0242	haib17CEM4890_H2NYMCCXY_SL254769.98	104646	no	Yes				prophage/temperate/lysogenic	Yes	15.0	0.0
MSP0243	haib17CEM4890_HKC32ALXX_SL254698.1	104563	no	No			Neisseria subflava	virulent/lytic	Yes	18.0	0.0
MSP0244	haib17CEM5106_HCCGHCCXY_SL270294.1	104454	yes	No				prophage/temperate/lysogenic	Yes	28.0	0.0
MSP0245	haib17DB4959_H3MGVCCXY_SL259989.13	104389	no	Yes	Staphylococcus	Staphylococcus_aureus	Staphylococcus delphini	prophage/temperate/lysogenic	No	28.0	0.0
MSP0246	haib17CEM4890_H75CGCCXY_SL263689.46	104044	no	Yes			Escherichia coli HS	prophage/temperate/lysogenic	Yes	31.0	1.0
MSP0247	haib17CEM4890_HKC32ALXX_SL254718.124	103873	no	Yes				prophage/temperate/lysogenic	Yes	17.0	0.0
MSP0248	haib17KIU4866_H7HJMCCXY_SL272743.16	103804	no	Yes			Serratia plymuthica AS9	prophage/temperate/lysogenic	No	31.0	0.0
MSP0249	haib17CEM4890_H2NYMCCXY_SL254795.138	103721	no	Yes				prophage/temperate/lysogenic	No	33.0	0.0
MSP0250	haib17CEM5106_HCY5JCCXY_SL269570.23	103641	no	Yes				prophage/temperate/lysogenic	Yes	33.0	0.0
MSP0252	haib17CEM5106_HCVMTCCXY_SL269601.49	102813	no	Yes			Pseudomonas chlororaphis	prophage/temperate/lysogenic	No	32.0	0.0
MSP0253	haib17KIU4866_H7HJMCCXY_SL272827.26	102662	no	Yes			Serratia plymuthica AS9	prophage/temperate/lysogenic	No	32.0	0.0
MSP0254	haib17KIU4866_H7HJMCCXY_SL272774.7	102403	no	No	Erwinia	Erwinia_amylovora		virulent/lytic	Yes	9.0	0.0
MSP0255	haib17CEM5106_HCV72CCXY_SL269759.11	102110	no	Yes			Dickeya zeae	prophage/temperate/lysogenic	Yes	28.0	0.0
MSP0256	haib17CEM5106_HCVMTCCXY_SL269535.68	102045	no	Yes	Gordonia	Gordonia_terrae		prophage/temperate/lysogenic	Yes	28.0	0.0
MSP0257	haib17CEM4890_HKC32ALXX_SL254681.106	101905	no	Yes			Clostridium botulinum	prophage/temperate/lysogenic	Yes	15.0	0.0
MSP0258	haib17KIU4866_H7HJMCCXY_SL272774.8	101602	no	Yes				prophage/temperate/lysogenic	Yes	32.0	0.0
MSP0259	haib17CEM5106_HCV72CCXY_SL269793.56	101545	no	Yes				prophage/temperate/lysogenic	Yes	24.0	0.0
MSP0260	haib17CEM4890_H75CGCCXY_SL263640.67	101504	no	Yes				prophage/temperate/lysogenic	Yes	33.0	0.0
MSP0261	haib17CEM4890_H2NYMCCXY_SL254770.68	101217	no	Yes	Clostridium	Clostridium_perfringens		prophage/temperate/lysogenic	Yes	12.0	0.0
MSP0263	haib17CEM4890_H2NYMCCXY_SL254820.2	101164	no	Yes			Enterobacter cloacae	prophage/temperate/lysogenic	No	32.0	0.0
MSP0262	haib17KIU4866_H7HJMCCXY_SL272751.20	101164	yes	No				virulent/lytic	Yes	5.0	0.0
MSP0264	haib17CEM4890_H75CGCCXY_SL263714.34	100973	no	Yes				prophage/temperate/lysogenic	Yes	30.0	0.0
MSP0265	haib17CEM4890_HMCMJCCXY_SL335833.79	100947	no	Yes	Staphylococcus	Staphylococcus_aureus	Staphylococcus epidermidis	prophage/temperate/lysogenic	No	32.0	0.0
MSP0266	haib17CEM4890_H7KYMCCXY_SL273087.2	100635	no	No				virulent/lytic	Yes	17.0	0.0
MSP0267	haib17CEM4890_H2NYMCCXY_SL254850.25	100605	no	No	Acinetobacter	Acinetobacter_baumannii		virulent/lytic	Yes	17.0	1.0
MSP0268	haib17CEM5106_HCV72CCXY_SL269738.33	100604	no	Yes	Gordonia	Gordonia_terrae		prophage/temperate/lysogenic	Yes	30.0	0.0
MSP0269	haib17CEM4890_H75CGCCXY_SL263677.15	100452	no	Yes			Acinetobacter baumannii	virulent/lytic	No	31.0	1.0
MSP0270	haib17CEM4890_H2NYMCCXY_SL254794.237	99942	no	Yes			Acidovorax avenae subsp. avenae ATCC 19860	prophage/temperate/lysogenic	Yes	33.0	0.0
MSP0271	haib17CEM4890_H2NYMCCXY_SL254805.135	99731	no	Yes				virulent/lytic	Yes	32.0	0.0
MSP0272	haib17CEM5106_HCY5HCCXY_SL270997.32	99625	no	Yes				virulent/lytic	Yes	25.0	0.0
MSP0273	haib17CEM5106_HCY5JCCXY_SL269557.39	99596	no	Yes	Arthrobacter	Arthrobacter_globiformis		prophage/temperate/lysogenic	Yes	32.0	0.0
MSP0274	haib17KIU4866_H7HJMCCXY_SL272757.25	99418	no	Yes	Clostridium	Clostridium_perfringens		prophage/temperate/lysogenic	Yes	14.0	0.0
MSP0275	haib17CEM4890_H2NYMCCXY_SL254841.85	99346	no	Yes				prophage/temperate/lysogenic	Yes	11.0	0.0
MSP0276	haib17CEM5106_HCV72CCXY_SL269782.23	99280	no	Yes	Arthrobacter	Arthrobacter_globiformis		prophage/temperate/lysogenic	Yes	33.0	0.0
MSP0277	haib17CEM4890_HKC32ALXX_SL254685.1	98848	yes	No	Acinetobacter	Acinetobacter_baumannii		virulent/lytic	Yes	14.0	1.0
MSP0278	haib17KIU4866_H7HJMCCXY_SL272773.82	98817	no	Yes			Pseudomonas aeruginosa 19BR	prophage/temperate/lysogenic	Yes	32.0	1.0
MSP0279	haib17CEM5106_HCY5HCCXY_SL270981.43	98805	no	Yes				prophage/temperate/lysogenic	Yes	24.0	0.0
MSP0280	haib17KIU4866_H7HJMCCXY_SL272777.31	98665	no	Yes			Acinetobacter sp. LoGeW2-3	prophage/temperate/lysogenic	Yes	30.0	0.0
MSP0281	haib17KIU4866_H7HJMCCXY_SL272778.35	98612	no	Yes				prophage/temperate/lysogenic	Yes	33.0	0.0
MSP0282	haib17CEM4890_H2NYMCCXY_SL254818.105	98516	no	Yes			Klebsiella pneumoniae subsp. pneumoniae	prophage/temperate/lysogenic	Yes	17.0	1.0
MSP0283	haib17CEM5106_HCVMTCCXY_SL269532.61	98174	no	Yes			Pseudomonas chlororaphis subsp. aurantiaca	virulent/lytic	Yes	32.0	0.0
MSP0284	haib17CEM4890_H2NYMCCXY_SL254829.120	97920	no	Yes			Kosakonia cowanii JCM 10956 = DSM 18146	prophage/temperate/lysogenic	No	28.0	0.0
MSP0285	haib17CEM4890_H2NYMCCXY_SL254854.85	97564	no	Yes			Xenorhabdus doucetiae	prophage/temperate/lysogenic	Yes	29.0	0.0
MSP0286	haib17CEM4890_HKC32ALXX_SL254675.1	97536	no	No				virulent/lytic	Yes	21.0	0.0
MSP0287	haib17DB4959_H3MGVCCXY_SL259985.11	97503	no	No				virulent/lytic	Yes	20.0	0.0
MSP0288	haib17KIU4866_HMCMJCCXY_SL335963.19	97492	no	Yes	Acinetobacter	Acinetobacter_baumannii	Acinetobacter baumannii	prophage/temperate/lysogenic	No	32.0	1.0
MSP0289	haib17CEM4890_H75CGCCXY_SL263654.27	97400	no	Yes	Cronobacter	Cronobacter_sakazakii	Dickeya sp. CSL RW240	prophage/temperate/lysogenic	Yes	31.0	0.0
MSP0290	haib17CEM4890_H2NYMCCXY_SL254854.86	97395	no	Yes			Citrobacter koseri	prophage/temperate/lysogenic	Yes	33.0	0.0

MSP0291	haib17CEM4890_H2NYMCCXY_SL254806.118	97232	no	Yes				prophage/temperate/lysogenic	Yes	15.0	0.0
MSP0293	haib17CEM4890_H2NYMCCXY_SL254843.171	97193	no	Yes	Pseudomonas	Pseudomonas_aeruginosa	Pseudomonas aeruginosa	prophage/temperate/lysogenic	Yes	33.0	1.0
MSP0292	haib17CEM4890_H7KYMCCXY_SL273127.9	97193	no	Yes	Staphylococcus	None	Bacillus halodurans C-125	prophage/temperate/lysogenic	No	33.0	0.0
MSP0294	haib17CEM4890_H2NYMCCXY_SL254795.154	97182	no	Yes				prophage/temperate/lysogenic	Yes	14.0	0.0
MSP0295	haib18CEM5453_HNHKFCCXY_SL345538.27	97154	no	No				prophage/temperate/lysogenic	Yes	33.0	0.0
MSP0296	haib17CEM4890_H2NYMCCXY_SL254770.77	96982	no	No				prophage/temperate/lysogenic	Yes	13.0	0.0
MSP0297	haib17CEM4890_H75CGCCXY_SL263647.49	96944	no	Yes				prophage/temperate/lysogenic	Yes	33.0	0.0
MSP0298	haib17CEM4890_H2NYMCCXY_SL254793.47	96484	no	Yes	Klebsiella	Klebsiella_pneumoniae	Klebsiella pneumoniae subsp. pneumoniae	prophage/temperate/lysogenic	No	33.0	1.0
MSP0299	haib17CEM4890_H2NYMCCXY_SL254781.232	96347	no	Yes	Clostridium	Clostridium_perfringens		prophage/temperate/lysogenic	No	13.0	0.0
MSP0300	haib17KIU4866_H7HJMCCXY_SL272796.80	96323	no	Yes			Klebsiella pneumoniae	prophage/temperate/lysogenic	Yes	26.0	1.0
MSP0301	haib17CEM5106_HCV72CCXY_SL269759.13	96175	no	Yes				prophage/temperate/lysogenic	Yes	19.0	0.0
MSP0302	haib17CEM4890_H75CGCCXY_SL263652.82	96131	no	Yes			Kosakonia cowanii JCM 10956 = DSM 18146	prophage/temperate/lysogenic	Yes	29.0	0.0
MSP0303	haib17CEM5106_HCCGHCCXY_SL270433.3	96123	yes	No				prophage/temperate/lysogenic	Yes	20.0	0.0
MSP0304	haib17CEM4890_H75CGCCXY_SL263639.59	95974	yes	No			Salmonella enterica subsp. enterica serovar Bergen str. ST350	prophage/temperate/lysogenic	Yes	27.0	1.0
MSP0305	haib17KIU4866_H7HJMCCXY_SL272816.55	95323	no	Yes	Acinetobacter	Acinetobacter_baumannii	Acinetobacter baumannii	prophage/temperate/lysogenic	Yes	33.0	1.0
MSP0306	haib17CEM5106_HCY5JCCXY_SL269608.31	95262	no	Yes	Staphylococcus	Staphylococcus_aureus		prophage/temperate/lysogenic	Yes	33.0	1.0
MSP0307	haib17CEM5106_HCY5JCCXY_SL269588.38	94886	no	Yes				prophage/temperate/lysogenic	Yes	25.0	0.0
MSP0308	haib17CEM5106_HCY5HCCXY_SL270953.45	94739	no	Yes	Propionibacterium	None	Acidipropionibacterium virtanenii	prophage/temperate/lysogenic	Yes	33.0	0.0
MSP0309	haib17CEM4890_HKC32ALXX_SL254676.26	94718	no	No				prophage/temperate/lysogenic	Yes	31.0	0.0
MSP0310	haib17CEM4890_H75CGCCXY_SL263713.43	94318	no	Yes			Cronobacter muytjensii ATCC 51329	prophage/temperate/lysogenic	Yes	29.0	0.0
MSP0311	haib17CEM4890_H75CGCCXY_SL263685.21	94117	no	Yes				prophage/temperate/lysogenic	Yes	32.0	0.0
MSP0312	haib17CEM5106_HCVMTCCXY_SL269545.76	94104	no	Yes			Actinoalloteichus sp. GBA129-24	prophage/temperate/lysogenic	Yes	32.0	0.0
MSP0313	haib17CEM5106_HCV72CCXY_SL269748.142	93761	no	Yes				prophage/temperate/lysogenic	Yes	30.0	0.0
MSP0314	haib17CEM4890_HMCMJCCXY_SL335783.22	93235	no	Yes			Shimwellia blattae DSM 4481 = NBRC 105725	prophage/temperate/lysogenic	Yes	25.0	0.0
MSP0315	haib17CEM4890_H75CGCCXY_SL263654.34	93202	no	Yes			Pseudomonas balearica DSM 6083	prophage/temperate/lysogenic	No	33.0	1.0
MSP0316	haib17KIU4866_H7HJMCCXY_SL272816.60	93173	no	Yes				prophage/temperate/lysogenic	Yes	32.0	0.0
MSP0317	haib18CEM5453_HMGN5CCXY_SL344653.1	93109	no	No				prophage/temperate/lysogenic	Yes	4.0	0.0
MSP0318	haib17CEM4890_H2NYMCCXY_SL254830.146	93055	no	No				prophage/temperate/lysogenic	Yes	10.0	0.0
MSP0319	haib17CEM4890_H2NYMCCXY_SL254818.119	92930	no	Yes				prophage/temperate/lysogenic	Yes	12.0	0.0
MSP0320	haib18CEM5453_HMGW3CCXY_SL342350.1	92885	no	No				virulent/lytic	Yes	16.0	0.0
MSP0321	haib17CEM4890_H2NYMCCXY_SL254781.240	92840	no	Yes			Fusobacterium varium	prophage/temperate/lysogenic	Yes	15.0	0.0
MSP0322	haib17CEM5106_HCY5HCCXY_SL270956.1	92650	no	Yes				prophage/temperate/lysogenic	Yes	30.0	0.0
MSP0323	haib17CEM4890_HMCMJCCXY_SL335820.20	92556	no	Yes				virulent/lytic	Yes	29.0	0.0
MSP0325	haib17CEM5106_HCY5HCCXY_SL270983.71	92403	no	Yes				prophage/temperate/lysogenic	Yes	26.0	0.0
MSP0326	haib17CEM4890_HMCMJCCXY_SL335833.89	92387	no	Yes			Acinetobacter sp. WCHA45	prophage/temperate/lysogenic	Yes	31.0	0.0
MSP0327	haib17KIU4866_HMCMJCCXY_SL335946.14	92191	no	Yes			Pasteurella multocida subsp. gallicida	prophage/temperate/lysogenic	Yes	32.0	0.0
MSP0328	haib17CEM4890_H75CGCCXY_SL263687.41	92184	no	Yes			Escherichia coli 042	prophage/temperate/lysogenic	No	31.0	1.0
MSP0329	haib17CEM4890_H2NYMCCXY_SL254829.128	92128	no	Yes				prophage/temperate/lysogenic	Yes	13.0	0.0
MSP0330	haib17DB4959_H3MGVCCXY_SL259974.23	91940	no	Yes			Enterococcus faecalis 62	prophage/temperate/lysogenic	No	26.0	1.0
MSP0331	haib17CEM4890_H2NYMCCXY_SL254770.81	91930	no	Yes				prophage/temperate/lysogenic	Yes	13.0	0.0
MSP0332	haib17KIU4866_HMCMJCCXY_SL335956.64	91907	no	Yes				prophage/temperate/lysogenic	Yes	13.0	0.0
MSP0333	haib17CEM4890_HKC32ALXX_SL254700.22	91833	no	Yes				prophage/temperate/lysogenic	Yes	21.0	0.0
MSP0334	haib17KIU4866_HMCMJCCXY_SL335956.65	91771	no	Yes			Metakosakonia sp. MRY16-398	prophage/temperate/lysogenic	Yes	32.0	0.0
MSP0335	haib17CEM4890_H75CGCCXY_SL263649.60	91692	no	Yes				virulent/lytic	Yes	21.0	0.0
MSP0336	haib17CEM4890_H2NYMCCXY_SL254806.129	91674	no	No			Salmonella enterica subsp. diarizonae	prophage/temperate/lysogenic	No	33.0	1.0
MSP0337	haib17CEM4890_H2NYMCCXY_SL254780.155	91634	no	Yes				prophage/temperate/lysogenic	Yes	33.0	0.0
MSP0338	haib17CEM4890_H2NYMCCXY_SL254846.22	91116	no	Yes			Salmonella enterica subsp. enterica serovar Sanjuan	prophage/temperate/lysogenic	No	32.0	1.0
MSP0340	haib17CEM4890_HKC32ALXX_SL254721.1	90901	no	No				virulent/lytic	Yes	20.0	0.0
MSP0341	haib17CEM4890_HKC32ALXX_SL254680.110	90860	no	Yes			Bacillus halodurans C-125	prophage/temperate/lysogenic	Yes	31.0	0.0
MSP0342	haib17CEM5106_HCV72CCXY_SL269765.88	90825	no	Yes				prophage/temperate/lysogenic	Yes	33.0	0.0
MSP0343	haib17CEM4890_H2NYMCCXY_SL254830.159	90745	no	Yes			Klebsiella michiganensis	prophage/temperate/lysogenic	No	33.0	0.0
MSP0344	haib17KIU4866_H7HJMCCXY_SL272814.21	90704	yes	No			Klebsiella pneumoniae subsp. pneumoniae	prophage/temperate/lysogenic	Yes	15.0	1.0
MSP0345	haib17CEM5106_HCVMTCCXY_SL269532.71	90675	no	Yes			Pseudomonas chlororaphis	prophage/temperate/lysogenic	Yes	30.0	0.0
MSP0346	haib17CEM4890_H75CGCCXY_SL263714.43	90404	no	Yes			Escherichia coli HS	prophage/temperate/lysogenic	No	32.0	1.0
MSP0347	haib17CEM4890_H2NYMCCXY_SL254790.30	90180	no	No				prophage/temperate/lysogenic	Yes	21.0	0.0
MSP0348	haib18CEM5453_HMC2KCCXY_SL336593.1	90010	no	No			Pseudomonas aeruginosa	prophage/temperate/lysogenic	Yes	14.0	1.0
MSP0349	haib17CEM5106_HCV72CCXY_SL269742.68	89982	no	No			Thermosipho sp. 1070	prophage/temperate/lysogenic	Yes	13.0	0.0
MSP0350	haib17CEM4890_HMCMJCCXY_SL335787.17	89764	yes	No				prophage/temperate/lysogenic	Yes	8.0	0.0
MSP0352	haib17CEM4890_H2NYMCCXY_SL254783.180	89750	no	No			Clostridium butyricum	virulent/lytic	Yes	7.0	0.0
MSP0353	haib17CEM5106_HCY5HCCXY_SL270942.40	89635	no	Yes			Bordetella sp. J329	prophage/temperate/lysogenic	Yes	32.0	0.0
MSP0354	haib17CEM5106_HCY5JCCXY_SL269609.58	89626	no	Yes			Enterococcus faecalis 62	prophage/temperate/lysogenic	No	29.0	1.0
MSP0355	haib17CEM4890_H2NYMCCXY_SL254856.32	89511	no	No	Vibrio	Vibrio_alginolyticus		virulent/lytic	Yes	21.0	0.0
MSP0356	haib17KIU4866_H7HJMCCXY_SL272796.88	89332	no	Yes			Dickeya chrysanthemi Ech1591	prophage/temperate/lysogenic	No	33.0	0.0
MSP0357	haib17CEM4890_H75CGCCXY_SL263663.29	89324	no	Yes			Pseudomonas sp. MRSN12121	prophage/temperate/lysogenic	Yes	32.0	0.0
MSP0358	haib17CEM4890_H2NYMCCXY_SL254809.2	89281	no	No				virulent/lytic	Yes	26.0	0.0
MSP0359	haib17KIU4866_H7HJMCCXY_SL272793.27	89103	no	No				virulent/lytic	Yes	16.0	0.0
MSP0360	haib17CEM5106_HCVMTCCXY_SL269612.42	89070	no	Yes				prophage/temperate/lysogenic	Yes	32.0	0.0
MSP0361	haib17CEM5106_HCY5HCCXY_SL270911.58	89043	no	Yes			Xanthomonas albilineans GPE PC73	prophage/temperate/lysogenic	Yes	33.0	0.0

MSP0362	haib17CEM4890_H2NYMCCXY_SL254781.255	88814	no	Yes				prophage/temperate/lysogenic	Yes	14.0	0.0
MSP0363	haib17CEM4890_HKC32ALXX_SL254766.94	88803	no	Yes			Pectobacterium brasiliense	prophage/temperate/lysogenic	No	31.0	0.0
MSP0364	haib17CEM5106_HCV72CCXY_SL269771.64	88709	no	Yes			Pseudomonas fluorescens	prophage/temperate/lysogenic	Yes	33.0	1.0
MSP0365	haib17CEM4890_H2NYMCCXY_SL254818.127	88705	no	Yes				prophage/temperate/lysogenic	Yes	29.0	0.0
MSP0366	haib17CEM4890_HKC32ALXX_SL254751.6	88591	no	No				virulent/lytic	Yes	11.0	0.0
MSP0367	haib17CEM5106_HCV72CCXY_SL269731.68	88510	no	Yes				prophage/temperate/lysogenic	Yes	32.0	0.0
MSP0368	haib17KIU4866_H7HJMCCXY_SL272784.26	88492	no	Yes	Pseudomonas	Pseudomonas_aeruginosa	Pseudomonas stutzeri	prophage/temperate/lysogenic	Yes	33.0	0.0
MSP0369	haib17CEM4890_H75CGCCXY_SL263663.30	88473	no	Yes			Pseudomonas chlororaphis	prophage/temperate/lysogenic	Yes	33.0	0.0
MSP0370	haib17CEM4890_H7KYMCCXY_SL273087.3	88457	no	Yes				virulent/lytic	Yes	32.0	0.0
MSP0371	haib17CEM4890_H2NYMCCXY_SL254769.124	88432	no	Yes				prophage/temperate/lysogenic	Yes	15.0	0.0
MSP0372	haib17CEM4890_HKC32ALXX_SL254752.88	88427	no	Yes			Mixta calida	prophage/temperate/lysogenic	Yes	28.0	0.0
MSP0373	haib17DB4959_H3MGVCCXY_SL259963.82	87995	no	Yes	Pseudomonas	Pseudomonas_aeruginosa		virulent/lytic	Yes	33.0	1.0
MSP0374	haib17CEM4890_H2NYMCCXY_SL254861.16	87975	yes	No				prophage/temperate/lysogenic	No	21.0	0.0
MSP0375	haib17CEM5106_HCY5JCCXY_SL269609.60	87919	no	Yes			Xanthomonas campestris pv. raphani 756C	prophage/temperate/lysogenic	Yes	32.0	1.0
MSP0376	haib18CEM5453_HMC2KCCXY_SL336681.7	87914	no	No				virulent/lytic	No	30.0	0.0
MSP0377	haib17CEM4890_HKC32ALXX_SL254681.147	87717	no	No				prophage/temperate/lysogenic	Yes	16.0	0.0
MSP0378	haib17KIU4866_HMCMJCCXY_SL335957.23	87665	no	Yes			Acinetobacter sp. ACNIH1	prophage/temperate/lysogenic	Yes	32.0	0.0
MSP0379	haib17KIU4866_H7HJMCCXY_SL272801.2	87638	yes	No	Escherichia	Escherichia_coli	Klebsiella pneumoniae subsp. pneumoniae	prophage/temperate/lysogenic	Yes	23.0	0.0
MSP0380	haib17CEM4890_H2NYMCCXY_SL254856.35	87401	no	Yes			Serratia plymuthica PRI-2C	prophage/temperate/lysogenic	Yes	29.0	0.0
MSP0381	haib17KIU4866_H7HJMCCXY_SL272798.47	87395	yes	No				prophage/temperate/lysogenic	Yes	18.0	0.0
MSP0382	haib17KIU4866_H7HJMCCXY_SL272813.6	87252	no	No				prophage/temperate/lysogenic	Yes	29.0	0.0
MSP0383	haib17CEM4890_H2NYMCCXY_SL254843.206	87081	no	Yes				prophage/temperate/lysogenic	No	20.0	0.0
MSP0384	haib17KIU4866_H7HJMCCXY_SL272792.4	87039	no	No	Erwinia	None		virulent/lytic	Yes	15.0	0.0
MSP0385	haib17CEM4890_H2NYMCCXY_SL254783.187	87006	no	Yes			Klebsiella pneumoniae	prophage/temperate/lysogenic	No	28.0	1.0
MSP0386	haib17CEM5106_HCV72CCXY_SL269743.127	86889	no	Yes				prophage/temperate/lysogenic	Yes	32.0	0.0
MSP0387	haib17KIU4866_H7HJMCCXY_SL272772.15	86639	yes	No				virulent/lytic	Yes	14.0	0.0
MSP0388	haib17CEM5106_HCV72CCXY_SL269728.73	86613	no	Yes			Haliangium ochraceum DSM 14365	prophage/temperate/lysogenic	Yes	33.0	0.0
MSP0389	haib17CEM4890_H3KHWCCXY_SL263315.1	86528	yes	No				virulent/lytic	Yes	17.0	0.0
MSP0391	haib17KIU4866_H7HJMCCXY_SL272806.13	86403	no	No			Salmonella enterica subsp. enterica serovar Kentucky	prophage/temperate/lysogenic	Yes	21.0	1.0
MSP0392	haib17CEM4890_HKC32ALXX_SL254706.136	86359	no	No				prophage/temperate/lysogenic	Yes	14.0	0.0
MSP0393	haib17CEM5106_HCY5JCCXY_SL269609.64	86179	yes	No				virulent/lytic	Yes	10.0	0.0
MSP0396	haib17CEM4890_H2NYMCCXY_SL254843.214	86147	no	Yes			Clostridioides difficile ATCC 9689 = DSM 1296	prophage/temperate/lysogenic	Yes	15.0	0.0
MSP0398	haib17CEM4890_H75CGCCXY_SL263678.59	85986	no	Yes			Acinetobacter baumannii	prophage/temperate/lysogenic	Yes	32.0	1.0
MSP0399	haib17KIU4866_HMCMJCCXY_SL335957.24	85938	no	Yes			Acinetobacter sp. ACNIH1	prophage/temperate/lysogenic	Yes	32.0	0.0
MSP0400	haib17CEM5106_HCVMTCCXY_SL269619.5	85869	no	Yes			Listeria monocytogenes	prophage/temperate/lysogenic	No	22.0	1.0
MSP0401	haib17CEM4890_H2NYMCCXY_SL254839.25	85817	no	Yes			Pseudomonas chlororaphis subsp. aurantiaca	prophage/temperate/lysogenic	Yes	33.0	0.0
MSP0402	haib17CEM4890_H75CGCCXY_SL263653.54	85631	no	Yes	Acinetobacter	Acinetobacter_baumannii	Acinetobacter baumannii	prophage/temperate/lysogenic	Yes	28.0	1.0
MSP0403	haib17CEM4890_H2NYMCCXY_SL254771.133	85572	yes	No				prophage/temperate/lysogenic	Yes	23.0	0.0
MSP0404	haib17CEM4890_HKC32ALXX_SL254725.1	85563	no	No			Pseudomonas aeruginosa	virulent/lytic	Yes	28.0	1.0
MSP0405	haib17CEM4890_H2NYMCCXY_SL254818.136	85542	no	Yes				prophage/temperate/lysogenic	Yes	12.0	0.0
MSP0406	haib17KIU4866_H7HJMCCXY_SL272774.12	85420	yes	No				virulent/lytic	Yes	15.0	0.0
MSP0407	haib17KIU4866_H7HJMCCXY_SL272752.27	85310	no	Yes			Thauera hydrothermalis	prophage/temperate/lysogenic	Yes	33.0	0.0
MSP0408	haib17CEM5106_HCY5HCCXY_SL270961.54	85272	no	Yes				prophage/temperate/lysogenic	Yes	24.0	0.0
MSP0409	haib17CEM4890_H75CGCCXY_SL263724.44	85183	no	Yes			Salmonella enterica subsp. enterica serovar Montevideo str.	prophage/temperate/lysogenic	Yes	30.0	1.0
MSP0410	haib17CEM4890_H2NYMCCXY_SL254783.190	85117	no	Yes				prophage/temperate/lysogenic	Yes	12.0	0.0
MSP0411	haib17CEM4890_H2NYMCCXY_SL254772.182	84845	no	Yes				prophage/temperate/lysogenic	Yes	17.0	0.0
MSP0412	haib17CEM5241_HMGMHCCXY_SL343354.1	84838	yes	No				virulent/lytic	Yes	31.0	0.0
MSP0414	haib17CEM4890_H2NYMCCXY_SL254795.190	84490	no	Yes				prophage/temperate/lysogenic	Yes	14.0	0.0
MSP0415	haib17CEM4890_HKC32ALXX_SL254706.145	84340	no	Yes			Pseudomonas sp. phDV1	prophage/temperate/lysogenic	Yes	33.0	0.0
MSP0417	haib17CEM5106_HCV72CCXY_SL269792.88	84096	no	Yes			Enterococcus faecalis	prophage/temperate/lysogenic	No	25.0	1.0
MSP0418	haib17CEM4890_H75CGCCXY_SL263700.63	84080	no	Yes			Pseudomonas agarici	prophage/temperate/lysogenic	No	31.0	0.0
MSP0419	haib17CEM5106_HCY5JCCXY_SL269583.27	84051	no	Yes	Rhodococcus	Rhodococcus_equi		prophage/temperate/lysogenic	Yes	18.0	0.0
MSP0420	haib17CEM4890_H2NYMCCXY_SL254843.221	84040	no	Yes			Methanotorris igneus Kol 5	prophage/temperate/lysogenic	Yes	17.0	0.0
MSP0421	haib17CEM5106_HCV72CCXY_SL269761.56	83862	no	Yes			Leclercia adecarboxylata	prophage/temperate/lysogenic	No	31.0	0.0
MSP0423	haib17CEM4890_H2NYMCCXY_SL254806.148	83766	yes	No				virulent/lytic	Yes	23.0	0.0
MSP0428	haib17CEM4890_H75CGCCXY_SL263647.59	83420	no	Yes			Acinetobacter sp. WCHA45	prophage/temperate/lysogenic	Yes	33.0	0.0
MSP0429	haib17CEM4890_H75CGCCXY_SL263700.64	83368	no	Yes				prophage/temperate/lysogenic	Yes	31.0	0.0
MSP0430	haib17CEM4890_H2NYMCCXY_SL254808.15	83345	no	Yes			Xanthomonas campestris pv. raphani 756C	prophage/temperate/lysogenic	No	33.0	1.0
MSP0431	haib17CEM5106_HCV72CCXY_SL269754.141	83183	no	Yes			Alloactinosynnema sp. L-07	prophage/temperate/lysogenic	Yes	33.0	0.0
MSP0432	haib17CEM4890_H2NYMCCXY_SL254783.196	83080	no	No			Pseudomonas balearica DSM 6083	prophage/temperate/lysogenic	Yes	21.0	1.0
MSP0433	haib18CEM5453_HNHKFCCXY_SL345930.2	82893	yes	No				virulent/lytic	Yes	20.0	0.0
MSP0434	haib17CEM4890_HKC32ALXX_SL254705.159	82887	no	Yes				prophage/temperate/lysogenic	Yes	15.0	0.0
MSP0435	haib17CEM5106_HCY5JCCXY_SL269566.31	82852	no	Yes	Lactococcus	None	Lactococcus sp. 1JSPR-7	virulent/lytic	Yes	30.0	0.0
MSP0436	haib17CEM5106_HCCGHCCXY_SL270206.1	82611	yes	No				virulent/lytic	Yes	25.0	0.0
MSP0437	haib18CEM5453_HMGN5CCXY_SL344702.7	82582	no	No				virulent/lytic	Yes	16.0	0.0
MSP0438	haib17CEM4890_H2NYMCCXY_SL254861.18	82498	no	Yes			Dickeya dianthicola	prophage/temperate/lysogenic	Yes	28.0	0.0
MSP0440	haib18CEM5453_HMCMJCCXY_SL336225.20	82156	no	Yes			Providencia rettgeri	prophage/temperate/lysogenic	No	24.0	0.0
MSP0441	haib17CEM5106_HCY5HCCXY_SL271123.25	82093	no	Yes				virulent/lytic	Yes	26.0	0.0

MSP0442	haib17CEM4890_H2NYMCCXY_SL254855.157	82063	no	Yes				prophage/temperate/lysogenic	Yes	28.0	0.0
MSP0443	haib17CEM4890_HKC32ALXX_SL254706.152	82053	no	Yes			Acinetobacter radioresistens	prophage/temperate/lysogenic	Yes	32.0	0.0
MSP0444	haib17CEM5106_HCY5JCCXY_SL269599.67	81989	no	Yes			Pseudomonas stutzeri	prophage/temperate/lysogenic	Yes	33.0	1.0
MSP0445	haib17CEM4890_H75CGCCXY_SL263649.67	81910	no	Yes			Pseudomonas aeruginosa 19BR	prophage/temperate/lysogenic	Yes	28.0	1.0
MSP0446	haib17CEM4890_H75CGCCXY_SL263713.57	81710	yes	No				prophage/temperate/lysogenic	Yes	15.0	0.0
MSP0448	haib17CEM5106_HCY5HCCXY_SL270978.76	81683	no	Yes				virulent/lytic	Yes	32.0	0.0
MSP0449	haib17CEM4890_HKC32ALXX_SL254711.29	81436	no	Yes				prophage/temperate/lysogenic	Yes	23.0	0.0
MSP0450	haib17CEM4890_H2NYMCCXY_SL254818.148	81339	no	No				virulent/lytic	Yes	10.0	0.0
MSP0451	haib17KIU4866_H7HJMCCXY_SL272782.46	81324	no	No				virulent/lytic	Yes	17.0	0.0
MSP0452	haib17CEM4890_H75CGCCXY_SL263647.60	81283	no	Yes				prophage/temperate/lysogenic	Yes	32.0	0.0
MSP0453	haib17CEM4890_H75CGCCXY_SL263665.38	81267	no	Yes	Pseudomonas	Pseudomonas_fluorescens	Pseudomonas stutzeri	prophage/temperate/lysogenic	Yes	31.0	0.0
MSP0454	haib17KIU4866_H7HJMCCXY_SL272752.29	81138	no	No				prophage/temperate/lysogenic	Yes	22.0	0.0
MSP0455	haib17CEM5106_HCVMTCCXY_SL269617.92	81101	no	Yes				virulent/lytic	No	33.0	0.0
MSP0456	haib17CEM4890_H75CGCCXY_SL263686.14	81053	yes	No				prophage/temperate/lysogenic	Yes	8.0	0.0
MSP0457	haib17CEM4890_H2NYMCCXY_SL254842.46	80881	no	Yes				prophage/temperate/lysogenic	Yes	15.0	0.0
MSP0458	haib17KIU4866_HMCMJCCXY_SL335963.29	80868	no	Yes				prophage/temperate/lysogenic	Yes	32.0	0.0
MSP0459	haib18CEM5526_HMGTJCCXY_SL342622.65	80721	no	Yes			Salmonella enterica subsp. diarizonae	prophage/temperate/lysogenic	Yes	30.0	1.0
MSP0460	haib17CEM4890_H75CGCCXY_SL263689.67	80611	no	Yes			Pseudomonas aeruginosa	virulent/lytic	Yes	33.0	1.0
MSP0461	haib17CEM5106_HCV72CCXY_SL269786.181	80443	no	No			Klebsiella pneumoniae	prophage/temperate/lysogenic	Yes	32.0	1.0
MSP0463	haib17CEM4890_HKC32ALXX_SL254693.114	80160	no	Yes				prophage/temperate/lysogenic	Yes	10.0	0.0
MSP0465	haib17CEM5106_HCCGHCCXY_SL270257.1	79949	no	Yes				virulent/lytic	Yes	21.0	0.0
MSP0466	haib17CEM5106_HCVMTCCXY_SL269542.46	79902	no	Yes	Staphylococcus	Staphylococcus_epidermidis		prophage/temperate/lysogenic	Yes	33.0	1.0
MSP0467	haib17CEM4890_H2NYMCCXY_SL254781.287	79645	no	Yes	Clostridium	Clostridium_perfringens	Methanococcus voltae A3	prophage/temperate/lysogenic	Yes	11.0	0.0
MSP0468	haib17CEM5241_HMGMHCCXY_SL343449.2	79483	no	No				virulent/lytic	Yes	17.0	0.0
MSP0469	haib17CEM4890_HKC32ALXX_SL254722.26	79461	no	Yes	Xanthomonas	None		virulent/lytic	Yes	23.0	0.0
MSP0470	haib17CEM4890_H2NYMCCXY_SL254832.24	79276	no	No				prophage/temperate/lysogenic	Yes	15.0	0.0
MSP0471	haib17CEM4890_H2NYMCCXY_SL254844.8	79171	yes	No				prophage/temperate/lysogenic	Yes	16.0	0.0
MSP0472	haib17CEM4890_H2NYMCCXY_SL254790.49	79014	no	Yes			Moraxella osloensis	prophage/temperate/lysogenic	Yes	30.0	1.0
MSP0473	haib17CEM4890_H7KYMCCXY_SL273121.2	78847	no	No			Escherichia albertii	virulent/lytic	Yes	12.0	0.0
MSP0474	haib17CEM4890_H75CGCCXY_SL263696.1	78810	yes	No				virulent/lytic	Yes	24.0	0.0
MSP0475	haib17CEM4890_H75CGCCXY_SL263699.61	78782	no	Yes				prophage/temperate/lysogenic	Yes	11.0	0.0
MSP0476	haib17CEM4890_H2NYMCCXY_SL254850.33	78401	no	No				virulent/lytic	Yes	15.0	0.0
MSP0477	haib17KIU4866_HMCMJCCXY_SL335961.3	78260	no	Yes			Serratia plymuthica AS9	prophage/temperate/lysogenic	No	33.0	0.0
MSP0478	haib17CEM5106_HCV72CCXY_SL269767.65	78217	no	Yes	Paracoccus	None		prophage/temperate/lysogenic	Yes	21.0	0.0
MSP0479	haib17KIU4866_H7HJMCCXY_SL272776.72	77994	no	No	Escherichia	Escherichia_coli	Salmonella enterica subsp. enterica serovar Kentucky	prophage/temperate/lysogenic	Yes	8.0	0.0
MSP0481	haib17KIU4866_H7HJMCCXY_SL272786.73	77814	no	Yes				prophage/temperate/lysogenic	Yes	15.0	0.0
MSP0483	haib17CEM5106_HCCGHCCXY_SL270290.17	77755	no	No				virulent/lytic	Yes	23.0	0.0
MSP0484	haib17CEM4890_H2NYMCCXY_SL254784.245	77544	no	Yes				prophage/temperate/lysogenic	Yes	9.0	0.0
MSP0485	haib17CEM5106_HCV72CCXY_SL269754.150	77467	no	Yes			Moraxella osloensis	prophage/temperate/lysogenic	No	25.0	1.0
MSP0486	haib17KIU4866_H7HJMCCXY_SL272823.27	77371	no	Yes			Acinetobacter baumannii	prophage/temperate/lysogenic	Yes	32.0	1.0
MSP0487	haib17CEM4890_H2NYMCCXY_SL254837.14	77336	yes	No				prophage/temperate/lysogenic	Yes	22.0	0.0
MSP0488	haib17CEM4890_H2NYMCCXY_SL254829.161	77324	yes	No				prophage/temperate/lysogenic	Yes	25.0	0.0
MSP0489	haib17CEM4890_HKC32ALXX_SL254718.154	77071	no	Yes			Klebsiella pneumoniae subsp. pneumoniae	prophage/temperate/lysogenic	Yes	32.0	1.0
MSP0491	haib17CEM4890_HKC32ALXX_SL254700.40	76991	no	No			Cutibacterium granulosum	virulent/lytic	Yes	29.0	1.0
MSP0492	haib17KIU4866_H7HJMCCXY_SL272826.77	76981	no	Yes				prophage/temperate/lysogenic	Yes	14.0	0.0
MSP0493	haib17CEM4890_HKC32ALXX_SL254706.169	76964	no	Yes			Citrobacter koseri	prophage/temperate/lysogenic	Yes	30.0	0.0
MSP0494	haib17CEM4890_H2NYMCCXY_SL254772.209	76933	no	Yes				prophage/temperate/lysogenic	No	14.0	0.0
MSP0495	haib17CEM4890_H2NYMCCXY_SL254848.34	76883	yes	No				prophage/temperate/lysogenic	Yes	13.0	0.0
MSP0496	haib17CEM4890_H2NYMCCXY_SL254778.26	76830	no	Yes				prophage/temperate/lysogenic	Yes	30.0	0.0
MSP0497	haib17CEM5106_HCVMTCCXY_SL269571.72	76714	no	Yes				prophage/temperate/lysogenic	Yes	31.0	0.0
MSP0498	haib17DB4959_H3MGVCCXY_SL259985.15	76642	no	No				virulent/lytic	Yes	24.0	0.0
MSP0499	haib17CEM5106_HCY5HCCXY_SL270916.44	76531	no	Yes				prophage/temperate/lysogenic	Yes	31.0	0.0
MSP0500	haib17CEM4890_H2NYMCCXY_SL254818.165	76528	no	No				virulent/lytic	Yes	14.0	0.0
MSP0501	haib17KIU4866_H7HJMCCXY_SL272796.104	76275	no	Yes	Klebsiella	Klebsiella_pneumoniae	Dickeya chrysanthemi Ech1591	prophage/temperate/lysogenic	No	33.0	0.0
MSP0502	haib17KIU4866_H7HJMCCXY_SL272823.29	76265	no	Yes			Acinetobacter sp. LoGeW2-3	prophage/temperate/lysogenic	Yes	32.0	0.0
MSP0503	haib17CEM4890_H75CGCCXY_SL263652.126	76086	no	Yes				virulent/lytic	Yes	32.0	0.0
MSP0504	haib17KIU4866_H7HJMCCXY_SL272794.18	76073	no	Yes				prophage/temperate/lysogenic	Yes	33.0	0.0
MSP0505	haib17KIU4866_H7HJMCCXY_SL272762.19	76008	no	Yes			Delftia acidovorans	prophage/temperate/lysogenic	Yes	28.0	0.0
MSP0506	haib17CEM5106_HCY5HCCXY_SL270911.68	75954	no	No			Pseudomonas chlororaphis subsp. aurantiaca	prophage/temperate/lysogenic	Yes	20.0	0.0
MSP0507	haib17CEM4890_HKC32ALXX_SL254742.179	75900	no	Yes				prophage/temperate/lysogenic	Yes	31.0	0.0
MSP0509	haib17CEM4890_H2NYMCCXY_SL254794.339	75868	no	Yes			Klebsiella pneumoniae	prophage/temperate/lysogenic	Yes	23.0	1.0
MSP0510	haib17CEM4890_H2NYMCCXY_SL254793.89	75784	no	Yes				prophage/temperate/lysogenic	No	33.0	0.0
MSP0511	haib17CEM5106_HCY5HCCXY_SL270975.77	75732	no	Yes			Pseudomonas fluorescens	prophage/temperate/lysogenic	Yes	33.0	1.0
MSP0512	haib17CEM5106_HCY5JCCXY_SL269560.18	75620	no	Yes			Pseudomonas stutzeri A1501	virulent/lytic	Yes	30.0	1.0
MSP0514	haib17CEM4890_H2NYMCCXY_SL254818.172	75556	no	Yes				prophage/temperate/lysogenic	Yes	31.0	0.0
MSP0515	haib17CEM5106_HCVMTCCXY_SL269598.69	75544	no	Yes				prophage/temperate/lysogenic	Yes	33.0	0.0
MSP0516	haib17CEM4890_H2NYMCCXY_SL254826.75	75394	no	Yes			Cronobacter muytjensii ATCC 51329	prophage/temperate/lysogenic	Yes	27.0	0.0
MSP0517	haib17KIU4866_H7HJMCCXY_SL272757.38	75273	no	Yes			Clostridium sp. JN-9	prophage/temperate/lysogenic	Yes	20.0	0.0

MSP0518	haib18CEM5453_HNGH3CCXY_SL344575.1	75205	no	No				virulent/lytic	Yes	23.0	0.0
MSP0519	haib17CEM5106_HCV72CCXY_SL269730.126	75190	no	Yes				prophage/temperate/lysogenic	Yes	32.0	0.0
MSP0520	haib17CEM4890_H75CGCCXY_SL263654.48	75186	no	Yes			Xanthomonas vasicola pv. vasculorum	prophage/temperate/lysogenic	Yes	23.0	0.0
MSP0521	haib17CEM4890_H2NYMCCXY_SL254852.94	75159	no	Yes	Staphylococcus	Staphylococcus_pseudintermedius	Staphylococcus pseudintermedius	prophage/temperate/lysogenic	No	19.0	0.0
MSP0522	haib17KIU4866_H7HJMCCXY_SL272775.24	74921	no	Yes			Pectobacterium parmentieri	prophage/temperate/lysogenic	Yes	33.0	0.0
MSP0523	haib17CEM5106_HCY5HCCXY_SL270971.72	74916	no	Yes	Lactococcus	Lactococcus_lactis	Streptococcus thermophilus ND03	prophage/temperate/lysogenic	No	29.0	0.0
MSP0524	haib17KIU4866_H7HJMCCXY_SL272769.3	74845	yes	No	Pseudomonas	Pseudomonas_fluorescens		prophage/temperate/lysogenic	Yes	21.0	1.0
MSP0526	haib17KIU4866_H7HJMCCXY_SL272768.29	74800	no	No	Pseudomonas	Pseudomonas_fluorescens		prophage/temperate/lysogenic	Yes	20.0	1.0
MSP0527	haib17CEM5106_HCV72CCXY_SL269785.80	74694	no	Yes			Acidipropionibacterium virtanenii	prophage/temperate/lysogenic	Yes	32.0	0.0
MSP0528	haib17CEM4890_H75CGCCXY_SL263716.39	74679	yes	No				prophage/temperate/lysogenic	Yes	15.0	0.0
MSP0529	haib17CEM5106_HCY5HCCXY_SL270976.125	74414	no	Yes	Gordonia	Gordonia_terrae	Streptomyces gilvosporeus	prophage/temperate/lysogenic	Yes	29.0	0.0
MSP0530	haib17CEM4890_H75CGCCXY_SL263716.41	74239	no	Yes				prophage/temperate/lysogenic	Yes	33.0	0.0
MSP0531	haib17CEM4890_H2NYMCCXY_SL254791.34	74202	no	Yes				virulent/lytic	Yes	33.0	0.0
MSP0533	haib17CEM5106_HCY5HCCXY_SL270975.80	74070	no	Yes				prophage/temperate/lysogenic	No	29.0	0.0
MSP0534	haib17CEM5106_HCY5JCCXY_SL269535.116	74056	no	Yes				prophage/temperate/lysogenic	Yes	31.0	0.0
MSP0535	haib17CEM4890_H2NYMCCXY_SL254830.193	74049	yes	No				virulent/lytic	Yes	13.0	0.0
MSP0536	haib17CEM5106_HCV72CCXY_SL269759.17	73858	no	Yes				virulent/lytic	Yes	30.0	0.0
MSP0537	haib17CEM4890_HKC32ALXX_SL254706.181	73846	no	Yes			Citrobacter koseri	prophage/temperate/lysogenic	Yes	30.0	0.0
MSP0538	haib17CEM4890_HMCMJCCXY_SL335794.20	73656	no	No			Saccharopolyspora erythraea NRRL 2338	prophage/temperate/lysogenic	Yes	20.0	0.0
MSP0539	haib17CEM4890_H2NYMCCXY_SL254806.178	73396	no	Yes	Escherichia	None	Serratia marcescens	virulent/lytic	No	25.0	0.0
MSP0540	haib17CEM5106_HCV72CCXY_SL269733.121	73340	no	Yes			Saccharomonospora glauca K62	prophage/temperate/lysogenic	Yes	32.0	0.0
MSP0541	haib17KIU4866_H7HJMCCXY_SL272743.25	73281	no	Yes				prophage/temperate/lysogenic	Yes	30.0	0.0
MSP0542	haib17CEM5106_HCVMTCCXY_SL269566.40	73280	no	Yes				prophage/temperate/lysogenic	Yes	29.0	0.0
MSP0543	haib17CEM4890_H2NYMCCXY_SL254855.176	73230	no	Yes				prophage/temperate/lysogenic	Yes	13.0	0.0
MSP0544	haib17CEM4890_H2NYMCCXY_SL254782.167	73046	no	Yes				prophage/temperate/lysogenic	Yes	32.0	0.0
MSP0545	haib17CEM4890_H75CGCCXY_SL263690.39	73026	no	Yes				prophage/temperate/lysogenic	Yes	33.0	0.0
MSP0546	haib18CEM5453_HNHKFCCXY_SL345590.3	72918	no	Yes				prophage/temperate/lysogenic	Yes	32.0	0.0
MSP0547	haib17CEM4890_H2NYMCCXY_SL254849.42	72858	no	No				virulent/lytic	Yes	29.0	0.0
MSP0548	haib17CEM5106_HCY5JCCXY_SL269564.22	72820	no	Yes				prophage/temperate/lysogenic	Yes	17.0	0.0
MSP0549	haib17CEM5106_HCV72CCXY_SL269792.109	72790	no	Yes				prophage/temperate/lysogenic	Yes	33.0	0.0
MSP0550	haib17CEM5106_HCY5HCCXY_SL270942.58	72662	no	Yes			Delftia acidovorans	prophage/temperate/lysogenic	Yes	26.0	0.0
MSP0552	haib17KIU4866_H7HJMCCXY_SL272765.42	72334	no	Yes				prophage/temperate/lysogenic	Yes	12.0	0.0
MSP0553	haib17CEM4890_H75CGCCXY_SL263641.83	72316	no	Yes			Pseudomonas chlororaphis subsp. aurantiaca	prophage/temperate/lysogenic	Yes	33.0	0.0
MSP0554	haib17CEM4890_H2NYMCCXY_SL254831.218	72305	no	Yes			Neisseria lactamica 020-06	prophage/temperate/lysogenic	No	33.0	0.0
MSP0555	haib17KIU4866_HMCMJCCXY_SL335938.64	72279	no	Yes			Dickeya zeae NCPPB 3531	virulent/lytic	No	24.0	0.0
MSP0556	haib17CEM4890_HKC32ALXX_SL254693.130	72153	no	Yes				prophage/temperate/lysogenic	Yes	13.0	0.0
MSP0557	haib17CEM5106_HCCGHCCXY_SL270210.6	72067	yes	No	Corynebacterium	Corynebacterium_vitaeruminis		virulent/lytic	Yes	21.0	0.0
MSP0558	haib17CEM4890_H2NYMCCXY_SL254817.142	72030	no	Yes			Peptoniphilus harei	prophage/temperate/lysogenic	Yes	12.0	0.0
MSP0559	haib17KIU4866_H7HJMCCXY_SL272804.30	72023	no	Yes	Cronobacter	Cronobacter_sakazakii	Pectobacterium parmentieri WPP163	prophage/temperate/lysogenic	No	33.0	0.0
MSP0560	haib17CEM4890_HMCMJCCXY_SL335833.121	72005	no	Yes				prophage/temperate/lysogenic	Yes	33.0	0.0
MSP0561	haib17CEM5106_HCV72CCXY_SL269772.35	71980	no	No			Salmonella enterica subsp. enterica	prophage/temperate/lysogenic	Yes	19.0	1.0
MSP0562	haib17CEM4890_HKC32ALXX_SL254706.192	71961	no	Yes			Pectobacterium versatile	prophage/temperate/lysogenic	No	30.0	0.0
MSP0563	haib17CEM4890_H2NYMCCXY_SL254807.123	71880	no	Yes				prophage/temperate/lysogenic	Yes	5.0	0.0
MSP0564	haib17CEM4890_H2NYMCCXY_SL254771.178	71843	no	Yes			Salmonella enterica subsp. diarizonae serovar 48:i:z	prophage/temperate/lysogenic	Yes	29.0	1.0
MSP0565	haib17CEM4890_H2NYMCCXY_SL254853.71	71832	no	Yes			Acinetobacter sp. ACNIH1	prophage/temperate/lysogenic	Yes	32.0	0.0
MSP0566	haib17KIU4866_H7HJMCCXY_SL272770.42	71812	no	Yes				prophage/temperate/lysogenic	Yes	11.0	0.0
MSP0567	haib17CEM5106_HCV72CCXY_SL269754.168	71808	no	Yes			Gordonia sp. YC-JH1	prophage/temperate/lysogenic	Yes	32.0	0.0
MSP0568	haib17CEM4890_H2NYMCCXY_SL254818.185	71787	yes	No				prophage/temperate/lysogenic	Yes	11.0	0.0
MSP0569	haib17CEM4890_HKC32ALXX_SL254676.41	71639	no	Yes				prophage/temperate/lysogenic	Yes	14.0	0.0
MSP0570	haib17CEM5106_HCVMTCCXY_SL269559.66	71626	no	Yes				prophage/temperate/lysogenic	Yes	33.0	0.0
MSP0571	haib17DB4959_HMCMJCCXY_SL336180.1	71592	yes	No				virulent/lytic	Yes	23.0	0.0
MSP0572	haib17CEM4890_H2NYMCCXY_SL254841.133	71402	no	Yes				prophage/temperate/lysogenic	Yes	14.0	0.0
MSP0573	haib17CEM5106_HCV72CCXY_SL269722.20	71331	no	Yes	Pseudomonas	Pseudomonas_aeruginosa	Pseudomonas alcaliphila JAB1	virulent/lytic	Yes	33.0	0.0
MSP0574	haib17CEM4890_HKC32ALXX_SL254677.1	71075	no	No				prophage/temperate/lysogenic	Yes	10.0	0.0
MSP0575	haib18CEM5453_HT5YVCCXY_SL357286.1	70856	no	No				virulent/lytic	Yes	19.0	0.0
MSP0576	haib17CEM4890_HKC32ALXX_SL254696.4	70850	no	Yes				virulent/lytic	Yes	16.0	0.0
MSP0577	haib17CEM4890_H75CGCCXY_SL263688.130	70737	no	Yes			Salmonella enterica subsp. enterica serovar Wandsworth str.	prophage/temperate/lysogenic	Yes	21.0	1.0
MSP0578	haib17CEM5106_HCV72CCXY_SL269718.84	70640	no	Yes				prophage/temperate/lysogenic	Yes	23.0	0.0
MSP0579	haib17CEM4890_HKC32ALXX_SL254707.11	70625	no	No			Micrococcus luteus	virulent/lytic	Yes	32.0	1.0
MSP0580	haib17CEM4890_H75CGCCXY_SL263726.16	70362	no	Yes				prophage/temperate/lysogenic	Yes	28.0	0.0
MSP0581	haib17KIU4866_H7HJMCCXY_SL272827.63	70352	no	Yes			Acinetobacter indicus	prophage/temperate/lysogenic	Yes	32.0	0.0
MSP0582	haib17CEM4890_H2NYMCCXY_SL254769.178	70309	no	Yes				prophage/temperate/lysogenic	Yes	14.0	0.0
MSP0583	haib17KIU4866_H7HJMCCXY_SL272826.95	70286	no	Yes			Pseudomonas aeruginosa	prophage/temperate/lysogenic	Yes	19.0	1.0
MSP0584	haib17CEM5106_HCVMTCCXY_SL269527.39	70127	no	Yes			Lactococcus sp. 1JSPR-7	virulent/lytic	No	28.0	0.0
MSP0586	haib17CEM5106_HCVMTCCXY_SL269607.96	70081	no	Yes	Enterococcus	Enterococcus_faecalis		prophage/temperate/lysogenic	No	29.0	1.0
MSP0587	haib17CEM4890_H2NYMCCXY_SL254848.37	70069	no	Yes	Staphylococcus	Staphylococcus_aureus	Bacillus halodurans C-125	prophage/temperate/lysogenic	No	33.0	0.0
MSP0588	haib17CEM4890_H2NYMCCXY_SL254794.375	69987	no	Yes				prophage/temperate/lysogenic	Yes	14.0	0.0
MSP0589	haib17CEM4890_H2NYMCCXY_SL254790.62	69960	no	No				prophage/temperate/lysogenic	Yes	17.0	0.0

MSP0590	haib18CEM5453_HMCMJCCXY_SL336349.1	69873	no	No			Klebsiella pneumoniae subsp. pneumoniae	prophage/temperate/lysogenic	Yes	32.0	1.0
MSP0591	haib17CEM5106_HCVMTCCXY_SL269598.78	69866	no	Yes				prophage/temperate/lysogenic	Yes	17.0	0.0
MSP0592	haib17CEM5106_HCY5JCCXY_SL269570.35	69813	no	Yes				prophage/temperate/lysogenic	Yes	28.0	0.0
MSP0593	haib17CEM4890_HKC32ALXX_SL254694.83	69647	no	Yes			Pasteurella multocida	prophage/temperate/lysogenic	Yes	15.0	0.0
MSP0594	haib17KIU4866_H7HJMCCXY_SL272778.71	69643	no	Yes				prophage/temperate/lysogenic	Yes	32.0	0.0
MSP0595	haib17CEM4890_HKC32ALXX_SL254723.1	69521	no	No				prophage/temperate/lysogenic	Yes	18.0	0.0
MSP0596	haib17CEM5106_HCY5JCCXY_SL269601.55	69436	no	Yes				prophage/temperate/lysogenic	Yes	33.0	0.0
MSP0598	haib17CEM4890_H2NYMCCXY_SL254770.147	69395	no	No				prophage/temperate/lysogenic	Yes	33.0	0.0
MSP0599	haib17CEM5106_HCY5HCCXY_SL270911.76	69385	no	Yes				virulent/lytic	Yes	33.0	0.0
MSP0600	haib17CEM4890_H75CGCCXY_SL263639.82	69316	no	Yes			Pseudomonas chlororaphis subsp. aurantiaca	prophage/temperate/lysogenic	Yes	33.0	0.0
MSP0601	haib17CEM4890_H2NYMCCXY_SL254806.197	69300	no	Yes				prophage/temperate/lysogenic	Yes	13.0	0.0
MSP0602	haib17CEM4890_HKC32ALXX_SL254728.210	69295	no	No	Bacillus	Bacillus_thuringiensis		prophage/temperate/lysogenic	Yes	9.0	0.0
MSP0603	haib17CEM4890_HMCMJCCXY_SL335794.24	69225	no	Yes	Pseudomonas	Pseudomonas_fluorescens		prophage/temperate/lysogenic	Yes	33.0	1.0
MSP0604	haib17CEM4890_HKC32ALXX_SL254688.9	69190	no	No				virulent/lytic	Yes	9.0	0.0
MSP0605	haib17CEM4890_H2NYMCCXY_SL254842.61	69137	no	Yes				prophage/temperate/lysogenic	Yes	14.0	0.0
MSP0606	haib17CEM5106_HCVMTCCXY_SL269561.50	69117	no	Yes			Xanthomonas campestris pv. raphani 756C	prophage/temperate/lysogenic	Yes	33.0	1.0
MSP0608	haib18CEM5453_HNHKFCCXY_SL345930.3	68886	no	No				virulent/lytic	Yes	20.0	0.0
MSP0609	haib17CEM5106_HCY5JCCXY_SL269600.74	68758	no	Yes				prophage/temperate/lysogenic	Yes	21.0	0.0
MSP0610	haib17CEM4890_HKC32ALXX_SL254706.209	68672	no	Yes				prophage/temperate/lysogenic	Yes	13.0	0.0
MSP0611	haib17CEM5106_HCY5HCCXY_SL270934.71	68660	no	Yes				prophage/temperate/lysogenic	Yes	31.0	0.0
MSP0612	haib18CEM5332_HK7G5CCXY_SL311017.28	68619	no	Yes				prophage/temperate/lysogenic	Yes	33.0	0.0
MSP0613	haib17KIU4866_HMCMJCCXY_SL335947.77	68603	no	Yes				prophage/temperate/lysogenic	Yes	32.0	0.0
MSP0614	haib17CEM4890_H2NYMCCXY_SL254856.46	68600	no	Yes				virulent/lytic	Yes	32.0	0.0
MSP0615	haib17KIU4866_H7HJMCCXY_SL272776.85	68576	no	Yes			Serratia plymuthica AS9	prophage/temperate/lysogenic	Yes	30.0	0.0
MSP0616	haib17CEM4890_H2NYMCCXY_SL254790.67	68572	yes	No				virulent/lytic	Yes	29.0	0.0
MSP0617	haib17CEM4890_HKC32ALXX_SL254705.205	68383	no	Yes				prophage/temperate/lysogenic	Yes	16.0	0.0
MSP0618	haib17CEM4890_H2NYMCCXY_SL254772.236	68364	no	Yes				prophage/temperate/lysogenic	Yes	11.0	0.0
MSP0619	haib17CEM4890_H2NYMCCXY_SL254784.301	68312	no	Yes			Acinetobacter radioresistens	prophage/temperate/lysogenic	Yes	32.0	0.0
MSP0621	haib17CEM4890_H2NYMCCXY_SL254861.30	68243	no	No				prophage/temperate/lysogenic	Yes	10.0	0.0
MSP0623	haib17CEM5106_HCY5HCCXY_SL270914.63	68009	no	Yes				prophage/temperate/lysogenic	Yes	31.0	0.0
MSP0624	haib17KIU4866_H7HJMCCXY_SL272790.9	67988	no	Yes			Pseudomonas stutzeri A1501	prophage/temperate/lysogenic	No	30.0	1.0
MSP0625	haib17CEM4890_H2NYMCCXY_SL254772.237	67943	no	Yes				prophage/temperate/lysogenic	Yes	12.0	0.0
MSP0626	haib17KIU4866_H7HJMCCXY_SL272776.86	67908	no	Yes				prophage/temperate/lysogenic	Yes	5.0	0.0
MSP0627	haib17CEM5106_HCV72CCXY_SL269748.219	67886	no	Yes				prophage/temperate/lysogenic	Yes	28.0	0.0
MSP0628	haib17CEM4890_H2NYMCCXY_SL254769.187	67837	no	Yes			Cronobacter sakazakii	virulent/lytic	Yes	30.0	0.0
MSP0629	haib17CEM4890_H2NYMCCXY_SL254825.12	67769	no	No				virulent/lytic	Yes	22.0	0.0
MSP0630	haib17CEM5106_HCVMTCCXY_SL269598.83	67764	no	Yes				prophage/temperate/lysogenic	Yes	14.0	0.0
MSP0632	haib17CEM5106_HCV72CCXY_SL269786.214	67699	no	Yes			Aeromonas veronii	prophage/temperate/lysogenic	Yes	22.0	0.0
MSP0633	haib17CEM5106_HCV72CCXY_SL269752.122	67691	no	Yes			Propionibacterium freudenreichii	prophage/temperate/lysogenic	Yes	32.0	0.0
MSP0634	haib17CEM5106_HCY5HCCXY_SL270942.66	67590	no	Yes				prophage/temperate/lysogenic	Yes	30.0	0.0
MSP0635	haib17KIU4866_H7HJMCCXY_SL272765.47	67322	yes	No				prophage/temperate/lysogenic	Yes	8.0	0.0
MSP0636	haib18CEM5453_HMGW3CCXY_SL342525.1	67232	yes	No				virulent/lytic	Yes	5.0	0.0
MSP0637	haib17CEM4890_H2NYMCCXY_SL254788.51	67224	no	Yes				prophage/temperate/lysogenic	Yes	30.0	0.0
MSP0638	haib17CEM5106_HCV72CCXY_SL269800.44	67217	no	Yes			Clostridium perfringens	prophage/temperate/lysogenic	Yes	20.0	0.0
MSP0639	haib17KIU4866_H7HJMCCXY_SL272775.34	67071	no	Yes			Salmonella enterica subsp. enterica serovar Tennessee	prophage/temperate/lysogenic	Yes	29.0	1.0
MSP0640	haib17KIU4866_H7HJMCCXY_SL272801.3	67070	no	No				prophage/temperate/lysogenic	Yes	12.0	0.0
MSP0642	haib17CEM4890_HMCMJCCXY_SL335795.37	66728	no	Yes			Pseudomonas pseudoalcaligenes CECT 5344	virulent/lytic	Yes	33.0	0.0
MSP0643	haib17CEM4890_H75CGCCXY_SL263649.79	66637	no	Yes			Dickeya sp. CSL RW240	prophage/temperate/lysogenic	Yes	26.0	0.0
MSP0644	haib17CEM4890_H2NYMCCXY_SL254794.405	66605	no	Yes				prophage/temperate/lysogenic	Yes	24.0	0.0
MSP0645	haib17CEM4890_H7KYMCCXY_SL273103.72	66585	no	No				virulent/lytic	Yes	22.0	0.0
MSP0646	haib17CEM4890_H2NYMCCXY_SL254831.235	66515	no	Yes				prophage/temperate/lysogenic	Yes	15.0	0.0
MSP0647	haib17KIU4866_HMCMJCCXY_SL335956.83	66503	no	Yes				prophage/temperate/lysogenic	Yes	17.0	0.0
MSP0648	haib17CEM4890_HKC32ALXX_SL254739.7	66480	no	No	Corynebacterium	Corynebacterium_vitaeruminis		virulent/lytic	Yes	27.0	0.0
MSP0650	haib17CEM4890_H75CGCCXY_SL263665.48	66410	no	Yes			Acinetobacter indicus	prophage/temperate/lysogenic	Yes	32.0	0.0
MSP0651	haib17CEM4890_H2NYMCCXY_SL254771.196	66396	no	Yes			Acinetobacter sp. WCHA45	prophage/temperate/lysogenic	Yes	32.0	0.0
MSP0652	haib17CEM4890_H2NYMCCXY_SL254855.190	66366	no	Yes				prophage/temperate/lysogenic	Yes	29.0	0.0
MSP0653	haib17CEM5106_HCV72CCXY_SL269799.36	66336	no	Yes				prophage/temperate/lysogenic	Yes	28.0	0.0
MSP0654	haib17CEM5106_HCV72CCXY_SL269759.20	66325	no	Yes				virulent/lytic	Yes	27.0	0.0
MSP0655	haib18CEM5332_HMCMJCCXY_SL335770.26	66257	no	No	Corynebacterium	Corynebacterium_vitaeruminis		virulent/lytic	Yes	21.0	0.0
MSP0656	haib17DB4959_H3MGVCCXY_SL259987.17	66247	no	Yes	Staphylococcus	Staphylococcus_aureus	Bacillus halodurans C-125	prophage/temperate/lysogenic	No	32.0	0.0
MSP0657	haib17KIU4866_H7HJMCCXY_SL272768.40	66207	no	No				virulent/lytic	Yes	12.0	0.0
MSP0658	haib17CEM4890_H2NYMCCXY_SL254818.216	66172	no	No				virulent/lytic	Yes	11.0	0.0
MSP0659	haib17CEM4890_H2NYMCCXY_SL254805.219	66102	no	Yes				prophage/temperate/lysogenic	Yes	32.0	0.0
MSP0660	haib17CEM5106_HCVMTCCXY_SL269541.31	66099	no	Yes				prophage/temperate/lysogenic	Yes	33.0	0.0
MSP0661	haib18CEM5453_HNHKFCCXY_SL346109.10	66094	no	No				virulent/lytic	Yes	19.0	0.0
MSP0662	haib17CEM5106_HCV72CCXY_SL269783.52	66083	no	Yes	Arthrobacter	Arthrobacter_globiformis		prophage/temperate/lysogenic	Yes	32.0	0.0
MSP0663	haib17CEM4890_H2NYMCCXY_SL254818.217	65991	no	No				prophage/temperate/lysogenic	Yes	33.0	0.0
MSP0664	haib17CEM4890_HKC32ALXX_SL254752.106	65954	no	No			Candidatus Tenderia electrophaga	prophage/temperate/lysogenic	Yes	16.0	0.0

MSP0665	haib17CEM5106_HCV72CCXY_SL269744.158	65953	no	Yes				prophage/temperate/lysogenic	Yes	29.0	0.0
MSP0666	haib17CEM4890_H7KYMCCXY_SL273060.34	65951	no	No	Microbacterium	Microbacterium_foliorum		virulent/lytic	No	21.0	0.0
MSP0667	haib17CEM4890_H2NYMCCXY_SL254856.49	65933	no	No			Acinetobacter junii	prophage/temperate/lysogenic	Yes	25.0	1.0
MSP0668	haib17CEM4890_H75CGCCXY_SL263699.79	65924	no	Yes				prophage/temperate/lysogenic	Yes	10.0	0.0
MSP0669	haib17CEM5106_HCV72CCXY_SL269788.197	65865	no	Yes			Corynebacterium flavescens	virulent/lytic	Yes	28.0	0.0
MSP0670	haib17CEM4890_HKC32ALXX_SL254681.232	65851	no	Yes				prophage/temperate/lysogenic	Yes	13.0	0.0
MSP0672	haib17CEM4890_H2NYMCCXY_SL254770.165	65695	no	Yes				prophage/temperate/lysogenic	Yes	11.0	0.0
MSP0673	haib17CEM4890_HKC32ALXX_SL254681.233	65691	no	Yes			Clostridium sp. JN-9	prophage/temperate/lysogenic	Yes	15.0	0.0
MSP0674	haib17CEM5106_HCY5HCCXY_SL270978.100	65610	no	Yes				prophage/temperate/lysogenic	Yes	28.0	0.0
MSP0675	haib17CEM4890_HKC32ALXX_SL254681.234	65548	no	Yes				prophage/temperate/lysogenic	Yes	21.0	0.0
MSP0676	haib17CEM5106_HCY5JCCXY_SL269595.66	65527	no	No				virulent/lytic	Yes	21.0	0.0
MSP0677	haib17CEM4890_H2NYMCCXY_SL254809.7	65481	no	No				virulent/lytic	Yes	18.0	0.0
MSP0678	haib17CEM4890_H2NYMCCXY_SL254782.190	65386	no	Yes				prophage/temperate/lysogenic	Yes	17.0	0.0
MSP0680	haib17CEM5106_HCV72CCXY_SL269777.133	65324	no	Yes				prophage/temperate/lysogenic	Yes	14.0	0.0
MSP0681	haib17CEM4890_HKC32ALXX_SL254706.226	65317	no	Yes	Cronobacter	Cronobacter_sakazakii	Serratia plymuthica AS9	prophage/temperate/lysogenic	No	25.0	0.0
MSP0682	haib17CEM4890_H2NYMCCXY_SL254772.258	65297	no	No				virulent/lytic	Yes	6.0	0.0
MSP0683	haib17DB4959_H3MGVCCXY_SL259882.38	65296	no	Yes			Blastochloris viridis	prophage/temperate/lysogenic	Yes	5.0	0.0
MSP0684	haib17CEM4890_H75CGCCXY_SL263723.3	65122	no	Yes				prophage/temperate/lysogenic	Yes	21.0	0.0
MSP0685	haib17KIU4866_H7HJMCCXY_SL272751.61	65102	no	Yes				prophage/temperate/lysogenic	Yes	13.0	0.0
MSP0686	haib18CEM5332_HMCMJCCXY_SL335741.10	64960	no	Yes				prophage/temperate/lysogenic	Yes	32.0	0.0
MSP0687	haib17CEM4890_H75CGCCXY_SL263675.83	64942	no	Yes				prophage/temperate/lysogenic	Yes	32.0	0.0
MSP0688	haib17CEM4890_H2NYMCCXY_SL254772.260	64864	no	Yes				virulent/lytic	Yes	15.0	0.0
MSP0689	haib17KIU4866_HMCMJCCXY_SL335946.29	64802	no	Yes			Salmonella enterica subsp. enterica serovar Waycross	prophage/temperate/lysogenic	Yes	27.0	1.0
MSP0690	haib17CEM4890_H2NYMCCXY_SL254772.261	64669	no	No				virulent/lytic	Yes	12.0	0.0
MSP0691	haib17CEM4890_H2NYMCCXY_SL254850.49	64646	no	No				virulent/lytic	Yes	33.0	0.0
MSP0692	haib17CEM4890_HKC32ALXX_SL254681.236	64630	no	No				prophage/temperate/lysogenic	Yes	8.0	0.0
MSP0693	haib17KIU4866_H7HJMCCXY_SL272802.18	64584	no	Yes				prophage/temperate/lysogenic	Yes	32.0	0.0
MSP0694	haib17CEM4890_H3KHWCCXY_SL263333.1	64479	yes	No			Leptolyngbya sp. O-77	virulent/lytic	Yes	13.0	0.0
MSP0695	haib17KIU4866_HMCMJCCXY_SL335952.37	64445	no	Yes			Acinetobacter sp. ACNIH1	prophage/temperate/lysogenic	Yes	31.0	0.0
MSP0696	haib17CEM4890_H2NYMCCXY_SL254806.221	64432	yes	No			Aromatoleum aromaticum EbN1	prophage/temperate/lysogenic	Yes	16.0	0.0
MSP0697	haib17CEM4890_H75CGCCXY_SL263677.58	64363	no	Yes				prophage/temperate/lysogenic	Yes	30.0	0.0
MSP0698	haib17CEM4890_HKC32ALXX_SL254728.234	64340	yes	No				prophage/temperate/lysogenic	Yes	8.0	0.0
MSP0699	haib17CEM5106_HCY5HCCXY_SL270943.26	64300	no	Yes				virulent/lytic	Yes	29.0	0.0
MSP0700	haib17CEM5106_HCVMTCCXY_SL269600.77	64232	no	Yes			Alkalihalobacillus clausii	prophage/temperate/lysogenic	Yes	14.0	0.0
MSP0701	haib17CEM4890_H2NYMCCXY_SL254848.44	64158	yes	No				virulent/lytic	Yes	6.0	0.0
MSP0702	haib18CEM5453_HT5YVCCXY_SL357286.3	64127	no	No				virulent/lytic	Yes	16.0	0.0
MSP0703	haib17CEM5106_HCV72CCXY_SL269748.230	64103	no	Yes				prophage/temperate/lysogenic	Yes	10.0	0.0
MSP0704	haib17CEM5106_HCVMTCCXY_SL269588.29	64089	no	Yes				prophage/temperate/lysogenic	Yes	28.0	0.0
MSP0705	haib17CEM4890_HKC32ALXX_SL254764.216	63871	yes	No				prophage/temperate/lysogenic	Yes	8.0	0.0
MSP0706	haib18CEM5453_HNGH3CCXY_SL344536.1	63847	no	No				virulent/lytic	Yes	32.0	0.0
MSP0707	haib17CEM4890_H75CGCCXY_SL263654.60	63836	no	Yes			Citrobacter rodentium ICC168	prophage/temperate/lysogenic	Yes	30.0	0.0
MSP0708	haib17KIU4866_H7HJMCCXY_SL272826.119	63742	no	Yes			Pigmentiphaga sp. H8	prophage/temperate/lysogenic	Yes	31.0	0.0
MSP0709	haib17CEM5106_HCVMTCCXY_SL269609.72	63739	no	Yes			Enterococcus faecalis	prophage/temperate/lysogenic	No	32.0	1.0
MSP0711	haib17CEM4890_H2NYMCCXY_SL254796.185	63695	no	Yes				prophage/temperate/lysogenic	Yes	28.0	0.0
MSP0710	haib17CEM4890_H2NYMCCXY_SL254829.199	63695	no	Yes			Pseudomonas stutzeri	virulent/lytic	Yes	33.0	1.0
MSP0712	haib17CEM4890_HKC32ALXX_SL254712.14	63690	yes	No			Granulibacter bethesdensis	prophage/temperate/lysogenic	Yes	16.0	0.0
MSP0713	haib17KIU4866_HMCMJCCXY_SL335961.8	63684	no	Yes			Acinetobacter sp. LoGeW2-3	prophage/temperate/lysogenic	Yes	25.0	0.0
MSP0714	haib17CEM4890_H2NYMCCXY_SL254806.230	63664	no	No	Bacillus	Bacillus_cereus		prophage/temperate/lysogenic	Yes	10.0	0.0
MSP0715	haib17KIU4866_H7HJMCCXY_SL272820.19	63613	yes	No				prophage/temperate/lysogenic	Yes	10.0	0.0
MSP0716	haib17KIU4866_HMCMJCCXY_SL335942.37	63544	no	Yes				virulent/lytic	Yes	24.0	0.0
MSP0717	haib18CEM5453_HMGN5CCXY_SL344674.1	63542	no	No				virulent/lytic	Yes	21.0	0.0
MSP0718	haib18CEM5453_HMC2KCCXY_SL336755.37	63518	no	No				virulent/lytic	Yes	16.0	0.0
MSP0719	haib17CEM5106_HCY5HCCXY_SL270938.86	63515	no	Yes				prophage/temperate/lysogenic	Yes	30.0	0.0
MSP0720	haib17CEM4890_H2NYMCCXY_SL254854.137	63395	no	No				prophage/temperate/lysogenic	Yes	7.0	0.0
MSP0721	haib17CEM5106_HCY5HCCXY_SL271123.34	63352	no	Yes				prophage/temperate/lysogenic	Yes	22.0	0.0
MSP0722	haib17CEM4890_H2NYMCCXY_SL254796.186	63321	no	Yes				prophage/temperate/lysogenic	Yes	9.0	0.0
MSP0723	haib17CEM4890_HKC32ALXX_SL254706.241	63274	no	No				prophage/temperate/lysogenic	Yes	13.0	0.0
MSP0724	haib17CEM4890_H75CGCCXY_SL263666.60	63248	no	Yes			Acinetobacter baumannii	prophage/temperate/lysogenic	Yes	30.0	1.0
MSP0726	haib17CEM4890_H75CGCCXY_SL263678.75	63171	no	Yes			Acinetobacter baumannii	prophage/temperate/lysogenic	Yes	24.0	1.0
MSP0727	haib17CEM5106_HCV72CCXY_SL269719.70	63169	no	Yes			Klebsiella sp. M5al	prophage/temperate/lysogenic	No	30.0	0.0
MSP0728	haib17CEM5106_HCV72CCXY_SL269744.161	63097	no	No				virulent/lytic	Yes	33.0	0.0
MSP0729	haib17KIU4866_H7HJMCCXY_SL272772.30	63032	yes	No			Pseudomonas balearica DSM 6083	prophage/temperate/lysogenic	Yes	20.0	1.0
MSP0730	haib17CEM4890_HKC32ALXX_SL254681.244	63007	no	Yes				prophage/temperate/lysogenic	Yes	19.0	0.0
MSP0731	haib17CEM4890_H2NYMCCXY_SL254771.211	62914	no	No				prophage/temperate/lysogenic	Yes	16.0	0.0
MSP0732	haib17DB4959_H3MGVCCXY_SL259846.1	62893	no	No			Xanthomonas campestris pv. raphani 756C	virulent/lytic	Yes	25.0	1.0
MSP0734	haib17CEM5241_HMGMHCCXY_SL343483.1	62834	no	Yes			Moraxella osloensis	prophage/temperate/lysogenic	Yes	31.0	1.0
MSP0735	haib17KIU4866_H7HJMCCXY_SL272744.45	62825	yes	No			Pseudomonas balearica DSM 6083	prophage/temperate/lysogenic	Yes	16.0	1.0
MSP0736	haib17CEM4890_H2NYMCCXY_SL254861.36	62823	no	Yes			Kosakonia cowanii JCM 10956 = DSM 18146	prophage/temperate/lysogenic	Yes	30.0	0.0

MSP0737	haib17CEM4890_HKC32ALXX_SL254706.246	62737	no	Yes	Acinetobacter	Acinetobacter_baumannii	Acinetobacter baumannii	prophage/temperate/lysogenic	No	31.0	1.0
MSP0738	haib17CEM5241_HMGMHCCXY_SL343449.4	62706	no	No				virulent/lytic	Yes	21.0	0.0
MSP0739	haib17KIU4866_H7HJMCCXY_SL272755.20	62645	no	Yes				prophage/temperate/lysogenic	Yes	13.0	0.0
MSP0740	haib17CEM4890_H2NYMCCXY_SL254783.283	62621	yes	No			Raoultella planticola	prophage/temperate/lysogenic	Yes	10.0	0.0
MSP0742	haib18CEM5453_HMGTJCCXY_SL342608.26	62609	no	Yes				prophage/temperate/lysogenic	Yes	32.0	0.0
MSP0743	haib17CEM5106_HCY5HCCXY_SL270936.96	62588	no	Yes			Desulfovibrio magneticus RS-1	prophage/temperate/lysogenic	Yes	27.0	0.0
MSP0744	haib17CEM5106_HCV72CCXY_SL269718.98	62584	no	Yes			Enterococcus cecorum	prophage/temperate/lysogenic	No	22.0	0.0
MSP0745	haib17CEM4890_H2NYMCCXY_SL254830.241	62561	no	Yes			Dickeya zeae NCPPB 3531	prophage/temperate/lysogenic	No	26.0	0.0
MSP0746	haib17CEM5106_HCVMTCCXY_SL269598.95	62545	no	No	Arthrobacter	Arthrobacter_globiformis		prophage/temperate/lysogenic	Yes	32.0	0.0
MSP0747	haib18CEM5453_HNHKFCCXY_SL345969.1	62532	no	No	Arthrobacter	None		virulent/lytic	Yes	28.0	0.0
MSP0748	haib17KIU4866_HMCMJCCXY_SL335960.40	62522	no	Yes			Acinetobacter sp. ACNIH1	prophage/temperate/lysogenic	Yes	32.0	0.0
MSP0749	haib17CEM4890_H2NYMCCXY_SL254829.205	62514	no	Yes				prophage/temperate/lysogenic	No	33.0	0.0
MSP0750	haib17DB4959_H3MGVCCXY_SL259963.112	62471	no	Yes			Escherichia coli ED1a	prophage/temperate/lysogenic	Yes	31.0	1.0
MSP0751	haib17CEM4890_H2NYMCCXY_SL254793.122	62463	no	Yes			Enterobacter cloacae	virulent/lytic	No	26.0	0.0
MSP0753	haib17CEM4890_H2NYMCCXY_SL254856.50	62430	no	Yes			Xenorhabdus doucetiae	prophage/temperate/lysogenic	Yes	29.0	0.0
MSP0754	haib17CEM4890_HKC32ALXX_SL254764.222	62398	no	Yes				virulent/lytic	Yes	15.0	0.0
MSP0755	haib17CEM4890_H2NYMCCXY_SL254770.177	62356	no	No				prophage/temperate/lysogenic	Yes	12.0	0.0
MSP0756	haib17KIU4866_H7HJMCCXY_SL272816.88	62323	no	Yes				prophage/temperate/lysogenic	Yes	29.0	0.0
MSP0757	haib17CEM4890_HKC32ALXX_SL254679.27	62285	no	Yes				prophage/temperate/lysogenic	Yes	22.0	0.0
MSP0758	haib17CEM5106_HCY5JCCXY_SL269564.35	62198	no	Yes			Aeribacillus pallidus	prophage/temperate/lysogenic	Yes	31.0	0.0
MSP0759	haib17CEM5106_HCY5JCCXY_SL269535.139	62183	no	Yes	Lactococcus	Lactococcus_lactis	Streptococcus thermophilus	prophage/temperate/lysogenic	No	29.0	0.0
MSP0760	haib18CEM5453_HMCMJCCXY_SL336225.21	62155	no	Yes			Proteus mirabilis	prophage/temperate/lysogenic	Yes	26.0	0.0
MSP0761	haib17CEM5106_HCV72CCXY_SL269759.25	62067	no	Yes				prophage/temperate/lysogenic	Yes	30.0	0.0
MSP0762	haib17CEM5106_HCV72CCXY_SL269751.72	61992	no	Yes				virulent/lytic	Yes	22.0	0.0
MSP0763	haib17CEM5106_HCV72CCXY_SL269784.57	61915	no	Yes				virulent/lytic	Yes	32.0	0.0
MSP0764	haib17KIU4866_H7HJMCCXY_SL272746.36	61908	no	No	Proteus	Proteus_mirabilis	Klebsiella pneumoniae subsp. pneumoniae	virulent/lytic	Yes	32.0	0.0
MSP0765	haib17CEM5106_HCY5HCCXY_SL270914.72	61888	no	Yes				prophage/temperate/lysogenic	Yes	33.0	0.0
MSP0766	haib17CEM4890_H2NYMCCXY_SL254783.290	61868	no	Yes			Citrobacter koseri	prophage/temperate/lysogenic	Yes	26.0	0.0
MSP0767	haib17CEM4890_H2NYMCCXY_SL254776.80	61860	no	No	Bacillus	Bacillus_cereus	Bacillus cereus AH1271	prophage/temperate/lysogenic	Yes	12.0	0.0
MSP0768	haib17CEM5106_HCV72CCXY_SL269744.166	61850	no	Yes				virulent/lytic	Yes	28.0	0.0
MSP0769	haib17CEM4890_HKC32ALXX_SL254750.6	61778	no	No	Ruegeria	Ruegeria_pomeroyi	Paracoccus contaminans	virulent/lytic	Yes	10.0	0.0
MSP0771	haib17CEM4890_H75CGCCXY_SL263654.65	61749	no	Yes			Ruminococcus bicirculans	virulent/lytic	Yes	33.0	0.0
MSP0772	haib17CEM5106_HCVMTCCXY_SL269601.70	61748	no	Yes	Lactococcus	Lactococcus_lactis	Streptococcus thermophilus TH1477	prophage/temperate/lysogenic	No	32.0	0.0
MSP0774	haib17CEM4890_H7KYMCCXY_SL273103.78	61672	no	Yes				prophage/temperate/lysogenic	Yes	11.0	0.0
MSP0775	haib17CEM5106_HCV72CCXY_SL269717.65	61671	no	Yes				prophage/temperate/lysogenic	Yes	24.0	0.0
MSP0776	haib18CEM5453_HMC2KCCXY_SL336638.1	61638	no	No				virulent/lytic	Yes	22.0	0.0
MSP0777	haib18CEM5453_HNGH3CCXY_SL344549.13	61625	no	No				virulent/lytic	Yes	27.0	0.0
MSP0779	haib17CEM5106_HCV72CCXY_SL269762.43	61485	no	Yes				prophage/temperate/lysogenic	Yes	29.0	0.0
MSP0781	haib17CEM4890_H2NYMCCXY_SL254818.236	61447	no	No			Desulfovibrio desulfuricans ND132	prophage/temperate/lysogenic	Yes	14.0	0.0
MSP0782	haib18CEM5453_HMGW3CCXY_SL342337.1	61391	no	No	Mycobacterium	Mycobacterium_smegmatis		prophage/temperate/lysogenic	Yes	33.0	0.0
MSP0783	haib17CEM4890_H2NYMCCXY_SL254783.295	61349	no	Yes				prophage/temperate/lysogenic	Yes	4.0	0.0
MSP0784	haib17CEM4890_H75CGCCXY_SL263652.171	61142	no	Yes			Citrobacter koseri	prophage/temperate/lysogenic	Yes	30.0	0.0
MSP0785	haib17CEM4890_H2NYMCCXY_SL254839.37	61064	no	Yes				prophage/temperate/lysogenic	Yes	33.0	0.0
MSP0786	haib17KIU4866_HMCMJCCXY_SL335947.87	61050	no	Yes			Kosakonia cowanii JCM 10956 = DSM 18146	prophage/temperate/lysogenic	No	27.0	0.0
MSP0787	haib17CEM4890_H75CGCCXY_SL263652.172	60928	no	Yes				prophage/temperate/lysogenic	Yes	31.0	0.0
MSP0788	haib17KIU4866_H7HJMCCXY_SL272826.128	60888	no	Yes	Pseudomonas	None	Pseudomonas stutzeri A1501	prophage/temperate/lysogenic	Yes	32.0	0.0
MSP0789	haib17CEM4890_HKC32ALXX_SL254718.197	60885	no	Yes				prophage/temperate/lysogenic	Yes	14.0	0.0
MSP0790	haib17CEM4890_H2NYMCCXY_SL254829.217	60884	no	No				virulent/lytic	Yes	5.0	0.0
MSP0791	haib17CEM4890_H75CGCCXY_SL263678.76	60871	no	Yes			Leptothrix cholodnii SP-6	prophage/temperate/lysogenic	Yes	29.0	0.0
MSP0792	haib17CEM4890_H2NYMCCXY_SL254854.148	60793	no	Yes			Cellvibrio japonicus Ueda107	prophage/temperate/lysogenic	Yes	29.0	0.0
MSP0793	haib17CEM4890_H2NYMCCXY_SL254805.231	60773	no	Yes	Stenotrophomonas	Stenotrophomonas_maltophilia		prophage/temperate/lysogenic	Yes	31.0	1.0
MSP0794	haib17CEM4890_HMCMJCCXY_SL335794.29	60764	no	Yes			Pseudomonas sp. CMR12a	virulent/lytic	Yes	33.0	0.0
MSP0795	haib17CEM4890_H2NYMCCXY_SL254794.457	60746	no	Yes			Salmonella enterica subsp. enterica serovar Derby	prophage/temperate/lysogenic	Yes	20.0	1.0
MSP0796	haib17CEM4890_H2NYMCCXY_SL254783.307	60683	no	No				prophage/temperate/lysogenic	Yes	22.0	0.0
MSP0797	haib17CEM5106_HCY5JCCXY_SL269598.77	60624	no	Yes				prophage/temperate/lysogenic	Yes	33.0	0.0
MSP0798	haib18CEM5453_HMGW3CCXY_SL342282.1	60560	yes	No				virulent/lytic	Yes	20.0	0.0
MSP0799	haib17CEM5106_HCY5JCCXY_SL269536.75	60422	no	Yes				prophage/temperate/lysogenic	Yes	32.0	0.0
MSP0800	haib17CEM4890_HKC32ALXX_SL254706.261	60383	no	Yes				prophage/temperate/lysogenic	Yes	16.0	0.0
MSP0801	haib17KIU4866_H7HJMCCXY_SL272816.97	60374	no	Yes			Pectobacterium parmentieri	prophage/temperate/lysogenic	Yes	33.0	0.0
MSP0803	haib17CEM4890_H2NYMCCXY_SL254818.241	60332	no	Yes			Pluralibacter gergoviae	virulent/lytic	Yes	24.0	0.0
MSP0804	haib17KIU4866_H7HJMCCXY_SL272814.49	60300	no	Yes				prophage/temperate/lysogenic	Yes	32.0	0.0
MSP0805	haib17CEM4890_H2NYMCCXY_SL254851.20	60294	no	Yes			Kosakonia cowanii JCM 10956 = DSM 18146	prophage/temperate/lysogenic	Yes	28.0	0.0
MSP0806	haib17KIU4866_HMCMJCCXY_SL335963.50	60290	no	No			Acinetobacter junii	prophage/temperate/lysogenic	Yes	24.0	1.0
MSP0807	haib18CEM5453_HNHKFCCXY_SL346109.13	60275	no	No			Salmonella enterica subsp. enterica serovar Waycross	virulent/lytic	Yes	13.0	1.0
MSP0808	haib18CEM5453_HMC2KCCXY_SL336644.22	60248	no	No				virulent/lytic	Yes	30.0	0.0
MSP0809	haib17CEM4890_HKC32ALXX_SL254752.114	60232	no	Yes	Pseudomonas	Pseudomonas_aeruginosa	Pseudomonas balearica DSM 6083	prophage/temperate/lysogenic	Yes	29.0	0.0
MSP0810	haib17CEM4890_H75CGCCXY_SL263714.80	60205	no	Yes				prophage/temperate/lysogenic	Yes	26.0	0.0
MSP0812	haib17CEM4890_H2NYMCCXY_SL254829.222	60168	no	Yes			Acinetobacter bereziniae	virulent/lytic	Yes	23.0	0.0

MSP0813	haib17CEM4890_HKC32ALXX_SL254681.253	60132	no	Yes				prophage/temperate/lysogenic	Yes	32.0	0.0
MSP0814	haib18CEM5453_HNHKFCCXY_SL346109.14	60079	yes	No				prophage/temperate/lysogenic	Yes	17.0	0.0
MSP0815	haib17CEM5106_HCY5HCCXY_SL270908.30	60005	no	Yes				prophage/temperate/lysogenic	Yes	21.0	0.0
MSP0817	haib17CEM4890_HMCMJCCXY_SL335774.26	59997	no	Yes			Streptomyces sp. SM17	prophage/temperate/lysogenic	Yes	31.0	0.0
MSP0818	haib17CEM4890_H2NYMCCXY_SL254796.202	59995	no	Yes			Serratia sp. FGI94	prophage/temperate/lysogenic	Yes	25.0	0.0
MSP0819	haib17KIU4866_H7HJMCCXY_SL272748.18	59936	no	No				virulent/lytic	Yes	21.0	0.0
MSP0820	haib17CEM5106_HCCGHCCXY_SL270477.1	59922	no	No				virulent/lytic	No	16.0	0.0
MSP0821	haib18CEM5453_HMGW3CCXY_SL342278.5	59857	no	Yes			Pseudomonas sp. S-6-2	prophage/temperate/lysogenic	Yes	21.0	0.0
MSP0823	haib17KIU4866_HMCMJCCXY_SL335963.52	59797	no	No			Ruminococcus bicirculans	prophage/temperate/lysogenic	Yes	32.0	0.0
MSP0824	haib17CEM5106_HCV72CCXY_SL269788.211	59787	no	Yes			Corynebacterium striatum	prophage/temperate/lysogenic	Yes	26.0	0.0
MSP0825	haib17KIU4866_HMCMJCCXY_SL335935.27	59733	yes	No				virulent/lytic	Yes	18.0	0.0
MSP0828	haib17CEM5106_HCV72CCXY_SL269792.142	59661	no	Yes				prophage/temperate/lysogenic	Yes	26.0	0.0
MSP0827	haib18CEM5453_HNHKFCCXY_SL345480.1	59661	no	No	Gordonia	Gordonia_terrae		virulent/lytic	No	32.0	0.0
MSP0829	haib18CEM5453_HT5YVCCXY_SL357341.1	59655	no	No				virulent/lytic	Yes	2.0	0.0
MSP0831	haib17CEM4890_H2NYMCCXY_SL254860.33	59577	no	Yes				prophage/temperate/lysogenic	Yes	31.0	0.0
MSP0832	haib18CEM5453_HNHKFCCXY_SL345930.4	59568	no	No				prophage/temperate/lysogenic	Yes	21.0	0.0
MSP0833	haib17CEM4890_H2NYMCCXY_SL254807.171	59558	no	Yes				prophage/temperate/lysogenic	Yes	11.0	0.0
MSP0834	haib17KIU4866_H7HJMCCXY_SL272809.21	59544	no	Yes				prophage/temperate/lysogenic	Yes	31.0	0.0
MSP0836	haib17CEM4890_HKC32ALXX_SL254718.204	59494	yes	No				prophage/temperate/lysogenic	Yes	11.0	0.0
MSP0835	haib17KIU4866_HMCMJCCXY_SL335949.89	59494	no	Yes				prophage/temperate/lysogenic	Yes	31.0	0.0
MSP0837	haib17CEM5106_HCY5JCCXY_SL269538.191	59479	no	Yes	Propionibacterium	Propionibacterium_freudenreichii		prophage/temperate/lysogenic	Yes	32.0	0.0
MSP0838	haib17DB4959_H3MGVCCXY_SL259990.14	59443	no	Yes			Enterococcus faecalis DENG1	prophage/temperate/lysogenic	No	25.0	1.0
MSP0839	haib17CEM4890_H2NYMCCXY_SL254843.364	59406	no	No				prophage/temperate/lysogenic	Yes	12.0	0.0
MSP0840	haib17CEM4890_HKC32ALXX_SL254742.249	59365	yes	No	Bacillus	Bacillus_cereus		prophage/temperate/lysogenic	Yes	10.0	0.0
MSP0841	haib17CEM4890_H75CGCCXY_SL263689.92	59361	no	Yes				prophage/temperate/lysogenic	Yes	28.0	0.0
MSP0842	haib17CEM5241_HMGMHCCXY_SL343449.5	59347	no	No				virulent/lytic	Yes	15.0	0.0
MSP0843	haib17CEM4890_HKC32ALXX_SL254763.83	59343	no	Yes	Bacillus	Bacillus_cereus		prophage/temperate/lysogenic	Yes	32.0	0.0
MSP0844	haib17CEM4890_HKC32ALXX_SL254728.266	59266	no	No				prophage/temperate/lysogenic	Yes	16.0	0.0
MSP0845	haib18CEM5453_HNHKFCCXY_SL345508.3	59217	yes	No				virulent/lytic	Yes	21.0	0.0
MSP0846	haib18CEM5453_HT5YVCCXY_SL357401.1	59104	no	No				virulent/lytic	Yes	4.0	0.0
MSP0847	haib17DB4959_H3MGVCCXY_SL259857.1	58950	no	No				virulent/lytic	Yes	16.0	0.0
MSP0848	haib17CEM4890_HKC32ALXX_SL254762.37	58862	yes	No			Vitreoscilla filiformis	virulent/lytic	Yes	11.0	0.0
MSP0850	haib17CEM5106_HCY5JCCXY_SL269526.68	58843	no	Yes				prophage/temperate/lysogenic	Yes	33.0	0.0
MSP0851	haib17DB4959_H3MGVCCXY_SL259923.38	58796	no	No				virulent/lytic	Yes	22.0	0.0
MSP0852	haib17CEM5106_HCY5HCCXY_SL270915.109	58793	no	Yes				prophage/temperate/lysogenic	Yes	29.0	0.0
MSP0853	haib17CEM4890_H2NYMCCXY_SL254806.260	58682	no	Yes				prophage/temperate/lysogenic	Yes	18.0	0.0
MSP0854	haib17CEM5106_HCVMTCCXY_SL269598.104	58674	no	Yes				virulent/lytic	Yes	14.0	0.0
MSP0855	haib17CEM5106_HCVMTCCXY_SL269582.57	58609	no	Yes	Propionibacterium	Propionibacterium_freudenreichii		prophage/temperate/lysogenic	Yes	33.0	0.0
MSP0856	haib17CEM4890_H7KYMCCXY_SL273087.4	58565	yes	No	Microbacterium	Microbacterium_foliorum		virulent/lytic	Yes	10.0	0.0
MSP0858	haib17CEM4890_H2NYMCCXY_SL254809.10	58520	no	No				virulent/lytic	Yes	21.0	0.0
MSP0859	haib17CEM5106_HCY5JCCXY_SL269561.83	58474	no	Yes			Ruminococcus bicirculans	virulent/lytic	No	31.0	0.0
MSP0860	haib17CEM4890_H75CGCCXY_SL263689.93	58450	no	Yes				prophage/temperate/lysogenic	Yes	26.0	0.0
MSP0861	haib17CEM4890_H2NYMCCXY_SL254772.302	58449	no	No				prophage/temperate/lysogenic	Yes	8.0	0.0
MSP0862	haib17CEM5106_HCY5HCCXY_SL270942.84	58445	no	Yes			Pseudomonas sp. CMR12a	prophage/temperate/lysogenic	Yes	33.0	0.0
MSP0863	haib17CEM5106_HCY5JCCXY_SL269575.64	58390	no	No				prophage/temperate/lysogenic	Yes	3.0	0.0
MSP0865	haib17CEM4890_H75CGCCXY_SL263714.83	58298	no	Yes	Pseudomonas	Pseudomonas_aeruginosa	Pseudomonas stutzeri A1501	prophage/temperate/lysogenic	Yes	26.0	0.0
MSP0867	haib17CEM4890_HKC32ALXX_SL254680.208	58182	yes	No			Bacillus thermoamylovorans	prophage/temperate/lysogenic	Yes	8.0	0.0
MSP0866	haib17CEM4890_HKC32ALXX_SL254697.1	58182	no	No				prophage/temperate/lysogenic	Yes	8.0	0.0
MSP0868	haib17CEM4890_H2NYMCCXY_SL254807.178	58173	no	No				prophage/temperate/lysogenic	Yes	14.0	0.0
MSP0869	haib18CEM5453_HMGW3CCXY_SL342603.1	58168	no	No			Pseudomonas parafulva	prophage/temperate/lysogenic	Yes	29.0	0.0
MSP0870	haib17CEM4890_H2NYMCCXY_SL254772.303	58158	no	Yes				prophage/temperate/lysogenic	Yes	11.0	0.0
MSP0871	haib17CEM4890_H75CGCCXY_SL263688.152	58105	no	No			Ruminococcus bicirculans	virulent/lytic	No	33.0	0.0
MSP0872	haib17CEM4890_HKC32ALXX_SL254762.38	58076	no	No				virulent/lytic	Yes	13.0	0.0
MSP0873	haib17CEM5106_HCV72CCXY_SL269762.45	58046	no	Yes			Cronobacter sakazakii ES15	virulent/lytic	No	31.0	0.0
MSP0874	haib17CEM4890_H2NYMCCXY_SL254808.32	58037	no	Yes			Mixta calida	virulent/lytic	Yes	17.0	0.0
MSP0875	haib17CEM5106_HCV72CCXY_SL269786.231	58036	no	Yes	Pseudomonas	Pseudomonas_fluorescens	Pseudomonas aeruginosa 19BR	prophage/temperate/lysogenic	Yes	28.0	0.0
MSP0876	haib17CEM4890_HKC32ALXX_SL254675.2	58014	no	No	Gordonia	Gordonia_terrae		prophage/temperate/lysogenic	Yes	13.0	0.0
MSP0877	haib17KIU4866_HMCMJCCXY_SL335912.1	57966	no	No			Pseudomonas sp. MRSN12121	virulent/lytic	Yes	22.0	0.0
MSP0878	haib17CEM5106_HCV72CCXY_SL269753.162	57965	no	Yes				prophage/temperate/lysogenic	Yes	33.0	0.0
MSP0880	haib17KIU4866_H7HJMCCXY_SL272797.3	57954	no	No				virulent/lytic	Yes	13.0	0.0
MSP0881	haib17CEM5106_HCV72CCXY_SL269786.233	57952	no	Yes			Pseudomonas aeruginosa 19BR	prophage/temperate/lysogenic	Yes	31.0	1.0
MSP0882	haib17CEM4890_H2NYMCCXY_SL254769.221	57921	no	Yes				prophage/temperate/lysogenic	Yes	24.0	0.0
MSP0883	haib17CEM4890_H2NYMCCXY_SL254790.79	57909	no	No				prophage/temperate/lysogenic	Yes	18.0	0.0
MSP0884	haib17CEM4890_H7KYMCCXY_SL273060.41	57825	no	Yes			Haemophilus sp. oral taxon 036	prophage/temperate/lysogenic	No	27.0	0.0
MSP0885	haib17CEM5106_HCY5JCCXY_SL269563.21	57777	no	Yes				prophage/temperate/lysogenic	Yes	31.0	0.0
MSP0886	haib17CEM4890_HKC32ALXX_SL254728.279	57764	no	Yes	Clostridium	None	Clostridium botulinum	prophage/temperate/lysogenic	Yes	12.0	0.0
MSP0887	haib18CEM5453_HMC2KCCXY_SL336773.20	57759	no	No				prophage/temperate/lysogenic	Yes	18.0	0.0
MSP0888	haib17CEM4890_HKC32ALXX_SL254765.155	57649	no	No				prophage/temperate/lysogenic	Yes	15.0	0.0

MSP0889	haib17CEM4890_HKC32ALXX_SL254706.273	57627	no	Yes			Citrobacter koseri	virulent/lytic	Yes	24.0	0.0
MSP0890	haib17CEM4890_HKC32ALXX_SL254680.209	57606	no	Yes			Aquiflexum balticum DSM 16537	prophage/temperate/lysogenic	Yes	33.0	0.0
MSP0891	haib17KIU4866_HMCMJCCXY_SL335938.84	57602	no	Yes			Pseudomonas balearica DSM 6083	prophage/temperate/lysogenic	No	30.0	1.0
MSP0892	haib17CEM4890_H2NYMCCXY_SL254793.134	57597	yes	No			Xanthomonas campestris pv. raphani	virulent/lytic	Yes	17.0	1.0
MSP0893	haib17CEM4890_H75CGCCXY_SL263703.1	57586	yes	No				prophage/temperate/lysogenic	Yes	7.0	0.0
MSP0894	haib17CEM4890_HKC32ALXX_SL254728.280	57583	yes	No	Bacillus	Bacillus_cereus	Bacillus thuringiensis	prophage/temperate/lysogenic	Yes	11.0	0.0
MSP0896	haib18CEM5453_HMGW3CCXY_SL342349.1	57533	no	No				virulent/lytic	No	16.0	0.0
MSP0898	haib17CEM5106_HCY5HCCXY_SL270977.117	57484	no	No				prophage/temperate/lysogenic	Yes	14.0	0.0
MSP0900	haib17CEM5106_HCV72CCXY_SL269728.124	57459	no	Yes				prophage/temperate/lysogenic	Yes	25.0	0.0
MSP0899	haib18CEM5453_HMGN5CCXY_SL344653.2	57459	yes	No				prophage/temperate/lysogenic	Yes	5.0	0.0
MSP0901	haib17CEM4890_H2NYMCCXY_SL254794.475	57380	no	Yes			Klebsiella pneumoniae	prophage/temperate/lysogenic	No	31.0	1.0
MSP0902	haib17KIU4866_H7HJMCCXY_SL272827.85	57346	no	Yes			Cedecea neteri	virulent/lytic	Yes	29.0	0.0
MSP0903	haib17CEM5106_HCY5JCCXY_SL269559.88	57330	no	Yes				prophage/temperate/lysogenic	Yes	29.0	0.0
MSP0904	haib17CEM4890_H2NYMCCXY_SL254784.392	57305	no	Yes				prophage/temperate/lysogenic	Yes	13.0	0.0
MSP0906	haib17CEM4890_H2NYMCCXY_SL254831.276	57214	no	Yes				prophage/temperate/lysogenic	Yes	15.0	0.0
MSP0907	haib17CEM5106_HCY5JCCXY_SL269565.38	57125	no	Yes				prophage/temperate/lysogenic	Yes	32.0	0.0
MSP0908	haib17CEM4890_HKC32ALXX_SL254705.246	57073	no	Yes			Pectobacterium versatile	prophage/temperate/lysogenic	Yes	23.0	0.0
MSP0909	haib17CEM4890_H2NYMCCXY_SL254793.138	57063	no	Yes			Clostridium kluyveri NBRC 12016	prophage/temperate/lysogenic	Yes	31.0	0.0
MSP0910	haib17CEM5106_HCY5JCCXY_SL269546.94	57058	no	Yes			Leclercia adecarboxylata	prophage/temperate/lysogenic	Yes	33.0	0.0
MSP0911	haib17CEM4890_H2NYMCCXY_SL254819.253	57029	no	Yes				prophage/temperate/lysogenic	Yes	16.0	0.0
MSP0912	haib17CEM4890_HKC32ALXX_SL254742.261	57018	no	No				prophage/temperate/lysogenic	Yes	8.0	0.0
MSP0913	haib17CEM4890_H2NYMCCXY_SL254848.53	56835	no	No	Bacillus	Bacillus_cereus	Bacillus cereus AH1271	prophage/temperate/lysogenic	Yes	12.0	0.0
MSP0914	haib17CEM5106_HCY5JCCXY_SL269538.205	56806	no	Yes	Arthrobacter	Arthrobacter_globiformis		virulent/lytic	Yes	31.0	0.0
MSP0915	haib17CEM5106_HCV72CCXY_SL269731.104	56770	no	Yes	Acinetobacter	Acinetobacter_baumannii	Ruminococcus bicirculans	virulent/lytic	Yes	31.0	0.0
MSP0916	haib17CEM4890_H2NYMCCXY_SL254772.316	56759	no	No	Faecalibacterium	Faecalibacterium_prausnitzii		prophage/temperate/lysogenic	Yes	10.0	0.0
MSP0917	haib17CEM4890_H75CGCCXY_SL263664.71	56750	no	Yes			Xanthomonas albilineans GPE PC73	prophage/temperate/lysogenic	Yes	24.0	0.0
MSP0918	haib17CEM4890_H2NYMCCXY_SL254814.85	56718	no	Yes				virulent/lytic	Yes	23.0	0.0
MSP0919	haib17CEM5106_HCY5JCCXY_SL269569.56	56656	no	Yes				prophage/temperate/lysogenic	Yes	23.0	0.0
MSP0920	haib17CEM4890_H2NYMCCXY_SL254830.275	56561	no	Yes			Serratia plymuthica AS9	prophage/temperate/lysogenic	Yes	23.0	0.0
MSP0921	haib17CEM4890_H2NYMCCXY_SL254783.341	56548	no	Yes			Kosakonia cowanii JCM 10956 = DSM 18146	virulent/lytic	No	23.0	0.0
MSP0922	haib17KIU4866_H7HJMCCXY_SL272741.34	56537	no	Yes			Kosakonia cowanii JCM 10956 = DSM 18146	prophage/temperate/lysogenic	Yes	27.0	0.0
MSP0923	haib17CEM5106_HCV72CCXY_SL269784.70	56536	no	Yes				virulent/lytic	Yes	31.0	0.0
MSP0924	haib17CEM5106_HCY5JCCXY_SL269557.70	56529	no	No			Geminocystis herdmanii PCC 6308	prophage/temperate/lysogenic	Yes	33.0	0.0
MSP0925	haib17DB4959_H3MGVCCXY_SL259882.44	56480	yes	No	Xylella	Xylella_fastidiosa		virulent/lytic	Yes	13.0	0.0
MSP0926	haib17CEM4890_HKC32ALXX_SL254685.2	56452	no	No				virulent/lytic	Yes	21.0	0.0
MSP0927	haib17CEM4890_HKC32ALXX_SL254763.87	56428	no	No				prophage/temperate/lysogenic	Yes	14.0	0.0
MSP0928	haib17CEM5106_HCVMTCCXY_SL269600.83	56380	no	Yes				prophage/temperate/lysogenic	Yes	25.0	0.0
MSP0929	haib17KIU4866_H7HJMCCXY_SL272804.55	56373	no	Yes			Pectobacterium odoriferum	prophage/temperate/lysogenic	No	31.0	0.0
MSP0930	haib17CEM4890_H2NYMCCXY_SL254843.393	56318	no	Yes				prophage/temperate/lysogenic	Yes	15.0	0.0
MSP0931	haib17KIU4866_H7HJMCCXY_SL272823.42	56298	no	Yes			Pseudomonas aeruginosa	virulent/lytic	Yes	28.0	1.0
MSP0934	haib17KIU4866_H7HJMCCXY_SL272786.102	56160	no	Yes	Pseudomonas	None	Pseudomonas aeruginosa	prophage/temperate/lysogenic	Yes	30.0	0.0
MSP0935	haib17CEM4890_HKC32ALXX_SL254680.221	56156	no	Yes			Kosakonia cowanii JCM 10956 = DSM 18146	prophage/temperate/lysogenic	No	33.0	0.0
MSP0936	haib18CEM5453_HT5YVCCXY_SL357297.2	56104	yes	No	Xylella	Xylella_fastidiosa		virulent/lytic	No	22.0	0.0
MSP0938	haib17CEM4890_H2NYMCCXY_SL254855.235	56036	no	Yes				prophage/temperate/lysogenic	Yes	13.0	0.0
MSP0940	haib17CEM4890_H2NYMCCXY_SL254784.411	56006	no	Yes				prophage/temperate/lysogenic	Yes	18.0	0.0
MSP0939	haib17CEM5106_HCY5HCCXY_SL270989.88	56006	no	Yes			Corynebacterium jeikeium	prophage/temperate/lysogenic	Yes	31.0	0.0
MSP0941	haib17CEM4890_H2NYMCCXY_SL254807.184	56002	no	No			Olsenella umbonata	prophage/temperate/lysogenic	No	15.0	0.0
MSP0942	haib17CEM4890_H75CGCCXY_SL263640.136	55980	no	Yes				prophage/temperate/lysogenic	Yes	9.0	0.0
MSP0944	haib17CEM4890_H2NYMCCXY_SL254814.88	55933	no	Yes	Cronobacter	None	Kosakonia cowanii JCM 10956 = DSM 18146	prophage/temperate/lysogenic	Yes	24.0	0.0
MSP0945	haib17CEM4890_HKC32ALXX_SL254692.122	55883	no	No				virulent/lytic	Yes	17.0	0.0
MSP0946	haib17CEM5106_HCV72CCXY_SL269773.134	55831	no	Yes			Pseudomonas sp. CMR12a	prophage/temperate/lysogenic	Yes	28.0	0.0
MSP0947	haib17KIU4866_H7HJMCCXY_SL272826.153	55829	no	No				prophage/temperate/lysogenic	Yes	13.0	0.0
MSP0948	haib17CEM4890_H7KYMCCXY_SL273082.1	55806	no	No			Klebsiella pneumoniae subsp. pneumoniae	virulent/lytic	Yes	19.0	1.0
MSP0949	haib17KIU4866_H7HJMCCXY_SL272787.20	55803	no	Yes			Acinetobacter sp. ACNIH1	prophage/temperate/lysogenic	Yes	31.0	0.0
MSP0950	haib17CEM4890_H75CGCCXY_SL263677.75	55792	no	Yes				prophage/temperate/lysogenic	Yes	25.0	0.0
MSP0951	haib17CEM4890_H75CGCCXY_SL263689.101	55772	no	No			Klebsiella pneumoniae subsp. pneumoniae	prophage/temperate/lysogenic	Yes	16.0	1.0
MSP0952	haib17CEM4890_HKC32ALXX_SL254764.246	55764	no	Yes				virulent/lytic	Yes	7.0	0.0
MSP0953	haib17CEM4890_H3KHWCCXY_SL263364.1	55762	no	No			Pectobacterium parmentieri WPP163	virulent/lytic	Yes	16.0	0.0
MSP0954	haib17KIU4866_H7HJMCCXY_SL272776.114	55761	no	No				virulent/lytic	Yes	12.0	0.0
MSP0955	haib17CEM4890_H75CGCCXY_SL263690.50	55717	no	Yes			Pectobacterium versatile	prophage/temperate/lysogenic	Yes	33.0	0.0
MSP0956	haib17DB4959_HMCMJCCXY_SL336172.14	55692	no	No				prophage/temperate/lysogenic	Yes	32.0	0.0
MSP0957	haib17KIU4866_HMCMJCCXY_SL335936.58	55661	no	No				virulent/lytic	Yes	14.0	0.0
MSP0959	haib17CEM4890_H75CGCCXY_SL263652.198	55648	no	No				virulent/lytic	Yes	25.0	0.0
MSP0958	haib17CEM4890_H7KYMCCXY_SL273105.1	55648	no	Yes			Klebsiella sp. PO552	virulent/lytic	Yes	23.0	0.0
MSP0960	haib17CEM4890_H2NYMCCXY_SL254780.293	55637	no	Yes			Desulfovibrio desulfuricans ND132	prophage/temperate/lysogenic	Yes	32.0	0.0
MSP0961	haib17CEM4890_H75CGCCXY_SL263702.30	55538	no	Yes				prophage/temperate/lysogenic	Yes	29.0	0.0
MSP0962	haib17KIU4866_HMCMJCCXY_SL335949.96	55521	no	Yes				virulent/lytic	Yes	32.0	0.0
MSP0963	haib17DB4959_H3MGVCCXY_SL259921.2	55473	no	No			Pseudomonas alcaliphila JAB1	prophage/temperate/lysogenic	Yes	24.0	0.0

MSP0965	haib17CEM5106_HCY5HCCXY_SL271138.82	55394	no	Yes				virulent/lytic	Yes	31.0	0.0
MSP0967	haib17KIU4866_H7HJMCCXY_SL272792.15	55332	no	No			Enterobacter cloacae subsp. cloacae	virulent/lytic	Yes	31.0	0.0
MSP0968	haib17CEM4890_HKC32ALXX_SL254696.5	55280	no	No				virulent/lytic	Yes	13.0	0.0
MSP0969	haib18CEM5453_HMC2KCCXY_SL336650.19	55207	no	No				prophage/temperate/lysogenic	Yes	25.0	0.0
MSP0970	haib17CEM5106_HCY5HCCXY_SL270937.79	55192	no	No				prophage/temperate/lysogenic	Yes	8.0	0.0
MSP0971	haib17CEM4890_H2NYMCCXY_SL254783.356	55120	no	Yes				virulent/lytic	Yes	9.0	0.0
MSP0972	haib17CEM5106_HCV72CCXY_SL269766.49	55081	yes	Yes				prophage/temperate/lysogenic	Yes	32.0	0.0
MSP0973	haib17CEM5106_HCVMTCCXY_SL269535.121	55066	no	Yes			Psychrobacter sp. P11F6	virulent/lytic	Yes	27.0	0.0
MSP0974	haib17CEM4890_H2NYMCCXY_SL254844.27	54996	no	No	Arthrobacter	Arthrobacter_globiformis		virulent/lytic	Yes	27.0	0.0
MSP0975	haib17CEM4890_H7KYMCCXY_SL273090.42	54986	no	Yes			Xanthomonas campestris pv. raphani 756C	prophage/temperate/lysogenic	Yes	31.0	1.0
MSP0976	haib17DB4959_H3MGVCCXY_SL259929.89	54970	no	Yes			Enterobacter kobei	prophage/temperate/lysogenic	No	33.0	0.0
MSP0977	haib17CEM4890_H75CGCCXY_SL263649.92	54939	yes	No			Listeria monocytogenes Finland 1998	virulent/lytic	Yes	9.0	1.0
MSP0978	haib17KIU4866_H7HJMCCXY_SL272759.51	54912	no	No				virulent/lytic	Yes	11.0	0.0
MSP0980	haib17CEM4890_HKC32ALXX_SL254758.4	54870	no	No				virulent/lytic	Yes	23.0	0.0
MSP0981	haib17CEM5106_HCV72CCXY_SL269761.86	54837	no	Yes	Lactobacillus	Lactobacillus_casei		prophage/temperate/lysogenic	Yes	24.0	0.0
MSP0982	haib17CEM5106_HCV72CCXY_SL269722.30	54693	no	Yes			Pseudomonas pseudoalcaligenes CECT 5344	virulent/lytic	Yes	33.0	0.0
MSP0983	haib17CEM4890_H2NYMCCXY_SL254853.103	54662	no	Yes				prophage/temperate/lysogenic	Yes	17.0	0.0
MSP0985	haib17KIU4866_H7HJMCCXY_SL272748.25	54581	no	Yes			Serratia plymuthica AS9	virulent/lytic	Yes	31.0	0.0
MSP0986	haib17CEM4890_H2NYMCCXY_SL254806.283	54562	yes	No				prophage/temperate/lysogenic	Yes	8.0	0.0
MSP0987	haib17KIU4866_H7HJMCCXY_SL272796.160	54507	no	No			Klebsiella pneumoniae	prophage/temperate/lysogenic	Yes	28.0	1.0
MSP0988	haib17CEM4890_H2NYMCCXY_SL254784.430	54476	no	Yes				virulent/lytic	Yes	31.0	0.0
MSP0989	haib17CEM4890_HKC32ALXX_SL254742.283	54444	no	No				virulent/lytic	Yes	15.0	0.0
MSP0990	haib17KIU4866_H7HJMCCXY_SL272768.62	54424	no	Yes			Halomonas ventosae	prophage/temperate/lysogenic	Yes	29.0	0.0
MSP0991	haib17CEM5106_HCY5JCCXY_SL269566.55	54286	no	Yes				virulent/lytic	Yes	30.0	0.0
MSP0992	haib17CEM4890_H2NYMCCXY_SL254794.501	54261	no	Yes				prophage/temperate/lysogenic	Yes	12.0	0.0
MSP0993	haib18CEM5453_HMC2KCCXY_SL336638.2	54231	no	No				virulent/lytic	Yes	32.0	0.0
MSP0994	haib17CEM4890_H2NYMCCXY_SL254794.503	54207	no	No			Klebsiella michiganensis	prophage/temperate/lysogenic	Yes	28.0	0.0
MSP0995	haib17CEM4890_H2NYMCCXY_SL254826.144	54191	no	Yes			Cronobacter muytjensii ATCC 51329	virulent/lytic	Yes	30.0	0.0
MSP0997	haib17CEM4890_H75CGCCXY_SL263677.82	54135	no	Yes	Xanthomonas	None		virulent/lytic	Yes	31.0	0.0
MSP0998	haib17CEM4890_H2NYMCCXY_SL254819.270	54115	no	Yes				prophage/temperate/lysogenic	Yes	11.0	0.0
MSP0999	haib18CEM5453_HMC2KCCXY_SL336679.4	54109	no	No				virulent/lytic	Yes	33.0	0.0
MSP1000	haib17CEM5106_HCY5HCCXY_SL271097.52	53982	no	No			Clostridioides difficile	prophage/temperate/lysogenic	Yes	4.0	0.0
MSP1001	haib17KIU4866_H7HJMCCXY_SL272823.46	53969	no	Yes			Acinetobacter sp. ACNIH1	prophage/temperate/lysogenic	Yes	28.0	0.0
MSP1003	haib17CEM4890_H2NYMCCXY_SL254790.86	53945	yes	No			Enterobacter sp. 638	virulent/lytic	Yes	17.0	0.0
MSP1004	haib17CEM5106_HCY5JCCXY_SL269564.53	53902	no	Yes				virulent/lytic	Yes	13.0	0.0
MSP1006	haib17CEM5106_HCY5JCCXY_SL269564.54	53787	no	Yes			Clostridium botulinum A str. ATCC 3502	prophage/temperate/lysogenic	Yes	32.0	0.0
MSP1007	haib17DB4959_HMCMJCCXY_SL336180.3	53773	no	No	Streptomyces	None		virulent/lytic	Yes	12.0	0.0
MSP1008	haib17CEM5106_HCY5HCCXY_SL270984.80	53750	no	Yes				prophage/temperate/lysogenic	Yes	32.0	0.0
MSP1010	haib17CEM4890_HMCMJCCXY_SL335857.27	53717	no	Yes				prophage/temperate/lysogenic	Yes	32.0	0.0
MSP1009	haib17KIU4866_HMCMJCCXY_SL335938.91	53717	no	Yes			Atlantibacter hermannii	prophage/temperate/lysogenic	Yes	33.0	0.0
MSP1011	haib17CEM4890_H2NYMCCXY_SL254830.295	53668	no	Yes			Enterobacter cloacae subsp. cloacae	prophage/temperate/lysogenic	No	33.0	0.0
MSP1012	haib17CEM4890_HKC32ALXX_SL254762.45	53656	no	Yes				prophage/temperate/lysogenic	Yes	30.0	0.0
MSP1013	haib17CEM4890_H75CGCCXY_SL263700.123	53645	no	Yes				prophage/temperate/lysogenic	Yes	28.0	0.0
MSP1014	haib17CEM4890_H2NYMCCXY_SL254783.372	53583	no	No				prophage/temperate/lysogenic	Yes	4.0	0.0
MSP1015	haib17CEM4890_H2NYMCCXY_SL254781.418	53581	no	Yes			Brevibacillus sp. SCSIO 07484	prophage/temperate/lysogenic	Yes	10.0	0.0
MSP1016	haib17CEM4890_H7KYMCCXY_SL273087.6	53544	no	No			Pragia fontium	prophage/temperate/lysogenic	Yes	13.0	0.0
MSP1017	haib17CEM4890_H75CGCCXY_SL263690.56	53541	no	No			Pseudomonas stutzeri A1501	prophage/temperate/lysogenic	Yes	22.0	1.0
MSP1018	haib17CEM4890_H2NYMCCXY_SL254788.62	53499	no	No				prophage/temperate/lysogenic	Yes	32.0	0.0
MSP1019	haib17CEM5106_HCY5JCCXY_SL269590.34	53452	no	Yes			Dickeya zeae	prophage/temperate/lysogenic	Yes	26.0	0.0
MSP1020	haib17KIU4866_H7HJMCCXY_SL272800.149	53431	no	Yes				virulent/lytic	No	14.0	0.0
MSP1021	haib17KIU4866_H7HJMCCXY_SL272792.17	53374	no	No			Klebsiella pneumoniae	prophage/temperate/lysogenic	Yes	20.0	1.0
MSP1022	haib17CEM5106_HCV72CCXY_SL269776.117	53350	no	Yes	Propionibacterium	Propionibacterium_freudenreichii		virulent/lytic	Yes	26.0	0.0
MSP1023	haib17CEM4890_H2NYMCCXY_SL254858.21	53291	yes	No				virulent/lytic	Yes	26.0	0.0
MSP1025	haib17CEM4890_H75CGCCXY_SL263637.55	53276	no	No				prophage/temperate/lysogenic	Yes	13.0	0.0
MSP1024	haib17CEM5106_HCCGHCCXY_SL270315.158	53276	no	Yes	Pseudomonas	Pseudomonas_aeruginosa	Pseudomonas aeruginosa	prophage/temperate/lysogenic	No	31.0	1.0
MSP1026	haib17CEM4890_H2NYMCCXY_SL254782.231	53271	no	No				prophage/temperate/lysogenic	Yes	33.0	0.0
MSP1027	haib17CEM5106_HCY5HCCXY_SL270953.128	53267	no	Yes	Arthrobacter	Arthrobacter_globiformis		prophage/temperate/lysogenic	Yes	31.0	0.0
MSP1028	haib18CEM5453_HNHKFCCXY_SL346109.20	53255	yes	No			Thermobacillus composti KWC4	virulent/lytic	Yes	15.0	0.0
MSP1029	haib17CEM5106_HCY5HCCXY_SL270961.98	53238	no	No				virulent/lytic	Yes	28.0	0.0
MSP1031	haib17CEM5106_HCV72CCXY_SL269718.116	53230	no	Yes				prophage/temperate/lysogenic	Yes	23.0	0.0
MSP1032	haib17CEM5106_HCY5HCCXY_SL270967.116	53184	no	Yes				virulent/lytic	Yes	16.0	0.0
MSP1033	haib17CEM5106_HCY5HCCXY_SL270953.129	53140	no	Yes			Psychrobacter sp. P11F6	virulent/lytic	Yes	24.0	0.0
MSP1034	haib18CEM5453_HMC2KCCXY_SL336808.1	53121	no	No			Neisseria mucosa	virulent/lytic	No	31.0	0.0
MSP1035	haib17CEM4890_HKC32ALXX_SL254749.3	53085	no	No			Corynebacterium renale	prophage/temperate/lysogenic	No	24.0	0.0
MSP1036	haib17CEM4890_H2NYMCCXY_SL254843.434	53010	no	Yes				virulent/lytic	Yes	9.0	0.0
MSP1037	haib17CEM4890_H2NYMCCXY_SL254825.15	52989	yes	No	Arthrobacter	Arthrobacter_globiformis		virulent/lytic	Yes	28.0	0.0
MSP1038	haib17CEM4890_HMCMJCCXY_SL335844.96	52976	no	Yes			Paracoccus yeei	prophage/temperate/lysogenic	Yes	29.0	0.0
MSP1039	haib17CEM5106_HCY5HCCXY_SL270976.184	52965	no	Yes				prophage/temperate/lysogenic	Yes	13.0	0.0

MSP1041	haib17CEM4890_H2NYMCCXY_SL254793.159	52810	no	No				prophage/temperate/lysogenic	Yes	16.0	0.0
MSP1042	haib17CEM4890_H2NYMCCXY_SL254794.516	52797	no	Yes			Dickeya sp. CSL RW240	prophage/temperate/lysogenic	Yes	22.0	0.0
MSP1043	haib17CEM4890_H2NYMCCXY_SL254780.309	52762	no	Yes			Bradymonas sediminis	prophage/temperate/lysogenic	Yes	27.0	0.0
MSP1044	haib17KIU4866_H7HJMCCXY_SL272787.24	52751	yes	No			Acinetobacter sp. LoGeW2-3	prophage/temperate/lysogenic	Yes	30.0	0.0
MSP1045	haib17KIU4866_H7HJMCCXY_SL272778.121	52711	no	Yes			Pasteurella multocida subsp. gallicida	prophage/temperate/lysogenic	Yes	22.0	0.0
MSP1046	haib17CEM4890_HKC32ALXX_SL254764.259	52704	no	Yes			Erwinia amylovora	prophage/temperate/lysogenic	No	21.0	0.0
MSP1047	haib17CEM4890_H2NYMCCXY_SL254769.259	52673	no	Yes				prophage/temperate/lysogenic	Yes	14.0	0.0
MSP1048	haib17CEM4890_H2NYMCCXY_SL254859.1	52662	no	No			Klebsiella pneumoniae subsp. pneumoniae	virulent/lytic	Yes	9.0	1.0
MSP1049	haib17CEM5106_HCVMTCCXY_SL269601.80	52631	no	Yes			Micromonospora viridifaciens	prophage/temperate/lysogenic	Yes	30.0	0.0
MSP1050	haib17CEM4890_H75CGCCXY_SL263714.95	52602	no	Yes				prophage/temperate/lysogenic	Yes	29.0	0.0
MSP1051	haib17CEM4890_HKC32ALXX_SL254706.313	52599	no	Yes				virulent/lytic	Yes	32.0	0.0
MSP1052	haib17CEM4890_HKC32ALXX_SL254764.260	52590	no	Yes			Serratia plymuthica AS9	prophage/temperate/lysogenic	Yes	26.0	0.0
MSP1054	haib17CEM4890_H2NYMCCXY_SL254854.174	52570	no	Yes			Pseudomonas stutzeri	prophage/temperate/lysogenic	Yes	32.0	1.0
MSP1055	haib17CEM4890_HKC32ALXX_SL254699.11	52559	yes	No	Arthrobacter	None	Thermaerobacter sp. FW80	virulent/lytic	Yes	29.0	0.0
MSP1056	haib17CEM4890_H2NYMCCXY_SL254842.94	52511	no	Yes				prophage/temperate/lysogenic	Yes	13.0	0.0
MSP1057	haib17CEM4890_H2NYMCCXY_SL254787.2	52412	no	No	Arthrobacter	None		virulent/lytic	Yes	25.0	0.0
MSP1058	haib17CEM5106_HCV72CCXY_SL269805.123	52411	no	No			Achromobacter xylosoxidans	prophage/temperate/lysogenic	Yes	13.0	0.0
MSP1059	haib17CEM4890_HKC32ALXX_SL254764.261	52366	no	No				virulent/lytic	Yes	22.0	0.0
MSP1060	haib17CEM5106_HCY5HCCXY_SL270942.98	52358	no	No				virulent/lytic	Yes	25.0	0.0
MSP1061	haib17CEM5106_HCVMTCCXY_SL269597.65	52344	no	Yes	Pseudomonas	Pseudomonas_fluorescens		prophage/temperate/lysogenic	Yes	33.0	1.0
MSP1062	haib17CEM4890_H2NYMCCXY_SL254817.213	52330	no	Yes				virulent/lytic	Yes	32.0	0.0
MSP1063	haib17CEM5106_HCY5HCCXY_SL270977.126	52298	no	Yes				virulent/lytic	Yes	32.0	0.0
MSP1064	haib17CEM4890_H2NYMCCXY_SL254796.261	52251	no	Yes				prophage/temperate/lysogenic	Yes	12.0	0.0
MSP1065	haib17CEM4890_HKC32ALXX_SL254681.303	52200	no	Yes			Alkalihalobacillus clausii	virulent/lytic	Yes	14.0	0.0
MSP1066	haib17KIU4866_H7HJMCCXY_SL272748.35	52183	no	No			Pseudomonas stutzeri	virulent/lytic	Yes	20.0	1.0
MSP1067	haib17CEM4890_H2NYMCCXY_SL254793.161	52174	no	Yes			Dickeya dianthicola IPO 980	prophage/temperate/lysogenic	No	18.0	0.0
MSP1068	haib17DB4959_HMCMJCCXY_SL336180.4	52151	no	No				virulent/lytic	Yes	33.0	0.0
MSP1069	haib17CEM4890_HMCMJCCXY_SL335833.168	52146	no	Yes				prophage/temperate/lysogenic	Yes	26.0	0.0
MSP1070	haib17CEM4890_HKC32ALXX_SL254699.12	52031	no	No				virulent/lytic	Yes	19.0	0.0
MSP1072	haib17CEM4890_H75CGCCXY_SL263688.168	52022	no	Yes				prophage/temperate/lysogenic	No	33.0	0.0
MSP1073	haib17CEM4890_HKC32ALXX_SL254685.3	51993	yes	No				virulent/lytic	Yes	13.0	0.0
MSP1074	haib17CEM5106_HCV72CCXY_SL269776.124	51900	no	Yes				virulent/lytic	No	15.0	0.0
MSP1075	haib17CEM4890_HKC32ALXX_SL254742.304	51884	no	No				prophage/temperate/lysogenic	Yes	15.0	0.0
MSP1076	haib17CEM4890_H75CGCCXY_SL263653.131	51786	no	Yes				virulent/lytic	No	30.0	0.0
MSP1077	haib17CEM4890_H2NYMCCXY_SL254784.464	51782	no	No				prophage/temperate/lysogenic	Yes	15.0	0.0
MSP1078	haib17CEM4890_H2NYMCCXY_SL254818.314	51747	no	No				prophage/temperate/lysogenic	Yes	12.0	0.0
MSP1079	haib17KIU4866_H7HJMCCXY_SL272804.65	51713	no	Yes			Salmonella enterica subsp. enterica serovar Sanjuan	prophage/temperate/lysogenic	No	32.0	1.0
MSP1080	haib17CEM4890_H2NYMCCXY_SL254831.311	51712	no	Yes			Pseudomonas aeruginosa SCV20265	virulent/lytic	Yes	33.0	1.0
MSP1081	haib17CEM5241_HMGMHCCXY_SL343483.2	51632	no	No				virulent/lytic	Yes	18.0	0.0
MSP1082	haib17KIU4866_HMCMJCCXY_SL335941.78	51615	no	Yes			Citrobacter koseri	prophage/temperate/lysogenic	Yes	28.0	0.0
MSP1083	haib17DB4959_HMCMJCCXY_SL336180.5	51556	yes	No				virulent/lytic	Yes	16.0	0.0
MSP1084	haib17CEM4890_HKC32ALXX_SL254716.127	51549	no	Yes				prophage/temperate/lysogenic	Yes	18.0	0.0
MSP1085	haib17CEM4890_H2NYMCCXY_SL254796.266	51530	no	Yes			Pseudomonas balearica DSM 6083	prophage/temperate/lysogenic	Yes	21.0	1.0
MSP1086	haib17CEM4890_HKC32ALXX_SL254746.38	51520	no	No				prophage/temperate/lysogenic	Yes	15.0	0.0
MSP1087	haib17CEM4890_HKC32ALXX_SL254719.9	51495	no	No				prophage/temperate/lysogenic	Yes	19.0	0.0
MSP1088	haib18CEM5453_HNHKFCCXY_SL345993.7	51485	no	No				virulent/lytic	Yes	32.0	0.0
MSP1089	haib17CEM5106_HCY5HCCXY_SL270928.115	51412	no	Yes				prophage/temperate/lysogenic	Yes	17.0	0.0
MSP1090	haib17CEM4890_HKC32ALXX_SL254681.308	51389	no	Yes				prophage/temperate/lysogenic	Yes	15.0	0.0
MSP1091	haib17CEM5106_HCVMTCCXY_SL269529.33	51367	yes	Yes				prophage/temperate/lysogenic	Yes	12.0	0.0
MSP1093	haib17KIU4866_H7HJMCCXY_SL272782.58	51319	no	No				prophage/temperate/lysogenic	Yes	30.0	0.0
MSP1094	haib17CEM5106_HCY5JCCXY_SL269564.62	51308	no	Yes				prophage/temperate/lysogenic	Yes	20.0	0.0
MSP1095	haib17CEM4890_HMCMJCCXY_SL335783.55	51271	no	No				virulent/lytic	Yes	18.0	0.0
MSP1096	haib17CEM5106_HCV72CCXY_SL269752.157	51260	yes	No			Corynebacterium sp. 2183	prophage/temperate/lysogenic	Yes	16.0	0.0
MSP1097	haib17CEM4890_H2NYMCCXY_SL254850.71	51221	yes	No	Arthrobacter	Arthrobacter_globiformis		virulent/lytic	Yes	28.0	0.0
MSP1098	haib17CEM5106_HCV72CCXY_SL269805.127	51154	no	No				prophage/temperate/lysogenic	Yes	17.0	0.0
MSP1099	haib17CEM4890_H2NYMCCXY_SL254788.65	51100	yes	No			Granulibacter bethesdensis	virulent/lytic	Yes	16.0	0.0
MSP1100	haib17KIU4866_HMCMJCCXY_SL335949.104	51099	no	No				virulent/lytic	Yes	30.0	0.0
MSP1101	haib17KIU4866_H7HJMCCXY_SL272827.91	51069	no	No				prophage/temperate/lysogenic	Yes	31.0	0.0
MSP1102	haib17CEM4890_H75CGCCXY_SL263651.75	51055	no	Yes				prophage/temperate/lysogenic	Yes	29.0	0.0
MSP1103	haib17CEM4890_H2NYMCCXY_SL254781.435	51047	no	Yes				prophage/temperate/lysogenic	Yes	12.0	0.0
MSP1104	haib18CEM5453_HMGW3CCXY_SL342529.1	51035	no	No				virulent/lytic	Yes	17.0	0.0
MSP1105	haib17CEM5106_HCV72CCXY_SL269718.124	51004	no	Yes				prophage/temperate/lysogenic	Yes	7.0	0.0
MSP1106	haib17CEM4890_HKC32ALXX_SL254705.278	50993	no	Yes			Desulfallas gibsoniae DSM 7213	prophage/temperate/lysogenic	Yes	33.0	0.0
MSP1107	haib17KIU4866_HMCMJCCXY_SL335939.46	50946	no	Yes			Acinetobacter baumannii	virulent/lytic	Yes	30.0	1.0
MSP1108	haib17DB4959_H3MGVCCXY_SL259953.36	50928	no	No	Staphylococcus	Staphylococcus_epidermidis		prophage/temperate/lysogenic	No	22.0	1.0
MSP1109	haib18CEM5453_HT5YVCCXY_SL357315.1	50906	yes	No				virulent/lytic	Yes	8.0	0.0
MSP1110	haib17CEM4890_HKC32ALXX_SL254716.133	50905	no	No				prophage/temperate/lysogenic	Yes	9.0	0.0
MSP1111	haib17CEM4890_H2NYMCCXY_SL254853.116	50863	no	No				prophage/temperate/lysogenic	Yes	6.0	0.0

MSP1113	haib17KIU4866_H7HJMCCXY_SL272824.71	50773	no	Yes				virulent/lytic	No	32.0	0.0
MSP1114	haib17DB4959_H3MGVCCXY_SL259882.49	50760	no	No				prophage/temperate/lysogenic	Yes	21.0	0.0
MSP1115	haib17CEM4890_H2NYMCCXY_SL254820.16	50746	no	Yes			Acinetobacter ursingii	prophage/temperate/lysogenic	Yes	31.0	0.0
MSP1116	haib17CEM4890_H2NYMCCXY_SL254830.314	50689	no	No				virulent/lytic	Yes	12.0	0.0
MSP1117	haib18CEM5453_HNGH3CCXY_SL344549.17	50687	no	No				prophage/temperate/lysogenic	Yes	7.0	0.0
MSP1118	haib17CEM5106_HCY5JCCXY_SL269535.171	50676	no	Yes				prophage/temperate/lysogenic	Yes	11.0	0.0
MSP1119	haib17CEM5106_HCV72CCXY_SL269750.124	50654	no	No			Pseudomonas mendocina ymp	prophage/temperate/lysogenic	Yes	33.0	0.0
MSP1120	haib17CEM4890_HKC32ALXX_SL254680.246	50644	no	No				virulent/lytic	Yes	32.0	0.0
MSP1121	haib17CEM4890_H2NYMCCXY_SL254849.57	50630	no	No	Staphylococcus	Staphylococcus_aureus	Staphylococcus schleiferi	prophage/temperate/lysogenic	Yes	20.0	0.0
MSP1122	haib17KIU4866_HMCMJCCXY_SL335869.3	50624	yes	No	Arthrobacter	None		virulent/lytic	Yes	27.0	0.0
MSP1123	haib17CEM4890_HKC32ALXX_SL254697.4	50619	yes	No	Arthrobacter	Arthrobacter_globiformis		virulent/lytic	Yes	12.0	0.0
MSP1124	haib18CEM5332_HK7G5CCXY_SL310962.1	50594	no	No				virulent/lytic	Yes	22.0	0.0
MSP1125	haib17CEM5106_HCY5HCCXY_SL271171.1	50572	no	No			Acinetobacter sp. ACNIH1	prophage/temperate/lysogenic	Yes	29.0	0.0
MSP1126	haib17CEM5106_HCVMTCCXY_SL269565.48	50557	no	Yes				prophage/temperate/lysogenic	Yes	27.0	0.0
MSP1127	haib17CEM5106_HCVMTCCXY_SL269546.79	50550	no	Yes			Propionibacterium freudenreichii	prophage/temperate/lysogenic	Yes	32.0	0.0
MSP1128	haib17DB4959_H3MGVCCXY_SL259835.13	50547	no	Yes			Miniimonas sp. S16	prophage/temperate/lysogenic	Yes	28.0	0.0
MSP1129	haib17KIU4866_H7HJMCCXY_SL272794.50	50537	no	No				virulent/lytic	Yes	29.0	0.0
MSP1130	haib17CEM4890_H2NYMCCXY_SL254795.366	50532	no	No				prophage/temperate/lysogenic	Yes	9.0	0.0
MSP1131	haib17CEM4890_HMCMJCCXY_SL335842.3	50510	no	No				virulent/lytic	Yes	17.0	0.0
MSP1133	haib17CEM4890_H2NYMCCXY_SL254794.540	50504	no	No			Klebsiella pneumoniae	prophage/temperate/lysogenic	No	26.0	1.0
MSP1134	haib17KIU4866_HMCMJCCXY_SL335939.48	50499	no	Yes				prophage/temperate/lysogenic	Yes	25.0	0.0
MSP1135	haib17CEM4890_H2NYMCCXY_SL254830.317	50479	no	No			Desulfovibrio desulfuricans ND132	prophage/temperate/lysogenic	Yes	14.0	0.0
MSP1136	haib17CEM5106_HCV72CCXY_SL269754.261	50460	no	Yes				prophage/temperate/lysogenic	Yes	31.0	0.0
MSP1137	haib17CEM5106_HCV72CCXY_SL269740.60	50457	no	No	Arthrobacter	Arthrobacter_globiformis		prophage/temperate/lysogenic	Yes	26.0	0.0
MSP1138	haib17CEM5106_HCV72CCXY_SL269732.235	50446	no	Yes				virulent/lytic	Yes	31.0	0.0
MSP1139	haib17CEM4890_H2NYMCCXY_SL254831.317	50439	no	No			Enterobacter cloacae	prophage/temperate/lysogenic	Yes	23.0	0.0
MSP1141	haib18CEM5453_HMC2KCCXY_SL336784.4	50306	no	No				prophage/temperate/lysogenic	Yes	27.0	0.0
MSP1142	haib17CEM4890_H2NYMCCXY_SL254843.466	50303	no	Yes				prophage/temperate/lysogenic	Yes	13.0	0.0
MSP1143	haib17CEM4890_H2NYMCCXY_SL254770.253	50263	yes	No	Escherichia	Escherichia_coli		virulent/lytic	Yes	9.0	1.0
MSP1144	haib17CEM4890_HKC32ALXX_SL254693.189	50244	no	Yes				prophage/temperate/lysogenic	Yes	12.0	0.0
MSP1145	haib17KIU4866_H7HJMCCXY_SL272827.92	50211	no	No			Salmonella enterica subsp. enterica serovar Kentucky	prophage/temperate/lysogenic	Yes	21.0	1.0
MSP1147	haib17CEM4890_H2NYMCCXY_SL254820.18	50202	no	Yes			Serratia sp. ATCC 39006	prophage/temperate/lysogenic	No	33.0	0.0
MSP1149	haib17CEM4890_HMCMJCCXY_SL335786.52	50175	yes	No			Corynebacterium aurimucosum ATCC 700975	prophage/temperate/lysogenic	Yes	21.0	0.0
MSP1151	haib17CEM4890_H2NYMCCXY_SL254830.319	50168	no	Yes				virulent/lytic	Yes	23.0	0.0
MSP1152	haib17CEM4890_H2NYMCCXY_SL254778.50	50166	no	Yes			Acinetobacter indicus	prophage/temperate/lysogenic	Yes	30.0	0.0
MSP1153	haib17CEM4890_H2NYMCCXY_SL254784.492	50163	no	Yes	Faecalibacterium	Faecalibacterium_prausnitzii		virulent/lytic	Yes	11.0	0.0
MSP1154	haib17CEM4890_H75CGCCXY_SL263650.109	50139	no	Yes			Pseudomonas stutzeri A1501	prophage/temperate/lysogenic	Yes	31.0	1.0
MSP1155	haib17CEM4890_HKC32ALXX_SL254688.14	50138	no	No			Bacillus thuringiensis serovar indiana	virulent/lytic	Yes	8.0	0.0
MSP1156	haib17CEM4890_HKC32ALXX_SL254675.5	50114	yes	No	Arthrobacter	None		virulent/lytic	Yes	27.0	0.0
MSP1157	haib17KIU4866_H7HJMCCXY_SL272738.39	50086	no	Yes			Hungateiclostridium thermocellum ATCC 27405	prophage/temperate/lysogenic	Yes	31.0	0.0
MSP1158	haib17CEM4890_H75CGCCXY_SL263689.118	50056	no	Yes				prophage/temperate/lysogenic	Yes	30.0	0.0
MSP1159	haib17CEM4890_H2NYMCCXY_SL254795.369	50055	no	Yes			Thermoanaerobacterium thermosaccharolyticum M0795	prophage/temperate/lysogenic	Yes	15.0	0.0
MSP1160	haib17CEM4890_H2NYMCCXY_SL254841.206	50049	no	No	Faecalibacterium	Faecalibacterium_prausnitzii		prophage/temperate/lysogenic	Yes	6.0	0.0
MSP1161	haib17KIU4866_H7HJMCCXY_SL272778.129	50021	no	Yes			Pseudomonas aeruginosa	virulent/lytic	Yes	33.0	1.0
MSP1162	haib17CEM4890_H75CGCCXY_SL263652.229	50019	no	No			Dickeya sp. CSL RW240	prophage/temperate/lysogenic	Yes	25.0	0.0
MSP1163	haib17CEM4890_H75CGCCXY_SL263637.61	50016	no	No				prophage/temperate/lysogenic	Yes	33.0	0.0
MSP1164	haib17CEM4890_H2NYMCCXY_SL254855.267	50015	no	No			Pseudomonas balearica DSM 6083	prophage/temperate/lysogenic	Yes	17.0	1.0
MSP1166	haib17CEM4890_H2NYMCCXY_SL254770.256	49969	no	No			Pseudomonas stutzeri A1501	prophage/temperate/lysogenic	Yes	18.0	1.0
MSP1167	haib17CEM4890_H3KHWCCXY_SL263315.2	49925	no	No	Pantoea	Pantoea_agglomerans		virulent/lytic	Yes	10.0	0.0
MSP1169	haib17CEM5106_HCY5HCCXY_SL270942.105	49892	no	Yes				prophage/temperate/lysogenic	Yes	27.0	0.0
MSP1170	haib17KIU4866_H7HJMCCXY_SL272744.71	49891	no	Yes			Clostridium kluyveri NBRC 12016	virulent/lytic	Yes	12.0	0.0
MSP1171	haib17CEM4890_H75CGCCXY_SL263663.86	49876	no	No				prophage/temperate/lysogenic	Yes	11.0	0.0
MSP1172	haib17CEM5106_HCVMTCCXY_SL269593.56	49842	no	Yes			Ornithinimicrobium sp. AMA3305	prophage/temperate/lysogenic	Yes	32.0	0.0
MSP1173	haib17CEM5106_HCV72CCXY_SL269761.94	49828	yes	No			Klebsiella pneumoniae subsp. pneumoniae	virulent/lytic	No	12.0	1.0
MSP1174	haib17CEM4890_H75CGCCXY_SL263665.75	49824	no	Yes			Acinetobacter sp. WCHA45	prophage/temperate/lysogenic	Yes	30.0	0.0
MSP1175	haib17CEM5106_HCV72CCXY_SL269721.46	49822	no	No			Pseudomonas stutzeri A1501	prophage/temperate/lysogenic	Yes	20.0	1.0
MSP1176	haib17CEM4890_H2NYMCCXY_SL254796.287	49814	no	Yes			Acinetobacter baumannii	prophage/temperate/lysogenic	Yes	33.0	1.0
MSP1178	haib17CEM4890_H2NYMCCXY_SL254790.95	49784	no	No				virulent/lytic	Yes	33.0	0.0
MSP1179	haib18CEM5453_HNHKFCCXY_SL345904.20	49763	no	Yes				virulent/lytic	Yes	5.0	0.0
MSP1180	haib17CEM4890_HKC32ALXX_SL254700.91	49744	yes	No				virulent/lytic	Yes	25.0	0.0
MSP1181	haib17CEM4890_HKC32ALXX_SL254705.287	49730	yes	No			Atlantibacter hermannii	prophage/temperate/lysogenic	Yes	11.0	0.0
MSP1182	haib17DB4959_H3MGVCCXY_SL259917.1	49725	no	No	Citrobacter	None		virulent/lytic	Yes	11.0	0.0
MSP1183	haib17CEM4890_H75CGCCXY_SL263654.78	49627	no	No			Acinetobacter sp. ACNIH1	prophage/temperate/lysogenic	Yes	30.0	0.0
MSP1184	haib17CEM5106_HCY5HCCXY_SL270929.96	49624	no	Yes			Moraxella osloensis	prophage/temperate/lysogenic	Yes	21.0	1.0
MSP1185	haib17CEM4890_HKC32ALXX_SL254764.272	49623	no	No				prophage/temperate/lysogenic	Yes	13.0	0.0
MSP1186	haib17KIU4866_H7HJMCCXY_SL272742.10	49585	no	Yes			Dickeya chrysanthemi Ech1591	prophage/temperate/lysogenic	No	16.0	0.0
MSP1187	haib17CEM5106_HCY5HCCXY_SL270932.62	49584	no	No				virulent/lytic	Yes	11.0	0.0
MSP1188	haib17CEM5106_HCV72CCXY_SL269724.95	49561	yes	No			Pseudomonas stutzeri A1501	prophage/temperate/lysogenic	No	19.0	1.0

MSP1189	haib17CEM4890_HKC32ALXX_SL254764.273	49552	no	Yes				prophage/temperate/lysogenic	Yes	12.0	0.0
MSP1190	haib17CEM4890_HKC32ALXX_SL254742.328	49539	no	No	Bacillus	Bacillus_anthracis		prophage/temperate/lysogenic	Yes	10.0	0.0
MSP1192	haib17CEM4890_H75CGCCXY_SL263663.88	49403	no	Yes			Vitreoscilla filiformis	prophage/temperate/lysogenic	Yes	27.0	0.0
MSP1193	haib17KIU4866_H7HJMCCXY_SL272832.97	49389	no	No				prophage/temperate/lysogenic	Yes	33.0	0.0
MSP1194	haib17KIU4866_H7HJMCCXY_SL272791.41	49386	no	Yes			Salmonella enterica	prophage/temperate/lysogenic	Yes	32.0	1.0
MSP1195	haib17CEM4890_H2NYMCCXY_SL254795.375	49376	no	Yes				prophage/temperate/lysogenic	Yes	16.0	0.0
MSP1197	haib17KIU4866_H7HJMCCXY_SL272751.95	49350	no	Yes				prophage/temperate/lysogenic	Yes	14.0	0.0
MSP1198	haib17CEM4890_H2NYMCCXY_SL254856.60	49322	no	Yes				virulent/lytic	Yes	29.0	0.0
MSP1199	haib17CEM4890_H2NYMCCXY_SL254807.226	49303	no	Yes				virulent/lytic	Yes	13.0	0.0
MSP1202	haib17CEM5106_HCV72CCXY_SL269785.125	49267	no	No				virulent/lytic	Yes	13.0	0.0
MSP1204	haib17KIU4866_H7HJMCCXY_SL272798.122	49238	no	No				prophage/temperate/lysogenic	Yes	33.0	0.0
MSP1206	haib17DB4959_H3MGVCCXY_SL259862.13	49127	no	No				virulent/lytic	Yes	13.0	0.0
MSP1207	haib18CEM5526_HMGTJCCXY_SL342628.49	49109	no	Yes				prophage/temperate/lysogenic	Yes	29.0	0.0
MSP1208	haib17KIU4866_HMCMJCCXY_SL335946.50	49107	no	Yes			Atlantibacter hermannii	prophage/temperate/lysogenic	Yes	28.0	0.0
MSP1209	haib18CEM5453_HNHKFCCXY_SL345981.14	49098	yes	No				virulent/lytic	Yes	17.0	0.0
MSP1211	haib17CEM4890_H2NYMCCXY_SL254830.329	49094	no	Yes				prophage/temperate/lysogenic	Yes	11.0	0.0
MSP1213	haib17CEM5106_HCY5HCCXY_SL270975.113	49023	no	Yes			Pseudomonas stutzeri	virulent/lytic	Yes	33.0	1.0
MSP1214	haib17CEM4890_HKC32ALXX_SL254705.291	49015	no	No				prophage/temperate/lysogenic	Yes	21.0	0.0
MSP1215	haib17CEM4890_H2NYMCCXY_SL254771.270	48987	no	Yes				prophage/temperate/lysogenic	Yes	15.0	0.0
MSP1216	haib17CEM5106_HCV72CCXY_SL269754.269	48962	no	Yes			Psychrobacter sp. YP14	prophage/temperate/lysogenic	Yes	21.0	0.0
MSP1217	haib17CEM4890_H2NYMCCXY_SL254815.29	48951	no	No				prophage/temperate/lysogenic	Yes	20.0	0.0
MSP1219	haib17CEM5106_HCV72CCXY_SL269734.29	48911	no	Yes				virulent/lytic	Yes	33.0	0.0
MSP1220	haib17CEM4890_H75CGCCXY_SL263662.61	48904	yes	No				virulent/lytic	Yes	11.0	0.0
MSP1221	haib17CEM4890_H2NYMCCXY_SL254850.80	48900	no	No	Arthrobacter	None		virulent/lytic	Yes	29.0	0.0
MSP1222	haib17CEM4890_H2NYMCCXY_SL254817.226	48893	no	No				prophage/temperate/lysogenic	Yes	13.0	0.0
MSP1223	haib17CEM4890_HMCMJCCXY_SL335845.88	48892	yes	No				prophage/temperate/lysogenic	Yes	23.0	0.0
MSP1225	haib17CEM4890_HKC32ALXX_SL254675.7	48833	yes	No				virulent/lytic	Yes	22.0	0.0
MSP1226	haib17KIU4866_HMCMJCCXY_SL335963.72	48788	no	Yes			Acinetobacter wuhouensis	prophage/temperate/lysogenic	Yes	32.0	0.0
MSP1227	haib17CEM4890_H2NYMCCXY_SL254825.21	48747	no	No				virulent/lytic	Yes	11.0	0.0
MSP1228	haib18CEM5453_HNHKFCCXY_SL346127.79	48742	no	Yes			Micrococcus luteus	prophage/temperate/lysogenic	Yes	30.0	1.0
MSP1229	haib17CEM4890_H2NYMCCXY_SL254830.335	48716	no	No				prophage/temperate/lysogenic	Yes	33.0	0.0
MSP1230	haib17CEM5106_HCV72CCXY_SL269773.152	48714	no	No			Klebsiella pneumoniae	prophage/temperate/lysogenic	No	22.0	1.0
MSP1232	haib17CEM4890_HKC32ALXX_SL254718.235	48638	no	Yes			Pseudomonas chlororaphis subsp. aurantiaca	prophage/temperate/lysogenic	Yes	26.0	0.0
MSP1233	haib17KIU4866_HMCMJCCXY_SL335925.15	48630	yes	No				virulent/lytic	Yes	3.0	0.0
MSP1234	haib17KIU4866_H7HJMCCXY_SL272803.118	48573	no	Yes	Enterobacter	None	Dickeya dianthicola IPO 980	prophage/temperate/lysogenic	Yes	17.0	0.0
MSP1235	haib17CEM4890_H2NYMCCXY_SL254784.518	48501	no	Yes			Clostridium baratii str. Sullivan	virulent/lytic	Yes	15.0	0.0
MSP1236	haib17CEM4890_H2NYMCCXY_SL254844.36	48487	no	Yes			Acinetobacter sp. ACNIH1	prophage/temperate/lysogenic	Yes	29.0	0.0
MSP1237	haib17CEM4890_H2NYMCCXY_SL254861.64	48456	no	Yes				virulent/lytic	Yes	27.0	0.0
MSP1238	haib17CEM4890_H2NYMCCXY_SL254806.328	48453	no	Yes				prophage/temperate/lysogenic	Yes	26.0	0.0
MSP1240	haib17CEM4890_H75CGCCXY_SL263685.45	48319	no	No				prophage/temperate/lysogenic	Yes	15.0	0.0
MSP1241	haib17KIU4866_H7HJMCCXY_SL272799.102	48300	no	No				virulent/lytic	Yes	7.0	0.0
MSP1242	haib17CEM4890_H2NYMCCXY_SL254831.339	48288	no	No	Bacillus	Bacillus_cereus		prophage/temperate/lysogenic	Yes	12.0	0.0
MSP1244	haib17KIU4866_H7HJMCCXY_SL272778.143	48246	no	Yes			Pseudomonas balearica DSM 6083	prophage/temperate/lysogenic	Yes	19.0	1.0
MSP1243	haib18CEM5526_HMGTJCCXY_SL342642.7	48246	no	No			Xanthomonas campestris pv. raphani 756C	virulent/lytic	Yes	32.0	1.0
MSP1245	haib18CEM5453_HMC2KCCXY_SL336638.3	48233	no	No				virulent/lytic	Yes	33.0	0.0
MSP1246	haib17CEM5106_HCY5HCCXY_SL270912.56	48207	no	Yes				virulent/lytic	No	30.0	0.0
MSP1247	haib17CEM4890_HKC32ALXX_SL254680.265	48192	no	No			Clostridium botulinum	virulent/lytic	Yes	22.0	0.0
MSP1248	haib17CEM4890_HKC32ALXX_SL254685.4	48179	no	No				virulent/lytic	Yes	4.0	0.0
MSP1249	haib17CEM5106_HCY5JCCXY_SL269532.130	48117	no	Yes				prophage/temperate/lysogenic	Yes	30.0	0.0
MSP1250	haib17CEM5106_HCY5HCCXY_SL270967.137	48087	no	Yes				prophage/temperate/lysogenic	Yes	32.0	0.0
MSP1251	haib17CEM4890_H75CGCCXY_SL263709.47	48011	no	Yes				prophage/temperate/lysogenic	Yes	16.0	0.0
MSP1252	haib17CEM5106_HCY5HCCXY_SL270952.76	47968	no	Yes				prophage/temperate/lysogenic	Yes	18.0	0.0
MSP1253	haib17CEM5106_HCY5JCCXY_SL269586.62	47928	no	Yes			Pseudomonas balearica DSM 6083	virulent/lytic	Yes	33.0	1.0
MSP1254	haib17CEM4890_H2NYMCCXY_SL254852.133	47924	no	No				prophage/temperate/lysogenic	Yes	31.0	0.0
MSP1255	haib17CEM4890_H2NYMCCXY_SL254854.200	47892	no	Yes			Dickeya dianthicola IPO 980	prophage/temperate/lysogenic	No	23.0	0.0
MSP1256	haib17CEM5106_HCY5HCCXY_SL270917.228	47890	no	Yes			Actinobaculum sp. 313	virulent/lytic	Yes	33.0	0.0
MSP1257	haib17CEM4890_H2NYMCCXY_SL254788.72	47869	no	No	Staphylococcus	Staphylococcus_aureus		prophage/temperate/lysogenic	Yes	17.0	1.0
MSP1259	haib17DB4959_H3MGVCCXY_SL259906.14	47831	no	No			Rhodobacter sphaeroides	prophage/temperate/lysogenic	Yes	27.0	0.0
MSP1260	haib18CEM5453_HMGW3CCXY_SL342282.2	47830	no	No				virulent/lytic	Yes	29.0	0.0
MSP1261	haib17CEM4890_HKC32ALXX_SL254693.199	47827	no	Yes			Actinoalloteichus hoggarensis	prophage/temperate/lysogenic	Yes	33.0	0.0
MSP1262	haib17CEM5106_HCV72CCXY_SL269784.94	47824	no	Yes				prophage/temperate/lysogenic	Yes	27.0	0.0
MSP1263	haib17CEM4890_H2NYMCCXY_SL254781.455	47794	no	Yes				prophage/temperate/lysogenic	Yes	14.0	0.0
MSP1264	haib17KIU4866_H7HJMCCXY_SL272777.77	47792	no	No			Acinetobacter sp. LoGeW2-3	prophage/temperate/lysogenic	Yes	31.0	0.0
MSP1266	haib17CEM4890_HKC32ALXX_SL254740.104	47773	no	Yes			Acinetobacter radioresistens	prophage/temperate/lysogenic	No	28.0	0.0
MSP1268	haib17KIU4866_H7HJMCCXY_SL272754.22	47750	no	No			Raoultella ornithinolytica	virulent/lytic	Yes	18.0	0.0
MSP1270	haib17KIU4866_H7HJMCCXY_SL272753.39	47734	no	No				prophage/temperate/lysogenic	Yes	12.0	0.0
MSP1272	haib17CEM5106_HCY5HCCXY_SL271110.105	47698	no	Yes			Klebsiella pneumoniae subsp. pneumoniae	prophage/temperate/lysogenic	Yes	31.0	1.0
MSP1273	haib17KIU4866_H7HJMCCXY_SL272749.24	47697	no	No			Acinetobacter baumannii	virulent/lytic	Yes	31.0	1.0

MSP1275	haib17CEM5106_HCY5JCCXY_SL269546.126	47687	no	Yes			Acinetobacter defluvii	virulent/lytic	Yes	26.0	0.0
MSP1276	haib17CEM5106_HCY5JCCXY_SL269610.106	47686	no	No				prophage/temperate/lysogenic	Yes	18.0	0.0
MSP1277	haib17CEM5106_HCV72CCXY_SL269750.129	47629	no	Yes	Pseudomonas	Pseudomonas_aeruginosa	Pseudomonas fluorescens	virulent/lytic	Yes	33.0	0.0
MSP1278	haib17CEM5106_HCV72CCXY_SL269769.88	47628	no	No	Gordonia	Gordonia_terrae	Chelatococcus daeguensis	virulent/lytic	Yes	11.0	0.0
MSP1279	haib17CEM5106_HCY5HCCXY_SL270988.180	47622	no	No				virulent/lytic	No	27.0	0.0
MSP1280	haib17KIU4866_H7HJMCCXY_SL272826.202	47592	no	No				prophage/temperate/lysogenic	Yes	28.0	0.0
MSP1281	haib18CEM5453_HNHKFCCXY_SL346119.8	47590	no	Yes			Lawsonella clevelandensis	virulent/lytic	No	30.0	0.0
MSP1283	haib17KIU4866_H7HJMCCXY_SL272756.50	47558	yes	No				virulent/lytic	Yes	25.0	0.0
MSP1285	haib17KIU4866_H7HJMCCXY_SL272772.46	47538	yes	No			Pseudomonas stutzeri	prophage/temperate/lysogenic	Yes	20.0	1.0
MSP1286	haib17CEM4890_H75CGCCXY_SL263721.109	47513	no	Yes				prophage/temperate/lysogenic	Yes	28.0	0.0
MSP1288	haib17CEM4890_H2NYMCCXY_SL254782.260	47479	no	Yes				prophage/temperate/lysogenic	Yes	27.0	0.0
MSP1289	haib18CEM5453_HNHKFCCXY_SL345946.9	47455	no	No				prophage/temperate/lysogenic	Yes	32.0	0.0
MSP1290	haib17KIU4866_HMCMJCCXY_SL335963.75	47450	no	Yes				prophage/temperate/lysogenic	Yes	31.0	0.0
MSP1292	haib17KIU4866_H7HJMCCXY_SL272824.88	47445	no	Yes				prophage/temperate/lysogenic	Yes	9.0	0.0
MSP1293	haib17CEM5106_HCY5HCCXY_SL270909.66	47444	no	No				prophage/temperate/lysogenic	Yes	6.0	0.0
MSP1294	haib17CEM5106_HCY5JCCXY_SL269589.57	47410	no	Yes				prophage/temperate/lysogenic	Yes	15.0	0.0
MSP1295	haib17CEM4890_H2NYMCCXY_SL254806.337	47398	no	Yes			Delftia acidovorans	prophage/temperate/lysogenic	Yes	24.0	0.0
MSP1296	haib17CEM5241_HMGMHCCXY_SL343449.9	47379	no	No				prophage/temperate/lysogenic	Yes	7.0	0.0
MSP1297	haib17CEM5106_HCY5JCCXY_SL269560.42	47345	no	Yes			Pseudomonas stutzeri	prophage/temperate/lysogenic	Yes	30.0	1.0
MSP1298	haib17CEM4890_H2NYMCCXY_SL254830.349	47339	yes	No				virulent/lytic	No	24.0	0.0
MSP1299	haib17CEM4890_H75CGCCXY_SL263665.80	47332	no	Yes			Acinetobacter sp. ACNIH1	prophage/temperate/lysogenic	Yes	31.0	0.0
MSP1300	haib17KIU4866_H7HJMCCXY_SL272778.147	47326	yes	No			Pseudomonas stutzeri A1501	prophage/temperate/lysogenic	Yes	20.0	1.0
MSP1301	haib17CEM4890_H2NYMCCXY_SL254805.277	47319	no	Yes				prophage/temperate/lysogenic	Yes	13.0	0.0
MSP1302	haib18CEM5453_HNGH3CCXY_SL344537.1	47299	yes	No				virulent/lytic	Yes	6.0	0.0
MSP1303	haib17CEM4890_H2NYMCCXY_SL254848.68	47288	no	Yes				prophage/temperate/lysogenic	Yes	6.0	0.0
MSP1304	haib17CEM4890_H2NYMCCXY_SL254830.352	47286	no	Yes			Escherichia coli	virulent/lytic	Yes	23.0	1.0
MSP1305	haib17CEM5106_HCY5JCCXY_SL269526.106	47264	no	Yes				prophage/temperate/lysogenic	Yes	31.0	0.0
MSP1306	haib17KIU4866_H7HJMCCXY_SL272796.194	47261	no	Yes	Burkholderia	Burkholderia_thailandensis	Izhakiella sp. KSNA2	prophage/temperate/lysogenic	No	30.0	0.0
MSP1307	haib17CEM5106_HCVMTCCXY_SL269598.131	47252	no	Yes				prophage/temperate/lysogenic	Yes	10.0	0.0
MSP1308	haib17CEM4890_H2NYMCCXY_SL254844.42	47246	no	Yes			Acinetobacter sp. ACNIH1	prophage/temperate/lysogenic	Yes	29.0	0.0
MSP1309	haib17CEM5106_HCY5HCCXY_SL270906.83	47216	no	Yes				prophage/temperate/lysogenic	Yes	24.0	0.0
MSP1310	haib17CEM4890_H2NYMCCXY_SL254794.577	47198	no	Yes				prophage/temperate/lysogenic	Yes	9.0	0.0
MSP1312	haib17CEM5106_HCV72CCXY_SL269759.42	47191	no	Yes				prophage/temperate/lysogenic	Yes	20.0	0.0
MSP1313	haib17CEM4890_HKC32ALXX_SL254762.53	47160	no	Yes				prophage/temperate/lysogenic	Yes	30.0	0.0
MSP1314	haib17CEM4890_H2NYMCCXY_SL254782.262	47122	no	Yes			Azospirillum ramasamyi	prophage/temperate/lysogenic	Yes	15.0	0.0
MSP1315	haib17CEM4890_H75CGCCXY_SL263640.173	47108	no	Yes				prophage/temperate/lysogenic	Yes	27.0	0.0
MSP1316	haib17KIU4866_H7HJMCCXY_SL272824.90	47088	no	Yes			Pseudomonas stutzeri A1501	virulent/lytic	Yes	24.0	1.0
MSP1318	haib17CEM4890_HKC32ALXX_SL254742.347	47019	no	Yes				prophage/temperate/lysogenic	Yes	10.0	0.0
MSP1319	haib17CEM4890_HMCMJCCXY_SL335783.65	46974	no	Yes			Acinetobacter sp. ACNIH2	virulent/lytic	Yes	19.0	0.0
MSP1320	haib17CEM4890_H2NYMCCXY_SL254784.534	46956	no	No				prophage/temperate/lysogenic	Yes	10.0	0.0
MSP1322	haib17CEM4890_H3KHWCCXY_SL263311.1	46929	yes	No	Staphylococcus	Staphylococcus_aureus	Vagococcus sp. CF-49	prophage/temperate/lysogenic	Yes	19.0	0.0
MSP1323	haib17CEM4890_H75CGCCXY_SL263647.116	46882	no	Yes			Delftia acidovorans	prophage/temperate/lysogenic	Yes	31.0	0.0
MSP1324	haib17CEM4890_H75CGCCXY_SL263654.82	46867	no	Yes			Serratia plymuthica AS9	prophage/temperate/lysogenic	Yes	21.0	0.0
MSP1326	haib18CEM5453_HMC2KCCXY_SL336828.1	46790	no	Yes			Micrococcus luteus	prophage/temperate/lysogenic	No	32.0	1.0
MSP1327	haib17KIU4866_H7HJMCCXY_SL272824.92	46789	no	Yes				virulent/lytic	Yes	33.0	0.0
MSP1328	haib17CEM4890_H2NYMCCXY_SL254844.43	46778	no	Yes			Ruminococcus bicirculans	prophage/temperate/lysogenic	Yes	24.0	0.0
MSP1329	haib18CEM5453_HT5YVCCXY_SL357286.5	46767	no	No				virulent/lytic	Yes	14.0	0.0
MSP1330	haib17CEM4890_H2NYMCCXY_SL254807.237	46727	no	No				virulent/lytic	Yes	11.0	0.0
MSP1331	haib17CEM4890_H2NYMCCXY_SL254796.323	46719	no	Yes			Acinetobacter soli	prophage/temperate/lysogenic	Yes	22.0	0.0
MSP1332	haib18CEM5453_HMC2KCCXY_SL336783.13	46707	no	No				prophage/temperate/lysogenic	Yes	20.0	0.0
MSP1333	haib17CEM4890_H2NYMCCXY_SL254784.539	46698	no	Yes			Blautia producta	prophage/temperate/lysogenic	Yes	13.0	0.0
MSP1334	haib17CEM4890_H2NYMCCXY_SL254818.364	46668	no	Yes			Pseudomonas pseudoalcaligenes CECT 5344	virulent/lytic	Yes	33.0	0.0
MSP1335	haib18CEM5453_HNHKFCCXY_SL345981.15	46661	no	No			Methylomicrobium album BG8	virulent/lytic	Yes	33.0	0.0
MSP1337	haib17CEM4890_HKC32ALXX_SL254764.294	46604	yes	Yes				prophage/temperate/lysogenic	Yes	7.0	0.0
MSP1338	haib17CEM4890_HKC32ALXX_SL254711.88	46599	yes	No			Corynebacterium striatum	prophage/temperate/lysogenic	No	30.0	0.0
MSP1339	haib17CEM4890_H2NYMCCXY_SL254794.586	46592	no	Yes			Acinetobacter sp. ACNIH1	prophage/temperate/lysogenic	Yes	29.0	0.0
MSP1340	haib17CEM5106_HCY5JCCXY_SL269601.82	46560	no	No				virulent/lytic	Yes	33.0	0.0
MSP1341	haib17CEM4890_H2NYMCCXY_SL254820.23	46559	no	No			Acinetobacter baumannii	prophage/temperate/lysogenic	Yes	27.0	1.0
MSP1342	haib17KIU4866_H7HJMCCXY_SL272762.52	46548	no	No			Leptothrix cholodnii SP-6	prophage/temperate/lysogenic	No	24.0	0.0
MSP1344	haib17KIU4866_HMCMJCCXY_SL335952.57	46519	no	Yes			Acinetobacter radioresistens	virulent/lytic	Yes	27.0	0.0
MSP1346	haib17CEM4890_HKC32ALXX_SL254681.350	46492	no	Yes				virulent/lytic	Yes	14.0	0.0
MSP1345	haib17KIU4866_HMCMJCCXY_SL335925.17	46492	yes	No				virulent/lytic	Yes	6.0	0.0
MSP1347	haib17KIU4866_H7HJMCCXY_SL272800.178	46450	no	No			Pseudomonas stutzeri A1501	prophage/temperate/lysogenic	Yes	19.0	1.0
MSP1348	haib17CEM4890_H2NYMCCXY_SL254814.130	46412	no	No			Acinetobacter sp. ACNIH1	prophage/temperate/lysogenic	Yes	27.0	0.0
MSP1349	haib17CEM4890_H75CGCCXY_SL263687.82	46398	no	No				prophage/temperate/lysogenic	Yes	19.0	0.0
MSP1350	haib17CEM4890_HKC32ALXX_SL254764.295	46383	yes	No				prophage/temperate/lysogenic	Yes	16.0	0.0
MSP1351	haib17CEM4890_H2NYMCCXY_SL254772.400	46374	no	No	Pseudomonas	Pseudomonas_aeruginosa		prophage/temperate/lysogenic	Yes	22.0	1.0
MSP1352	haib17CEM4890_H2NYMCCXY_SL254784.551	46332	no	Yes				prophage/temperate/lysogenic	Yes	5.0	0.0

MSP1353	haib17CEM5106_HCY5JCCXY_SL269596.165	46328	no	Yes			Enterococcus faecalis	prophage/temperate/lysogenic	Yes	22.0	1.0
MSP1355	haib17CEM4890_HKC32ALXX_SL254719.19	46322	no	No			Dickeya fangzhongdai	prophage/temperate/lysogenic	Yes	15.0	0.0
MSP1354	haib18CEM5453_HNHKFCCXY_SL345455.1	46322	no	No				prophage/temperate/lysogenic	Yes	1.0	0.0
MSP1358	haib17CEM4890_H2NYMCCXY_SL254805.283	46285	no	Yes				virulent/lytic	Yes	31.0	0.0
MSP1360	haib17CEM4890_H2NYMCCXY_SL254805.284	46268	no	Yes			Xanthomonas campestris pv. raphani 756C	virulent/lytic	Yes	33.0	1.0
MSP1359	haib17CEM4890_HKC32ALXX_SL254746.43	46268	yes	No				virulent/lytic	Yes	14.0	0.0
MSP1361	haib17CEM4890_H2NYMCCXY_SL254820.24	46266	no	No			Klebsiella variicola	prophage/temperate/lysogenic	Yes	33.0	0.0
MSP1362	haib17CEM5106_HCY5HCCXY_SL270942.118	46265	no	Yes			Pseudomonas parafulva	prophage/temperate/lysogenic	Yes	32.0	0.0
MSP1363	haib17CEM5106_HCY5HCCXY_SL270912.59	46238	no	Yes			Salmonella enterica subsp. enterica serovar Montevideo str.	prophage/temperate/lysogenic	No	32.0	1.0
MSP1364	haib17CEM5106_HCV72CCXY_SL269750.135	46235	no	No			Pseudomonas aeruginosa	prophage/temperate/lysogenic	Yes	27.0	1.0
MSP1365	haib17CEM4890_H75CGCCXY_SL263687.83	46229	no	Yes			Halomonas hydrothermalis	virulent/lytic	Yes	23.0	0.0
MSP1366	haib17CEM4890_H2NYMCCXY_SL254784.554	46228	no	Yes				prophage/temperate/lysogenic	Yes	11.0	0.0
MSP1367	haib17CEM4890_H2NYMCCXY_SL254838.95	46216	no	No				virulent/lytic	Yes	21.0	0.0
MSP1368	haib17CEM4890_H2NYMCCXY_SL254806.352	46215	no	Yes				prophage/temperate/lysogenic	No	24.0	0.0
MSP1370	haib17CEM5106_HCY5HCCXY_SL270958.89	46187	yes	No				prophage/temperate/lysogenic	Yes	17.0	0.0
MSP1371	haib18CEM5453_HMGN5CCXY_SL344649.1	46183	yes	No				virulent/lytic	Yes	2.0	0.0
MSP1372	haib18CEM5453_HNHKFCCXY_SL345945.4	46163	no	No				prophage/temperate/lysogenic	Yes	32.0	0.0
MSP1373	haib17CEM4890_HKC32ALXX_SL254716.162	46134	no	Yes				prophage/temperate/lysogenic	Yes	18.0	0.0
MSP1374	haib17CEM4890_HMCMJCCXY_SL335843.72	46126	no	Yes				prophage/temperate/lysogenic	Yes	21.0	0.0
MSP1375	haib17CEM4890_H7KYMCCXY_SL273105.3	46117	no	No			Klebsiella variicola	virulent/lytic	Yes	12.0	0.0
MSP1376	haib17CEM4890_H75CGCCXY_SL263665.83	46102	yes	No				virulent/lytic	Yes	18.0	0.0
MSP1377	haib17DB4959_H3MGVCCXY_SL259975.42	46063	no	No	Staphylococcus	Staphylococcus_aureus		prophage/temperate/lysogenic	Yes	28.0	1.0
MSP1378	haib17CEM4890_H2NYMCCXY_SL254805.285	46049	no	No				prophage/temperate/lysogenic	Yes	23.0	0.0
MSP1379	haib17CEM4890_H75CGCCXY_SL263641.143	46042	no	No			Pseudomonas balearica DSM 6083	prophage/temperate/lysogenic	Yes	28.0	1.0
MSP1380	haib17CEM4890_H75CGCCXY_SL263712.135	46016	no	No			Cronobacter sakazakii SP291	prophage/temperate/lysogenic	Yes	22.0	0.0
MSP1383	haib17CEM5106_HCVMTCCXY_SL269564.47	46004	no	No			Atlantibacter hermannii	prophage/temperate/lysogenic	Yes	19.0	0.0
MSP1382	haib17KIU4866_H7HJMCCXY_SL272768.88	46004	no	No			Pseudomonas balearica DSM 6083	prophage/temperate/lysogenic	Yes	13.0	1.0
MSP1384	haib17CEM4890_HKC32ALXX_SL254711.90	45995	yes	No			Klebsiella pneumoniae	virulent/lytic	Yes	14.0	1.0
MSP1387	haib17CEM4890_H2NYMCCXY_SL254772.404	45987	no	No				prophage/temperate/lysogenic	Yes	7.0	0.0
MSP1388	haib17CEM4890_H2NYMCCXY_SL254793.213	45981	no	Yes			Escherichia coli HS	virulent/lytic	Yes	27.0	1.0
MSP1389	haib17CEM5106_HCY5HCCXY_SL270943.58	45969	no	Yes				prophage/temperate/lysogenic	Yes	20.0	0.0
MSP1390	haib17CEM4890_H7KYMCCXY_SL273056.23	45968	no	No			Yersinia pseudotuberculosis YPIII	prophage/temperate/lysogenic	Yes	32.0	0.0
MSP1391	haib17CEM5241_HMGMHCCXY_SL343453.2	45952	no	No				prophage/temperate/lysogenic	Yes	33.0	0.0
MSP1392	haib17CEM5106_HCV72CCXY_SL269733.210	45917	no	Yes				prophage/temperate/lysogenic	Yes	32.0	0.0
MSP1393	haib17CEM5106_HCY5HCCXY_SL271123.48	45916	no	Yes				virulent/lytic	Yes	32.0	0.0
MSP1394	haib17CEM4890_H2NYMCCXY_SL254784.557	45910	no	Yes				virulent/lytic	Yes	18.0	0.0
MSP1395	haib17CEM4890_H75CGCCXY_SL263714.119	45876	no	Yes			Desulfarculus baarsii DSM 2075	prophage/temperate/lysogenic	Yes	26.0	0.0
MSP1397	haib17CEM4890_HKC32ALXX_SL254716.164	45820	no	Yes				prophage/temperate/lysogenic	Yes	13.0	0.0
MSP1399	haib17CEM4890_H2NYMCCXY_SL254772.408	45779	yes	No				prophage/temperate/lysogenic	Yes	22.0	0.0
MSP1400	haib17CEM4890_H2NYMCCXY_SL254783.458	45764	no	Yes			Pectobacterium parmentieri	prophage/temperate/lysogenic	Yes	31.0	0.0
MSP1401	haib17KIU4866_H7HJMCCXY_SL272752.87	45760	no	No			Alkalihalobacillus clausii	virulent/lytic	Yes	22.0	0.0
MSP1402	haib17CEM4890_H7KYMCCXY_SL273060.58	45751	no	No				virulent/lytic	Yes	22.0	0.0
MSP1403	haib17CEM5106_HCV72CCXY_SL269754.288	45731	no	No	Gordonia	Gordonia_terrae		prophage/temperate/lysogenic	Yes	23.0	0.0
MSP1404	haib17CEM5106_HCV72CCXY_SL269747.65	45727	no	Yes				virulent/lytic	Yes	18.0	0.0
MSP1405	haib18CEM5453_HNHKFCCXY_SL345483.1	45698	no	Yes				prophage/temperate/lysogenic	Yes	32.0	0.0
MSP1406	haib17CEM5106_HCY5HCCXY_SL270960.46	45690	yes	No			Klebsiella pneumoniae subsp. pneumoniae	virulent/lytic	Yes	21.0	1.0
MSP1408	haib17CEM4890_H75CGCCXY_SL263652.253	45687	no	No				prophage/temperate/lysogenic	Yes	18.0	0.0
MSP1409	haib17DB4959_H3MGVCCXY_SL259838.38	45650	no	Yes			Acidovorax avenae subsp. avenae ATCC 19860	prophage/temperate/lysogenic	Yes	21.0	0.0
MSP1411	haib17CEM4890_H2NYMCCXY_SL254818.373	45641	no	No				virulent/lytic	Yes	11.0	0.0
MSP1412	haib17CEM4890_HMCMJCCXY_SL335783.70	45622	no	Yes				prophage/temperate/lysogenic	Yes	32.0	0.0
MSP1413	haib17KIU4866_H7HJMCCXY_SL272797.16	45595	no	No				virulent/lytic	Yes	18.0	0.0
MSP1414	haib17KIU4866_H7HJMCCXY_SL272775.76	45594	no	No	Pseudomonas	Pseudomonas_aeruginosa	Pseudomonas chlororaphis subsp. aurantiaca	prophage/temperate/lysogenic	Yes	17.0	0.0
MSP1415	haib17CEM4890_H2NYMCCXY_SL254771.296	45587	no	Yes				prophage/temperate/lysogenic	Yes	33.0	0.0
MSP1416	haib17DB4959_HMGW3CCXY_SL342474.13	45570	no	Yes			Rothia mucilaginosa	prophage/temperate/lysogenic	No	32.0	1.0
MSP1417	haib17CEM4890_H2NYMCCXY_SL254806.356	45569	no	No			Cronobacter sakazakii ES15	prophage/temperate/lysogenic	Yes	16.0	0.0
MSP1418	haib18CEM5332_HK7G5CCXY_SL310979.6	45557	no	No				virulent/lytic	Yes	11.0	0.0
MSP1420	haib17CEM4890_H2NYMCCXY_SL254809.25	45543	yes	No				virulent/lytic	Yes	12.0	0.0
MSP1419	haib18CEM5453_HMCMJCCXY_SL336349.2	45543	yes	No				virulent/lytic	Yes	3.0	0.0
MSP1421	haib17KIU4866_HMCMJCCXY_SL335950.64	45542	no	No			Pseudomonas stutzeri	prophage/temperate/lysogenic	Yes	22.0	1.0
MSP1423	haib17CEM4890_H75CGCCXY_SL263650.123	45468	no	No				prophage/temperate/lysogenic	Yes	14.0	0.0
MSP1424	haib17CEM5106_HCV72CCXY_SL269767.111	45452	no	Yes				prophage/temperate/lysogenic	Yes	28.0	0.0
MSP1425	haib17CEM4890_H2NYMCCXY_SL254787.3	45422	no	No	Staphylococcus	Staphylococcus_epidermidis	Staphylococcus sp. M0911	prophage/temperate/lysogenic	Yes	20.0	0.0
MSP1427	haib17CEM4890_H2NYMCCXY_SL254777.3	45405	no	No			Propionibacterium acidifaciens	virulent/lytic	No	33.0	0.0
MSP1428	haib18CEM5453_HT5YVCCXY_SL357341.3	45385	yes	No				prophage/temperate/lysogenic	Yes	16.0	0.0
MSP1429	haib17CEM5106_HCV72CCXY_SL269793.80	45377	no	No				prophage/temperate/lysogenic	Yes	32.0	0.0
MSP1432	haib17CEM5106_HCY5HCCXY_SL270948.133	45325	no	Yes				prophage/temperate/lysogenic	Yes	28.0	0.0
MSP1433	haib18CEM5453_HMC2KCCXY_SL336681.34	45324	yes	No	Arthrobacter	None	Micrococcus luteus	virulent/lytic	Yes	31.0	0.0
MSP1434	haib17CEM5241_HMGMHCCXY_SL343467.1	45313	yes	No				prophage/temperate/lysogenic	Yes	7.0	0.0

MSP1435	haib17CEM5106_HCY5HCCXY_SL270939.36	45301	no	Yes	Lactococcus	Lactococcus_garvieae		prophage/temperate/lysogenic	Yes	28.0	0.0
MSP1436	haib17CEM5106_HCVMTCCXY_SL269538.229	45298	no	Yes	Arthrobacter	Arthrobacter_globiformis	Bifidobacterium choerinum	virulent/lytic	Yes	29.0	0.0
MSP1438	haib17CEM4890_HKC32ALXX_SL254681.362	45245	no	No				prophage/temperate/lysogenic	Yes	11.0	0.0
MSP1439	haib17CEM4890_HMCMJCCXY_SL335821.26	45219	no	No			Micrococcus luteus	prophage/temperate/lysogenic	Yes	33.0	1.0
MSP1440	haib17KIU4866_H7HJMCCXY_SL272744.88	45211	no	No				virulent/lytic	Yes	2.0	0.0
MSP1441	haib17CEM4890_H7KYMCCXY_SL273071.1	45207	no	No			Serratia plymuthica AS9	virulent/lytic	Yes	32.0	0.0
MSP1442	haib17CEM5106_HCY5HCCXY_SL270943.61	45204	no	Yes			Xanthomonas campestris pv. raphani 756C	prophage/temperate/lysogenic	Yes	20.0	1.0
MSP1444	haib17KIU4866_H7HJMCCXY_SL272816.132	45202	no	Yes	Stenotrophomonas	None		prophage/temperate/lysogenic	Yes	32.0	0.0
MSP1443	haib17KIU4866_HMCMJCCXY_SL335947.120	45202	no	Yes			Dickeya zeae NCPPB 3531	prophage/temperate/lysogenic	Yes	20.0	0.0
MSP1445	haib18CEM5526_HMGTJCCXY_SL342642.9	45199	no	Yes			Vitreoscilla filiformis	prophage/temperate/lysogenic	Yes	32.0	0.0
MSP1448	haib17CEM5106_HCY5HCCXY_SL270908.42	45168	no	Yes	Paracoccus	None		prophage/temperate/lysogenic	Yes	11.0	0.0
MSP1449	haib17CEM4890_H2NYMCCXY_SL254770.295	45165	no	No			Atlantibacter hermannii	prophage/temperate/lysogenic	No	27.0	0.0
MSP1450	haib17KIU4866_H7HJMCCXY_SL272827.109	45149	no	Yes			Serratia plymuthica AS9	prophage/temperate/lysogenic	No	32.0	0.0
MSP1451	haib17CEM4890_H2NYMCCXY_SL254830.368	45142	no	Yes				prophage/temperate/lysogenic	Yes	20.0	0.0
MSP1453	haib17CEM4890_HKC32ALXX_SL254705.317	45135	no	Yes				virulent/lytic	Yes	29.0	0.0
MSP1454	haib17DB4959_H3MGVCCXY_SL259906.18	45130	yes	No			Micrococcus luteus	prophage/temperate/lysogenic	Yes	31.0	1.0
MSP1456	haib17CEM5106_HCV72CCXY_SL269723.62	45108	no	No			Klebsiella sp. PO552	prophage/temperate/lysogenic	No	28.0	0.0
MSP1457	haib17CEM4890_HKC32ALXX_SL254752.160	45086	no	Yes			Klebsiella pneumoniae	prophage/temperate/lysogenic	No	25.0	1.0
MSP1458	haib17CEM4890_H2NYMCCXY_SL254828.105	45085	no	No				prophage/temperate/lysogenic	Yes	30.0	0.0
MSP1459	haib17KIU4866_H7HJMCCXY_SL272752.90	45082	no	Yes				prophage/temperate/lysogenic	Yes	30.0	0.0
MSP1460	haib17CEM4890_H2NYMCCXY_SL254773.2	45004	yes	No	Staphylococcus	Staphylococcus_aureus	Staphylococcus argenteus	prophage/temperate/lysogenic	Yes	20.0	0.0
MSP1461	haib17CEM4890_HKC32ALXX_SL254750.16	44982	no	No			Corynebacterium singulare	prophage/temperate/lysogenic	Yes	21.0	0.0
MSP1462	haib17CEM5106_HCY5JCCXY_SL269547.21	44980	no	Yes			Serratia marcescens	prophage/temperate/lysogenic	No	27.0	0.0
MSP1463	haib17CEM5106_HCY5JCCXY_SL269530.40	44951	yes	No			Enterobacter cloacae	prophage/temperate/lysogenic	Yes	19.0	0.0
MSP1466	haib17KIU4866_H7HJMCCXY_SL272774.49	44949	yes	No			Pseudomonas stutzeri A1501	prophage/temperate/lysogenic	Yes	21.0	1.0
MSP1468	haib17CEM5106_HCV72CCXY_SL269773.172	44918	no	No				virulent/lytic	Yes	25.0	0.0
MSP1469	haib17KIU4866_H7HJMCCXY_SL272772.53	44903	no	Yes				prophage/temperate/lysogenic	Yes	29.0	0.0
MSP1470	haib17CEM5106_HCY5JCCXY_SL269551.60	44889	no	Yes			Dickeya zeae	prophage/temperate/lysogenic	Yes	21.0	0.0
MSP1472	haib17CEM4890_HKC32ALXX_SL254722.43	44880	no	No			Actinomyces sp. oral taxon 897	virulent/lytic	Yes	12.0	0.0
MSP1473	haib17CEM4890_H75CGCCXY_SL263652.262	44862	no	Yes				prophage/temperate/lysogenic	Yes	16.0	0.0
MSP1474	haib17CEM4890_HKC32ALXX_SL254675.11	44852	yes	No				virulent/lytic	Yes	18.0	0.0
MSP1475	haib18CEM5453_HT5YVCCXY_SL357286.6	44850	no	No				virulent/lytic	Yes	20.0	0.0
MSP1476	haib17CEM4890_H2NYMCCXY_SL254795.431	44845	no	Yes			Pseudomonas balearica DSM 6083	virulent/lytic	Yes	26.0	1.0
MSP1477	haib17CEM4890_HKC32ALXX_SL254698.16	44836	yes	No				prophage/temperate/lysogenic	No	19.0	0.0
MSP1478	haib17KIU4866_H7HJMCCXY_SL272752.92	44822	no	No				virulent/lytic	Yes	28.0	0.0
MSP1479	haib17KIU4866_H7HJMCCXY_SL272832.138	44796	no	No				prophage/temperate/lysogenic	Yes	17.0	0.0
MSP1480	haib17KIU4866_H7HJMCCXY_SL272830.31	44769	no	Yes			Pseudomonas aeruginosa	virulent/lytic	Yes	28.0	1.0
MSP1482	haib17CEM4890_H75CGCCXY_SL263651.82	44764	no	No				virulent/lytic	Yes	4.0	0.0
MSP1483	haib17KIU4866_H7HJMCCXY_SL272824.106	44753	no	Yes			Pectobacterium brasiliense	prophage/temperate/lysogenic	No	26.0	0.0
MSP1484	haib17CEM4890_H2NYMCCXY_SL254782.286	44751	no	No				prophage/temperate/lysogenic	Yes	13.0	0.0
MSP1485	haib17KIU4866_HMCMJCCXY_SL335878.6	44747	no	No				virulent/lytic	Yes	32.0	0.0
MSP1487	haib17KIU4866_H7HJMCCXY_SL272801.6	44726	no	No				virulent/lytic	Yes	6.0	0.0
MSP1489	haib17CEM5106_HCVMTCCXY_SL269574.107	44721	no	No				prophage/temperate/lysogenic	Yes	32.0	0.0
MSP1488	haib17KIU4866_H7HJMCCXY_SL272776.146	44721	yes	No				prophage/temperate/lysogenic	Yes	3.0	0.0
MSP1490	haib17CEM4890_H75CGCCXY_SL263640.181	44686	no	Yes			Delftia sp. HK171	prophage/temperate/lysogenic	Yes	26.0	0.0
MSP1491	haib17CEM4890_H2NYMCCXY_SL254796.344	44674	no	No				prophage/temperate/lysogenic	Yes	11.0	0.0
MSP1492	haib17KIU4866_HMCMJCCXY_SL335936.90	44661	no	Yes			Pluralibacter gergoviae	virulent/lytic	Yes	17.0	0.0
MSP1493	haib17CEM4890_H2NYMCCXY_SL254847.55	44642	yes	No				virulent/lytic	No	18.0	0.0
MSP1494	haib17CEM4890_HMCMJCCXY_SL335834.149	44630	no	No				virulent/lytic	Yes	4.0	0.0
MSP1495	haib17CEM4890_HKC32ALXX_SL254752.162	44629	no	Yes			Escherichia coli	prophage/temperate/lysogenic	Yes	32.0	1.0
MSP1496	haib17CEM4890_H2NYMCCXY_SL254776.108	44619	no	No			Pseudomonas pseudoalcaligenes CECT 5344	virulent/lytic	No	15.0	0.0
MSP1497	haib18CEM5453_HMC2KCCXY_SL336736.6	44611	no	No				prophage/temperate/lysogenic	Yes	19.0	0.0
MSP1498	haib17DB4959_H3MGVCCXY_SL259896.5	44592	yes	No				prophage/temperate/lysogenic	Yes	21.0	0.0
MSP1500	haib17CEM5106_HCY5HCCXY_SL270934.103	44591	no	Yes				prophage/temperate/lysogenic	Yes	13.0	0.0
MSP1501	haib18CEM5332_HK7G5CCXY_SL311022.33	44580	no	No				virulent/lytic	Yes	31.0	0.0
MSP1502	haib18CEM5453_HNHKFCCXY_SL346098.1	44568	no	No				prophage/temperate/lysogenic	Yes	28.0	0.0
MSP1503	haib17KIU4866_H7HJMCCXY_SL272820.45	44566	no	Yes			Clostridium tyrobutyricum	prophage/temperate/lysogenic	Yes	6.0	0.0
MSP1504	haib17KIU4866_H7HJMCCXY_SL272826.222	44563	no	Yes			Desulfallas gibsoniae DSM 7213	prophage/temperate/lysogenic	Yes	14.0	0.0
MSP1505	haib17CEM4890_H2NYMCCXY_SL254855.299	44560	no	No				virulent/lytic	Yes	33.0	0.0
MSP1506	haib17CEM4890_HKC32ALXX_SL254746.44	44555	no	Yes			Pseudomonas mendocina ymp	virulent/lytic	Yes	22.0	0.0
MSP1507	haib17CEM5106_HCCGHCCXY_SL270438.10	44552	no	No			Corynebacterium singulare	prophage/temperate/lysogenic	Yes	24.0	0.0
MSP1508	haib17CEM4890_H2NYMCCXY_SL254807.249	44548	no	No				prophage/temperate/lysogenic	Yes	32.0	0.0
MSP1509	haib17CEM4890_H7KYMCCXY_SL273060.61	44531	no	No				virulent/lytic	Yes	13.0	0.0
MSP1510	haib17KIU4866_H7HJMCCXY_SL272808.29	44522	no	No				prophage/temperate/lysogenic	Yes	30.0	0.0
MSP1511	haib17CEM4890_H2NYMCCXY_SL254843.542	44512	no	No				prophage/temperate/lysogenic	Yes	13.0	0.0
MSP1512	haib17KIU4866_H7HJMCCXY_SL272782.64	44457	no	No				prophage/temperate/lysogenic	Yes	10.0	0.0
MSP1513	haib17CEM4890_H2NYMCCXY_SL254818.393	44432	no	No			Methylomicrobium album BG8	prophage/temperate/lysogenic	Yes	22.0	0.0
MSP1514	haib17CEM4890_H3KHWCCXY_SL263369.1	44418	yes	No				virulent/lytic	Yes	2.0	0.0

MSP1515	haib17KIU4866_H7HJMCCXY_SL272745.79	44392	yes	No	Xanthomonas	None		virulent/lytic	Yes	13.0	0.0
MSP1516	haib17CEM4890_H2NYMCCXY_SL254779.28	44381	yes	No	Staphylococcus	Staphylococcus_aureus	Clostridioides difficile	prophage/temperate/lysogenic	Yes	32.0	0.0
MSP1517	haib17KIU4866_H7HJMCCXY_SL272759.75	44348	no	No			Acinetobacter ursingii	virulent/lytic	Yes	21.0	0.0
MSP1521	haib17CEM4890_H7KYMCCXY_SL273060.62	44310	no	No	Streptomyces	Streptomyces_platensis		virulent/lytic	Yes	26.0	0.0
MSP1522	haib17KIU4866_H7HJMCCXY_SL272778.162	44303	yes	No			Rhodospirillum rubrum ATCC 11170	virulent/lytic	Yes	22.0	0.0
MSP1526	haib17CEM4890_H7KYMCCXY_SL273060.63	44277	no	No	Streptomyces	Streptomyces_platensis		virulent/lytic	Yes	32.0	0.0
MSP1527	haib17KIU4866_H7HJMCCXY_SL272796.209	44248	no	Yes				virulent/lytic	No	32.0	0.0
MSP1530	haib17CEM4890_HKC32ALXX_SL254706.396	44221	no	No	Pseudomonas	Pseudomonas_putida	Pseudomonas stutzeri	prophage/temperate/lysogenic	Yes	23.0	0.0
MSP1531	haib17DB4959_H3MGVCCXY_SL259912.9	44197	yes	No				virulent/lytic	Yes	5.0	0.0
MSP1532	haib17CEM4890_HKC32ALXX_SL254693.220	44191	no	Yes				prophage/temperate/lysogenic	Yes	14.0	0.0
MSP1533	haib17CEM4890_H2NYMCCXY_SL254832.68	44190	no	No			Acinetobacter sp. ACNIH1	prophage/temperate/lysogenic	Yes	30.0	0.0
MSP1534	haib17CEM4890_H2NYMCCXY_SL254769.326	44186	no	No			Kosakonia cowanii JCM 10956 = DSM 18146	prophage/temperate/lysogenic	Yes	17.0	0.0
MSP1535	haib17KIU4866_H7HJMCCXY_SL272788.91	44184	no	Yes				prophage/temperate/lysogenic	Yes	27.0	0.0
MSP1537	haib17CEM5106_HCV72CCXY_SL269750.141	44171	no	Yes				prophage/temperate/lysogenic	Yes	33.0	0.0
MSP1538	haib17CEM4890_HMCMJCCXY_SL335851.11	44137	no	No			Acinetobacter sp. LoGeW2-3	prophage/temperate/lysogenic	Yes	30.0	0.0
MSP1539	haib17CEM5106_HCY5HCCXY_SL270973.56	44120	no	Yes			Dickeya zeae	prophage/temperate/lysogenic	No	21.0	0.0
MSP1540	haib17CEM4890_H2NYMCCXY_SL254796.346	44097	no	Yes				prophage/temperate/lysogenic	Yes	12.0	0.0
MSP1541	haib17CEM5106_HCY5JCCXY_SL269571.124	44092	no	Yes				prophage/temperate/lysogenic	Yes	18.0	0.0
MSP1542	haib17CEM4890_HKC32ALXX_SL254765.200	44088	yes	No			Salmonella enterica subsp. enterica serovar Bergen str. ST350	virulent/lytic	Yes	26.0	1.0
MSP1543	haib18CEM5453_HMGW3CCXY_SL342298.1	44086	yes	No				virulent/lytic	Yes	19.0	0.0
MSP1545	haib17DB4959_H3MGVCCXY_SL259967.35	44049	no	Yes	Staphylococcus	Staphylococcus_epidermidis		prophage/temperate/lysogenic	No	27.0	1.0
MSP1546	haib17CEM4890_HKC32ALXX_SL254676.68	44024	no	No				virulent/lytic	Yes	22.0	0.0
MSP1547	haib17CEM4890_H2NYMCCXY_SL254829.318	44019	no	Yes				virulent/lytic	Yes	9.0	0.0
MSP1548	haib17KIU4866_H7HJMCCXY_SL272751.109	44014	no	No				prophage/temperate/lysogenic	Yes	2.0	0.0
MSP1549	haib17KIU4866_H7HJMCCXY_SL272796.210	44009	no	No				prophage/temperate/lysogenic	Yes	31.0	0.0
MSP1551	haib17CEM5106_HCY5HCCXY_SL270959.311	43984	no	Yes			Acidipropionibacterium virtanenii	virulent/lytic	Yes	32.0	0.0
MSP1553	haib17CEM5106_HCVMTCCXY_SL269526.17	43973	no	Yes				prophage/temperate/lysogenic	Yes	18.0	0.0
MSP1554	haib17KIU4866_H7HJMCCXY_SL272747.82	43962	no	Yes	Escherichia	Escherichia_coli	Dickeya zeae NCPPB 3532	prophage/temperate/lysogenic	No	31.0	0.0
MSP1556	haib17CEM5106_HCY5HCCXY_SL270948.140	43909	no	Yes				prophage/temperate/lysogenic	Yes	30.0	0.0
MSP1557	haib17CEM4890_H2NYMCCXY_SL254796.352	43899	no	Yes				prophage/temperate/lysogenic	Yes	13.0	0.0
MSP1558	haib17CEM4890_H2NYMCCXY_SL254783.486	43883	no	No				virulent/lytic	Yes	11.0	0.0
MSP1559	haib17CEM4890_H2NYMCCXY_SL254855.306	43880	no	No				prophage/temperate/lysogenic	Yes	9.0	0.0
MSP1560	haib17CEM5106_HCV72CCXY_SL269777.219	43879	no	No	Gordonia	Gordonia_terrae		virulent/lytic	Yes	21.0	0.0
MSP1561	haib17CEM4890_H2NYMCCXY_SL254772.426	43843	no	Yes			Desulfotomaculum ferrireducens	prophage/temperate/lysogenic	Yes	6.0	0.0
MSP1562	haib17CEM4890_H2NYMCCXY_SL254814.146	43836	no	No				prophage/temperate/lysogenic	Yes	23.0	0.0
MSP1563	haib17CEM5106_HCCGHCCXY_SL270293.2	43827	no	No				prophage/temperate/lysogenic	Yes	23.0	0.0
MSP1564	haib17CEM5241_HMGMHCCXY_SL343449.11	43799	yes	No				virulent/lytic	Yes	16.0	0.0
MSP1565	haib17CEM5106_HCV72CCXY_SL269776.154	43788	no	Yes			Psychrobacter sp. P11F6	prophage/temperate/lysogenic	Yes	19.0	0.0
MSP1566	haib17CEM4890_H2NYMCCXY_SL254847.58	43783	no	No			Actinomyces sp. oral taxon 414	virulent/lytic	No	23.0	0.0
MSP1567	haib17CEM4890_H2NYMCCXY_SL254814.150	43762	no	No				prophage/temperate/lysogenic	Yes	33.0	0.0
MSP1568	haib17CEM4890_HKC32ALXX_SL254764.303	43750	no	No				virulent/lytic	Yes	28.0	0.0
MSP1569	haib18CEM5453_HMC2KCCXY_SL336679.9	43746	no	No				virulent/lytic	Yes	14.0	0.0
MSP1570	haib17CEM4890_HKC32ALXX_SL254722.46	43705	no	No				prophage/temperate/lysogenic	Yes	9.0	0.0
MSP1571	haib17CEM4890_H2NYMCCXY_SL254818.408	43692	no	No			Klebsiella pneumoniae subsp. pneumoniae	prophage/temperate/lysogenic	Yes	15.0	1.0
MSP1572	haib17CEM4890_H2NYMCCXY_SL254810.48	43666	no	No	Staphylococcus	Staphylococcus_aureus	Staphylococcus epidermidis	prophage/temperate/lysogenic	No	28.0	0.0
MSP1573	haib17CEM4890_H2NYMCCXY_SL254858.26	43665	yes	No				prophage/temperate/lysogenic	Yes	6.0	0.0
MSP1574	haib17CEM4890_H2NYMCCXY_SL254807.255	43625	no	No			Kosakonia cowanii JCM 10956 = DSM 18146	prophage/temperate/lysogenic	No	24.0	0.0
MSP1575	haib17CEM4890_H2NYMCCXY_SL254796.358	43623	no	Yes			Acinetobacter baumannii	prophage/temperate/lysogenic	Yes	28.0	1.0
MSP1576	haib17KIU4866_HMCMJCCXY_SL335936.96	43621	yes	Yes				prophage/temperate/lysogenic	No	18.0	0.0
MSP1577	haib17CEM5106_HCY5JCCXY_SL269554.74	43614	no	No				virulent/lytic	Yes	27.0	0.0
MSP1578	haib17CEM5106_HCV72CCXY_SL269748.339	43593	no	Yes	Pseudomonas	Pseudomonas_aeruginosa		prophage/temperate/lysogenic	Yes	32.0	1.0
MSP1579	haib17CEM4890_HKC32ALXX_SL254681.373	43574	no	Yes	Faecalibacterium	Faecalibacterium_prausnitzii		prophage/temperate/lysogenic	Yes	8.0	0.0
MSP1580	haib17CEM4890_HKC32ALXX_SL254764.304	43541	no	No			Fusobacterium nucleatum subsp. polymorphum	prophage/temperate/lysogenic	Yes	13.0	0.0
MSP1581	haib17KIU4866_H7HJMCCXY_SL272751.112	43501	no	No			Acinetobacter sp. LoGeW2-3	prophage/temperate/lysogenic	Yes	25.0	0.0
MSP1582	haib17CEM5080_H7VL7CCXY_SL267266.56	43493	no	Yes			Enterococcus faecalis	prophage/temperate/lysogenic	No	24.0	1.0
MSP1583	haib18CEM5332_HK7G5CCXY_SL310981.4	43474	no	No				virulent/lytic	Yes	24.0	0.0
MSP1584	haib18CEM5453_HMGW3CCXY_SL342609.169	43467	no	No				virulent/lytic	Yes	4.0	0.0
MSP1585	haib17CEM4890_HKC32ALXX_SL254722.47	43465	no	No				virulent/lytic	Yes	28.0	0.0
MSP1586	haib18CEM5453_HMC2KCCXY_SL336627.82	43450	no	No				prophage/temperate/lysogenic	Yes	22.0	0.0
MSP1587	haib17KIU4866_H7HJMCCXY_SL272807.43	43449	no	No			Atlantibacter hermannii	virulent/lytic	Yes	15.0	0.0
MSP1588	haib17KIU4866_HMCMJCCXY_SL335961.32	43435	no	Yes				prophage/temperate/lysogenic	Yes	31.0	0.0
MSP1589	haib17KIU4866_H7HJMCCXY_SL272790.58	43427	no	Yes			Pseudomonas stutzeri	prophage/temperate/lysogenic	Yes	28.0	1.0
MSP1590	haib17CEM4890_H75CGCCXY_SL263714.132	43415	no	Yes				prophage/temperate/lysogenic	Yes	23.0	0.0
MSP1591	haib17CEM4890_H75CGCCXY_SL263638.41	43391	no	Yes			Desulfovibrio desulfuricans ND132	prophage/temperate/lysogenic	Yes	20.0	0.0
MSP1592	haib17CEM4890_H2NYMCCXY_SL254837.43	43373	no	Yes			Pseudomonas balearica DSM 6083	prophage/temperate/lysogenic	Yes	33.0	1.0
MSP1593	haib17KIU4866_H7HJMCCXY_SL272759.78	43360	no	Yes				prophage/temperate/lysogenic	Yes	9.0	0.0
MSP1594	haib18CEM5453_HMGW3CCXY_SL342540.6	43349	no	No			Thermaerobacter sp. FW80	virulent/lytic	Yes	33.0	0.0
MSP1595	haib17CEM5106_HCV72CCXY_SL269761.109	43334	yes	No			Xanthomonas campestris pv. raphani 756C	prophage/temperate/lysogenic	Yes	23.0	1.0

MSP1597	haib17CEM5106_HCY5HCCXY_SL270948.146	43279	no	Yes			Pseudomonas aeruginosa SCV20265	virulent/lytic	Yes	29.0	1.0
MSP1598	haib17CEM5106_HCV72CCXY_SL269717.113	43266	no	Yes			Pseudomonas balearica DSM 6083	prophage/temperate/lysogenic	Yes	29.0	1.0
MSP1599	haib17CEM4890_H2NYMCCXY_SL254771.320	43252	no	No				prophage/temperate/lysogenic	Yes	32.0	0.0
MSP1600	haib17CEM5106_HCV72CCXY_SL269754.302	43244	no	No				prophage/temperate/lysogenic	Yes	10.0	0.0
MSP1602	haib17CEM4890_H2NYMCCXY_SL254818.418	43239	no	Yes			Caldicellulosiruptor kronotskyensis 2002	prophage/temperate/lysogenic	Yes	8.0	0.0
MSP1601	haib17CEM5106_HCY5HCCXY_SL270945.91	43239	no	Yes			Pseudomonas chlororaphis	prophage/temperate/lysogenic	Yes	22.0	0.0
MSP1604	haib17CEM4890_H2NYMCCXY_SL254804.50	43236	no	Yes				virulent/lytic	Yes	13.0	0.0
MSP1603	haib18CEM5453_HMC2KCCXY_SL336697.36	43236	no	No				virulent/lytic	Yes	28.0	0.0
MSP1605	haib17CEM4890_H2NYMCCXY_SL254818.419	43212	no	No				prophage/temperate/lysogenic	Yes	10.0	0.0
MSP1606	haib17CEM4890_H2NYMCCXY_SL254854.237	43192	no	No			Pseudomonas balearica DSM 6083	prophage/temperate/lysogenic	Yes	21.0	1.0
MSP1607	haib17CEM4890_HKC32ALXX_SL254681.374	43187	no	No				prophage/temperate/lysogenic	Yes	13.0	0.0
MSP1608	haib17CEM4890_H2NYMCCXY_SL254781.494	43179	no	No				prophage/temperate/lysogenic	Yes	7.0	0.0
MSP1609	haib17CEM5241_HMGMHCCXY_SL343449.12	43151	no	No			Klebsiella variicola	virulent/lytic	Yes	14.0	0.0
MSP1610	haib17CEM4890_HKC32ALXX_SL254723.4	43140	no	No	Staphylococcus	Staphylococcus_aureus	Staphylococcus epidermidis	prophage/temperate/lysogenic	No	27.0	0.0
MSP1611	haib17CEM5106_HCVMTCCXY_SL269554.57	43138	no	No			Chlorobium limicola DSM 245	virulent/lytic	Yes	6.0	0.0
MSP1612	haib18CEM5453_HMC2KCCXY_SL336650.34	43135	no	No				virulent/lytic	Yes	33.0	0.0
MSP1613	haib17CEM5106_HCCGHCCXY_SL270411.20	43132	no	No				virulent/lytic	Yes	30.0	0.0
MSP1614	haib17CEM4890_H2NYMCCXY_SL254794.631	43097	no	No			Klebsiella pneumoniae	prophage/temperate/lysogenic	Yes	22.0	1.0
MSP1615	haib17KIU4866_H7HJMCCXY_SL272830.36	43084	yes	No			Xanthomonas campestris pv. raphani 756C	virulent/lytic	Yes	21.0	1.0
MSP1616	haib17CEM4890_H2NYMCCXY_SL254806.394	43071	yes	No			Pseudomonas stutzeri	prophage/temperate/lysogenic	Yes	19.0	1.0
MSP1617	haib18CEM5453_HNHKFCCXY_SL345575.26	43070	no	Yes				virulent/lytic	Yes	2.0	0.0
MSP1618	haib17CEM4890_H3KHWCCXY_SL263364.2	43062	no	No				virulent/lytic	Yes	4.0	0.0
MSP1619	haib17CEM4890_HKC32ALXX_SL254747.69	43051	no	No			Cutibacterium granulosum	prophage/temperate/lysogenic	No	32.0	1.0
MSP1620	haib17CEM5106_HCV72CCXY_SL269747.69	43048	no	Yes				prophage/temperate/lysogenic	Yes	24.0	0.0
MSP1621	haib17CEM5106_HCCGHCCXY_SL270547.1	43027	yes	No			Micrococcus luteus	prophage/temperate/lysogenic	Yes	29.0	1.0
MSP1622	haib17CEM4890_HKC32ALXX_SL254680.299	43026	no	No				prophage/temperate/lysogenic	Yes	22.0	0.0
MSP1623	haib17CEM4890_HKC32ALXX_SL254718.266	43023	no	No				prophage/temperate/lysogenic	Yes	12.0	0.0
MSP1624	haib17CEM4890_H2NYMCCXY_SL254784.607	43002	no	Yes	Faecalibacterium	Faecalibacterium_prausnitzii	Desulfofundulus kuznetsovii DSM 6115	virulent/lytic	Yes	9.0	0.0
MSP1625	haib17CEM4890_HMCMJCCXY_SL335773.49	42969	no	No	Staphylococcus	Staphylococcus_aureus		prophage/temperate/lysogenic	No	23.0	1.0
MSP1626	haib17CEM5106_HCY5HCCXY_SL270924.102	42955	no	Yes				virulent/lytic	Yes	32.0	0.0
MSP1627	haib17CEM5106_HCV72CCXY_SL269773.182	42941	no	Yes			Enterobacter hormaechei subsp. oharae	prophage/temperate/lysogenic	No	33.0	0.0
MSP1629	haib17CEM4890_HKC32ALXX_SL254677.9	42931	no	Yes				virulent/lytic	Yes	23.0	0.0
MSP1630	haib17CEM4890_H2NYMCCXY_SL254793.240	42930	no	No				prophage/temperate/lysogenic	Yes	12.0	0.0
MSP1631	haib17CEM5106_HCY5HCCXY_SL270932.76	42917	no	No				prophage/temperate/lysogenic	Yes	26.0	0.0
MSP1632	haib17CEM4890_H2NYMCCXY_SL254857.9	42893	yes	No			Xenorhabdus doucetiae	prophage/temperate/lysogenic	No	19.0	0.0
MSP1633	haib17CEM5106_HCY5JCCXY_SL269596.176	42879	no	Yes				prophage/temperate/lysogenic	No	27.0	0.0
MSP1634	haib17CEM4890_H2NYMCCXY_SL254830.397	42877	no	Yes	Enterobacter	None	Klebsiella pneumoniae	prophage/temperate/lysogenic	No	25.0	0.0
MSP1635	haib17KIU4866_H7HJMCCXY_SL272787.39	42864	no	No	Bacillus	None		prophage/temperate/lysogenic	Yes	16.0	0.0
MSP1636	haib17KIU4866_HMCMJCCXY_SL335940.102	42844	no	Yes			Aquitalea magnusonii	virulent/lytic	Yes	26.0	0.0
MSP1637	haib17KIU4866_HMCMJCCXY_SL335954.28	42842	no	No			Pectobacterium versatile	prophage/temperate/lysogenic	Yes	16.0	0.0
MSP1638	haib18CEM5453_HMGW3CCXY_SL342530.13	42837	no	No				virulent/lytic	Yes	32.0	0.0
MSP1639	haib17CEM5106_HCY5JCCXY_SL269596.177	42825	yes	No				prophage/temperate/lysogenic	Yes	14.0	0.0
MSP1642	haib17KIU4866_H7HJMCCXY_SL272773.205	42805	yes	No				prophage/temperate/lysogenic	Yes	9.0	0.0
MSP1644	haib17CEM4890_H2NYMCCXY_SL254783.499	42781	no	No				prophage/temperate/lysogenic	Yes	7.0	0.0
MSP1645	haib17CEM4890_H75CGCCXY_SL263686.42	42772	no	Yes			Acinetobacter sp. WCHA45	prophage/temperate/lysogenic	Yes	28.0	0.0
MSP1646	haib17CEM5106_HCY5JCCXY_SL269565.52	42761	no	Yes				prophage/temperate/lysogenic	Yes	29.0	0.0
MSP1647	haib18CEM5453_HMGW3CCXY_SL342282.4	42752	yes	No				virulent/lytic	Yes	7.0	0.0
MSP1649	haib17CEM4890_H75CGCCXY_SL263689.146	42750	no	Yes				prophage/temperate/lysogenic	Yes	27.0	0.0
MSP1650	haib18CEM5453_HMGW3CCXY_SL342295.6	42735	no	No	Propionibacterium	Propionibacterium_freudenreichii		virulent/lytic	Yes	31.0	0.0
MSP1651	haib17CEM4890_H2NYMCCXY_SL254781.500	42714	no	Yes				prophage/temperate/lysogenic	Yes	14.0	0.0
MSP1652	haib17CEM4890_H75CGCCXY_SL263641.151	42698	no	Yes			Dickeya sp. CSL RW240	virulent/lytic	Yes	30.0	0.0
MSP1653	haib17KIU4866_H7HJMCCXY_SL272792.38	42667	no	Yes			Dickeya chrysanthemi Ech1591	virulent/lytic	No	31.0	0.0
MSP1654	haib17KIU4866_H7HJMCCXY_SL272754.28	42648	no	No			Acinetobacter radioresistens	prophage/temperate/lysogenic	Yes	28.0	0.0
MSP1657	haib17CEM4890_H2NYMCCXY_SL254784.618	42589	yes	No				virulent/lytic	No	14.0	0.0
MSP1659	haib17CEM4890_HKC32ALXX_SL254763.120	42585	no	Yes				prophage/temperate/lysogenic	Yes	13.0	0.0
MSP1660	haib17CEM4890_H2NYMCCXY_SL254825.28	42579	no	No				virulent/lytic	Yes	12.0	0.0
MSP1661	haib17CEM4890_H2NYMCCXY_SL254770.328	42578	no	Yes			Cedecea lapagei	prophage/temperate/lysogenic	No	21.0	0.0
MSP1662	haib17DB4959_H3MGVCCXY_SL259989.58	42566	no	No	Staphylococcus	Staphylococcus_epidermidis		prophage/temperate/lysogenic	No	29.0	1.0
MSP1664	haib17CEM5106_HCV72CCXY_SL269798.188	42553	no	No				prophage/temperate/lysogenic	Yes	31.0	0.0
MSP1666	haib17CEM4890_HKC32ALXX_SL254749.7	42540	yes	No			Propionibacterium australiense	prophage/temperate/lysogenic	No	26.0	0.0
MSP1667	haib18CEM5453_HNHKFCCXY_SL345538.111	42522	no	No				prophage/temperate/lysogenic	Yes	13.0	0.0
MSP1668	haib17CEM5106_HCV72CCXY_SL269757.99	42521	no	Yes				virulent/lytic	Yes	29.0	0.0
MSP1669	haib17CEM4890_H2NYMCCXY_SL254783.501	42519	no	No				virulent/lytic	Yes	11.0	0.0
MSP1670	haib17CEM5106_HCCGHCCXY_SL270289.1	42491	no	No				virulent/lytic	Yes	19.0	0.0
MSP1671	haib17CEM5106_HCV72CCXY_SL269806.166	42488	no	No	Gordonia	Gordonia_terrae	Propionibacterium australiense	prophage/temperate/lysogenic	Yes	21.0	0.0
MSP1672	haib17CEM4890_HKC32ALXX_SL254763.121	42482	no	No				prophage/temperate/lysogenic	Yes	22.0	0.0
MSP1673	haib18CEM5453_HMC2KCCXY_SL336764.29	42477	no	No				prophage/temperate/lysogenic	Yes	33.0	0.0
MSP1674	haib17CEM4890_HKC32ALXX_SL254705.336	42471	yes	No	Faecalibacterium	Faecalibacterium_prausnitzii		prophage/temperate/lysogenic	Yes	11.0	0.0

MSP1676	haib17CEM4890_H2NYMCCXY_SL254807.263	42465	no	No				prophage/temperate/lysogenic	Yes	4.0	0.0
MSP1677	haib17KIU4866_H7HJMCCXY_SL272759.79	42458	no	Yes				prophage/temperate/lysogenic	Yes	7.0	0.0
MSP1678	haib17CEM5106_HCY5HCCXY_SL270917.263	42452	no	No				virulent/lytic	Yes	21.0	0.0
MSP1680	haib17CEM4890_HKC32ALXX_SL254764.312	42445	no	No			Pseudomonas balearica DSM 6083	prophage/temperate/lysogenic	Yes	19.0	1.0
MSP1681	haib17CEM4890_H2NYMCCXY_SL254843.572	42431	no	Yes				prophage/temperate/lysogenic	Yes	12.0	0.0
MSP1682	haib17CEM4890_HKC32ALXX_SL254685.6	42430	yes	No				virulent/lytic	Yes	21.0	0.0
MSP1683	haib17CEM4890_H2NYMCCXY_SL254827.40	42429	yes	No	Arthrobacter	Arthrobacter_globiformis		virulent/lytic	Yes	27.0	0.0
MSP1684	haib17CEM4890_HKC32ALXX_SL254705.338	42428	no	No			Bacillus pseudomycoides DSM 12442	prophage/temperate/lysogenic	Yes	19.0	0.0
MSP1685	haib17CEM4890_H2NYMCCXY_SL254859.4	42386	yes	No			Actinomyces qiguomingii	virulent/lytic	Yes	24.0	0.0
MSP1686	haib17CEM4890_HKC32ALXX_SL254728.358	42371	no	Yes			Clostridium chauvoei	prophage/temperate/lysogenic	Yes	32.0	0.0
MSP1687	haib17CEM4890_H2NYMCCXY_SL254838.110	42335	no	Yes			Serratia plymuthica AS9	prophage/temperate/lysogenic	Yes	27.0	0.0
MSP1689	haib17CEM4890_H2NYMCCXY_SL254781.502	42310	no	No				virulent/lytic	Yes	11.0	0.0
MSP1691	haib17KIU4866_H7HJMCCXY_SL272827.113	42292	no	No				prophage/temperate/lysogenic	Yes	31.0	0.0
MSP1692	haib17CEM4890_HMCMJCCXY_SL335833.200	42261	no	Yes				virulent/lytic	Yes	15.0	0.0
MSP1693	haib17CEM4890_H75CGCCXY_SL263724.109	42260	no	Yes			Acinetobacter radioresistens	prophage/temperate/lysogenic	Yes	30.0	0.0
MSP1694	haib17CEM5106_HCY5JCCXY_SL269586.81	42234	no	Yes			Pseudomonas aeruginosa SCV20265	prophage/temperate/lysogenic	Yes	30.0	1.0
MSP1695	haib17CEM4890_H2NYMCCXY_SL254818.438	42231	no	Yes				virulent/lytic	Yes	2.0	0.0
MSP1696	haib17CEM4890_H75CGCCXY_SL263700.166	42218	no	No				virulent/lytic	Yes	33.0	0.0
MSP1697	haib17CEM5106_HCY5HCCXY_SL271153.2	42181	no	Yes				virulent/lytic	Yes	21.0	0.0
MSP1698	haib17KIU4866_H7HJMCCXY_SL272742.15	42176	no	Yes			Kosakonia cowanii JCM 10956 = DSM 18146	prophage/temperate/lysogenic	No	28.0	0.0
MSP1699	haib17CEM4890_H7KYMCCXY_SL273067.63	42174	no	No	Staphylococcus	Staphylococcus_aureus		prophage/temperate/lysogenic	No	26.0	1.0
MSP1700	haib17CEM5106_HCY5HCCXY_SL270906.101	42170	no	No				virulent/lytic	Yes	24.0	0.0
MSP1701	haib17DB4959_H3MGVCCXY_SL259838.43	42164	yes	No				prophage/temperate/lysogenic	Yes	24.0	0.0
MSP1702	haib17CEM4890_HKC32ALXX_SL254728.361	42151	no	Yes				virulent/lytic	Yes	14.0	0.0
MSP1703	haib17CEM4890_H2NYMCCXY_SL254795.480	42140	yes	No				virulent/lytic	Yes	10.0	0.0
MSP1704	haib17CEM5106_HCY5HCCXY_SL270946.26	42138	no	No				virulent/lytic	Yes	16.0	0.0
MSP1705	haib17CEM4890_H2NYMCCXY_SL254852.154	42121	no	No			Staphylococcus pseudintermedius	prophage/temperate/lysogenic	Yes	18.0	0.0
MSP1706	haib17KIU4866_H7HJMCCXY_SL272821.74	42117	no	Yes			Pseudomonas stutzeri A1501	prophage/temperate/lysogenic	Yes	18.0	1.0
MSP1708	haib17KIU4866_HMCMJCCXY_SL335963.88	42096	yes	No			Acinetobacter radioresistens	prophage/temperate/lysogenic	Yes	31.0	0.0
MSP1709	haib18CEM5332_HK7G5CCXY_SL310952.4	42077	no	Yes				prophage/temperate/lysogenic	Yes	33.0	0.0
MSP1710	haib17CEM5106_HCV72CCXY_SL269747.72	42076	no	Yes				prophage/temperate/lysogenic	Yes	20.0	0.0
MSP1711	haib17CEM4890_HKC32ALXX_SL254757.3	42065	no	No				prophage/temperate/lysogenic	Yes	8.0	0.0
MSP1712	haib18CEM5332_HK7G5CCXY_SL311016.57	42044	no	Yes				virulent/lytic	Yes	31.0	0.0
MSP1713	haib17CEM5106_HCVMTCCXY_SL269529.50	42040	no	No				prophage/temperate/lysogenic	Yes	14.0	0.0
MSP1714	haib17CEM4890_H2NYMCCXY_SL254828.116	42038	yes	No	Staphylococcus	Staphylococcus_epidermidis		prophage/temperate/lysogenic	Yes	26.0	1.0
MSP1715	haib17KIU4866_H7HJMCCXY_SL272792.39	42008	no	No				virulent/lytic	Yes	20.0	0.0
MSP1716	haib17CEM4890_H2NYMCCXY_SL254795.482	41963	no	Yes			Desulfovibrio desulfuricans ND132	prophage/temperate/lysogenic	Yes	29.0	0.0
MSP1717	haib17CEM4890_H2NYMCCXY_SL254855.327	41954	no	No				prophage/temperate/lysogenic	Yes	20.0	0.0
MSP1718	haib17CEM4890_HKC32ALXX_SL254694.160	41952	no	No	Bacillus	Bacillus_anthracis		prophage/temperate/lysogenic	Yes	12.0	0.0
MSP1719	haib17CEM4890_H2NYMCCXY_SL254830.406	41946	no	Yes			Salmonella enterica subsp. salamae	virulent/lytic	Yes	22.0	1.0
MSP1720	haib17CEM4890_H2NYMCCXY_SL254777.6	41939	yes	No	Staphylococcus	Staphylococcus_epidermidis		prophage/temperate/lysogenic	Yes	18.0	1.0
MSP1721	haib17CEM4890_H2NYMCCXY_SL254827.42	41935	yes	No				prophage/temperate/lysogenic	Yes	20.0	0.0
MSP1722	haib17CEM4890_HKC32ALXX_SL254742.403	41933	no	Yes				prophage/temperate/lysogenic	Yes	11.0	0.0
MSP1723	haib17KIU4866_H7HJMCCXY_SL272822.93	41924	no	No			Kosakonia cowanii JCM 10956 = DSM 18146	virulent/lytic	Yes	19.0	0.0
MSP1724	haib17CEM4890_H2NYMCCXY_SL254860.46	41916	yes	No				prophage/temperate/lysogenic	Yes	19.0	0.0
MSP1725	haib17CEM4890_H2NYMCCXY_SL254846.65	41915	no	No				prophage/temperate/lysogenic	Yes	33.0	0.0
MSP1726	haib17CEM4890_H2NYMCCXY_SL254818.445	41912	no	No				virulent/lytic	Yes	8.0	0.0
MSP1727	haib17KIU4866_H7HJMCCXY_SL272752.101	41904	no	No				virulent/lytic	Yes	19.0	0.0
MSP1728	haib17CEM4890_HKC32ALXX_SL254758.11	41901	yes	No			Microbacterium hominis	virulent/lytic	Yes	17.0	0.0
MSP1729	haib17CEM4890_H2NYMCCXY_SL254824.56	41887	no	No				prophage/temperate/lysogenic	Yes	10.0	0.0
MSP1730	haib17CEM4890_HKC32ALXX_SL254697.7	41880	no	No				prophage/temperate/lysogenic	Yes	4.0	0.0
MSP1731	haib17CEM4890_H2NYMCCXY_SL254825.31	41877	yes	No				prophage/temperate/lysogenic	Yes	7.0	0.0
MSP1732	haib17CEM4890_HKC32ALXX_SL254764.319	41876	yes	No				prophage/temperate/lysogenic	Yes	9.0	0.0
MSP1733	haib18CEM5332_HK7G5CCXY_SL311009.21	41873	yes	No				prophage/temperate/lysogenic	Yes	4.0	0.0
MSP1735	haib18CEM5453_HNHKFCCXY_SL346109.37	41872	no	No				virulent/lytic	Yes	31.0	0.0
MSP1734	haib18CEM5453_HT5YVCCXY_SL357298.1	41872	yes	No				virulent/lytic	Yes	22.0	0.0
MSP1736	haib17CEM5106_HCY5HCCXY_SL270948.155	41870	no	Yes			Azospirillum ramasamyi	prophage/temperate/lysogenic	Yes	28.0	0.0
MSP1737	haib17CEM4890_HKC32ALXX_SL254680.311	41865	no	No			Klebsiella pneumoniae subsp. pneumoniae	prophage/temperate/lysogenic	No	26.0	1.0
MSP1738	haib17CEM4890_H2NYMCCXY_SL254805.319	41862	yes	No				virulent/lytic	Yes	11.0	0.0
MSP1740	haib17CEM4890_HKC32ALXX_SL254723.5	41835	no	No				virulent/lytic	Yes	20.0	0.0
MSP1742	haib17CEM4890_H2NYMCCXY_SL254770.344	41804	no	No				prophage/temperate/lysogenic	Yes	7.0	0.0
MSP1743	haib17CEM4890_H75CGCCXY_SL263725.103	41802	no	No			Acinetobacter radioresistens	prophage/temperate/lysogenic	Yes	23.0	0.0
MSP1744	haib17CEM4890_H2NYMCCXY_SL254772.461	41789	no	No				prophage/temperate/lysogenic	Yes	4.0	0.0
MSP1746	haib17KIU4866_HMCMJCCXY_SL335961.33	41750	yes	No			Cedecea lapagei	prophage/temperate/lysogenic	Yes	23.0	0.0
MSP1747	haib17CEM4890_H75CGCCXY_SL263690.78	41729	no	Yes				virulent/lytic	Yes	33.0	0.0
MSP1748	haib17CEM5106_HCY5HCCXY_SL270967.175	41721	no	Yes				prophage/temperate/lysogenic	Yes	23.0	0.0
MSP1749	haib18CEM5453_HMC2KCCXY_SL336697.44	41720	yes	No				prophage/temperate/lysogenic	Yes	9.0	0.0
MSP1750	haib17CEM4890_H2NYMCCXY_SL254850.107	41718	yes	No				prophage/temperate/lysogenic	No	14.0	0.0

MSP1753	haib18CEM5453_HMC2KCCXY_SL336743.62	41715	no	Yes				prophage/temperate/lysogenic	Yes	19.0	0.0
MSP1754	haib17KIU4866_H7HJMCCXY_SL272802.58	41709	no	Yes				prophage/temperate/lysogenic	Yes	8.0	0.0
MSP1755	haib17CEM5106_HCV72CCXY_SL269799.100	41698	no	Yes				prophage/temperate/lysogenic	Yes	27.0	0.0
MSP1756	haib17CEM5106_HCY5JCCXY_SL269559.117	41692	yes	No				virulent/lytic	Yes	4.0	0.0
MSP1757	haib17CEM4890_H75CGCCXY_SL263723.21	41679	yes	No				prophage/temperate/lysogenic	Yes	9.0	0.0
MSP1760	haib17CEM4890_H2NYMCCXY_SL254783.524	41672	no	Yes				prophage/temperate/lysogenic	Yes	3.0	0.0
MSP1761	haib17KIU4866_H7HJMCCXY_SL272741.74	41666	no	Yes			Pluralibacter gergoviae	virulent/lytic	Yes	23.0	0.0
MSP1762	haib17DB4959_H3MGVCCXY_SL259882.57	41664	yes	No				virulent/lytic	Yes	20.0	0.0
MSP1763	haib17CEM5106_HCV72CCXY_SL269733.241	41655	yes	No			Psychrobacter sp. YP14	prophage/temperate/lysogenic	Yes	12.0	0.0
MSP1766	haib17KIU4866_HMCMJCCXY_SL335956.115	41628	no	No				virulent/lytic	Yes	12.0	0.0
MSP1768	haib17CEM5106_HCV72CCXY_SL269789.138	41624	no	Yes				prophage/temperate/lysogenic	Yes	26.0	0.0
MSP1767	haib17CEM5106_HCY5JCCXY_SL269565.54	41624	no	Yes				prophage/temperate/lysogenic	Yes	23.0	0.0
MSP1769	haib18CEM5453_HMC2KCCXY_SL336755.71	41617	no	No				prophage/temperate/lysogenic	Yes	22.0	0.0
MSP1770	haib17KIU4866_H7HJMCCXY_SL272766.31	41604	yes	No				virulent/lytic	No	10.0	0.0
MSP1771	haib17KIU4866_H7HJMCCXY_SL272780.12	41595	no	No			Xanthomonas campestris pv. raphani	prophage/temperate/lysogenic	Yes	25.0	1.0
MSP1772	haib17CEM5106_HCVMTCCXY_SL269548.52	41587	no	Yes				prophage/temperate/lysogenic	Yes	16.0	0.0
MSP1773	haib17CEM4890_H2NYMCCXY_SL254805.324	41580	no	Yes				virulent/lytic	Yes	31.0	0.0
MSP1774	haib17CEM4890_H2NYMCCXY_SL254784.637	41564	yes	No	Pseudomonas	Pseudomonas_putida	Dickeya zeae CSL RW192	prophage/temperate/lysogenic	Yes	11.0	0.0
MSP1776	haib17CEM4890_HKC32ALXX_SL254752.175	41534	no	No				prophage/temperate/lysogenic	Yes	27.0	0.0
MSP1777	haib17CEM4890_H2NYMCCXY_SL254843.581	41527	no	Yes				virulent/lytic	Yes	18.0	0.0
MSP1778	haib17CEM4890_H2NYMCCXY_SL254854.254	41500	no	Yes				prophage/temperate/lysogenic	Yes	23.0	0.0
MSP1779	haib17CEM5106_HCY5HCCXY_SL270945.96	41498	no	Yes				prophage/temperate/lysogenic	Yes	29.0	0.0
MSP1780	haib17CEM4890_H2NYMCCXY_SL254771.340	41492	no	Yes				virulent/lytic	Yes	9.0	0.0
MSP1781	haib17CEM4890_H2NYMCCXY_SL254855.331	41488	no	Yes			Tissierellia sp. JN-28	virulent/lytic	Yes	12.0	0.0
MSP1782	haib18CEM5453_HMGN5CCXY_SL344637.1	41482	no	No			Streptomyces sp. 2323.1	prophage/temperate/lysogenic	Yes	8.0	0.0
MSP1783	haib17KIU4866_H7HJMCCXY_SL272797.19	41465	no	No				virulent/lytic	Yes	10.0	0.0
MSP1784	haib17KIU4866_H7HJMCCXY_SL272773.210	41459	no	No	Enterobacter	None	Methylomicrobium album BG8	prophage/temperate/lysogenic	Yes	14.0	0.0
MSP1785	haib17CEM4890_H2NYMCCXY_SL254809.38	41454	no	No				prophage/temperate/lysogenic	Yes	22.0	0.0
MSP1786	haib18CEM5332_HK7G5CCXY_SL311009.25	41435	yes	No				prophage/temperate/lysogenic	Yes	3.0	0.0
MSP1787	haib17CEM4890_HKC32ALXX_SL254681.390	41417	no	Yes				prophage/temperate/lysogenic	Yes	7.0	0.0
MSP1788	haib17CEM4890_HMCMJCCXY_SL335819.74	41414	no	Yes			Ruminococcus bicirculans	virulent/lytic	Yes	32.0	0.0
MSP1789	haib17CEM5106_HCVMTCCXY_SL269604.100	41393	no	Yes			Dickeya dianthicola RNS04.9	prophage/temperate/lysogenic	Yes	21.0	0.0
MSP1790	haib17CEM4890_H75CGCCXY_SL263721.128	41390	no	No			Citrobacter koseri	prophage/temperate/lysogenic	No	17.0	0.0
MSP1791	haib17CEM4890_H2NYMCCXY_SL254843.582	41380	no	Yes				prophage/temperate/lysogenic	Yes	12.0	0.0
MSP1792	haib17CEM4890_H75CGCCXY_SL263664.108	41364	no	Yes			Xanthomonas campestris pv. raphani 756C	prophage/temperate/lysogenic	Yes	24.0	1.0
MSP1793	haib17CEM5106_HCY5JCCXY_SL269589.64	41360	no	No				prophage/temperate/lysogenic	Yes	32.0	0.0
MSP1796	haib17KIU4866_H7HJMCCXY_SL272803.139	41349	no	No				prophage/temperate/lysogenic	Yes	17.0	0.0
MSP1795	haib18CEM5453_HMGN5CCXY_SL344634.32	41349	yes	No				prophage/temperate/lysogenic	Yes	27.0	0.0
MSP1797	haib17KIU4866_HMCMJCCXY_SL335946.68	41347	no	No				virulent/lytic	Yes	19.0	0.0
MSP1798	haib17CEM5106_HCY5HCCXY_SL270962.101	41336	no	Yes				virulent/lytic	Yes	20.0	0.0
MSP1799	haib17CEM4890_H2NYMCCXY_SL254817.263	41313	no	No			Acinetobacter sp. ACNIH1	prophage/temperate/lysogenic	Yes	26.0	0.0
MSP1800	haib18CEM5453_HNHKFCCXY_SL345980.24	41307	no	No				virulent/lytic	Yes	5.0	0.0
MSP1801	haib17CEM4890_H75CGCCXY_SL263654.90	41303	no	No			Acinetobacter sp. LoGeW2-3	prophage/temperate/lysogenic	Yes	31.0	0.0
MSP1802	haib17CEM4890_HMCMJCCXY_SL335859.19	41256	no	No	Staphylococcus	Staphylococcus_aureus		prophage/temperate/lysogenic	No	22.0	1.0
MSP1803	haib17CEM4890_H2NYMCCXY_SL254843.584	41239	no	Yes				prophage/temperate/lysogenic	Yes	9.0	0.0
MSP1804	haib17CEM4890_HKC32ALXX_SL254718.278	41229	no	No				virulent/lytic	Yes	29.0	0.0
MSP1805	haib17CEM4890_H75CGCCXY_SL263677.140	41223	yes	No				virulent/lytic	Yes	19.0	0.0
MSP1806	haib17KIU4866_HMCMJCCXY_SL335949.125	41207	no	No			Pseudomonas sp. CMR12a	prophage/temperate/lysogenic	Yes	17.0	0.0
MSP1807	haib17CEM5106_HCY5JCCXY_SL269579.61	41202	no	Yes				virulent/lytic	Yes	14.0	0.0
MSP1808	haib17CEM4890_H2NYMCCXY_SL254793.270	41198	yes	No				prophage/temperate/lysogenic	Yes	13.0	0.0
MSP1809	haib17CEM4890_H7KYMCCXY_SL273035.1	41177	no	No				virulent/lytic	Yes	26.0	0.0
MSP1811	haib17KIU4866_H7HJMCCXY_SL272778.184	41174	yes	No			Pseudomonas aeruginosa 19BR	prophage/temperate/lysogenic	Yes	16.0	1.0
MSP1810	haib18CEM5453_HMC2KCCXY_SL336784.7	41174	no	Yes				virulent/lytic	Yes	33.0	0.0
MSP1812	haib17CEM5241_HMGMHCCXY_SL343483.3	41172	yes	No				virulent/lytic	Yes	5.0	0.0
MSP1814	haib17CEM5106_HCV72CCXY_SL269798.198	41151	no	No	Arthrobacter	Arthrobacter_globiformis		virulent/lytic	Yes	23.0	0.0
MSP1815	haib17CEM5106_HCV72CCXY_SL269762.74	41150	no	No			Enterobacter cloacae	virulent/lytic	No	27.0	0.0
MSP1816	haib17KIU4866_HMCMJCCXY_SL335956.116	41124	no	Yes			Moraxella osloensis	prophage/temperate/lysogenic	Yes	30.0	1.0
MSP1819	haib17CEM4890_H2NYMCCXY_SL254771.347	41106	no	Yes			Pseudomonas sp. Leaf58	prophage/temperate/lysogenic	Yes	31.0	0.0
MSP1820	haib17CEM5106_HCV72CCXY_SL269772.110	41084	no	Yes			Acinetobacter baumannii	prophage/temperate/lysogenic	Yes	32.0	1.0
MSP1821	haib17CEM4890_HKC32ALXX_SL254718.279	41068	no	No				virulent/lytic	Yes	31.0	0.0
MSP1822	haib17KIU4866_H7HJMCCXY_SL272809.58	41063	no	Yes			Acinetobacter sp. ACNIH1	prophage/temperate/lysogenic	Yes	29.0	0.0
MSP1823	haib17CEM4890_HKC32ALXX_SL254763.125	41055	no	No	Bacillus	Bacillus_cereus	Aeribacillus pallidus	prophage/temperate/lysogenic	Yes	14.0	0.0
MSP1824	haib17CEM4890_HKC32ALXX_SL254762.63	41035	no	Yes				virulent/lytic	Yes	21.0	0.0
MSP1825	haib17CEM4890_H7KYMCCXY_SL273060.70	41025	yes	No				virulent/lytic	Yes	14.0	0.0
MSP1826	haib17CEM4890_H2NYMCCXY_SL254794.657	41018	no	No				virulent/lytic	Yes	19.0	0.0
MSP1827	haib17KIU4866_H7HJMCCXY_SL272779.27	41013	no	No			Pseudomonas sp. phDV1	prophage/temperate/lysogenic	Yes	19.0	0.0
MSP1828	haib18CEM5453_HNHKFCCXY_SL345590.20	40993	no	Yes				prophage/temperate/lysogenic	Yes	32.0	0.0
MSP1829	haib18CEM5453_HMC2KCCXY_SL336650.36	40989	no	Yes				prophage/temperate/lysogenic	No	29.0	0.0

MSP1830	haib17CEM4890_H2NYMCCXY_SL254843.589	40968	no	No			Pseudomonas aeruginosa	prophage/temperate/lysogenic	Yes	8.0	1.0
MSP1831	haib17CEM5106_HCY5HCCXY_SL270908.49	40956	no	No				prophage/temperate/lysogenic	Yes	16.0	0.0
MSP1832	haib17CEM5106_HCV72CCXY_SL269768.104	40949	no	No				prophage/temperate/lysogenic	Yes	21.0	0.0
MSP1833	haib17CEM4890_H2NYMCCXY_SL254838.117	40938	no	No	Enterococcus	Enterococcus_faecium		virulent/lytic	No	9.0	0.0
MSP1834	haib17DB4959_HMCMJCCXY_SL336180.7	40917	no	No				virulent/lytic	Yes	19.0	0.0
MSP1835	haib17CEM5106_HCV72CCXY_SL269784.111	40916	no	Yes			Bifidobacterium breve	virulent/lytic	Yes	25.0	0.0
MSP1836	haib17KIU4866_HMCMJCCXY_SL335947.139	40914	no	Yes			Pseudomonas aeruginosa	prophage/temperate/lysogenic	Yes	21.0	1.0
MSP1838	haib17CEM4890_H2NYMCCXY_SL254784.646	40897	no	No				virulent/lytic	Yes	28.0	0.0
MSP1839	haib18CEM5332_HMGW3CCXY_SL342430.1	40874	no	No			Roseomonas sp. FDAARGOS_362	virulent/lytic	Yes	33.0	1.0
MSP1840	haib18CEM5453_HMC2KCCXY_SL336626.1	40868	no	No			Klebsiella pneumoniae subsp. pneumoniae	virulent/lytic	No	11.0	1.0
MSP1841	haib17CEM4890_HKC32ALXX_SL254679.70	40865	no	Yes				virulent/lytic	Yes	7.0	0.0
MSP1842	haib17CEM4890_H2NYMCCXY_SL254783.540	40854	no	No				prophage/temperate/lysogenic	Yes	12.0	0.0
MSP1843	haib17CEM4890_HKC32ALXX_SL254698.26	40848	yes	No			Halorussus sp. RC-68	prophage/temperate/lysogenic	Yes	25.0	0.0
MSP1844	haib17CEM4890_HKC32ALXX_SL254684.9	40829	no	No				virulent/lytic	Yes	23.0	0.0
MSP1845	haib17CEM4890_H2NYMCCXY_SL254770.349	40827	no	No				virulent/lytic	Yes	26.0	0.0
MSP1846	haib17DB4959_H3MGVCCXY_SL259882.59	40824	no	No				virulent/lytic	Yes	5.0	0.0
MSP1847	haib18CEM5453_HMGN5CCXY_SL344674.2	40818	no	No				virulent/lytic	Yes	4.0	0.0
MSP1848	haib17CEM4890_H2NYMCCXY_SL254818.469	40802	no	No			Pectobacterium brasiliense	prophage/temperate/lysogenic	No	26.0	0.0
MSP1849	haib17KIU4866_HMCMJCCXY_SL335956.117	40800	no	Yes			Paenibacillus riograndensis SBR5	virulent/lytic	Yes	15.0	0.0
MSP1850	haib17KIU4866_H7HJMCCXY_SL272827.120	40799	no	No			Salmonella enterica subsp. enterica serovar Bergen str. ST350	virulent/lytic	Yes	17.0	1.0
MSP1851	haib17CEM5106_HCV72CCXY_SL269799.104	40797	no	No	Propionibacterium	Propionibacterium_freudenreichii	Corynebacterium humireducens NBRC 106098 = DSM 45392	prophage/temperate/lysogenic	Yes	26.0	0.0
MSP1854	haib17KIU4866_HMCMJCCXY_SL335937.38	40774	yes	No	Enterococcus	Enterococcus_faecalis	Vagococcus penaei	virulent/lytic	Yes	7.0	0.0
MSP1855	haib17CEM4890_H75CGCCXY_SL263689.159	40760	no	Yes				virulent/lytic	Yes	23.0	0.0
MSP1857	haib17CEM5106_HCY5HCCXY_SL270926.21	40755	no	No				virulent/lytic	Yes	31.0	0.0
MSP1858	haib17KIU4866_H7HJMCCXY_SL272804.95	40750	no	No				virulent/lytic	No	26.0	0.0
MSP1860	haib17CEM4890_HKC32ALXX_SL254722.53	40742	yes	No			Actinomyces sp. Chiba101	prophage/temperate/lysogenic	No	11.0	0.0
MSP1861	haib17KIU4866_H7HJMCCXY_SL272764.53	40737	no	Yes				virulent/lytic	Yes	26.0	0.0
MSP1862	haib17CEM4890_H2NYMCCXY_SL254838.118	40712	no	Yes			Pectobacterium brasiliense	virulent/lytic	No	33.0	0.0
MSP1863	haib18CEM5332_HK7G5CCXY_SL311009.26	40676	no	No				virulent/lytic	Yes	27.0	0.0
MSP1864	haib17CEM5106_HCY5JCCXY_SL269573.108	40660	no	Yes	Lactococcus	Lactococcus_garvieae		prophage/temperate/lysogenic	Yes	23.0	0.0
MSP1865	haib17CEM4890_HKC32ALXX_SL254706.435	40641	no	No			Peptoniphilus harei	prophage/temperate/lysogenic	Yes	12.0	0.0
MSP1867	haib17CEM4890_H2NYMCCXY_SL254769.372	40624	yes	No				prophage/temperate/lysogenic	Yes	16.0	0.0
MSP1868	haib17CEM5106_HCV72CCXY_SL269806.180	40596	yes	No	Faecalibacterium	Faecalibacterium_prausnitzii		prophage/temperate/lysogenic	Yes	11.0	0.0
MSP1869	haib17KIU4866_H7HJMCCXY_SL272824.128	40591	no	Yes			Dickeya sp. CSL RW240	virulent/lytic	Yes	28.0	0.0
MSP1870	haib17CEM5106_HCVMTCCXY_SL269603.81	40586	no	Yes				virulent/lytic	Yes	19.0	0.0
MSP1871	haib17CEM4890_H2NYMCCXY_SL254781.517	40584	no	No				prophage/temperate/lysogenic	Yes	10.0	0.0
MSP1872	haib17CEM4890_HKC32ALXX_SL254681.398	40579	no	No			Clostridium botulinum	prophage/temperate/lysogenic	Yes	9.0	0.0
MSP1873	haib17CEM4890_H2NYMCCXY_SL254783.547	40573	no	No				virulent/lytic	No	17.0	0.0
MSP1875	haib17CEM4890_HMCMJCCXY_SL335797.101	40569	no	No				virulent/lytic	Yes	3.0	0.0
MSP1876	haib17CEM4890_HKC32ALXX_SL254705.353	40564	no	No				prophage/temperate/lysogenic	Yes	33.0	0.0
MSP1877	haib17CEM4890_HKC32ALXX_SL254747.73	40558	yes	No				virulent/lytic	Yes	18.0	0.0
MSP1878	haib18CEM5453_HNHKFCCXY_SL345575.30	40552	no	Yes				prophage/temperate/lysogenic	Yes	11.0	0.0
MSP1880	haib17CEM4890_H2NYMCCXY_SL254776.114	40550	no	No			Corynebacterium striatum	prophage/temperate/lysogenic	Yes	32.0	0.0
MSP1879	haib17CEM5106_HCCGHCCXY_SL270293.4	40550	yes	No				virulent/lytic	Yes	5.0	0.0
MSP1881	haib18CEM5453_HMGW3CCXY_SL342294.1	40542	no	No			Klebsiella pneumoniae subsp. pneumoniae	virulent/lytic	Yes	21.0	1.0
MSP1882	haib17CEM4890_HKC32ALXX_SL254717.29	40477	no	Yes			Acinetobacter sp. LoGeW2-3	prophage/temperate/lysogenic	Yes	28.0	0.0
MSP1883	haib17CEM4890_H2NYMCCXY_SL254848.89	40466	no	No			Staphylococcus warneri	prophage/temperate/lysogenic	No	32.0	0.0
MSP1884	haib17CEM5106_HCY5JCCXY_SL269583.79	40464	no	Yes				prophage/temperate/lysogenic	Yes	13.0	0.0
MSP1885	haib17KIU4866_H7HJMCCXY_SL272768.115	40454	no	No				virulent/lytic	Yes	4.0	0.0
MSP1886	haib17CEM4890_H2NYMCCXY_SL254843.593	40448	no	Yes			Paenibacillus yonginensis	virulent/lytic	Yes	9.0	0.0
MSP1887	haib17CEM4890_HKC32ALXX_SL254717.30	40431	no	Yes				prophage/temperate/lysogenic	Yes	20.0	0.0
MSP1889	haib17CEM4890_HKC32ALXX_SL254718.281	40408	no	No				prophage/temperate/lysogenic	Yes	14.0	0.0
MSP1890	haib17CEM4890_H2NYMCCXY_SL254826.250	40403	no	Yes				virulent/lytic	Yes	23.0	0.0
MSP1891	haib17CEM4890_H2NYMCCXY_SL254843.594	40399	no	Yes				prophage/temperate/lysogenic	Yes	14.0	0.0
MSP1892	haib17CEM4890_H2NYMCCXY_SL254805.331	40398	no	Yes	Stenotrophomonas	None	Dickeya chrysanthemi NCPPB 402	prophage/temperate/lysogenic	No	28.0	0.0
MSP1893	haib18CEM5453_HNHKFCCXY_SL345538.133	40390	yes	No				prophage/temperate/lysogenic	Yes	4.0	0.0
MSP1894	haib17DB4959_HMGW3CCXY_SL342470.1	40375	no	No				virulent/lytic	Yes	16.0	0.0
MSP1895	haib17CEM5106_HCCGHCCXY_SL270510.369	40371	no	Yes			Salmonella enterica subsp. diarizonae	virulent/lytic	No	31.0	1.0
MSP1896	haib17CEM4890_HKC32ALXX_SL254706.440	40344	no	Yes				virulent/lytic	Yes	18.0	0.0
MSP1897	haib18CEM5453_HMC2KCCXY_SL336578.17	40305	no	No			Streptomonospora sp. M2	virulent/lytic	Yes	18.0	0.0
MSP1898	haib17CEM4890_H2NYMCCXY_SL254790.123	40291	no	No	Arthrobacter	None		virulent/lytic	Yes	24.0	0.0
MSP1899	haib17CEM4890_HMCMJCCXY_SL335783.85	40277	no	Yes			Acinetobacter baumannii	prophage/temperate/lysogenic	Yes	31.0	1.0
MSP1900	haib17KIU4866_H7HJMCCXY_SL272740.114	40265	no	Yes				prophage/temperate/lysogenic	Yes	14.0	0.0
MSP1901	haib17CEM4890_H2NYMCCXY_SL254818.476	40264	no	No				virulent/lytic	Yes	22.0	0.0
MSP1902	haib17CEM5106_HCY5HCCXY_SL271111.49	40259	no	No			Clostridioides difficile	prophage/temperate/lysogenic	Yes	31.0	0.0
MSP1903	haib17CEM4890_H2NYMCCXY_SL254854.270	40198	no	Yes	Escherichia	None	Atlantibacter hermannii	prophage/temperate/lysogenic	No	16.0	0.0
MSP1904	haib17KIU4866_HMCMJCCXY_SL335888.25	40197	no	Yes			Pseudomonas balearica DSM 6083	virulent/lytic	Yes	30.0	1.0
MSP1905	haib18CEM5453_HNHKFCCXY_SL345930.6	40192	no	No				virulent/lytic	Yes	1.0	0.0

MSP1907	haib17CEM4890_H2NYMCCXY_SL254772.484	40177	no	No				prophage/temperate/lysogenic	Yes	33.0	0.0
MSP1908	haib18CEM5453_HNHKFCCXY_SL346051.5	40149	no	No			Corynebacterium urealyticum DSM 7111	virulent/lytic	Yes	32.0	0.0
MSP1909	haib17KIU4866_HMCMJCCXY_SL335947.142	40138	no	No				virulent/lytic	Yes	18.0	0.0
MSP1910	haib17CEM4890_H2NYMCCXY_SL254783.562	40129	no	Yes				virulent/lytic	Yes	14.0	0.0
MSP1911	haib17KIU4866_H7HJMCCXY_SL272773.215	40115	no	Yes				virulent/lytic	Yes	12.0	0.0
MSP1912	haib17CEM4890_H75CGCCXY_SL263654.94	40111	no	Yes				prophage/temperate/lysogenic	Yes	23.0	0.0
MSP1913	haib17CEM4890_H75CGCCXY_SL263709.58	40110	no	No				prophage/temperate/lysogenic	Yes	21.0	0.0
MSP1914	haib18CEM5453_HMC2KCCXY_SL336638.4	40101	no	No				prophage/temperate/lysogenic	Yes	25.0	0.0
MSP1915	haib17CEM4890_H2NYMCCXY_SL254841.290	40097	no	No				prophage/temperate/lysogenic	Yes	31.0	0.0
MSP1916	haib17CEM5106_HCY5HCCXY_SL270976.242	40096	no	Yes				virulent/lytic	Yes	12.0	0.0
MSP1917	haib17KIU4866_H7HJMCCXY_SL272799.128	40083	no	Yes				prophage/temperate/lysogenic	Yes	14.0	0.0
MSP1918	haib17KIU4866_H7HJMCCXY_SL272773.216	40080	no	No				virulent/lytic	Yes	33.0	0.0
MSP1919	haib17CEM5106_HCV72CCXY_SL269772.115	40063	no	Yes				prophage/temperate/lysogenic	Yes	26.0	0.0
MSP1920	haib17KIU4866_H7HJMCCXY_SL272771.127	40053	no	Yes	Klebsiella	Klebsiella_pneumoniae	Salmonella enterica subsp. enterica serovar Montevideo str.	prophage/temperate/lysogenic	Yes	25.0	0.0
MSP1921	haib17CEM5106_HCY5HCCXY_SL270923.1	40049	no	No			Bacillus coagulans	prophage/temperate/lysogenic	Yes	18.0	0.0
MSP1922	haib17CEM4890_H2NYMCCXY_SL254790.125	40021	no	Yes				virulent/lytic	Yes	24.0	0.0
MSP1923	haib17CEM5106_HCV72CCXY_SL269762.81	40001	no	No			Cedecea lapagei	prophage/temperate/lysogenic	Yes	25.0	0.0

Table S2

Supplementary Table S2. Recovered phages assigned with a putative host by vHULK. The dashed cells present the recovered genomes with predictions only at the genus level.

Genus	Species	Number of PPGs
Acinetobacter	*Acinetobacter baumannii*	7
Enterobacter	----	3
Enterococcus	*Enterococcus faecium*	2
	Enterococcus faecalis	2
Klebsiella	Klebsiella pneumoniae	4
Pseudomonas	----	4
	Pseudomonas aeruginosa	20
	Pseudomonas fluorescens	8
	Pseudomonas putida	2
Staphylococcus	----	1
	Staphylococcus aureus	17
	Staphylococcus epidermidis	12
	Staphylococcus pseudintermedius	1
	Total	83

Table S3

Supplementary Table S3. Rank of PPG hosts predicted by vHULK.

City	Bacteria/ PPG Host	Bacteria abudance rank	Bacteria abundance (%)	PPGs abudance rank	PPGs abundance (%)
auckland	Stenotrophomonas maltophilia	1	0,32824	15	0,08389
auckland	Pseudomonas aeruginosa	3	0,08573	2	0,96212
auckland	Pseudomonas fluorescens	4	0,08441	5	0,30690
auckland	Escherichia coli	7	0,02382	23	0,00100
auckland	Acinetobacter baumannii	10	0,01772	7	0,14387
auckland	Enterococcus faecalis	25	0,00058	21	0,00419
auckland	Staphylococcus aureus	28	0,00034	6	0,21352
auckland	Staphylococcus epidermidis	29	0,00031	4	0,54592
berlin	Stenotrophomonas maltophilia	3	0,40484	20	0,02466
berlin	Staphylococcus epidermidis	4	0,30936	1	2,74046
berlin	Pseudomonas aeruginosa	6	0,09053	3	1,79556
berlin	Staphylococcus aureus	9	0,04986	2	2,07602
berlin	Acinetobacter baumannii	10	0,03613	6	0,57434
berlin	Escherichia coli	12	0,02049	26	0,01138
berlin	Pseudomonas fluorescens	21	0,00954	4	1,58783
berlin	Enterococcus faecalis	26	0,00362	31	0,00498
bogota	Pseudomonas fluorescens	2	0,17466	6	0,63733
bogota	Staphylococcus epidermidis	3	0,11095	1	2,02663
bogota	Acinetobacter baumannii	7	0,03153	3	1,38348
bogota	Pseudomonas aeruginosa	9	0,02862	5	0,67338
bogota	Staphylococcus aureus	11	0,01976	2	1,76053
bogota	Escherichia coli	12	0,01959	21	0,03320
bogota	Stenotrophomonas maltophilia	17	0,01641	28	0,00820
bogota	Enterococcus faecalis	29	0,00495	25	0,01344
cairngorm	Pseudomonas aeruginosa	2	0,22953	4	0,69385
cairngorm	Staphylococcus aureus	5	0,15873	2	1,26327
cairngorm	Enterococcus faecalis	6	0,14260	14	0,09711
cairngorm	Escherichia coli	9	0,09505	16	0,06401
cairngorm	Staphylococcus epidermidis	11	0,02596	1	9,95584
cairngorm	Stenotrophomonas maltophilia	17	0,00716	26	0,00000

Table S3

cairngorm	Acinetobacter baumanni	18	0,00387	17	0,04704
cairngorm	Pseudomonas fluorescens	27	0,00180	5	0,62473
denver	Stenotrophomonas maltophilia	2	0,45928	26	0,00261
denver	Staphylococcus epidermidis	6	0,02133	5	0,22569
denver	Pseudomonas aeruginosa	8	0,01474	8	0,13303
denver	Pseudomonas fluorescens	9	0,01181	6	0,19429
denver	Acinetobacter baumanni	11	0,00956	9	0,11382
denver	Escherichia coli	12	0,00827	22	0,00363
denver	Staphylococcus aureus	22	0,00512	4	0,34066
denver	Enterococcus faecalis	31	0,00132	20	0,00680
doha	Pseudomonas aeruginosa	2	3,99929	1	4,57110
doha	Escherichia coli	4	2,47975	5	0,69663
doha	Staphylococcus aureus	6	0,65281	4	0,91822
doha	Enterococcus faecalis	7	0,17743	14	0,06614
doha	Pseudomonas fluorescens	12	0,03567	3	1,00666
doha	Stenotrophomonas maltophilia	13	0,02550	25	0,00081
doha	Acinetobacter baumanni	15	0,02114	13	0,06673
doha	Staphylococcus epidermidis	17	0,01252	2	2,23113
fairbanks	Staphylococcus epidermidis	1	9,00361	2	4,80617
fairbanks	Staphylococcus aureus	5	0,11906	1	8,01142
fairbanks	Stenotrophomonas maltophilia	9	0,06400	17	0,04035
fairbanks	Escherichia coli	12	0,02799	20	0,01025
fairbanks	Pseudomonas fluorescens	14	0,01791	7	0,26657
fairbanks	Pseudomonas aeruginosa	16	0,01154	9	0,22009
fairbanks	Acinetobacter baumanni	18	0,00979	6	0,29207
fairbanks	Enterococcus faecalis	27	0,00174	23	0,00491
hamilton	Stenotrophomonas maltophilia	1	0,77765	5	0,25532
hamilton	Pseudomonas fluorescens	3	0,20556	2	0,55694
hamilton	Pseudomonas aeruginosa	4	0,08008	1	0,68051
hamilton	Acinetobacter baumanni	5	0,06056	9	0,07794
hamilton	Escherichia coli	7	0,05464	21	0,00183
hamilton	Enterococcus faecalis	12	0,01254	18	0,00788
hamilton	Staphylococcus epidermidis	24	0,00021	10	0,04421
hamilton	Staphylococcus aureus	28	0,00008	13	0,01872

Table S3

hanoi	Escherichia coli	1	1,17647	1	2,57564
hanoi	Acinetobacter baumanni	5	0,06655	5	0,39403
hanoi	Stenotrophomonas maltophilia	7	0,04055	14	0,02761
hanoi	Pseudomonas aeruginosa	10	0,01564	8	0,12179
hanoi	Staphylococcus aureus	12	0,01011	3	0,82786
hanoi	Enterococcus faecalis	14	0,00898	15	0,02269
hanoi	Staphylococcus epidermidis	17	0,00704	4	0,63092
hanoi	Pseudomonas fluorescens	27	0,00167	11	0,06171
hong_kong	Pseudomonas aeruginosa	4	0,07228	6	0,30082
hong_kong	Stenotrophomonas maltophilia	6	0,04472	15	0,01369
hong_kong	Acinetobacter baumanni	8	0,02743	12	0,09828
hong_kong	Staphylococcus epidermidis	16	0,01020	2	0,64528
hong_kong	Escherichia coli	23	0,00485	20	0,00531
hong_kong	Staphylococcus aureus	25	0,00351	5	0,38241
hong_kong	Pseudomonas fluorescens	26	0,00275	7	0,21947
hong_kong	Enterococcus faecalis	28	0,00131	23	0,00306
ilorin	Stenotrophomonas maltophilia	2	0,45615	20	0,02425
ilorin	Escherichia coli	4	0,18761	7	0,20947
ilorin	Pseudomonas aeruginosa	5	0,09112	1	4,89105
ilorin	Acinetobacter baumanni	7	0,06359	8	0,19310
ilorin	Staphylococcus epidermidis	13	0,00675	12	0,10186
ilorin	Pseudomonas fluorescens	16	0,00540	2	2,58153
ilorin	Enterococcus faecalis	17	0,00440	24	0,01292
ilorin	Staphylococcus aureus	26	0,00167	15	0,07690
kuala_lumpur	Pseudomonas aeruginosa	1	43,17741	1	38,35489
kuala_lumpur	Escherichia coli	3	11,57926	2	3,22304
kuala_lumpur	Enterococcus faecalis	6	0,58779	5	0,14180
kuala_lumpur	Staphylococcus aureus	7	0,58672	8	0,02480
kuala_lumpur	Stenotrophomonas maltophilia	14	0,00129	24	0,00000
kuala_lumpur	Pseudomonas fluorescens	17	0,00048	4	0,26736
kuala_lumpur	Staphylococcus epidermidis	23	0,00014	10	0,00890
kuala_lumpur	Acinetobacter baumanni	30	0,00003	21	0,00009
kyiv	Acinetobacter baumanni	4	0,29822	10	0,26061
kyiv	Escherichia coli	6	0,07665	15	0,07762

Table S3

kyiv	Pseudomonas aeruginosa	7	0,06321	14	0,21806
kyiv	Stenotrophomonas maltophilia	9	0,04070	20	0,00492
kyiv	Staphylococcus epidermidis	24	0,00339	2	1,07761
kyiv	Pseudomonas fluorescens	26	0,00298	8	0,31141
kyiv	Enterococcus faecalis	29	0,00179	28	0,00023
kyiv	Staphylococcus aureus	32	0,00095	3	0,54730
lisbon	Stenotrophomonas maltophilia	2	1,47592	21	0,01088
lisbon	Acinetobacter baumannii	3	0,77363	3	1,70853
lisbon	Pseudomonas aeruginosa	6	0,10960	2	2,05801
lisbon	Enterococcus faecalis	7	0,07494	5	0,46307
lisbon	Escherichia coli	8	0,04810	24	0,00497
lisbon	Staphylococcus epidermidis	15	0,01086	9	0,12975
lisbon	Pseudomonas fluorescens	16	0,01068	1	2,23028
lisbon	Staphylococcus aureus	17	0,00582	7	0,31662
london	Pseudomonas aeruginosa	1	5,04627	2	1,28101
london	Staphylococcus aureus	3	3,75576	6	0,68081
london	Enterococcus faecalis	4	3,59397	9	0,32270
london	Escherichia coli	5	3,24318	19	0,10813
london	Stenotrophomonas maltophilia	14	0,18658	22	0,05304
london	Pseudomonas fluorescens	15	0,06375	5	0,90036
london	Staphylococcus epidermidis	19	0,02314	3	1,23459
london	Acinetobacter baumannii	24	0,00409	16	0,16042
marseille	Stenotrophomonas maltophilia	4	0,06283	20	0,01158
marseille	Pseudomonas aeruginosa	6	0,05407	2	0,24244
marseille	Acinetobacter baumannii	9	0,03945	3	0,23120
marseille	Staphylococcus epidermidis	10	0,03041	6	0,12626
marseille	Pseudomonas fluorescens	16	0,01577	7	0,09790
marseille	Escherichia coli	17	0,01542	16	0,02734
marseille	Staphylococcus aureus	27	0,00396	5	0,16116
marseille	Enterococcus faecalis	32	0,00176	23	0,00535
minneapolis	Staphylococcus epidermidis	1	22,18504	1	27,21991
minneapolis	Staphylococcus aureus	5	0,99549	2	12,96274
minneapolis	Enterococcus faecalis	7	0,07809	13	0,01223
minneapolis	Pseudomonas aeruginosa	12	0,01170	5	0,11098

Table S3

minneapolis	Stenotrophomonas maltophilia	14	0,00924	21	0,00209
minneapolis	Escherichia coli	21	0,00227	17	0,00412
minneapolis	Pseudomonas fluorescens	24	0,00182	4	0,13593
minneapolis	Acinetobacter baumanni	30	0,00030	19	0,00342
naples	Staphylococcus epidermidis	6	0,06313	2	1,54142
naples	Stenotrophomonas maltophilia	7	0,03375	22	0,00263
naples	Pseudomonas aeruginosa	9	0,02645	6	0,24586
naples	Escherichia coli	10	0,01718	8	0,08005
naples	Acinetobacter baumanni	16	0,01183	5	0,53742
naples	Staphylococcus aureus	22	0,00762	4	0,89072
naples	Pseudomonas fluorescens	23	0,00643	7	0,16003
naples	Enterococcus faecalis	25	0,00367	33	0,00000
new_york_city	Stenotrophomonas maltophilia	4	0,50966	19	0,03671
new_york_city	Acinetobacter baumanni	6	0,23944	7	0,58176
new_york_city	Enterococcus faecalis	8	0,20652	11	0,21213
new_york_city	Pseudomonas fluorescens	9	0,17718	4	1,08560
new_york_city	Pseudomonas aeruginosa	12	0,11191	2	1,32995
new_york_city	Escherichia coli	14	0,04720	25	0,01978
new_york_city	Staphylococcus epidermidis	16	0,04262	9	0,40155
new_york_city	Staphylococcus aureus	17	0,04037	6	0,68677
offa	Escherichia coli	3	0,42705	8	0,14852
offa	Stenotrophomonas maltophilia	4	0,37813	12	0,07418
offa	Pseudomonas aeruginosa	5	0,08176	1	3,51296
offa	Acinetobacter baumanni	7	0,03776	9	0,13290
offa	Pseudomonas fluorescens	12	0,00429	2	1,72508
offa	Enterococcus faecalis	15	0,00292	21	0,00576
offa	Staphylococcus epidermidis	21	0,00092	19	0,01144
offa	Staphylococcus aureus	22	0,00076	23	0,00407
oslo	Stenotrophomonas maltophilia	2	0,21660	17	0,01163
oslo	Staphylococcus epidermidis	4	0,02196	1	0,49688
oslo	Pseudomonas aeruginosa	12	0,00931	8	0,15437
oslo	Pseudomonas fluorescens	14	0,00791	7	0,17267
oslo	Acinetobacter baumanni	19	0,00416	10	0,06730
oslo	Escherichia coli	21	0,00301	34	0,00000

Table S3

oslo	Staphylococcus aureus	23	0,00202	3	0,41477
oslo	Enterococcus faecalis	30	0,00049	23	0,00174
others	Enterococcus faecalis	1	47,54807	4	1,41964
others	Pseudomonas aeruginosa	2	37,70298	1	11,53677
others	Staphylococcus aureus	3	16,12318	3	4,44067
others	Escherichia coli	6	9,92451	7	0,51513
others	Staphylococcus epidermidis	9	4,28076	2	11,31146
others	Stenotrophomonas maltophilia	18	0,02135	21	0,00187
others	Pseudomonas fluorescens	19	0,01486	6	0,61556
others	Acinetobacter baumannii	26	0,00379	16	0,00879
porto	Stenotrophomonas maltophilia	2	0,19856	20	0,00776
porto	Staphylococcus epidermidis	4	0,09565	1	3,68248
porto	Acinetobacter baumannii	10	0,02167	12	0,18334
porto	Pseudomonas aeruginosa	12	0,01560	4	0,67798
porto	Escherichia coli	13	0,01161	23	0,00343
porto	Staphylococcus aureus	15	0,00994	2	2,21520
porto	Pseudomonas fluorescens	19	0,00688	3	0,83341
porto	Enterococcus faecalis	31	0,00114	28	0,00096
rio_de_janeiro	Acinetobacter baumannii	4	0,19590	1	1,84482
rio_de_janeiro	Staphylococcus epidermidis	7	0,08100	2	1,78515
rio_de_janeiro	Enterococcus faecalis	8	0,07935	19	0,03369
rio_de_janeiro	Escherichia coli	11	0,03538	15	0,04896
rio_de_janeiro	Stenotrophomonas maltophilia	13	0,02760	25	0,00785
rio_de_janeiro	Pseudomonas aeruginosa	14	0,02330	5	0,66934
rio_de_janeiro	Staphylococcus aureus	15	0,01933	3	1,77258
rio_de_janeiro	Pseudomonas fluorescens	23	0,00416	6	0,56701
santiago	Pseudomonas aeruginosa	4	0,03697	6	0,57349
santiago	Staphylococcus epidermidis	5	0,02854	1	2,62716
santiago	Escherichia coli	6	0,02416	16	0,03206
santiago	Acinetobacter baumannii	9	0,01892	2	1,46137
santiago	Stenotrophomonas maltophilia	10	0,01814	27	0,00588
santiago	Staphylococcus aureus	11	0,01283	3	1,01904
santiago	Pseudomonas fluorescens	14	0,01104	5	0,70206
santiago	Enterococcus faecalis	26	0,00311	24	0,00717

Table S3

sao paulo	Enterococcus faecalis	3	0,49891	10	0,13821
sao paulo	Pseudomonas aeruginosa	6	0,34283	4	0,40723
sao paulo	Escherichia coli	9	0,05109	14	0,01855
sao paulo	Staphylococcus epidermidis	10	0,03492	1	4,85565
sao paulo	Staphylococcus aureus	15	0,02394	2	1,13767
sao paulo	Stenotrophomonas maltophilia	18	0,01677	21	0,00106
sao paulo	Acinetobacter baumanni	23	0,00923	13	0,03691
sao paulo	Pseudomonas fluorescens	27	0,00391	3	0,69175
seoul	Pseudomonas aeruginosa	3	0,06907	10	0,34839
seoul	Staphylococcus epidermidis	9	0,02340	1	2,42021
seoul	Stenotrophomonas maltophilia	10	0,02167	23	0,00108
seoul	Acinetobacter baumanni	11	0,01479	12	0,02562
seoul	Escherichia coli	17	0,00941	13	0,01487
seoul	Pseudomonas fluorescens	20	0,00771	4	0,73770
seoul	Staphylococcus aureus	23	0,00469	2	1,31292
seoul	Enterococcus faecalis	32	0,00269	29	0,00000
singapore	Escherichia coli	3	0,31923	9	0,13262
singapore	Acinetobacter baumanni	4	0,26913	3	0,37242
singapore	Stenotrophomonas maltophilia	8	0,07942	17	0,01145
singapore	Pseudomonas aeruginosa	10	0,06437	6	0,21671
singapore	Staphylococcus epidermidis	18	0,01356	1	0,43078
singapore	Pseudomonas fluorescens	21	0,01000	2	0,42984
singapore	Enterococcus faecalis	22	0,00824	18	0,00731
singapore	Staphylococcus aureus	27	0,00201	5	0,22531
sofia	Stenotrophomonas maltophilia	4	0,19107	21	0,01591
sofia	Pseudomonas fluorescens	5	0,12091	7	0,29806
sofia	Staphylococcus epidermidis	11	0,03052	6	0,33165
sofia	Pseudomonas aeruginosa	12	0,03040	4	0,54884
sofia	Acinetobacter baumanni	16	0,02050	3	1,08383
sofia	Escherichia coli	22	0,01039	15	0,03073
sofia	Staphylococcus aureus	26	0,00602	5	0,36382
sofia	Enterococcus faecalis	34	0,00077	28	0,00373
taipei	Stenotrophomonas maltophilia	3	0,11351	13	0,06065
taipei	Pseudomonas aeruginosa	5	0,04199	7	0,13700

Table S3

taipei	Acinetobacter baumanni	16	0,00644	14	0,03088
taipei	Pseudomonas fluorescens	18	0,00349	3	0,34388
taipei	Escherichia coli	19	0,00220	21	0,00206
taipei	Staphylococcus epidermidis	22	0,00182	2	0,57050
taipei	Staphylococcus aureus	29	0,00046	6	0,17561
taipei	Enterococcus faecalis	32	0,00008	30	0,00000
tokyo	Staphylococcus epidermidis	3	0,29841	2	4,61402
tokyo	Pseudomonas fluorescens	5	0,10028	7	0,49164
tokyo	Staphylococcus aureus	7	0,07276	1	4,85441
tokyo	Acinetobacter baumanni	8	0,06761	3	1,71600
tokyo	Pseudomonas aeruginosa	13	0,02354	4	0,69752
tokyo	Escherichia coli	15	0,02135	20	0,04159
tokyo	Stenotrophomonas maltophilia	16	0,01910	23	0,01204
tokyo	Enterococcus faecalis	31	0,00342	26	0,00678
vienna	Stenotrophomonas maltophilia	3	0,15312	8	0,08815
vienna	Pseudomonas aeruginosa	5	0,08412	1	0,83098
vienna	Pseudomonas fluorescens	6	0,05431	4	0,29058
vienna	Acinetobacter baumanni	13	0,01639	2	0,50323
vienna	Escherichia coli	20	0,00460	14	0,02620
vienna	Staphylococcus epidermidis	21	0,00403	6	0,16189
vienna	Staphylococcus aureus	28	0,00095	7	0,13937
vienna	Enterococcus faecalis	34	0,00023	26	0,00000
zurich	Stenotrophomonas maltophilia	1	1,86656	12	0,01482
zurich	Pseudomonas aeruginosa	7	0,02790	1	0,93058
zurich	Escherichia coli	16	0,00265	8	0,03456
zurich	Pseudomonas fluorescens	18	0,00244	3	0,19484
zurich	Acinetobacter baumanni	21	0,00093	4	0,07697
zurich	Staphylococcus epidermidis	25	0,00018	5	0,05728
zurich	Staphylococcus aureus	26	0,00018	7	0,05156
zurich	Enterococcus faecalis	27	0,00015	21	0,00071

Table S4

Supplementary Table S4. Rank of PPG hosts predicted by CRISPR.

City	Bacteria/ PPG Host	Bacteria abudance ranl	Bacteria abundance (%)	PPGs abudance ranl	PPGs abundance (%)
auckland	Pseudomonas aeruginosa	3	0,08573	15	0,85106
auckland	Pseudomonas fluorescens	4	0,08441	8	2,19907
auckland	Pseudomonas stutzeri	5	0,04574	13	0,97370
auckland	Xanthomonas campestris	6	0,03666	7	2,29225
auckland	Escherichia coli	7	0,02382	27	0,44122
auckland	Salmonella enterica	9	0,01787	6	2,34556
auckland	Acinetobacter baumanni	10	0,01772	14	0,90908
auckland	Klebsiella pneumoniae	11	0,01650	19	0,70092
auckland	Acinetobacter juni	13	0,00634	232	0,00000
auckland	Moraxella osloensis	14	0,00267	149	0,00363
auckland	Rothia mucilaginosa	21	0,00104	156	0,00187
auckland	Listeria monocytogenes	22	0,00102	4	2,60886
auckland	Enterococcus faecalis	25	0,00058	68	0,10899
auckland	Pseudomonas balearica	36	0,00000	43	0,23392
auckland	Cutibacterium granulosum	55	0,00000	64	0,12470
auckland	Micrococcus luteus	69	0,00000	86	0,08102
berlin	Pseudomonas stutzeri	1	4,52229	1	6,55877
berlin	Moraxella osloensis	5	0,13890	55	0,26791
berlin	Pseudomonas aeruginosa	6	0,09053	9	1,59055
berlin	Acinetobacter baumanni	10	0,03613	17	0,94560
berlin	Escherichia coli	12	0,02049	67	0,13199
berlin	Acinetobacter juni	13	0,01830	125	0,01893
berlin	Rothia mucilaginosa	14	0,01808	103	0,03887
berlin	Salmonella enterica	16	0,01418	25	0,64757
berlin	Klebsiella pneumoniae	17	0,01301	22	0,69782
berlin	Pseudomonas fluorescens	21	0,00954	15	1,05155
berlin	Xanthomonas campestris	24	0,00670	3	4,77459
berlin	Enterococcus faecalis	26	0,00362	29	0,48392
berlin	Listeria monocytogenes	30	0,00210	162	0,00778
berlin	Pseudomonas balearica	52	0,00000	5	2,70975

Table S4

berlin	Micrococcus luteus	66	0,00000	6	2,25634
berlin	Cutibacterium granulosum	68	0,00000	31	0,45049
bogota	Pseudomonas fluorescens	2	0,17466	10	1,55469
bogota	Pseudomonas stutzeri	4	0,10621	6	1,95882
bogota	Moraxella osloensis	6	0,06720	29	0,48058
bogota	Acinetobacter baumannii	7	0,03153	4	2,52002
bogota	Pseudomonas aeruginosa	9	0,02862	22	0,84119
bogota	Klebsiella pneumoniae	10	0,02302	15	1,10017
bogota	Escherichia coli	12	0,01959	30	0,44807
bogota	Rothia mucilaginosa	19	0,01154	109	0,05740
bogota	Salmonella enterica	20	0,00908	13	1,46765
bogota	Acinetobacter junii	28	0,00537	149	0,02330
bogota	Enterococcus faecalis	29	0,00495	49	0,25701
bogota	Xanthomonas campestris	31	0,00487	11	1,48748
bogota	Listeria monocytogenes	33	0,00128	191	0,00754
bogota	Pseudomonas balearica	63	0,00000	21	0,88108
bogota	Micrococcus luteus	65	0,00000	39	0,38170
bogota	Cutibacterium granulosum	70	0,00000	12	1,46911
cairngorm	Pseudomonas aeruginosa	2	0,22953	38	0,40364
cairngorm	Listeria monocytogenes	4	0,16912	47	0,21200
cairngorm	Enterococcus faecalis	6	0,14260	9	0,98259
cairngorm	Salmonella enterica	8	0,09895	16	0,78112
cairngorm	Escherichia coli	9	0,09505	46	0,24652
cairngorm	Moraxella osloensis	10	0,02972	64	0,03591
cairngorm	Pseudomonas stutzeri	13	0,00955	42	0,29979
cairngorm	Acinetobacter baumannii	18	0,00387	54	0,09933
cairngorm	Rothia mucilaginosa	22	0,00258	86	0,01005
cairngorm	Xanthomonas campestris	25	0,00195	12	0,85223
cairngorm	Pseudomonas fluorescens	27	0,00180	50	0,10774
cairngorm	Acinetobacter junii	29	0,00105	214	0,00000
cairngorm	Klebsiella pneumoniae	33	0,00054	5	1,23322
cairngorm	Pseudomonas balearica	37	0,00000	73	0,02076
cairngorm	Cutibacterium granulosum	51	0,00000	15	0,79188
cairngorm	Micrococcus luteus	70	0,00000	11	0,88378

Table S4

denver	Moraxella osloensis	5	0,03368	66	0,05404
denver	Klebsiella pneumoniae	7	0,02088	20	0,30137
denver	Pseudomonas aeruginosa	8	0,01474	35	0,12653
denver	Pseudomonas fluorescens	9	0,01181	16	0,36119
denver	Pseudomonas stutzeri	10	0,01010	31	0,14562
denver	Acinetobacter baumannii	11	0,00956	19	0,30748
denver	Escherichia coli	12	0,00827	71	0,04812
denver	Rothia mucilaginosa	14	0,00713	103	0,01743
denver	Xanthomonas campestris	21	0,00575	1	8,13530
denver	Acinetobacter junii	26	0,00198	223	0,00000
denver	Enterococcus faecalis	31	0,00132	60	0,06939
denver	Salmonella enterica	32	0,00096	23	0,21440
denver	Listeria monocytogenes	34	0,00057	175	0,00185
denver	Pseudomonas balearica	37	0,00000	52	0,08386
denver	Cutibacterium granulosum	56	0,00000	18	0,31151
denver	Micrococcus luteus	68	0,00000	11	0,56875
doha	Pseudomonas aeruginosa	2	3,99929	2	3,93636
doha	Salmonella enterica	3	2,87261	3	3,29462
doha	Escherichia coli	4	2,47975	7	2,08987
doha	Pseudomonas stutzeri	5	0,99980	4	3,21919
doha	Enterococcus faecalis	7	0,17743	16	0,74048
doha	Moraxella osloensis	11	0,07067	75	0,03734
doha	Pseudomonas fluorescens	12	0,03567	30	0,51092
doha	Listeria monocytogenes	14	0,02491	82	0,02663
doha	Acinetobacter baumannii	15	0,02114	56	0,20663
doha	Acinetobacter junii	16	0,01413	148	0,00197
doha	Klebsiella pneumoniae	19	0,00830	13	0,94099
doha	Rothia mucilaginosa	24	0,00184	130	0,00503
doha	Xanthomonas campestris	27	0,00116	8	1,48539
doha	Pseudomonas balearica	37	0,00000	5	2,88663
doha	Cutibacterium granulosum	52	0,00000	26	0,55770
doha	Micrococcus luteus	71	0,00000	48	0,32858
fairbanks	Moraxella osloensis	7	0,07937	30	0,40549
fairbanks	Klebsiella pneumoniae	10	0,04040	20	0,78527

Table S4

fairbanks	Escherichia coli	12	0,02799	64	0,13365
fairbanks	Salmonella enterica	13	0,01847	32	0,37813
fairbanks	Pseudomonas fluorescens	14	0,01791	8	1,53401
fairbanks	Pseudomonas stutzeri	15	0,01412	35	0,31945
fairbanks	Pseudomonas aeruginosa	16	0,01154	40	0,24254
fairbanks	Acinetobacter baumanni	18	0,00979	25	0,68835
fairbanks	Rothia mucilaginosa	20	0,00482	128	0,01998
fairbanks	Xanthomonas campestris	21	0,00478	1	3,33440
fairbanks	Enterococcus faecalis	27	0,00174	42	0,23730
fairbanks	Acinetobacter juni	29	0,00112	110	0,03096
fairbanks	Listeria monocytogenes	32	0,00072	205	0,00097
fairbanks	Pseudomonas balearica	51	0,00000	60	0,14537
fairbanks	Cutibacterium granulosum	57	0,00000	72	0,11812
fairbanks	Micrococcus luteus	68	0,00000	38	0,24919
hamilton	Pseudomonas fluorescens	3	0,20556	4	4,67082
hamilton	Pseudomonas aeruginosa	4	0,08008	17	1,28133
hamilton	Acinetobacter baumanni	5	0,06056	24	0,80555
hamilton	Escherichia coli	7	0,05464	35	0,53520
hamilton	Pseudomonas stutzeri	8	0,04890	33	0,56056
hamilton	Salmonella enterica	9	0,04879	3	5,17294
hamilton	Xanthomonas campestris	10	0,03839	2	5,32160
hamilton	Klebsiella pneumoniae	11	0,03372	7	2,50113
hamilton	Enterococcus faecalis	12	0,01254	65	0,09989
hamilton	Acinetobacter juni	13	0,00868	187	0,00014
hamilton	Moraxella osloensis	14	0,00358	123	0,00982
hamilton	Listeria monocytogenes	22	0,00064	152	0,00222
hamilton	Rothia mucilaginosa	30	0,00004	199	0,00005
hamilton	Pseudomonas balearica	37	0,00000	49	0,32604
hamilton	Cutibacterium granulosum	55	0,00000	128	0,00889
hamilton	Micrococcus luteus	70	0,00000	135	0,00792
hanoi	Escherichia coli	1	1,17647	5	3,73438
hanoi	Moraxella osloensis	2	0,40188	14	1,00667
hanoi	Acinetobacter baumanni	5	0,06655	9	1,72548
hanoi	Acinetobacter juni	6	0,06102	93	0,02855

Table S4

hanoi	Klebsiella pneumoniae	8	0,03840	21	0,42399
hanoi	Pseudomonas aeruginosa	10	0,01564	36	0,16365
hanoi	Xanthomonas campestris	13	0,00963	25	0,28410
hanoi	Enterococcus faecalis	14	0,00898	34	0,16918
hanoi	Rothia mucilaginosa	16	0,00764	79	0,04070
hanoi	Pseudomonas stutzeri	18	0,00543	41	0,13940
hanoi	Pseudomonas fluorescens	27	0,00167	8	1,74143
hanoi	Salmonella enterica	30	0,00134	4	4,01262
hanoi	Listeria monocytogenes	32	0,00084	163	0,00411
hanoi	Pseudomonas balearica	45	0,00000	66	0,06015
hanoi	Cutibacterium granulosum	53	0,00000	147	0,00640
hanoi	Micrococcus luteus	70	0,00000	43	0,13482
hong_kong	Moraxella osloensis	2	0,23359	38	0,22450
hong_kong	Pseudomonas aeruginosa	4	0,07228	8	0,81921
hong_kong	Acinetobacter baumannii	8	0,02743	16	0,48337
hong_kong	Pseudomonas stutzeri	9	0,02576	33	0,28006
hong_kong	Xanthomonas campestris	10	0,02401	7	0,94496
hong_kong	Acinetobacter junii	18	0,00810	116	0,01718
hong_kong	Rothia mucilaginosa	19	0,00759	111	0,02016
hong_kong	Escherichia coli	23	0,00485	93	0,03564
hong_kong	Klebsiella pneumoniae	24	0,00467	4	2,34789
hong_kong	Pseudomonas fluorescens	26	0,00275	17	0,48301
hong_kong	Enterococcus faecalis	28	0,00131	55	0,13779
hong_kong	Salmonella enterica	31	0,00050	21	0,43386
hong_kong	Listeria monocytogenes	34	0,00009	251	0,00010
hong_kong	Pseudomonas balearica	44	0,00000	25	0,35621
hong_kong	Cutibacterium granulosum	55	0,00000	36	0,24531
hong_kong	Micrococcus luteus	70	0,00000	3	4,35046
ilorin	Pseudomonas stutzeri	1	6,19747	1	15,41225
ilorin	Klebsiella pneumoniae	3	0,20051	7	2,16928
ilorin	Escherichia coli	4	0,18761	42	0,26991
ilorin	Pseudomonas aeruginosa	5	0,09112	3	5,12184
ilorin	Moraxella osloensis	6	0,06750	116	0,04091
ilorin	Acinetobacter baumannii	7	0,06359	14	0,96001

Table S4

ilorin	Salmonella enterica	9	0,04110	4	2,72481
ilorin	Acinetobacter juni	11	0,01170	222	0,00189
ilorin	Pseudomonas fluorescens	16	0,00540	8	2,15907
ilorin	Enterococcus faecalis	17	0,00440	82	0,10435
ilorin	Listeria monocytogenes	18	0,00288	149	0,02131
ilorin	Xanthomonas campestris	20	0,00236	21	0,75109
ilorin	Rothia mucilaginosa	30	0,00047	202	0,00424
ilorin	Pseudomonas balearica	47	0,00000	2	7,67846
ilorin	Micrococcus luteus	69	0,00000	120	0,03842
ilorin	Cutibacterium granulosum	70	0,00000	136	0,02848
kuala_lumpur	Pseudomonas aeruginosa	1	43,17741	1	32,87846
kuala_lumpur	Salmonella enterica	2	16,69142	3	11,64484
kuala_lumpur	Escherichia coli	3	11,57926	2	12,76409
kuala_lumpur	Listeria monocytogenes	5	0,94545	14	0,45021
kuala_lumpur	Enterococcus faecalis	6	0,58779	10	0,96272
kuala_lumpur	Pseudomonas stutzeri	11	0,00240	11	0,86990
kuala_lumpur	Klebsiella pneumoniae	15	0,00076	20	0,28497
kuala_lumpur	Xanthomonas campestris	16	0,00073	53	0,02860
kuala_lumpur	Pseudomonas fluorescens	17	0,00048	8	1,30556
kuala_lumpur	Moraxella osloensis	19	0,00036	300	0,00000
kuala_lumpur	Rothia mucilaginosa	24	0,00012	161	0,00000
kuala_lumpur	Acinetobacter baumannii	30	0,00003	107	0,00044
kuala_lumpur	Acinetobacter juni	31	0,00002	193	0,00000
kuala_lumpur	Pseudomonas balearica	35	0,00000	19	0,29001
kuala_lumpur	Cutibacterium granulosum	49	0,00000	251	0,00000
kuala_lumpur	Micrococcus luteus	67	0,00000	94	0,00114
kyiv	Moraxella osloensis	1	1,91439	2	4,76673
kyiv	Klebsiella pneumoniae	2	1,24649	3	4,09160
kyiv	Acinetobacter baumannii	4	0,29822	15	1,11743
kyiv	Escherichia coli	6	0,07665	66	0,14511
kyiv	Pseudomonas aeruginosa	7	0,06321	71	0,11268
kyiv	Salmonella enterica	13	0,01718	4	3,28529
kyiv	Xanthomonas campestris	15	0,01342	27	0,67705
kyiv	Acinetobacter juni	16	0,01111	114	0,00934

Table S4

kyiv	Pseudomonas stutzeri	17	0,01055	63	0,20856
kyiv	Rothia mucilaginosa	23	0,00459	119	0,00857
kyiv	Pseudomonas fluorescens	26	0,00298	30	0,51067
kyiv	Enterococcus faecalis	29	0,00179	61	0,23873
kyiv	Listeria monocytogenes	30	0,00137	217	0,00013
kyiv	Pseudomonas balearica	43	0,00000	94	0,02308
kyiv	Cutibacterium granulosum	52	0,00000	35	0,39258
kyiv	Micrococcus luteus	71	0,00000	50	0,30237
lisbon	Pseudomonas stutzeri	1	6,53718	1	10,98051
lisbon	Acinetobacter baumanni	3	0,77363	4	5,19350
lisbon	Moraxella osloensis	4	0,53827	39	0,39775
lisbon	Pseudomonas aeruginosa	6	0,10960	5	4,37174
lisbon	Enterococcus faecalis	7	0,07494	58	0,19315
lisbon	Escherichia coli	8	0,04810	12	1,41448
lisbon	Klebsiella pneumoniae	9	0,02830	19	0,80472
lisbon	Salmonella enterica	10	0,02745	6	3,55380
lisbon	Xanthomonas campestris	11	0,02237	23	0,64077
lisbon	Acinetobacter juni	12	0,01633	119	0,02262
lisbon	Pseudomonas fluorescens	16	0,01068	10	1,91723
lisbon	Rothia mucilaginosa	19	0,00466	163	0,00583
lisbon	Listeria monocytogenes	22	0,00172	150	0,01210
lisbon	Pseudomonas balearica	37	0,00000	3	6,07824
lisbon	Cutibacterium granulosum	55	0,00000	133	0,01608
lisbon	Micrococcus luteus	70	0,00000	115	0,02538
london	Pseudomonas aeruginosa	1	5,04627	7	1,09211
london	Listeria monocytogenes	2	4,10031	25	0,63378
london	Enterococcus faecalis	4	3,59397	3	1,94957
london	Escherichia coli	5	3,24318	34	0,44164
london	Salmonella enterica	7	2,84640	22	0,74314
london	Pseudomonas stutzeri	11	0,36400	1	2,21719
london	Pseudomonas fluorescens	15	0,06375	21	0,77069
london	Moraxella osloensis	20	0,01139	20	0,77279
london	Rothia mucilaginosa	21	0,00758	136	0,03283
london	Xanthomonas campestris	23	0,00448	2	2,02440

Table S4

london	Acinetobacter baumanni	24	0,00409	16	0,84502
london	Klebsiella pneumoniae	32	0,00156	9	1,01296
london	Acinetobacter juni	34	0,00145	173	0,00764
london	Pseudomonas balearica	42	0,00000	10	0,96846
london	Cutibacterium granulosum	58	0,00000	56	0,25487
london	Micrococcus luteus	67	0,00000	12	0,91879
marseille	Moraxella osloensis	3	0,15305	34	0,14252
marseille	Rothia mucilaginosa	5	0,06153	40	0,10894
marseille	Pseudomonas aeruginosa	6	0,05407	11	0,46929
marseille	Pseudomonas stutzeri	7	0,05354	15	0,32113
marseille	Acinetobacter baumanni	9	0,03945	7	0,70525
marseille	Xanthomonas campestris	15	0,01675	20	0,21123
marseille	Pseudomonas fluorescens	16	0,01577	6	0,72850
marseille	Escherichia coli	17	0,01542	38	0,12259
marseille	Klebsiella pneumoniae	18	0,01504	23	0,19865
marseille	Acinetobacter juni	21	0,01007	121	0,01289
marseille	Salmonella enterica	28	0,00388	9	0,50272
marseille	Enterococcus faecalis	32	0,00176	68	0,04693
marseille	Listeria monocytogenes	34	0,00074	194	0,00185
marseille	Pseudomonas balearica	47	0,00000	30	0,16568
marseille	Cutibacterium granulosum	54	0,00000	17	0,22237
marseille	Micrococcus luteus	71	0,00000	3	1,71408
minneapolis	Rothia mucilaginosa	6	0,13942	7	3,14955
minneapolis	Enterococcus faecalis	7	0,07809	19	0,22352
minneapolis	Pseudomonas aeruginosa	12	0,01170	29	0,10709
minneapolis	Pseudomonas stutzeri	13	0,00971	22	0,16805
minneapolis	Salmonella enterica	19	0,00238	27	0,11524
minneapolis	Escherichia coli	21	0,00227	79	0,02148
minneapolis	Xanthomonas campestris	22	0,00222	12	0,71878
minneapolis	Moraxella osloensis	23	0,00200	139	0,00318
minneapolis	Pseudomonas fluorescens	24	0,00182	17	0,45894
minneapolis	Listeria monocytogenes	26	0,00144	107	0,00895
minneapolis	Klebsiella pneumoniae	28	0,00079	8	3,08158
minneapolis	Acinetobacter baumanni	30	0,00030	72	0,02465

Table S4

minneapolis	Acinetobacter juni	34	0,00011	239	0,00000
minneapolis	Pseudomonas balearica	53	0,00000	41	0,07178
minneapolis	Micrococcus luteus	67	0,00000	28	0,11426
minneapolis	Cutibacterium granulosum	69	0,00000	24	0,14791
naples	Moraxella osloensis	3	0,09374	28	0,20132
naples	Pseudomonas stutzeri	8	0,03235	18	0,51591
naples	Pseudomonas aeruginosa	9	0,02645	12	0,80814
naples	Escherichia coli	10	0,01718	48	0,10022
naples	Acinetobacter baumanni	16	0,01183	9	1,17351
naples	Acinetobacter juni	18	0,01154	163	0,00485
naples	Rothia mucilaginosa	19	0,01117	69	0,04915
naples	Xanthomonas campestris	21	0,00783	16	0,54700
naples	Pseudomonas fluorescens	23	0,00643	10	0,92241
naples	Enterococcus faecalis	25	0,00367	62	0,06919
naples	Salmonella enterica	26	0,00297	23	0,27629
naples	Klebsiella pneumoniae	29	0,00173	29	0,18680
naples	Listeria monocytogenes	35	0,00016	281	0,00000
naples	Pseudomonas balearica	47	0,00000	37	0,14756
naples	Cutibacterium granulosum	58	0,00000	39	0,13553
naples	Micrococcus luteus	68	0,00000	15	0,59382
new_york_city	Pseudomonas stutzeri	2	1,01739	1	4,28840
new_york_city	Acinetobacter baumanni	6	0,23944	3	2,60855
new_york_city	Enterococcus faecalis	8	0,20652	8	1,40663
new_york_city	Pseudomonas fluorescens	9	0,17718	7	1,50503
new_york_city	Pseudomonas aeruginosa	12	0,11191	9	1,20954
new_york_city	Escherichia coli	14	0,04720	38	0,38183
new_york_city	Moraxella osloensis	15	0,04262	18	0,93274
new_york_city	Salmonella enterica	18	0,03054	16	1,05024
new_york_city	Listeria monocytogenes	19	0,02107	48	0,30442
new_york_city	Klebsiella pneumoniae	20	0,01869	22	0,64894
new_york_city	Acinetobacter juni	22	0,01444	185	0,01838
new_york_city	Xanthomonas campestris	23	0,00822	4	2,11218
new_york_city	Rothia mucilaginosa	26	0,00609	173	0,02520
new_york_city	Pseudomonas balearica	41	0,00000	5	2,04452

Table S4

new_york_city	Micrococcus luteus	67	0,00000	70	0,16110
new_york_city	Cutibacterium granulosum	71	0,00000	151	0,03871
offa	Pseudomonas stutzeri	1	4,30273	1	13,26220
offa	Klebsiella pneumoniae	2	1,02931	7	2,16938
offa	Escherichia coli	3	0,42705	20	0,89874
offa	Pseudomonas aeruginosa	5	0,08176	4	3,90077
offa	Salmonella enterica	6	0,05196	3	4,14323
offa	Acinetobacter baumannii	7	0,03776	29	0,58623
offa	Acinetobacter junii	11	0,01056	161	0,00490
offa	Pseudomonas fluorescens	12	0,00429	10	1,70458
offa	Xanthomonas campestris	13	0,00353	30	0,58480
offa	Moraxella osloensis	14	0,00296	172	0,00276
offa	Enterococcus faecalis	15	0,00292	72	0,10221
offa	Listeria monocytogenes	17	0,00224	182	0,00186
offa	Rothia mucilaginosa	32	0,00008	244	0,00004
offa	Pseudomonas balearica	37	0,00000	2	6,68918
offa	Cutibacterium granulosum	53	0,00000	170	0,00288
offa	Micrococcus luteus	70	0,00000	204	0,00043
oslo	Moraxella osloensis	5	0,02138	77	0,04076
oslo	Pseudomonas stutzeri	10	0,00947	30	0,17046
oslo	Pseudomonas aeruginosa	12	0,00931	41	0,11858
oslo	Pseudomonas fluorescens	14	0,00791	12	0,66193
oslo	Xanthomonas campestris	16	0,00506	1	9,59473
oslo	Rothia mucilaginosa	17	0,00494	98	0,01792
oslo	Acinetobacter baumannii	19	0,00416	29	0,18785
oslo	Escherichia coli	21	0,00301	87	0,02794
oslo	Acinetobacter junii	25	0,00137	181	0,00099
oslo	Klebsiella pneumoniae	26	0,00132	26	0,21277
oslo	Enterococcus faecalis	30	0,00049	58	0,07178
oslo	Salmonella enterica	31	0,00047	38	0,13381
oslo	Listeria monocytogenes	34	0,00019	285	0,00000
oslo	Pseudomonas balearica	36	0,00000	61	0,06847
oslo	Cutibacterium granulosum	49	0,00000	17	0,33108
oslo	Micrococcus luteus	68	0,00000	5	2,88370

Table S4

others	Enterococcus faecalis	1	47,54807	1	13,60492
others	Pseudomonas aeruginosa	2	37,70298	2	10,33503
others	Listeria monocytogenes	5	12,24438	12	1,46657
others	Escherichia coli	6	9,92451	5	3,80827
others	Salmonella enterica	7	9,59761	8	2,15302
others	Pseudomonas stutzeri	10	0,77212	7	2,25665
others	Klebsiella pneumoniae	16	0,03380	27	0,39037
others	Pseudomonas fluorescens	19	0,01486	19	0,65709
others	Moraxella osloensis	23	0,00546	125	0,00223
others	Acinetobacter baumanni	26	0,00379	77	0,04023
others	Rothia mucilaginosa	30	0,00152	183	0,00026
others	Acinetobacter juni	31	0,00107	212	0,00006
others	Xanthomonas campestris	32	0,00075	6	3,75250
others	Pseudomonas balearica	53	0,00000	15	1,11758
others	Micrococcus luteus	66	0,00000	68	0,09694
others	Cutibacterium granulosum	68	0,00000	42	0,18221
porto	Pseudomonas stutzeri	3	0,14555	11	1,27928
porto	Moraxella osloensis	7	0,03837	100	0,02732
porto	Acinetobacter baumanni	10	0,02167	50	0,33972
porto	Pseudomonas aeruginosa	12	0,01560	48	0,37929
porto	Escherichia coli	13	0,01161	15	0,89464
porto	Rothia mucilaginosa	16	0,00873	144	0,00462
porto	Klebsiella pneumoniae	18	0,00859	9	1,33434
porto	Pseudomonas fluorescens	19	0,00688	46	0,42636
porto	Salmonella enterica	22	0,00588	16	0,87115
porto	Acinetobacter juni	23	0,00502	147	0,00387
porto	Xanthomonas campestris	25	0,00389	7	1,43126
porto	Enterococcus faecalis	31	0,00114	41	0,49713
porto	Listeria monocytogenes	32	0,00033	225	0,00016
porto	Pseudomonas balearica	52	0,00000	43	0,48740
porto	Micrococcus luteus	66	0,00000	33	0,59122
porto	Cutibacterium granulosum	68	0,00000	22	0,72659
rio_de_janeiro	Pseudomonas stutzeri	1	0,85683	6	2,15705
rio_de_janeiro	Moraxella osloensis	3	0,20560	13	1,30679

Table S4

rio_de_janeirc	Acinetobacter baumanni	4	0,19590	2	5,36695
rio_de_janeirc	Enterococcus faecalis	8	0,07935	46	0,27812
rio_de_janeirc	Acinetobacter juni	9	0,06500	142	0,02266
rio_de_janeirc	Escherichia coli	11	0,03538	21	0,96718
rio_de_janeirc	Klebsiella pneumoniae	12	0,02927	24	0,69983
rio_de_janeirc	Pseudomonas aeruginosa	14	0,02330	19	1,01889
rio_de_janeirc	Salmonella enterica	16	0,01770	23	0,83304
rio_de_janeirc	Rothia mucilaginosa	20	0,00790	146	0,02080
rio_de_janeirc	Pseudomonas fluorescens	23	0,00416	7	1,99884
rio_de_janeirc	Xanthomonas campestris	28	0,00289	25	0,64512
rio_de_janeirc	Listeria monocytogenes	31	0,00241	185	0,00849
rio_de_janeirc	Micrococcus luteus	65	0,00000	51	0,23786
rio_de_janeirc	Cutibacterium granulosum	70	0,00000	87	0,09190
rio_de_janeirc	Pseudomonas balearica	71	0,00000	14	1,26208
santiago	Pseudomonas stutzeri	2	0,07849	7	1,20328
santiago	Pseudomonas aeruginosa	4	0,03697	25	0,56510
santiago	Escherichia coli	6	0,02416	67	0,16156
santiago	Klebsiella pneumoniae	7	0,02410	17	0,98032
santiago	Acinetobacter baumanni	9	0,01892	4	2,40785
santiago	Salmonella enterica	12	0,01198	9	1,11961
santiago	Moraxella osloensis	13	0,01173	38	0,44851
santiago	Pseudomonas fluorescens	14	0,01104	12	1,06388
santiago	Rothia mucilaginosa	18	0,00885	108	0,03026
santiago	Acinetobacter juni	21	0,00463	128	0,01808
santiago	Xanthomonas campestris	22	0,00388	5	2,30607
santiago	Enterococcus faecalis	26	0,00311	31	0,49190
santiago	Listeria monocytogenes	32	0,00183	172	0,00470
santiago	Cutibacterium granulosum	60	0,00000	28	0,51852
santiago	Micrococcus luteus	64	0,00000	54	0,29706
santiago	Pseudomonas balearica	70	0,00000	34	0,45846
sao paulo	Enterococcus faecalis	3	0,49891	7	1,99944
sao paulo	Pseudomonas aeruginosa	6	0,34283	28	0,27696
sao paulo	Salmonella enterica	7	0,05843	20	0,44120
sao paulo	Escherichia coli	9	0,05109	33	0,24959

Table S4

sao paulo	Rothia mucilaginosa	14	0,02592	62	0,08704
sao paulo	Acinetobacter juni:	16	0,01895	166	0,00077
sao paulo	Listeria monocytogenes	17	0,01690	61	0,09475
sao paulo	Klebsiella pneumoniae	22	0,00964	15	0,60954
sao paulo	Acinetobacter baumanni	23	0,00923	56	0,11194
sao paulo	Moraxella osloensis	24	0,00716	127	0,00243
sao paulo	Pseudomonas stutzeri	26	0,00399	58	0,10314
sao paulo	Pseudomonas fluorescens	27	0,00391	50	0,16710
sao paulo	Xanthomonas campestris	29	0,00107	2	9,31724
sao paulo	Cutibacterium granulosum	53	0,00000	25	0,34213
sao paulo	Pseudomonas balearica	63	0,00000	97	0,00986
sao paulo	Micrococcus luteus	65	0,00000	54	0,15550
seoul	Moraxella osloensis	2	0,12567	61	0,02491
seoul	Pseudomonas aeruginosa	3	0,06907	54	0,06566
seoul	Rothia mucilaginosa	8	0,04211	64	0,01937
seoul	Acinetobacter baumanni	11	0,01479	47	0,20136
seoul	Xanthomonas campestris	12	0,01476	9	1,24019
seoul	Klebsiella pneumoniae	14	0,01280	4	2,12680
seoul	Pseudomonas stutzeri	15	0,01157	44	0,32522
seoul	Escherichia coli	17	0,00941	51	0,09765
seoul	Pseudomonas fluorescens	20	0,00771	52	0,07937
seoul	Acinetobacter juni:	24	0,00432	186	0,00054
seoul	Salmonella enterica	27	0,00399	24	0,72010
seoul	Listeria monocytogenes	31	0,00273	156	0,00115
seoul	Enterococcus faecalis	32	0,00269	33	0,59031
seoul	Cutibacterium granulosum	43	0,00000	13	0,83329
seoul	Micrococcus luteus	56	0,00000	21	0,74583
seoul	Pseudomonas balearica	69	0,00000	68	0,01666
singapore	Moraxella osloensis	2	0,44844	52	0,11419
singapore	Escherichia coli	3	0,31923	22	0,29344
singapore	Acinetobacter baumanni	4	0,26913	7	1,19746
singapore	Rothia mucilaginosa	7	0,09101	57	0,09391
singapore	Pseudomonas aeruginosa	10	0,06437	33	0,19517
singapore	Klebsiella pneumoniae	12	0,03731	12	0,54034

Table S4

singapore	Pseudomonas stutzeri	13	0,03659	30	0,23050
singapore	Acinetobacter juni:	17	0,01535	139	0,00667
singapore	Xanthomonas campestris	20	0,01152	3	5,14627
singapore	Pseudomonas fluorescens	21	0,01000	14	0,47515
singapore	Enterococcus faecalis	22	0,00824	44	0,14657
singapore	Salmonella enterica	33	0,00070	8	0,84397
singapore	Listeria monocytogenes	35	0,00033	292	0,00000
singapore	Pseudomonas balearica	47	0,00000	61	0,09187
singapore	Cutibacterium granulosum	52	0,00000	36	0,18541
singapore	Micrococcus luteus	69	0,00000	1	32,88693
sofia	Moraxella osloensis	2	0,36995	22	0,44925
sofia	Pseudomonas fluorescens	5	0,12091	8	1,98887
sofia	Xanthomonas campestris	8	0,05462	13	0,88798
sofia	Pseudomonas stutzeri	10	0,04762	16	0,70331
sofia	Pseudomonas aeruginosa	12	0,03040	19	0,55493
sofia	Rothia mucilaginosa	13	0,03008	74	0,07430
sofia	Acinetobacter juni:	15	0,02083	131	0,02252
sofia	Acinetobacter baumanni:	16	0,02050	7	2,20440
sofia	Escherichia coli	22	0,01039	50	0,15112
sofia	Klebsiella pneumoniae	28	0,00429	3	5,52393
sofia	Salmonella enterica	30	0,00367	20	0,50140
sofia	Enterococcus faecalis	34	0,00077	71	0,07995
sofia	Listeria monocytogenes	35	0,00023	225	0,00044
sofia	Pseudomonas balearica	41	0,00000	29	0,32596
sofia	Cutibacterium granulosum	53	0,00000	45	0,17351
sofia	Micrococcus luteus	71	0,00000	4	3,62901
taipei	Moraxella osloensis	2	0,22785	46	0,09103
taipei	Acinetobacter juni:	4	0,05343	102	0,00769
taipei	Pseudomonas aeruginosa	5	0,04199	30	0,14234
taipei	Xanthomonas campestris	7	0,02972	4	4,74514
taipei	Pseudomonas stutzeri	9	0,02641	19	0,22837
taipei	Acinetobacter baumanni:	16	0,00644	26	0,17134
taipei	Pseudomonas fluorescens	18	0,00349	11	0,36919
taipei	Escherichia coli	19	0,00220	78	0,02475

Table S4

taipei	Rothia mucilaginosa	20	0,00218	126	0,00333
taipei	Klebsiella pneumoniae	21	0,00201	3	5,91000
taipei	Salmonella enterica	31	0,00035	21	0,21333
taipei	Enterococcus faecalis	32	0,00008	43	0,09930
taipei	Listeria monocytogenes	34	0,00003	209	0,00008
taipei	Pseudomonas balearica	45	0,00000	51	0,08090
taipei	Cutibacterium granulosum	51	0,00000	37	0,12377
taipei	Micrococcus luteus	68	0,00000	27	0,16229
tokyo	Moraxella osloensis	4	0,21466	27	0,54145
tokyo	Pseudomonas fluorescens	5	0,10028	11	1,23242
tokyo	Acinetobacter baumannii	8	0,06761	3	4,00990
tokyo	Pseudomonas stutzeri	9	0,06002	13	1,17399
tokyo	Xanthomonas campestris	10	0,05559	12	1,18356
tokyo	Pseudomonas aeruginosa	13	0,02354	9	1,46390
tokyo	Klebsiella pneumoniae	14	0,02233	28	0,53080
tokyo	Escherichia coli	15	0,02135	37	0,41667
tokyo	Acinetobacter junii	18	0,01495	125	0,02945
tokyo	Salmonella enterica	20	0,00981	24	0,60081
tokyo	Rothia mucilaginosa	22	0,00799	124	0,03019
tokyo	Enterococcus faecalis	31	0,00342	56	0,22543
tokyo	Listeria monocytogenes	33	0,00175	196	0,00502
tokyo	Pseudomonas balearica	45	0,00000	33	0,45772
tokyo	Cutibacterium granulosum	55	0,00000	52	0,24005
tokyo	Micrococcus luteus	70	0,00000	19	0,78457
vienna	Moraxella osloensis	2	0,25280	53	0,09127
vienna	Pseudomonas aeruginosa	5	0,08412	2	7,68060
vienna	Pseudomonas fluorescens	6	0,05431	8	1,28066
vienna	Pseudomonas stutzeri	7	0,05053	10	1,02288
vienna	Xanthomonas campestris	10	0,02752	11	1,00213
vienna	Acinetobacter baumannii	13	0,01639	12	1,00032
vienna	Acinetobacter junii	14	0,01199	82	0,04164
vienna	Rothia mucilaginosa	19	0,00482	128	0,01392
vienna	Escherichia coli	20	0,00460	50	0,09660
vienna	Klebsiella pneumoniae	22	0,00375	29	0,22635

Table S4

vienna	Salmonella enterica	24	0,00261	28	0,23963
vienna	Listeria monocytogenes	33	0,00023	263	0,00000
vienna	Enterococcus faecalis	34	0,00023	91	0,03385
vienna	Pseudomonas balearica	45	0,00000	18	0,47791
vienna	Cutibacterium granulosum	53	0,00000	38	0,14770
vienna	Micrococcus luteus	69	0,00000	9	1,11670
zurich	Pseudomonas aeruginosa	7	0,02790	13	0,63036
zurich	Pseudomonas stutzeri	9	0,00703	10	0,72835
zurich	Rothia mucilaginosa	13	0,00544	109	0,01263
zurich	Xanthomonas campestris	14	0,00388	1	10,02636
zurich	Salmonella enterica	15	0,00385	32	0,17543
zurich	Escherichia coli	16	0,00265	44	0,08088
zurich	Pseudomonas fluorescens	18	0,00244	4	3,01900
zurich	Klebsiella pneumoniae	19	0,00153	37	0,13373
zurich	Acinetobacter baumannii	21	0,00093	36	0,13757
zurich	Moraxella osloensis	22	0,00049	115	0,01020
zurich	Listeria monocytogenes	23	0,00026	263	0,00000
zurich	Enterococcus faecalis	27	0,00015	98	0,01530
zurich	Acinetobacter junii	29	0,00009	243	0,00000
zurich	Pseudomonas balearica	48	0,00000	25	0,23320
zurich	Cutibacterium granulosum	56	0,00000	51	0,06798
zurich	Micrococcus luteus	70	0,00000	30	0,17632

Supplementary Table S5. Rank of PPG hosts predicted by both vHULK and CRISPR. Only PPGs with the same prediction in both approaches appear in this table.

City	Bacteria/ PPG Host	Bacteria abudance ranl	Bacteria abundance (%)	PPGs abudance ranl	PPGs abundance (%)
auckland	Pseudomonas aeruginosa	3	0,08573	2	0,96212
auckland	Acinetobacter baumanni	10	0,01772	7	0,14387
auckland	Klebsiella pneumoniae	11	0,01650	16	0,06934
auckland	Listeria monocytogenes	22	0,00102	31	0,00000
berlin	Pseudomonas aeruginosa	6	0,09053	3	1,79556
berlin	Acinetobacter baumanni	10	0,03613	6	0,57434
berlin	Klebsiella pneumoniae	17	0,01301	17	0,03999
berlin	Listeria monocytogenes	30	0,00210	34	0,00125
bogota	Acinetobacter baumanni	7	0,03153	3	1,38348
bogota	Pseudomonas aeruginosa	9	0,02862	5	0,67338
bogota	Klebsiella pneumoniae	10	0,02302	14	0,09483
bogota	Listeria monocytogenes	33	0,00128	32	0,00240
cairngorm	Pseudomonas aeruginosa	2	0,22953	4	0,69385
cairngorm	Listeria monocytogenes	4	0,16912	12	0,16780
cairngorm	Acinetobacter baumanni	18	0,00387	17	0,04704
cairngorm	Klebsiella pneumoniae	33	0,00054	18	0,00688
denver	Klebsiella pneumoniae	7	0,02088	11	0,04480
denver	Pseudomonas aeruginosa	8	0,01474	8	0,13303
denver	Acinetobacter baumanni	11	0,00956	9	0,11382
denver	Listeria monocytogenes	34	0,00057	29	0,00000
doha	Pseudomonas aeruginosa	2	3,99929	1	4,57110
doha	Listeria monocytogenes	14	0,02491	16	0,01216
doha	Acinetobacter baumanni	15	0,02114	13	0,06673
doha	Klebsiella pneumoniae	19	0,00830	15	0,04587
fairbanks	Klebsiella pneumoniae	10	0,04040	15	0,06206
fairbanks	Pseudomonas aeruginosa	16	0,01154	9	0,22009
fairbanks	Acinetobacter baumanni	18	0,00979	6	0,29207
fairbanks	Listeria monocytogenes	32	0,00072	36	0,00002
hamilton	Pseudomonas aeruginosa	4	0,08008	1	0,68051

Table S5

hamilton	Acinetobacter baumanni	5	0,06056	9	0,07794
hamilton	Klebsiella pneumoniae	11	0,03372	7	0,15391
hamilton	Listeria monocytogenes	22	0,00064	38	0,00000
hanoi	Acinetobacter baumanni	5	0,06655	5	0,39403
hanoi	Klebsiella pneumoniae	8	0,03840	7	0,15230
hanoi	Pseudomonas aeruginosa	10	0,01564	8	0,12179
hanoi	Listeria monocytogenes	32	0,00084	34	0,00000
hong_kong	Pseudomonas aeruginosa	4	0,07228	6	0,30082
hong_kong	Acinetobacter baumanni	8	0,02743	12	0,09828
hong_kong	Klebsiella pneumoniae	24	0,00467	14	0,02036
hong_kong	Listeria monocytogenes	34	0,00009	34	0,00006
ilorin	Klebsiella pneumoniae	3	0,20051	3	0,58563
ilorin	Pseudomonas aeruginosa	5	0,09112	1	4,89105
ilorin	Acinetobacter baumanni	7	0,06359	8	0,19310
ilorin	Listeria monocytogenes	18	0,00288	34	0,00030
kuala_lumpur	Pseudomonas aeruginosa	1	43,17741	1	38,35489
kuala_lumpur	Listeria monocytogenes	5	0,94545	3	0,38271
kuala_lumpur	Klebsiella pneumoniae	15	0,00076	6	0,12375
kuala_lumpur	Acinetobacter baumanni	30	0,00003	21	0,00009
kyiv	Klebsiella pneumoniae	2	1,24649	1	1,25038
kyiv	Acinetobacter baumanni	4	0,29822	10	0,26061
kyiv	Pseudomonas aeruginosa	7	0,06321	14	0,21806
kyiv	Listeria monocytogenes	30	0,00137	31	0,00001
lisbon	Acinetobacter baumanni	3	0,77363	3	1,70853
lisbon	Pseudomonas aeruginosa	6	0,10960	2	2,05801
lisbon	Klebsiella pneumoniae	9	0,02830	4	0,55244
lisbon	Listeria monocytogenes	22	0,00172	35	0,00000
london	Pseudomonas aeruginosa	1	5,04627	2	1,28101
london	Listeria monocytogenes	2	4,10031	10	0,24606
london	Acinetobacter baumanni	24	0,00409	16	0,16042
london	Klebsiella pneumoniae	32	0,00156	24	0,02350
marseille	Pseudomonas aeruginosa	6	0,05407	2	0,24244
marseille	Acinetobacter baumanni	9	0,03945	3	0,23120
marseille	Klebsiella pneumoniae	18	0,01504	9	0,05204

Table S5

marseille	Listeria monocytogenes	34	0,00074	33	0,00034
minneapolis	Pseudomonas aeruginosa	12	0,01170	5	0,11098
minneapolis	Listeria monocytogenes	26	0,00144	28	0,00025
minneapolis	Klebsiella pneumoniae	28	0,00079	14	0,00774
minneapolis	Acinetobacter baumanni	30	0,00030	19	0,00342
naples	Pseudomonas aeruginosa	9	0,02645	6	0,24586
naples	Acinetobacter baumanni	16	0,01183	5	0,53742
naples	Klebsiella pneumoniae	29	0,00173	14	0,01429
naples	Listeria monocytogenes	35	0,00016	30	0,00000
new_york_city	Acinetobacter baumanni	6	0,23944	7	0,58176
new_york_city	Pseudomonas aeruginosa	12	0,11191	2	1,32995
new_york_city	Listeria monocytogenes	19	0,02107	24	0,02067
new_york_city	Klebsiella pneumoniae	20	0,01869	13	0,18260
offa	Klebsiella pneumoniae	2	1,02931	3	0,98044
offa	Pseudomonas aeruginosa	5	0,08176	1	3,51296
offa	Acinetobacter baumanni	7	0,03776	9	0,13290
offa	Listeria monocytogenes	17	0,00224	33	0,00002
oslo	Pseudomonas aeruginosa	12	0,00931	8	0,15437
oslo	Acinetobacter baumanni	19	0,00416	10	0,06730
oslo	Klebsiella pneumoniae	26	0,00132	18	0,01013
oslo	Listeria monocytogenes	34	0,00019	36	0,00000
others	Pseudomonas aeruginosa	2	37,70298	1	11,53677
others	Listeria monocytogenes	5	12,24438	5	0,70122
others	Klebsiella pneumoniae	16	0,03380	14	0,03262
others	Acinetobacter baumanni	26	0,00379	16	0,00879
porto	Acinetobacter baumanni	10	0,02167	12	0,18334
porto	Pseudomonas aeruginosa	12	0,01560	4	0,67798
porto	Klebsiella pneumoniae	18	0,00859	16	0,01866
porto	Listeria monocytogenes	32	0,00033	32	0,00000
rio_de_janeiro	Acinetobacter baumanni	4	0,19590	1	1,84482
rio_de_janeiro	Klebsiella pneumoniae	12	0,02927	11	0,09067
rio_de_janeiro	Pseudomonas aeruginosa	14	0,02330	5	0,66934
rio_de_janeiro	Listeria monocytogenes	31	0,00241	36	0,00068
santiago	Pseudomonas aeruginosa	4	0,03697	6	0,57349

Table S5

santiago	Klebsiella pneumoniae	7	0,02410	15	0,04902
santiago	Acinetobacter baumanni	9	0,01892	2	1,46137
santiago	Listeria monocytogenes	32	0,00183	30	0,00238
sao paulo	Pseudomonas aeruginosa	6	0,34283	4	0,40723
sao paulo	Listeria monocytogenes	17	0,01690	17	0,00558
sao paulo	Klebsiella pneumoniae	22	0,00964	15	0,01702
sao paulo	Acinetobacter baumanni	23	0,00923	13	0,03691
seoul	Pseudomonas aeruginosa	3	0,06907	10	0,34839
seoul	Acinetobacter baumanni	11	0,01479	12	0,02562
seoul	Klebsiella pneumoniae	14	0,01280	14	0,00725
seoul	Listeria monocytogenes	31	0,00273	22	0,00115
singapore	Acinetobacter baumanni	4	0,26913	3	0,37242
singapore	Pseudomonas aeruginosa	10	0,06437	6	0,21671
singapore	Klebsiella pneumoniae	12	0,03731	7	0,15272
singapore	Listeria monocytogenes	35	0,00033	34	0,00000
sofia	Pseudomonas aeruginosa	12	0,03040	4	0,54884
sofia	Acinetobacter baumanni	16	0,02050	3	1,08383
sofia	Klebsiella pneumoniae	28	0,00429	11	0,06800
sofia	Listeria monocytogenes	35	0,00023	33	0,00044
taipei	Pseudomonas aeruginosa	5	0,04199	7	0,13700
taipei	Acinetobacter baumanni	16	0,00644	14	0,03088
taipei	Klebsiella pneumoniae	21	0,00201	17	0,00611
taipei	Listeria monocytogenes	34	0,00003	27	0,00008
tokyo	Acinetobacter baumanni	8	0,06761	3	1,71600
tokyo	Pseudomonas aeruginosa	13	0,02354	4	0,69752
tokyo	Klebsiella pneumoniae	14	0,02233	18	0,06942
tokyo	Listeria monocytogenes	33	0,00175	32	0,00155
vienna	Pseudomonas aeruginosa	5	0,08412	1	0,83098
vienna	Acinetobacter baumanni	13	0,01639	2	0,50323
vienna	Klebsiella pneumoniae	22	0,00375	16	0,02222
vienna	Listeria monocytogenes	33	0,00023	34	0,00000
zurich	Pseudomonas aeruginosa	7	0,02790	1	0,93058
zurich	Klebsiella pneumoniae	19	0,00153	13	0,01379
zurich	Acinetobacter baumanni	21	0,00093	4	0,07697

Table S5

zurich	Listeria monocytogenes	23	0,00026	35	0,00000

Table S6

Supplementary Table S6. Hosts present in the list of 75 most common bacteria of Danko et. al (2021).

Hosts in Danko et. al. (2021) list	# of PPGs assigned
Salmonella enterica	32
Pseudomonas aeruginosa	31
Klebsiella pneumoniae	30
Pseudomonas stutzeri	25
Acinetobacter baumannii	18
Pseudomonas balearica	18
Xanthomonas campestris	13
Escherichia coli	10
Enterococcus faecalis	9
Staphylococcus epidermidis	8
Staphylococcus aureus	7
Pseudomonas fluorescens	6
Micrococcus luteus	6
Moraxella osloensis	5
Listeria monocytogenes	3
Cutibacterium granulosum	2
Acinetobacter junii	2
Stenotrophomonas maltophilia	1
Rothia mucilaginosa	1
Roseomonas sp.	1

Supplementary Table S7. CRISPR-Cas system found in PPGs. Only predictions with CRISPR spacers evidence level above 3 were considered. The completeness, putative host (vHULK and CRISPR species) and lifestyle were shown.

PPG	CRISPR array(s) [coordenates]	Evidence Level	Cas Types/Subtypes [coordenates]	PPG Size (bp)	CheckV Quality	CRISPR Species prediction	vHULK Species prediction	CheckV Lifestyle	Bacphlip Lifestyle	Vibrant Lifestyle
MSP0016	MSP0016_1 [97520;100773]	2		250778	Complete			virulent/lytic	prophage/temperate/lysogenic	prophage/temperate/lysogenic
MSP0034	MSP0034_1 [6741;7097]	3	CAS-TypeIB [7235;14811]	212281	High-quality	Clostridium botulinum		virulent/lytic	prophage/temperate/lysogenic	
MSP0217	MSP0217_1 [54461;55156]	4	CAS-TypeIIA [48222;54440]	110983	Medium-quality			prophage/temperate/lysogenic	prophage/temperate/lysogenic	prophage/temperate/lysogenic
MSP0656	MSP0656_1 [43012;43301]	2		66247	Medium-quality	Bacillus halodurans	Staphylococcus aureus	prophage/temperate/lysogenic	prophage/temperate/lysogenic	prophage/temperate/lysogenic
MSP0734	MSP0734_1 [134;461]	4		62834	High-quality	Moraxella osloensis		prophage/temperate/lysogenic	prophage/temperate/lysogenic	prophage/temperate/lysogenic
MSP1038	MSP1038_1 [31669;34626]	4	CAS-TypeIE [34691;39020]	52976	Medium-quality	Paracoccus yeei		prophage/temperate/lysogenic	prophage/temperate/lysogenic	prophage/temperate/lysogenic
MSP1127	MSP1127_1 [6679;6888]	2		50550	Low-quality	Propionibacterium freudenreichii		prophage/temperate/lysogenic	prophage/temperate/lysogenic	prophage/temperate/lysogenic
MSP1562	MSP1562_1 [23846;31485]	4	CAS-TypeIC [37143;37120]	43836	Not-determined			virulent/lytic	prophage/temperate/lysogenic	
MSP1816	MSP1816_1 [702;4572]	4	CAS-TypeIF [10;537]	41124	Medium-quality	Moraxella osloensis		prophage/temperate/lysogenic	prophage/temperate/lysogenic	prophage/temperate/lysogenic

Table S8

Supplementary Table S8. Most abundant PPG per sequenced sample.

Samples	PPG	Abudance (%)	City
HMCMJCCXY_SL335787	MSP0350	98,03	Fairbanks
HMCMJCCXY_SL335821	MSP1223	89,33	Fairbanks
HMC2KCCXY_SL336697	MSP0887	87,37	Hong Kong
HMGW3CCXY_SL342609	MSP1584	85,31	Tokyo
HMC2KCCXY_SL336764	MSP0887	83,81	Hong Kong
H75CGCCXY_SL263638	MSP0030	82,74	Auckland
HMCMJCCXY_SL335834	MSP1494	78,63	Fairbanks
HNHKFCCXY_SL345575	MSP1878	77,45	Hong Kong
HMC2KCCXY_SL336755	MSP0887	73,94	Hong Kong
HNHKFCCXY_SL346098	MSP1228	71,50	Hong Kong
HMC2KCCXY_SL336804	MSP0212	69,79	Hong Kong
HMC2KCCXY_SL336626	MSP1840	69,65	Hong Kong
HMGW3CCXY_SL342349	MSP0896	69,04	Marseille
HCY5JCCXY_SL269589	MSP0322	67,74	New York City
HMGN5CCXY_SL344637	MSP1782	67,34	Ilorin
HMC2KCCXY_SL336564	MSP0887	67,17	Hong Kong
HMCMJCCXY_SL335857	MSP1010	65,79	Fairbanks
HCV72CCXY_SL269738	MSP0197	65,19	New York City
HMGN5CCXY_SL344676	MSP0070	65,14	Ilorin
H3MGVCCXY_SL259989	MSP0186	62,53	--
HCY5HCCXY_SL270918	MSP0043	62,48	London
HCCGHCCXY_SL270433	MSP0303	62,42	London
HT5YVCCXY_SL357341	MSP1428	61,49	Taipei
HMCMJCCXY_SL335774	MSP0817	61,33	Fairbanks
HNHKFCCXY_SL346119	MSP1612	59,02	Hong Kong
HMCMJCCXY_SL335935	MSP0825	57,64	Lisbon
HMCMJCCXY_SL335869	MSP1122	57,54	Doha

Table S8

HMGTJCCXY_SL342628	MSP1207	57,29	Zurich
HT5YVCCXY_SL357315	MSP1109	56,14	Taipei
HMC2KCCXY_SL336773	MSP0887	55,47	Hong Kong
H3KHWCCXY_SL263333	MSP0694	53,69	Porto
H75CGCCXY_SL263662	MSP1220	52,54	Auckland
HCY5JCCXY_SL269547	MSP1857	50,84	New York City
HMC2KCCXY_SL336743	MSP0887	50,13	Hong Kong
HCY5HCCXY_SL270926	MSP1857	47,74	London
HMGW3CCXY_SL342282	MSP1647	46,39	Vienna
HNHKFCCXY_SL345981	MSP1612	45,97	Hong Kong
HCV72CCXY_SL269800	MSP1193	44,03	New York City
HCCGHCCXY_SL270547	MSP1621	43,32	London
HMC2KCCXY_SL336783	MSP0887	42,36	Hong Kong
H2NYMCCXY_SL254828	MSP0965	40,96	Fairbanks
HCV72CCXY_SL269768	MSP0972	40,95	New York City
HNHKFCCXY_SL345455	MSP1354	39,80	Hong Kong
H7HJMCCXY_SL272809	MSP1193	39,30	Lisbon
HCCGHCCXY_SL270289	MSP1670	39,21	London
H3MGVCCXY_SL259917	MSP1182	38,57	New York City
HMGN5CCXY_SL344653	MSP0317	38,46	Ilorin
HKC32ALXX_SL254693	MSP0038	37,75	Ilorin
HCV72CCXY_SL269721	MSP1175	37,53	New York City
HMGW3CCXY_SL342350	MSP0320	36,42	Marseille
HCY5JCCXY_SL269554	MSP1611	35,72	New York City
HMCMJCCXY_SL335859	MSP1802	35,25	Fairbanks
HMGW3CCXY_SL342470	MSP1876	34,27	Minneapolis
HMCMJCCXY_SL336349	MSP0590	34,18	Taipei
HMC2KCCXY_SL336828	MSP1876	34,01	Hong Kong
HMC2KCCXY_SL336681	MSP1612	33,94	Hong Kong
HNGH3CCXY_SL344537	MSP1302	33,94	Ilorin

Table S8

HMC2KCCXY_SL336679	MSP1612	33,81	Hong Kong
HCV72CCXY_SL269788	MSP0669	33,52	New York City
HCV72CCXY_SL269782	MSP1193	33,45	New York City
HMGMHCCXY_SL343354	MSP1228	33,35	Singapore
H2NYMCCXY_SL254815	MSP0133	33,10	Rio De Janeiro
HT5YVCCXY_SL357286	MSP0936	33,04	Taipei
HMCMJCCXY_SL335963	MSP1708	33,03	Lisbon
H2NYMCCXY_SL254824	MSP1729	32,95	Rio De Janeiro
H75CGCCXY_SL263639	MSP0600	32,44	Hamilton
HMCMJCCXY_SL335942	MSP1193	32,09	Lisbon
HCVMTCCXY_SL269554	MSP1611	31,74	New York City
HCCGHCCXY_SL270294	MSP0244	31,55	London
HCY5JCCXY_SL269595	MSP0676	31,36	New York City
HNGH3CCXY_SL344549	MSP0777	31,27	Ilorin
HMGW3CCXY_SL342530	MSP1876	31,24	Hong Kong
HMGW3CCXY_SL342278	MSP0821	30,96	Vienna
HNHKFCCXY_SL346109	MSP1612	30,67	Hong Kong
H7HJMCCXY_SL272782	MSP1512	30,58	Ilorin
H7KYMCCXY_SL273103	MSP0975	30,29	Fairbanks
HMC2KCCXY_SL336578	MSP1612	30,28	Hong Kong
HMCMJCCXY_SL335936	MSP1214	30,08	Lisbon
H2NYMCCXY_SL254804	MSP0965	29,97	Fairbanks
HCV72CCXY_SL269722	MSP1219	29,82	New York City
HMGW3CCXY_SL342525	MSP1876	29,60	Hong Kong
HMC2KCCXY_SL336736	MSP1497	29,50	Hong Kong
HCY5JCCXY_SL269596	MSP1639	29,32	New York City
H2NYMCCXY_SL254825	MSP1660	29,15	Rio De Janeiro
HCVMTCCXY_SL269605	MSP1008	29,07	New York City
HMCMJCCXY_SL335925	MSP1345	29,06	Doha
H7HJMCCXY_SL272755	MSP1268	28,28	Ilorin

Table S8

H7KYMCCXY_SL273087	MSP1016	28,15	Fairbanks
H2NYMCCXY_SL254780	MSP1043	28,11	Fairbanks
HMGW3CCXY_SL342430	MSP1839	27,84	London
HCY5HCCXY_SL270936	MSP0273	27,82	London
H3MGVCCXY_SL259921	MSP1907	27,79	Other Control
HMCMJCCXY_SL335845	MSP1223	27,74	Fairbanks
H3MGVCCXY_SL259966	MSP0186	27,64	Minneapolis
HCV72CCXY_SL269734	MSP1219	27,39	New York City
H3MGVCCXY_SL259953	MSP1907	26,79	--
H7HJMCCXY_SL272745	MSP1515	26,68	Ilorin
H3MGVCCXY_SL259846	MSP1612	26,63	New York City
HT5YVCCXY_SL357298	MSP1907	26,55	Taipei
HCV72CCXY_SL269799	MSP1193	26,54	New York City
HNHKFCCXY_SL345483	MSP1673	26,50	Hong Kong
H2NYMCCXY_SL254773	MSP1460	26,45	New York City
HNHKFCCXY_SL345590	MSP0546	26,35	Hong Kong
H75CGCCXY_SL263637	MSP0167	26,30	Auckland
HMGW3CCXY_SL342529	MSP1876	26,30	Hong Kong
HKC32ALXX_SL254746	MSP1359	26,22	Bogota
H3MGVCCXY_SL259967	MSP0236	26,13	Tokyo
HMGN5CCXY_SL344674	MSP0717	26,05	Ilorin
H75CGCCXY_SL263664	MSP0917	25,81	Hamilton
HCV72CCXY_SL269806	MSP1008	25,54	New York City
H3MGVCCXY_SL259985	MSP1907	25,53	Sao Paulo
HNHKFCCXY_SL345980	MSP1800	25,50	Hong Kong
HMCMJCCXY_SL335833	MSP1664	25,46	Fairbanks
HKC32ALXX_SL254763	MSP1672	25,44	Ilorin
HKC32ALXX_SL254675	MSP1156	25,40	Bogota
HMGMHCCXY_SL343467	MSP1434	25,37	Kyiv
HMGW3CCXY_SL342337	MSP0782	25,33	Naples

Table S8

HKC32ALXX_SL254706	MSP1355	25,26	Ilorin
HMGN5CCXY_SL344702	MSP0437	25,04	Ilorin
HCY5JCCXY_SL269608	MSP0306	24,83	New York City
HMC2KCCXY_SL336650	MSP1612	24,76	Hong Kong
HCY5HCCXY_SL270960	MSP1406	24,44	London
HCVMTCCXY_SL269603	MSP1393	24,30	New York City
HCY5JCCXY_SL269611	MSP0143	24,27	New York City
H3MGVCCXY_SL259912	MSP1612	23,94	New York City
HMGW3CCXY_SL342474	MSP1876	23,77	Minneapolis
H2NYMCCXY_SL254836	MSP1612	23,74	Rio De Janeiro
HT5YVCCXY_SL357401	MSP1907	23,54	Singapore
H3MGVCCXY_SL259963	MSP0750	23,51	--
H75CGCCXY_SL263649	MSP0977	23,46	Auckland
H7KYMCCXY_SL273105	MSP1081	23,38	Fairbanks
HNHKFCCXY_SL345480	MSP1673	23,23	Hong Kong
HCCGHCCXY_SL270477	MSP0820	23,15	London
HNHKFCCXY_SL345930	MSP0433	22,91	Hong Kong
HCV72CCXY_SL269798	MSP1664	22,90	New York City
H7KYMCCXY_SL273127	MSP0656	22,34	Fairbanks
HCY5JCCXY_SL269590	MSP1019	22,30	New York City
HNHKFCCXY_SL346127	MSP1876	22,27	Hong Kong
H75CGCCXY_SL263723	MSP0743	22,25	Hamilton
H7KYMCCXY_SL273060	MSP0003	22,23	Fairbanks
HMC2KCCXY_SL336644	MSP1612	22,16	Hong Kong
HCV72CCXY_SL269784	MSP1436	22,12	New York City
HCY5JCCXY_SL269535	MSP0765	22,00	New York City
HCY5HCCXY_SL270914	MSP0765	21,96	London
HCVMTCCXY_SL269535	MSP0765	21,75	New York City
HT5YVCCXY_SL357297	MSP0936	21,60	Taipei
H7KYMCCXY_SL273121	MSP0473	21,52	Fairbanks

Table S8

H75CGCCXY_SL263712	MSP0165	21,50	Hamilton
HCY5JCCXY_SL269557	MSP0273	21,18	New York City
H2NYMCCXY_SL254770	MSP1166	21,15	Offa
HMC2KCCXY_SL336808	MSP1034	21,10	Hong Kong
HK7G5CCXY_SL310981	MSP1193	21,09	Hong Kong
HCCGHCCXY_SL270290	MSP1612	20,68	London
HMCMJCCXY_SL335820	MSP1788	20,45	Fairbanks
H7HJMCCXY_SL272753	MSP0454	20,44	Ilorin
H2NYMCCXY_SL254851	MSP1691	20,30	Fairbanks
HCY5JCCXY_SL269571	MSP1541	20,27	New York City
H7HJMCCXY_SL272820	MSP0715	20,20	Lisbon
HCY5JCCXY_SL269530	MSP1463	20,17	New York City
H3MGVCCXY_SL259975	MSP1545	20,15	Minneapolis
HCY5HCCXY_SL270958	MSP1807	20,09	London
HMCMJCCXY_SL335844	MSP1374	20,09	Fairbanks
HMCMJCCXY_SL335770	MSP0655	20,09	Denver
HCV72CCXY_SL269759	MSP0301	19,98	New York City
H3MGVCCXY_SL259974	MSP0330	19,93	Other Control
H7HJMCCXY_SL272744	MSP0735	19,82	Ilorin
HCVMTCCXY_SL269541	MSP0660	19,80	New York City
H3MGVCCXY_SL259906	MSP1907	19,75	New York City
H3MGVCCXY_SL259990	MSP0330	19,66	Other Control
HCV72CCXY_SL269766	MSP1372	19,59	New York City
HCY5HCCXY_SL270916	MSP1664	19,40	London
HKC32ALXX_SL254679	MSP0848	19,35	Tokyo
HMCMJCCXY_SL336225	MSP0760	19,32	Hanoi
HMGW3CCXY_SL342540	MSP1876	19,30	Hong Kong
H2NYMCCXY_SL254860	MSP0831	18,90	Rio De Janeiro
H7HJMCCXY_SL272778	MSP1522	18,76	Ilorin
HK7G5CCXY_SL310979	MSP1193	18,65	Hong Kong

Table S8

H7HJMCCXY_SL272821	MSP1706	18,59	Lisbon
HKC32ALXX_SL254681	MSP1346	18,58	Ilorin
HCVMTCCXY_SL269545	MSP0312	18,54	New York City
HCY5JCCXY_SL269569	MSP0919	18,45	New York City
HCV72CCXY_SL269769	MSP1664	18,38	New York City
HKC32ALXX_SL254700	MSP1612	18,32	Bogota
H3MGVCCXY_SL259923	MSP1907	18,23	Sao Paulo
HCY5HCCXY_SL270924	MSP0312	18,15	London
HCY5HCCXY_SL270959	MSP0898	18,08	London
HMC2KCCXY_SL336627	MSP0376	18,07	Hong Kong
HMGTJCCXY_SL342608	MSP1612	18,05	Tokyo
HMCMJCCXY_SL335773	MSP0408	17,99	Fairbanks
HKC32ALXX_SL254762	MSP0848	17,99	Tokyo
HCV72CCXY_SL269718	MSP1105	17,98	New York City
HCY5JCCXY_SL269579	MSP1807	17,90	New York City
HCVMTCCXY_SL269607	MSP0400	17,71	New York City
H7HJMCCXY_SL272766	MSP1770	17,68	Ilorin
H2NYMCCXY_SL254819	MSP1080	17,56	Offa
HKC32ALXX_SL254711	MSP1612	17,54	Bogota
H7HJMCCXY_SL272747	MSP1080	17,46	Ilorin
HMGMHCCXY_SL343483	MSP0734	17,40	Kyiv
HMCMJCCXY_SL335948	MSP0215	17,38	Lisbon
HCV72CCXY_SL269733	MSP1392	17,29	New York City
H3MGVCCXY_SL259857	MSP0847	17,28	New York City
HKC32ALXX_SL254725	MSP1612	16,88	Tokyo
H7KYMCCXY_SL273071	MSP1441	16,80	Fairbanks
HCY5HCCXY_SL270937	MSP1393	16,68	London
H7HJMCCXY_SL272786	MSP0934	16,65	Ilorin
HMCMJCCXY_SL335786	MSP0111	16,60	Fairbanks
HCVMTCCXY_SL269565	MSP0907	16,60	New York City

Table S8

HCY5JCCXY_SL269565	MSP1646	16,53	New York City
HNGH3CCXY_SL344575	MSP0518	16,40	Ilorin
HCV72CCXY_SL269771	MSP0279	16,37	New York City
H7HJMCCXY_SL272822	MSP1080	16,24	Lisbon
HKC32ALXX_SL254712	MSP0049	16,12	Bogota
HK7G5CCXY_SL310962	MSP1124	16,04	Hong Kong
H3MGVCCXY_SL259862	MSP1612	15,96	New York City
HMCMJCCXY_SL335938	MSP1854	15,89	Lisbon
HMCMJCCXY_SL336180	MSP1083	15,85	Tokyo
HCCGHCCXY_SL270293	MSP1879	15,84	London
HCY5HCCXY_SL270984	MSP1008	15,84	London
HNHKFCCXY_SL345946	MSP1709	15,81	Hong Kong
HCVMTCCXY_SL269582	MSP1289	15,80	New York City
H75CGCCXY_SL263686	MSP1645	15,67	Auckland
HMGW3CCXY_SL342294	MSP1881	15,63	Sofia
HCV72CCXY_SL269767	MSP1697	15,58	New York City
H75CGCCXY_SL263650	MSP0158	15,55	Auckland
HCV72CCXY_SL269776	MSP0388	15,51	New York City
HCY5JCCXY_SL269573	MSP0123	15,40	New York City
HCY5HCCXY_SL270975	MSP1639	15,39	London
HKC32ALXX_SL254684	MSP1612	15,22	Berlin
HCY5HCCXY_SL270952	MSP0123	15,00	London
HCVMTCCXY_SL269612	MSP1372	14,96	New York City
HCV72CCXY_SL269755	MSP0972	14,96	New York City
H75CGCCXY_SL263699	MSP0668	14,94	Hamilton
H7HJMCCXY_SL272808	MSP0428	14,89	Lisbon
HMC2KCCXY_SL336590	MSP1876	14,89	Hong Kong
H2NYMCCXY_SL254847	MSP1566	14,88	Rio De Janeiro
HCVMTCCXY_SL269573	MSP0123	14,80	New York City
HCVMTCCXY_SL269571	MSP1541	14,79	New York City

Table S8

H7HJMCCXY_SL272784	MSP0368	14,73	Ilorin
HKC32ALXX_SL254688	MSP1612	14,72	Bogota
HCVMTCCXY_SL269542	MSP0123	14,70	New York City
HCV72CCXY_SL269792	MSP0549	14,70	New York City
HKC32ALXX_SL254722	MSP1472	14,65	Bogota
HK7G5CCXY_SL310952	MSP1193	14,63	Hong Kong
HKC32ALXX_SL254694	MSP0150	14,63	Ilorin
HCY5HCCXY_SL270986	MSP0400	14,62	London
H3MGVCCXY_SL259987	MSP1545	14,62	Other Control
HCY5HCCXY_SL270961	MSP1289	14,55	London
H3MGVCCXY_SL259835	MSP1128	14,48	New York City
HMC2KCCXY_SL336784	MSP1876	14,45	Hong Kong
HKC32ALXX_SL254749	MSP1612	14,35	Tokyo
H3MGVCCXY_SL259838	MSP1193	14,31	New York City
HMGN5CCXY_SL344649	MSP1371	14,13	Ilorin
HCV72CCXY_SL269752	MSP0633	13,96	New York City
H7HJMCCXY_SL272803	MSP0693	13,95	Ilorin
HCY5JCCXY_SL269570	MSP0250	13,93	New York City
HMGW3CCXY_SL342298	MSP1543	13,89	Sofia
H7HJMCCXY_SL272790	MSP0624	13,88	Ilorin
HMCMJCCXY_SL335957	MSP0168	13,87	Lisbon
HNGH3CCXY_SL344536	MSP0706	13,85	Ilorin
HCV72CCXY_SL269793	MSP0026	13,84	New York City
HMCMJCCXY_SL335961	MSP1588	13,84	Lisbon
HMGW3CCXY_SL342295	MSP1650	13,83	Sofia
HKC32ALXX_SL254742	MSP0230	13,81	Ilorin
HCV72CCXY_SL269777	MSP0924	13,74	New York City
HMCMJCCXY_SL335960	MSP1084	13,64	Lisbon
HCVMTCCXY_SL269561	MSP0606	13,63	New York City
H2NYMCCXY_SL254777	MSP1612	13,59	Rio De Janeiro

Table S8

H7KYMCCXY_SL273067	MSP1613	13,44	Fairbanks
HNHKFCCXY_SL345508	MSP0845	13,42	Hong Kong
H3MGVCCXY_SL259882	MSP0016	13,34	New York City
H2NYMCCXY_SL254832	MSP0428	13,33	New York City
HCCGHCCXY_SL270210	MSP0557	13,33	London
H7HJMCCXY_SL272787	MSP0075	13,23	Ilorin
HCV72CCXY_SL269786	MSP0972	13,22	New York City
HMCMJCCXY_SL335952	MSP0078	13,20	Lisbon
HCVMTCCXY_SL269597	MSP0276	13,19	New York City
H2NYMCCXY_SL254790	MSP0472	13,19	Rio De Janeiro
H7HJMCCXY_SL272762	MSP0058	13,10	Ilorin
H3MGVCCXY_SL259896	MSP1907	13,09	New York City
H7HJMCCXY_SL272828	MSP0163	13,01	Lisbon
HCV72CCXY_SL269743	MSP0386	12,97	New York City
HK7G5CCXY_SL311017	MSP1193	12,89	Oslo
HCY5HCCXY_SL270909	MSP1463	12,79	London
HCY5JCCXY_SL269561	MSP0606	12,78	New York City
HCY5HCCXY_SL270945	MSP0991	12,59	London
H2NYMCCXY_SL254857	MSP1612	12,59	New York City
H7KYMCCXY_SL273056	MSP1390	12,52	Berlin
H75CGCCXY_SL263721	MSP1286	12,51	Auckland
H7HJMCCXY_SL272749	MSP1459	12,49	Ilorin
HCV72CCXY_SL269772	MSP1820	12,30	New York City
H75CGCCXY_SL263685	MSP1691	12,23	Auckland
HCY5HCCXY_SL270976	MSP0276	12,22	London
HCV72CCXY_SL269754	MSP0431	12,19	New York City
HCVMTCCXY_SL269566	MSP0991	12,18	New York City
H75CGCCXY_SL263703	MSP0893	12,16	Porto
HCY5HCCXY_SL270932	MSP1393	12,13	London
HMC2KCCXY_SL336593	MSP1876	12,12	Hong Kong

Table S8

HCV72CCXY_SL269765	MSP0342	12,09	New York City
HMC2KCCXY_SL336638	MSP1914	12,09	Hong Kong
H7KYMCCXY_SL273082	MSP0948	12,02	Fairbanks
HMCMJCCXY_SL335797	MSP1429	11,93	Fairbanks
HKC32ALXX_SL254707	MSP1612	11,89	Berlin
H7HJMCCXY_SL272746	MSP0061	11,79	Ilorin
H7HJMCCXY_SL272830	MSP1615	11,77	Other Control
H7VL7CCXY_SL267266	MSP1876	11,72	Cairngorm
H2NYMCCXY_SL254850	MSP1221	11,64	Rio De Janeiro
HCY5JCCXY_SL269601	MSP0772	11,56	New York City
HK7G5CCXY_SL311022	MSP1501	11,50	Oslo
H2NYMCCXY_SL254837	MSP0428	11,45	Rio De Janeiro
H2NYMCCXY_SL254809	MSP1193	11,33	New York City
HMGTJCCXY_SL342642	MSP1243	11,28	Zurich
H2NYMCCXY_SL254787	MSP1425	11,15	New York City
H7HJMCCXY_SL272789	MSP0075	11,13	Ilorin
HKC32ALXX_SL254758	MSP0980	11,11	Bogota
H75CGCCXY_SL263724	MSP0409	11,08	Hamilton
H2NYMCCXY_SL254805	MSP1738	11,07	Offa
H7HJMCCXY_SL272752	MSP0407	11,06	Ilorin
H7HJMCCXY_SL272764	MSP1861	11,05	Ilorin
H75CGCCXY_SL263725	MSP0409	10,99	New York City
HCY5JCCXY_SL269586	MSP1253	10,99	New York City
HCY5JCCXY_SL269551	MSP0972	10,90	New York City
HCY5JCCXY_SL269583	MSP1884	10,86	New York City
H75CGCCXY_SL263647	MSP0428	10,86	Santiago
HKC32ALXX_SL254705	MSP0908	10,86	Ilorin
HCY5JCCXY_SL269610	MSP1276	10,86	New York City
HCY5HCCXY_SL270923	MSP1907	10,78	London
HMCMJCCXY_SL335819	MSP0458	10,76	New York City

Table S8

H7HJMCCXY_SL272792	MSP1021	10,69	Ilorin
HKC32ALXX_SL254717	MSP0315	10,66	Ilorin
H2NYMCCXY_SL254820	MSP0288	10,66	New York City
HMCMJCCXY_SL335912	MSP1907	10,62	Doha
HCV72CCXY_SL269740	MSP0123	10,61	New York City
HKC32ALXX_SL254676	MSP0569	10,57	Bogota
HCY5HCCXY_SL270908	MSP1091	10,56	London
HCVMTCCXY_SL269529	MSP1091	10,56	New York City
HKC32ALXX_SL254728	MSP0377	10,56	Ilorin
HCY5HCCXY_SL270983	MSP1789	10,51	London
H7HJMCCXY_SL272797	MSP0058	10,48	Ilorin
HNHKFCCXY_SL346051	MSP1876	10,36	Seoul
HCVMTCCXY_SL269604	MSP1789	10,29	New York City
HNHKFCCXY_SL345904	MSP1612	10,26	Hong Kong
H75CGCCXY_SL263675	MSP0687	10,22	Hamilton
H75CGCCXY_SL263666	MSP0554	10,20	New York City
H2NYMCCXY_SL254852	MSP1613	10,17	Fairbanks
H7HJMCCXY_SL272779	MSP0315	10,15	Ilorin
H7HJMCCXY_SL272771	MSP1080	10,15	Ilorin
H2NYMCCXY_SL254781	MSP0362	10,13	Offa
H75CGCCXY_SL263653	MSP0380	10,12	New York City
HMCMJCCXY_SL335939	MSP0554	10,12	Lisbon
HCV72CCXY_SL269805	MSP1098	10,11	New York City
H75CGCCXY_SL263654	MSP0315	10,09	New York City
H7HJMCCXY_SL272824	MSP1869	10,08	Lisbon
HMGTJCCXY_SL342622	MSP0750	10,03	Kuala Lumpur
HKC32ALXX_SL254752	MSP0058	10,02	Ilorin
HCY5HCCXY_SL271111	MSP1907	10,02	London
H3MGVCCXY_SL259929	MSP1024	9,94	Other Control
H2NYMCCXY_SL254856	MSP0428	9,92	New York City

Table S8

HCY5HCCXY_SL270989	MSP0273	9,90	London
HCVMTCCXY_SL269601	MSP0772	9,89	New York City
H75CGCCXY_SL263640	MSP0260	9,78	Hamilton
HCV72CCXY_SL269742	MSP0315	9,75	New York City
HKC32ALXX_SL254685	MSP1907	9,73	Santiago
HCY5JCCXY_SL269560	MSP0533	9,71	New York City
H2NYMCCXY_SL254827	MSP1869	9,69	Rio De Janeiro
H7HJMCCXY_SL272772	MSP0058	9,68	Ilorin
H2NYMCCXY_SL254848	MSP0198	9,66	Rio De Janeiro
HCY5HCCXY_SL271171	MSP1907	9,66	London
HMCMJCCXY_SL335954	MSP0315	9,65	Lisbon
HCY5HCCXY_SL270948	MSP0919	9,64	London
HK7G5CCXY_SL311016	MSP1193	9,61	Oslo
H2NYMCCXY_SL254817	MSP0058	9,59	Offa
H75CGCCXY_SL263688	MSP1696	9,58	Hamilton
HCVMTCCXY_SL269619	MSP0400	9,56	New York City
HCY5JCCXY_SL269566	MSP0991	9,55	New York City
HCV72CCXY_SL269749	MSP0213	9,50	New York City
HCVMTCCXY_SL269610	MSP0273	9,49	New York City
H75CGCCXY_SL263702	MSP0961	9,46	New York City
H7HJMCCXY_SL272802	MSP0640	9,45	Ilorin
HMCMJCCXY_SL335795	MSP0642	9,35	New York City
HK7G5CCXY_SL311009	MSP1193	9,34	Hong Kong
HCY5HCCXY_SL271110	MSP1393	9,33	London
H2NYMCCXY_SL254846	MSP0428	9,29	New York City
H7HJMCCXY_SL272823	MSP0458	9,29	Lisbon
H2NYMCCXY_SL254782	MSP1827	9,26	Offa
HCV72CCXY_SL269731	MSP0915	9,26	New York City
HCVMTCCXY_SL269574	MSP1372	9,22	New York City
HNHKFCCXY_SL345538	MSP1893	9,22	Hong Kong

Table S8

H7KYMCCXY_SL273090	MSP1243	9,21	Berlin
HCY5HCCXY_SL270934	MSP0196	9,21	London
H2NYMCCXY_SL254841	MSP0315	9,14	Offa
H2NYMCCXY_SL254844	MSP1308	9,11	New York City
H7HJMCCXY_SL272799	MSP1164	9,08	Ilorin
HCY5JCCXY_SL269526	MSP0850	9,07	New York City
H2NYMCCXY_SL254778	MSP0133	9,05	Rio De Janeiro
HCV72CCXY_SL269785	MSP0527	9,02	New York City
HCCGHCCXY_SL270411	MSP1612	9,00	London
HCY5JCCXY_SL269600	MSP0928	8,95	New York City
HMGW3CCXY_SL342603	MSP0869	8,91	Tokyo
H7KYMCCXY_SL273035	MSP1809	8,87	Fairbanks
H7HJMCCXY_SL272777	MSP0061	8,85	Ilorin
HCVMTCCXY_SL269526	MSP0850	8,84	New York City
HCY5JCCXY_SL269536	MSP0799	8,79	New York City
HNHKFCCXY_SL345993	MSP1088	8,70	Hong Kong
HCY5JCCXY_SL269527	MSP0155	8,69	New York City
HMCMJCCXY_SL335843	MSP0458	8,68	New York City
HCY5JCCXY_SL269599	MSP0448	8,64	New York City
H3KHWCCXY_SL263369	MSP1514	8,63	Porto
HCV72CCXY_SL269757	MSP0113	8,60	New York City
HKC32ALXX_SL254716	MSP1080	8,60	Ilorin
HCVMTCCXY_SL269538	MSP0197	8,60	New York City
H75CGCCXY_SL263651	MSP0771	8,59	Hamilton
HKC32ALXX_SL254698	MSP1876	8,58	Santiago
H7HJMCCXY_SL272814	MSP1918	8,55	Lisbon
H7HJMCCXY_SL272806	MSP0315	8,52	Lisbon
H75CGCCXY_SL263700	MSP1102	8,48	Hamilton
HCV72CCXY_SL269761	MSP1173	8,48	New York City
HMGMHCCXY_SL343453	MSP1876	8,48	Kyiv

Table S8

H3KHWCCXY_SL263315	MSP0389	8,45	Porto
H7HJMCCXY_SL272816	MSP0756	8,43	Lisbon
H7HJMCCXY_SL272788	MSP0058	8,40	Ilorin
HKC32ALXX_SL254740	MSP0058	8,39	Ilorin
HMCMJCCXY_SL335783	MSP0314	8,37	New York City
HCVMTCCXY_SL269600	MSP0928	8,35	New York City
H75CGCCXY_SL263677	MSP1805	8,34	New York City
HKC32ALXX_SL254718	MSP0315	8,32	Ilorin
HMCMJCCXY_SL335950	MSP1421	8,29	Lisbon
HMCMJCCXY_SL335888	MSP1904	8,24	Doha
HKC32ALXX_SL254699	MSP1612	8,24	Bogota
HMCMJCCXY_SL335741	MSP0686	8,22	Control
HCVMTCCXY_SL269599	MSP0448	8,21	New York City
HCY5HCCXY_SL270978	MSP0448	8,20	London
HCY5HCCXY_SL271138	MSP1907	8,20	London
HCVMTCCXY_SL269527	MSP0160	8,16	New York City
HMCMJCCXY_SL335807	MSP0428	8,16	New York City
H7HJMCCXY_SL272743	MSP0061	8,15	Ilorin
HMGW3CCXY_SL342291	MSP0750	8,14	Doha
HMCMJCCXY_SL336172	MSP0956	8,11	Tokyo
H75CGCCXY_SL263687	MSP0250	8,09	Hamilton
HCY5JCCXY_SL269564	MSP0758	8,07	New York City
HCV72CCXY_SL269751	MSP0375	8,04	New York City
HCVMTCCXY_SL269617	MSP0750	8,03	New York City
HCVMTCCXY_SL269559	MSP0903	8,03	New York City
H75CGCCXY_SL263678	MSP0791	7,99	New York City
H75CGCCXY_SL263726	MSP0078	7,99	New York City
HCY5HCCXY_SL270938	MSP0903	7,92	London
H2NYMCCXY_SL254810	MSP0428	7,87	New York City
H7HJMCCXY_SL272756	MSP0739	7,84	Ilorin

Table S8

HCY5HCCXY_SL270906	MSP0155	7,81	London
HMCMJCCXY_SL335794	MSP0642	7,78	New York City
HCV72CCXY_SL269750	MSP1389	7,75	New York City
HCY5HCCXY_SL270917	MSP0197	7,66	London
HMCMJCCXY_SL335956	MSP0334	7,65	Lisbon
HNHKFCCXY_SL345969	MSP1612	7,59	Hong Kong
H2NYMCCXY_SL254779	MSP1510	7,58	Rio De Janeiro
HCVMTCCXY_SL269564	MSP0758	7,54	New York City
H2NYMCCXY_SL254807	MSP0061	7,52	Offa
H7HJMCCXY_SL272807	MSP0512	7,51	Lisbon
HCY5JCCXY_SL269532	MSP0345	7,48	New York City
HCY5HCCXY_SL270928	MSP1089	7,45	London
HMGN5CCXY_SL344634	MSP1795	7,45	Ilorin
HCV72CCXY_SL269758	MSP1305	7,41	New York City
HKC32ALXX_SL254726	MSP0910	7,35	Tokyo
H75CGCCXY_SL263689	MSP0860	7,29	New York City
HCV72CCXY_SL269783	MSP0662	7,25	New York City
H7HJMCCXY_SL272740	MSP1918	7,25	Ilorin
HCY5HCCXY_SL271097	MSP1393	7,23	London
HCV72CCXY_SL269724	MSP0972	7,22	New York City
H7HJMCCXY_SL272791	MSP0315	7,22	Ilorin
HMCMJCCXY_SL335940	MSP0178	7,19	Lisbon
HCY5HCCXY_SL271153	MSP1907	7,18	London
H2NYMCCXY_SL254831	MSP0164	7,17	Offa
H2NYMCCXY_SL254839	MSP0761	7,17	Rio De Janeiro
H7HJMCCXY_SL272826	MSP0078	7,16	Lisbon
HKC32ALXX_SL254765	MSP0363	7,15	Ilorin
H7HJMCCXY_SL272794	MSP0504	7,13	Ilorin
HCV72CCXY_SL269719	MSP0241	7,08	New York City
H7HJMCCXY_SL272751	MSP1548	7,03	Ilorin

Table S8

HKC32ALXX_SL254692	MSP0058	7,01	Ilorin
HCY5JCCXY_SL269534	MSP0044	7,01	New York City
H7HJMCCXY_SL272804	MSP0559	6,97	Lisbon
H7HJMCCXY_SL272776	MSP0061	6,95	Ilorin
HCY5HCCXY_SL270953	MSP1372	6,95	London
H2NYMCCXY_SL254849	MSP0428	6,94	Rio De Janeiro
HCV72CCXY_SL269717	MSP0315	6,88	New York City
H2NYMCCXY_SL254772	MSP1657	6,88	Offa
H7HJMCCXY_SL272793	MSP0058	6,87	Ilorin
H2NYMCCXY_SL254859	MSP1685	6,86	Rio De Janeiro
H7HJMCCXY_SL272754	MSP1654	6,83	Ilorin
HKC32ALXX_SL254764	MSP0150	6,83	Ilorin
H2NYMCCXY_SL254795	MSP0058	6,77	Offa
HNHKFCCXY_SL345945	MSP1612	6,67	Hong Kong
HCV72CCXY_SL269744	MSP0599	6,67	New York City
HCCGHCCXY_SL270206	MSP0436	6,64	London
H7HJMCCXY_SL272783	MSP0058	6,63	Ilorin
H2NYMCCXY_SL254861	MSP0024	6,63	Rio De Janeiro
HCY5JCCXY_SL269559	MSP1756	6,62	New York City
H3KHWCCXY_SL263364	MSP0953	6,59	Porto
HCVMTCCXY_SL269593	MSP0536	6,51	New York City
HCY5HCCXY_SL270929	MSP0342	6,51	London
H3KHWCCXY_SL263311	MSP1322	6,50	Porto
HCVMTCCXY_SL269548	MSP0085	6,49	New York City
HCVMTCCXY_SL269532	MSP0345	6,48	New York City
H7HJMCCXY_SL272768	MSP0315	6,43	Ilorin
HCV72CCXY_SL269747	MSP1620	6,42	New York City
HMGMHCCXY_SL343449	MSP1081	6,42	Kyiv
H7HJMCCXY_SL272748	MSP0315	6,41	Ilorin
H75CGCCXY_SL263713	MSP0446	6,34	New York City

Table S8

HCY5HCCXY_SL270996	MSP0750	6,34	London
HKC32ALXX_SL254739	MSP0648	6,32	Tokyo
H75CGCCXY_SL263665	MSP1376	6,32	New York City
HKC32ALXX_SL254747	MSP1619	6,32	Bogota
HCY5JCCXY_SL269575	MSP0452	6,31	New York City
H75CGCCXY_SL263709	MSP1251	6,29	Auckland
HCCGHCCXY_SL270510	MSP0750	6,27	London
HMCMJCCXY_SL335949	MSP0962	6,27	Lisbon
HCVMTCCXY_SL269598	MSP0515	6,27	New York City
H75CGCCXY_SL263663	MSP0369	6,26	Hamilton
HCY5HCCXY_SL270977	MSP0515	6,26	London
HKC32ALXX_SL254757	MSP0142	6,25	Santiago
HCV72CCXY_SL269723	MSP1316	6,22	New York City
HCCGHCCXY_SL270315	MSP0750	6,18	London
HMCMJCCXY_SL335851	MSP0458	6,16	New York City
H2NYMCCXY_SL254798	MSP1612	6,16	New York City
H7HJMCCXY_SL272827	MSP1850	6,14	Lisbon
HCV72CCXY_SL269789	MSP1063	6,13	New York City
HCY5JCCXY_SL269598	MSP0515	6,12	New York City
HCY5HCCXY_SL270911	MSP0345	6,06	London
HCY5HCCXY_SL270997	MSP0272	6,06	London
HKC32ALXX_SL254766	MSP0315	6,06	Berlin
HCY5HCCXY_SL270988	MSP0078	6,04	London
H2NYMCCXY_SL254808	MSP0554	6,04	New York City
H75CGCCXY_SL263696	MSP0474	6,01	Santiago
HCY5JCCXY_SL269538	MSP0197	5,99	New York City
H2NYMCCXY_SL254830	MSP1298	5,95	Offa
H2NYMCCXY_SL254855	MSP1918	5,95	Offa
HCY5HCCXY_SL270942	MSP0878	5,90	London
HCY5JCCXY_SL269588	MSP0704	5,87	New York City

Table S8

H2NYMCCXY_SL254776	MSP0915	5,81	Rio De Janeiro
HMCMJCCXY_SL335937	MSP1576	5,79	Lisbon
H75CGCCXY_SL263690	MSP1747	5,79	New York City
HCY5HCCXY_SL270973	MSP0260	5,77	London
HCY5HCCXY_SL270962	MSP1884	5,77	London
HKC32ALXX_SL254719	MSP1907	5,73	Berlin
HCCGHCCXY_SL270257	MSP1260	5,73	London
HCVMTCCXY_SL269609	MSP0078	5,73	New York City
H7HJMCCXY_SL272775	MSP1918	5,71	Ilorin
HCV72CCXY_SL269753	MSP0878	5,70	New York City
HCV72CCXY_SL269728	MSP0900	5,69	New York City
H2NYMCCXY_SL254783	MSP0432	5,66	Offa
H7HJMCCXY_SL272801	MSP0381	5,64	Ilorin
HCY5HCCXY_SL270943	MSP0758	5,63	London
H7HJMCCXY_SL272800	MSP1080	5,62	Ilorin
HKC32ALXX_SL254680	MSP0554	5,55	Ilorin
HKC32ALXX_SL254696	MSP1907	5,54	Berlin
HCV72CCXY_SL269762	MSP0779	5,53	New York City
H7HJMCCXY_SL272796	MSP0987	5,51	Ilorin
H7HJMCCXY_SL272769	MSP0524	5,51	Ilorin
HCVMTCCXY_SL269588	MSP0704	5,50	New York City
HMCMJCCXY_SL335941	MSP1082	5,50	Lisbon
HCV72CCXY_SL269730	MSP1598	5,45	New York City
H2NYMCCXY_SL254854	MSP0058	5,44	Offa
HCY5HCCXY_SL270956	MSP1260	5,39	London
H7HJMCCXY_SL272813	MSP0382	5,39	Lisbon
H2NYMCCXY_SL254842	MSP1361	5,29	Offa
HCY5HCCXY_SL270981	MSP0540	5,23	London
HCY5JCCXY_SL269563	MSP1169	5,19	New York City
H7HJMCCXY_SL272759	MSP0029	5,19	Ilorin

Table S8

HCY5HCCXY_SL271123	MSP1260	5,17	London
H75CGCCXY_SL263652	MSP0554	5,13	Hamilton
HCVMTCCXY_SL269546	MSP0260	5,11	New York City
HCY5HCCXY_SL270915	MSP0799	5,08	London
H7HJMCCXY_SL272741	MSP1918	5,05	Ilorin
H2NYMCCXY_SL254858	MSP0428	5,05	New York City
H2NYMCCXY_SL254814	MSP0315	4,96	Rio De Janeiro
HCY5HCCXY_SL270967	MSP0704	4,94	London
HCY5HCCXY_SL270946	MSP1260	4,92	London
HCY5JCCXY_SL269609	MSP0078	4,84	New York City
H2NYMCCXY_SL254826	MSP1687	4,81	Rio De Janeiro
H75CGCCXY_SL263641	MSP0001	4,77	New York City
HCCGHCCXY_SL270438	MSP1612	4,75	London
HKC32ALXX_SL254723	MSP1612	4,74	Bogota
HMCMJCCXY_SL335947	MSP0554	4,73	Lisbon
H2NYMCCXY_SL254784	MSP1838	4,64	Offa
HKC32ALXX_SL254677	MSP1907	4,63	Tokyo
H75CGCCXY_SL263714	MSP0530	4,56	New York City
HKC32ALXX_SL254751	MSP1612	4,55	Tokyo
HCY5JCCXY_SL269546	MSP0260	4,53	New York City
H2NYMCCXY_SL254829	MSP0458	4,50	Offa
HCV72CCXY_SL269773	MSP0946	4,50	New York City
HMCMJCCXY_SL335946	MSP0458	4,49	Lisbon
HCY5HCCXY_SL270939	MSP0533	4,47	London
H2NYMCCXY_SL254806	MSP1295	4,27	Offa
HMCMJCCXY_SL335878	MSP1907	4,24	Doha
HKC32ALXX_SL254697	MSP1260	4,22	Santiago
H7HJMCCXY_SL272761	MSP1204	4,18	Ilorin
HKC32ALXX_SL254750	MSP1193	4,15	Tokyo
H7HJMCCXY_SL272770	MSP0315	4,15	Ilorin

Table S8

H7HJMCCXY_SL272780	MSP1771	4,10	Ilorin
HCY5HCCXY_SL270971	MSP0523	4,01	London
H2NYMCCXY_SL254793	MSP1388	3,91	Offa
H2NYMCCXY_SL254853	MSP0315	3,88	Offa
H7HJMCCXY_SL272757	MSP0274	3,84	Ilorin
H2NYMCCXY_SL254843	MSP0458	3,78	Offa
H7HJMCCXY_SL272773	MSP0278	3,77	Ilorin
HCV72CCXY_SL269748	MSP0627	3,73	New York City
H2NYMCCXY_SL254771	MSP0564	3,63	Offa
HCY5HCCXY_SL270912	MSP1907	3,57	London
H75CGCCXY_SL263716	MSP0530	3,56	Porto
H7HJMCCXY_SL272738	MSP0554	3,48	Ilorin
H2NYMCCXY_SL254796	MSP1838	3,46	New York City
H7HJMCCXY_SL272742	MSP1186	3,46	Ilorin
H7HJMCCXY_SL272832	MSP0186	3,42	Other Control
HCV72CCXY_SL269732	MSP1393	3,42	New York City
H7HJMCCXY_SL272774	MSP0254	3,41	Ilorin
H7HJMCCXY_SL272765	MSP0552	3,37	Ilorin
HMCMJCCXY_SL335842	MSP0186	3,37	New York City
H2NYMCCXY_SL254788	MSP1613	3,26	Rio De Janeiro
H2NYMCCXY_SL254838	MSP1687	3,22	Rio De Janeiro
H7HJMCCXY_SL272798	MSP0554	3,20	Ilorin
H2NYMCCXY_SL254818	MSP1918	3,17	Offa
H2NYMCCXY_SL254791	MSP0430	3,08	Rio De Janeiro
H2NYMCCXY_SL254794	MSP1080	2,96	Offa

Table S9

Supplementary Table S9. Significance of the mean abundance of ARGs carriers versus non-carriers. We carried out two different approaches for testing the mean abundance of the two groups as described in Material and Methods. The asterisk (*) shows the number of tests below $p < 0.05$.

	p-values	
**Cities**	**ttest**	**permutation**
Auckland	0,952	0,071
Berlin	0,938	0,399
Bogota	0,290	0,058
Cairngorm	0,602	0,398
Denver*	0,048	0,514
Doha**	0,030	0,001
Fairbanks	0,706	0,445
Hamilton**	0,007	0,000
Hanoi**	0,031	0,000
Hong Kong*	0,039	0,832
Ilorin**	0,010	0,014
Kuala Lumpur**	0,020	0,001
Kyiv**	0,016	0,000
Lisbon**	0,020	0,004
London**	0,020	0,000
Marseille	0,514	0,151
Minneapolis*	0,000	0,552
Naples	0,654	0,319
New York City**	0,016	0,000
Offa**	0,000	0,000
Oslo	0,064	0,572
Others*	0,090	0,002
Porto	0,560	0,236
Rio de Janeiro*	0,084	0,006
Santiago	0,978	0,394
Sao Paulo	0,990	0,275
Seoul	0,346	0,665
Singapore	0,486	0,186
Sofia	0,946	0,299
Taipei*	0,035	0,486
Tokyo	0,324	0,152
Vienna	0,524	0,277
Zurich	0,407	0,460

Supplemmentary Table S10. GenBank Accession Numbers of Putative Phage Genomes (PPGs)

These phage sequences are also available from http://www.iq.usp.br/setubal/metasubphages/ppgsequences.fasta

PPG	ACCESSION	PPG	ACCESSION	PPG	ACCESSION	PPG	ACCESSION	PPG	ACCESSION
MSP0001	OR222109	MSP0368	OR222566	MSP0747	OR222221	MSP1125	OR221877	MSP1539	OR221540
MSP0002	OR222107	MSP0369	OR222569	MSP0748	OR222222	MSP1126	OR221879	MSP1540	OR221531
MSP0003	OR222104	MSP0370	OR222562	MSP0749	OR222223	MSP1127	OR221878	MSP1541	OR221533
MSP0004	OR222108	MSP0371	OR222564	MSP0750	OR222217	MSP1128	OR221883	MSP1542	OR221534
MSP0005	OR222105	MSP0372	OR222563	MSP0751	OR222218	MSP1129	OR221872	MSP1543	OR221535
MSP0006	OR222106	MSP0373	OR222559	MSP0753	OR222219	MSP1130	OR221873	MSP1545	OR221537
MSP0007	OR222103	MSP0374	OR222560	MSP0754	OR222220	MSP1131	OR221875	MSP1546	OR221525
MSP0008	OR222102	MSP0375	OR222561	MSP0755	OR222212	MSP1133	OR221874	MSP1547	OR221527
MSP0010	OR222100	MSP0376	OR222556	MSP0756	OR222214	MSP1134	OR221876	MSP1548	OR221529
MSP0011	OR222101	MSP0377	OR222558	MSP0757	OR222215	MSP1135	OR221869	MSP1549	OR221532
MSP0012	OR222099	MSP0378	OR222557	MSP0758	OR222216	MSP1136	OR221868	MSP1551	OR221530
MSP0013	OR222098	MSP0379	OR222552	MSP0759	OR222209	MSP1137	OR221870	MSP1553	OR221523
MSP0014	OR222095	MSP0380	OR222554	MSP0760	OR222210	MSP1138	OR221871	MSP1554	OR221520
MSP0015	OR222096	MSP0381	OR222555	MSP0761	OR222211	MSP1139	OR221865	MSP1556	OR221521
MSP0016	OR222097	MSP0382	OR222551	MSP0762	OR222213	MSP1141	OR221864	MSP1557	OR221524
MSP0017	OR222092	MSP0383	OR222553	MSP0763	OR222205	MSP1142	OR221866	MSP1558	OR221526
MSP0018	OR222094	MSP0384	OR222548	MSP0764	OR222206	MSP1143	OR221867	MSP1559	OR221528
MSP0019	OR222093	MSP0385	OR222547	MSP0765	OR222207	MSP1144	OR221858	MSP1560	OR221516
MSP0020	OR222090	MSP0386	OR222549	MSP0766	OR222208	MSP1145	OR221860	MSP1561	OR221519
MSP0022	OR222091	MSP0387	OR222550	MSP0767	OR222204	MSP1147	OR221861	MSP1562	OR221517
MSP0023	OR222089	MSP0388	OR222545	MSP0768	OR222201	MSP1149	OR221863	MSP1563	OR221518
MSP0024	OR222088	MSP0389	OR222546	MSP0769	OR222202	MSP1151	OR221862	MSP1564	OR221522
MSP0025	OR222087	MSP0391	OR222542	MSP0771	OR222203	MSP1152	OR221854	MSP1565	OR221511
MSP0026	OR222085	MSP0392	OR222544	MSP0772	OR222199	MSP1153	OR221855	MSP1566	OR221514
MSP0027	OR222084	MSP0393	OR222540	MSP0774	OR222197	MSP1154	OR221857	MSP1567	OR221512
MSP0028	OR222082	MSP0396	OR222543	MSP0775	OR222200	MSP1155	OR221856	MSP1568	OR221513
MSP0029	OR222086	MSP0398	OR222541	MSP0776	OR222193	MSP1156	OR221859	MSP1569	OR221515
MSP0030	OR222083	MSP0399	OR222538	MSP0777	OR222194	MSP1157	OR221848	MSP1570	OR221510
MSP0031	OR222081	MSP0400	OR222539	MSP0779	OR222196	MSP1158	OR221850	MSP1571	OR221505
MSP0032	OR222080	MSP0401	OR222535	MSP0781	OR222198	MSP1159	OR221851	MSP1572	OR221506
MSP0033	OR222079	MSP0402	OR222534	MSP0782	OR222190	MSP1160	OR221852	MSP1573	OR221507
MSP0034	OR222078	MSP0403	OR222537	MSP0783	OR222191	MSP1161	OR221853	MSP1574	OR221508

MSP0035	OR222076	MSP0404	OR222536	MSP0784	OR222192	MSP1162	OR221845	MSP1575	OR221509
MSP0036	OR222077	MSP0405	OR222532	MSP0785	OR222195	MSP1163	OR221847	MSP1576	OR221500
MSP0037	OR222074	MSP0406	OR222533	MSP0786	OR222187	MSP1164	OR221846	MSP1577	OR221501
MSP0038	OR222073	MSP0407	OR222530	MSP0787	OR222189	MSP1166	OR221849	MSP1578	OR221502
MSP0040	OR222075	MSP0408	OR222531	MSP0788	OR222188	MSP1167	OR221840	MSP1579	OR221503
MSP0041	OR222072	MSP0409	OR222527	MSP0789	OR222184	MSP1169	OR221841	MSP1580	OR221504
MSP0042	OR222070	MSP0410	OR222525	MSP0790	OR222182	MSP1170	OR221842	MSP1581	OR221495
MSP0043	OR222071	MSP0411	OR222529	MSP0791	OR222185	MSP1171	OR221843	MSP1582	OR221496
MSP0044	OR222069	MSP0412	OR222524	MSP0792	OR222186	MSP1172	OR221844	MSP1583	OR221497
MSP0045	OR222066	MSP0414	OR222528	MSP0793	OR222179	MSP1173	OR221835	MSP1584	OR221498
MSP0046	OR222068	MSP0415	OR222526	MSP0794	OR222180	MSP1174	OR221836	MSP1585	OR221499
MSP0047	OR222065	MSP0417	OR222521	MSP0795	OR222181	MSP1175	OR221837	MSP1586	OR221489
MSP0048	OR222064	MSP0418	OR222523	MSP0796	OR222183	MSP1176	OR221838	MSP1587	OR221490
MSP0049	OR222067	MSP0419	OR222519	MSP0797	OR222174	MSP1178	OR221839	MSP1588	OR221491
MSP0050	OR222063	MSP0420	OR222520	MSP0798	OR222176	MSP1179	OR221831	MSP1589	OR221492
MSP0051	OR222060	MSP0421	OR222522	MSP0799	OR222177	MSP1180	OR221832	MSP1590	OR221493
MSP0052	OR222062	MSP0423	OR222516	MSP0800	OR222178	MSP1181	OR221833	MSP1591	OR221494
MSP0054	OR222061	MSP0428	OR222518	MSP0801	OR222172	MSP1182	OR221834	MSP1592	OR221484
MSP0055	OR222058	MSP0429	OR222517	MSP0803	OR222171	MSP1183	OR221826	MSP1593	OR221485
MSP0056	OR222059	MSP0430	OR222510	MSP0804	OR222173	MSP1184	OR221827	MSP1594	OR221487
MSP0057	OR222056	MSP0431	OR222512	MSP0805	OR222175	MSP1185	OR221828	MSP1595	OR221486
MSP0058	OR222057	MSP0432	OR222515	MSP0806	OR222166	MSP1186	OR221829	MSP1597	OR221488
MSP0059	OR222055	MSP0433	OR222513	MSP0807	OR222168	MSP1187	OR221830	MSP1598	OR221479
MSP0060	OR222053	MSP0434	OR222511	MSP0808	OR222169	MSP1188	OR221820	MSP1599	OR221480
MSP0061	OR222054	MSP0435	OR222514	MSP0809	OR222170	MSP1189	OR221821	MSP1600	OR221482
MSP0062	OR222052	MSP0436	OR222906	MSP0810	OR222163	MSP1190	OR221823	MSP1601	OR221481
MSP0063	OR222050	MSP0437	OR222907	MSP0812	OR222165	MSP1192	OR221824	MSP1602	OR221483
MSP0064	OR222051	MSP0438	OR222908	MSP0813	OR222164	MSP1193	OR221825	MSP1603	OR221474
MSP0065	OR222048	MSP0440	OR222909	MSP0814	OR222167	MSP1194	OR221816	MSP1604	OR221477
MSP0066	OR222049	MSP0441	OR222904	MSP0815	OR222159	MSP1195	OR221817	MSP1605	OR221475
MSP0067	OR222046	MSP0442	OR222905	MSP0817	OR222158	MSP1197	OR221818	MSP1606	OR221476
MSP0068	OR222047	MSP0443	OR222899	MSP0818	OR222160	MSP1198	OR221819	MSP1607	OR221478
MSP0069	OR222045	MSP0444	OR222900	MSP0819	OR222161	MSP1199	OR221822	MSP1608	OR221468

MSP0070	OR222043	MSP0445	OR222902	MSP0820	OR222162	MSP1202	OR221810	MSP1609	OR221471
MSP0071	OR222044	MSP0446	OR222903	MSP0821	OR222155	MSP1204	OR221812	MSP1610	OR221469
MSP0072	OR222042	MSP0448	OR222897	MSP0823	OR222153	MSP1206	OR221813	MSP1611	OR221470
MSP0073	OR222041	MSP0449	OR222898	MSP0824	OR222157	MSP1207	OR221814	MSP1612	OR221472
MSP0074	OR222040	MSP0450	OR222901	MSP0825	OR222156	MSP1208	OR221815	MSP1613	OR221473
MSP0075	OR222038	MSP0451	OR222893	MSP0827	OR222150	MSP1209	OR221805	MSP1614	OR221462
MSP0076	OR222039	MSP0452	OR222894	MSP0828	OR222151	MSP1211	OR221808	MSP1615	OR221465
MSP0077	OR222036	MSP0453	OR222895	MSP0829	OR222154	MSP1213	OR221807	MSP1616	OR221463
MSP0078	OR222037	MSP0454	OR222896	MSP0831	OR222152	MSP1214	OR221811	MSP1617	OR221464
MSP0079	OR222034	MSP0455	OR222889	MSP0832	OR222145	MSP1215	OR221809	MSP1618	OR221466
MSP0081	OR222033	MSP0456	OR222891	MSP0833	OR222146	MSP1216	OR221802	MSP1619	OR221467
MSP0082	OR222032	MSP0457	OR222892	MSP0834	OR222147	MSP1217	OR221803	MSP1620	OR221455
MSP0083	OR222031	MSP0458	OR222887	MSP0835	OR222148	MSP1219	OR221804	MSP1621	OR221458
MSP0084	OR222035	MSP0459	OR222890	MSP0836	OR222149	MSP1220	OR221806	MSP1622	OR221457
MSP0085	OR222029	MSP0460	OR222888	MSP0837	OR222141	MSP1221	OR221797	MSP1623	OR221459
MSP0086	OR222030	MSP0461	OR222884	MSP0838	OR222143	MSP1222	OR221798	MSP1624	OR221460
MSP0088	OR222027	MSP0463	OR222885	MSP0839	OR222142	MSP1223	OR221799	MSP1625	OR221461
MSP0089	OR222028	MSP0465	OR222886	MSP0840	OR222144	MSP1225	OR221801	MSP1626	OR221451
MSP0090	OR222025	MSP0466	OR222879	MSP0841	OR222137	MSP1226	OR221800	MSP1627	OR221453
MSP0091	OR222026	MSP0467	OR222881	MSP0842	OR222138	MSP1227	OR221793	MSP1629	OR221454
MSP0094	OR222023	MSP0468	OR222883	MSP0843	OR222140	MSP1228	OR221794	MSP1630	OR221456
MSP0095	OR222024	MSP0469	OR222882	MSP0844	OR222139	MSP1229	OR221796	MSP1631	OR221446
MSP0097	OR222022	MSP0470	OR222878	MSP0845	OR222133	MSP1230	OR221795	MSP1632	OR221447
MSP0101	OR222021	MSP0471	OR222877	MSP0846	OR222134	MSP1232	OR221787	MSP1633	OR221448
MSP0102	OR222020	MSP0472	OR222880	MSP0847	OR222135	MSP1233	OR221788	MSP1634	OR221449
MSP0103	OR222019	MSP0473	OR222874	MSP0848	OR222136	MSP1234	OR221790	MSP1635	OR221450
MSP0104	OR222018	MSP0474	OR222875	MSP0850	OR222128	MSP1235	OR221789	MSP1636	OR221452
MSP0105	OR222015	MSP0475	OR222876	MSP0851	OR222129	MSP1236	OR221791	MSP1637	OR221441
MSP0106	OR222017	MSP0476	OR222873	MSP0852	OR222130	MSP1237	OR221792	MSP1638	OR221442
MSP0107	OR222014	MSP0477	OR222871	MSP0853	OR222132	MSP1238	OR221783	MSP1639	OR221444
MSP0108	OR222016	MSP0478	OR222872	MSP0854	OR222131	MSP1240	OR221786	MSP1642	OR221443
MSP0109	OR222012	MSP0479	OR222869	MSP0855	OR222125	MSP1241	OR221784	MSP1644	OR221445
MSP0110	OR222013	MSP0481	OR222867	MSP0856	OR222126	MSP1242	OR221785	MSP1645	OR221436

MSP0111	OR222011	MSP0483	OR222868	MSP0858	OR222127	MSP1243	OR221780	MSP1646	OR221439
MSP0112	OR222010	MSP0484	OR222870	MSP0859	OR222121	MSP1244	OR221779	MSP1647	OR221437
MSP0113	OR222009	MSP0485	OR222864	MSP0860	OR222122	MSP1245	OR221782	MSP1649	OR221438
MSP0114	OR222008	MSP0486	OR222865	MSP0861	OR222124	MSP1246	OR221781	MSP1650	OR221440
MSP0115	OR222006	MSP0487	OR222866	MSP0862	OR222123	MSP1247	OR221777	MSP1651	OR221431
MSP0116	OR222007	MSP0488	OR222860	MSP0863	OR222118	MSP1248	OR221775	MSP1652	OR221430
MSP0119	OR222004	MSP0489	OR222861	MSP0865	OR222119	MSP1249	OR221774	MSP1653	OR221432
MSP0120	OR222005	MSP0491	OR222862	MSP0866	OR222116	MSP1250	OR221776	MSP1654	OR221433
MSP0121	OR222002	MSP0492	OR222863	MSP0867	OR222120	MSP1251	OR221778	MSP1657	OR221434
MSP0122	OR222003	MSP0493	OR222857	MSP0868	OR222113	MSP1252	OR221769	MSP1659	OR221435
MSP0123	OR221999	MSP0494	OR222858	MSP0869	OR222112	MSP1253	OR221771	MSP1660	OR221425
MSP0124	OR222000	MSP0495	OR222859	MSP0870	OR222114	MSP1254	OR221772	MSP1661	OR221426
MSP0125	OR221998	MSP0496	OR222854	MSP0871	OR222115	MSP1255	OR221770	MSP1662	OR221427
MSP0127	OR222001	MSP0497	OR222855	MSP0872	OR222117	MSP1256	OR221773	MSP1664	OR221428
MSP0128	OR221997	MSP0498	OR222856	MSP0873	OR222110	MSP1257	OR221763	MSP1666	OR221429
MSP0129	OR221996	MSP0499	OR222851	MSP0874	OR222111	MSP1259	OR221766	MSP1667	OR221421
MSP0130	OR221995	MSP0500	OR222852	MSP0875	OR222509	MSP1260	OR221767	MSP1668	OR221423
MSP0131	OR221993	MSP0501	OR222853	MSP0876	OR222505	MSP1261	OR221765	MSP1669	OR221420
MSP0132	OR221994	MSP0502	OR222847	MSP0877	OR222504	MSP1262	OR221768	MSP1670	OR221422
MSP0133	OR221991	MSP0503	OR222849	MSP0878	OR222508	MSP1263	OR221759	MSP1671	OR221424
MSP0134	OR221992	MSP0504	OR222850	MSP0880	OR222507	MSP1264	OR221762	MSP1672	OR221417
MSP0135	OR221989	MSP0505	OR222844	MSP0881	OR222506	MSP1266	OR221760	MSP1673	OR221415
MSP0136	OR221990	MSP0506	OR222845	MSP0882	OR222500	MSP1268	OR221761	MSP1674	OR221416
MSP0137	OR221987	MSP0507	OR222846	MSP0883	OR222502	MSP1270	OR221764	MSP1676	OR221418
MSP0139	OR221988	MSP0509	OR222848	MSP0884	OR222501	MSP1272	OR221754	MSP1677	OR221419
MSP0140	OR221986	MSP0510	OR222841	MSP0885	OR222503	MSP1273	OR221756	MSP1678	OR221409
MSP0141	OR221985	MSP0511	OR222843	MSP0886	OR222497	MSP1275	OR221755	MSP1680	OR221411
MSP0142	OR221983	MSP0512	OR222842	MSP0887	OR222496	MSP1276	OR221757	MSP1681	OR221410
MSP0143	OR221984	MSP0514	OR222838	MSP0888	OR222498	MSP1277	OR221758	MSP1682	OR221412
MSP0144	OR221982	MSP0515	OR222839	MSP0889	OR222499	MSP1278	OR221748	MSP1683	OR221413
MSP0145	OR221979	MSP0516	OR222840	MSP0890	OR222491	MSP1279	OR221750	MSP1684	OR221414
MSP0146	OR221980	MSP0517	OR222836	MSP0891	OR222493	MSP1280	OR221751	MSP1685	OR221405
MSP0147	OR221981	MSP0518	OR222837	MSP0892	OR222494	MSP1281	OR221752	MSP1686	OR221404

MSP0148	OR221976	MSP0519	OR222835	MSP0893	OR222495	MSP1283	OR221753	MSP1687	OR221406
MSP0149	OR221978	MSP0520	OR222834	MSP0894	OR222487	MSP1285	OR221745	MSP1689	OR221407
MSP0150	OR221975	MSP0521	OR222831	MSP0896	OR222489	MSP1286	OR221744	MSP1691	OR221408
MSP0151	OR221977	MSP0522	OR222832	MSP0898	OR222490	MSP1288	OR221746	MSP1692	OR221398
MSP0152	OR221973	MSP0523	OR222833	MSP0899	OR222492	MSP1289	OR221747	MSP1693	OR221399
MSP0153	OR221974	MSP0524	OR222827	MSP0900	OR222484	MSP1290	OR221749	MSP1694	OR221401
MSP0154	OR221971	MSP0526	OR222828	MSP0901	OR222486	MSP1292	OR221737	MSP1695	OR221400
MSP0155	OR221972	MSP0527	OR222830	MSP0902	OR222488	MSP1293	OR221739	MSP1696	OR221402
MSP0156	OR221968	MSP0528	OR222829	MSP0903	OR222485	MSP1294	OR221741	MSP1697	OR221403
MSP0157	OR221969	MSP0529	OR222822	MSP0904	OR222480	MSP1295	OR221742	MSP1698	OR221393
MSP0158	OR221966	MSP0530	OR222825	MSP0906	OR222482	MSP1296	OR221743	MSP1699	OR221394
MSP0159	OR221967	MSP0531	OR222826	MSP0907	OR222481	MSP1297	OR221733	MSP1700	OR221395
MSP0160	OR221970	MSP0533	OR222824	MSP0908	OR222483	MSP1298	OR221734	MSP1701	OR221397
MSP0161	OR221963	MSP0534	OR222819	MSP0909	OR222475	MSP1299	OR221735	MSP1702	OR221396
MSP0162	OR221965	MSP0535	OR222820	MSP0910	OR222478	MSP1300	OR221740	MSP1703	OR221386
MSP0163	OR221962	MSP0536	OR222821	MSP0911	OR222477	MSP1301	OR221738	MSP1704	OR221388
MSP0164	OR221964	MSP0537	OR222823	MSP0912	OR222479	MSP1302	OR221729	MSP1705	OR221389
MSP0165	OR221958	MSP0538	OR222818	MSP0913	OR222472	MSP1303	OR221730	MSP1706	OR221390
MSP0166	OR221961	MSP0539	OR222815	MSP0914	OR222474	MSP1304	OR221731	MSP1708	OR221392
MSP0167	OR221959	MSP0540	OR222816	MSP0915	OR222476	MSP1305	OR221732	MSP1709	OR221391
MSP0168	OR221960	MSP0541	OR222811	MSP0916	OR222467	MSP1306	OR221736	MSP1710	OR221381
MSP0169	OR221957	MSP0542	OR222814	MSP0917	OR222469	MSP1307	OR221723	MSP1711	OR221383
MSP0170	OR221956	MSP0543	OR222817	MSP0918	OR222471	MSP1308	OR221725	MSP1712	OR221384
MSP0171	OR221954	MSP0544	OR222812	MSP0919	OR222470	MSP1309	OR221726	MSP1713	OR221385
MSP0172	OR221953	MSP0545	OR222809	MSP0920	OR222473	MSP1310	OR221727	MSP1714	OR221387
MSP0173	OR221952	MSP0546	OR222813	MSP0921	OR222464	MSP1312	OR221728	MSP1715	OR221377
MSP0174	OR221955	MSP0547	OR222810	MSP0922	OR222466	MSP1313	OR221719	MSP1716	OR221378
MSP0176	OR221948	MSP0548	OR222806	MSP0923	OR222465	MSP1314	OR221720	MSP1717	OR221379
MSP0177	OR221951	MSP0549	OR222808	MSP0924	OR222468	MSP1315	OR221721	MSP1718	OR221380
MSP0178	OR221950	MSP0550	OR222807	MSP0925	OR222459	MSP1316	OR221722	MSP1719	OR221382
MSP0179	OR221949	MSP0552	OR222802	MSP0926	OR222461	MSP1318	OR221724	MSP1720	OR221371
MSP0180	OR221946	MSP0553	OR222803	MSP0927	OR222462	MSP1319	OR221713	MSP1721	OR221373
MSP0181	OR221947	MSP0554	OR222805	MSP0928	OR222463	MSP1320	OR221716	MSP1722	OR221372

MSP0182	OR221943	MSP0555	OR222804	MSP0929	OR222456	MSP1322	OR221714	MSP1723	OR221376
MSP0183	OR221945	MSP0556	OR222799	MSP0930	OR222457	MSP1323	OR221717	MSP1724	OR221374
MSP0184	OR221942	MSP0557	OR222800	MSP0931	OR222458	MSP1324	OR221718	MSP1725	OR221375
MSP0185	OR221944	MSP0558	OR222801	MSP0934	OR222460	MSP1326	OR221708	MSP1726	OR221367
MSP0186	OR221941	MSP0559	OR222795	MSP0935	OR222451	MSP1327	OR221710	MSP1727	OR221368
MSP0187	OR221940	MSP0560	OR222796	MSP0936	OR222453	MSP1328	OR221709	MSP1728	OR221366
MSP0188	OR221938	MSP0561	OR222797	MSP0938	OR222454	MSP1329	OR221711	MSP1729	OR221370
MSP0189	OR221937	MSP0562	OR222798	MSP0939	OR222455	MSP1330	OR221712	MSP1730	OR221369
MSP0190	OR221939	MSP0563	OR222792	MSP0940	OR222448	MSP1331	OR221715	MSP1731	OR221360
MSP0192	OR221934	MSP0564	OR222793	MSP0941	OR222450	MSP1332	OR221703	MSP1732	OR221362
MSP0193	OR221936	MSP0565	OR222794	MSP0942	OR222449	MSP1333	OR221704	MSP1733	OR221361
MSP0195	OR221933	MSP0566	OR222789	MSP0944	OR222452	MSP1334	OR221705	MSP1734	OR221363
MSP0196	OR221935	MSP0567	OR222790	MSP0945	OR222443	MSP1335	OR221706	MSP1735	OR221364
MSP0197	OR221931	MSP0568	OR222791	MSP0946	OR222445	MSP1337	OR221707	MSP1736	OR221365
MSP0198	OR221932	MSP0569	OR222785	MSP0947	OR222444	MSP1338	OR221698	MSP1737	OR221354
MSP0199	OR221928	MSP0570	OR222786	MSP0948	OR222447	MSP1339	OR221699	MSP1738	OR221355
MSP0200	OR221929	MSP0571	OR222787	MSP0949	OR222446	MSP1340	OR221700	MSP1740	OR221356
MSP0201	OR221930	MSP0572	OR222788	MSP0950	OR222441	MSP1341	OR221701	MSP1742	OR221358
MSP0202	OR221927	MSP0573	OR222782	MSP0951	OR222439	MSP1342	OR221702	MSP1743	OR221357
MSP0203	OR221926	MSP0574	OR222783	MSP0952	OR222442	MSP1344	OR221695	MSP1744	OR221359
MSP0204	OR221923	MSP0575	OR222784	MSP0953	OR222440	MSP1345	OR221693	MSP1746	OR221350
MSP0205	OR221925	MSP0576	OR222778	MSP0954	OR222437	MSP1346	OR221694	MSP1747	OR221349
MSP0206	OR221921	MSP0577	OR222779	MSP0955	OR222435	MSP1347	OR221696	MSP1748	OR221351
MSP0207	OR221922	MSP0578	OR222780	MSP0956	OR222436	MSP1348	OR221697	MSP1749	OR221353
MSP0208	OR221924	MSP0579	OR222781	MSP0957	OR222438	MSP1349	OR221688	MSP1750	OR221352
MSP0209	OR221919	MSP0580	OR222775	MSP0958	OR222430	MSP1350	OR221689	MSP1753	OR221344
MSP0210	OR221920	MSP0581	OR222776	MSP0959	OR222432	MSP1351	OR221690	MSP1754	OR221345
MSP0211	OR221916	MSP0582	OR222777	MSP0960	OR222431	MSP1352	OR221691	MSP1755	OR221347
MSP0212	OR221918	MSP0583	OR222773	MSP0961	OR222433	MSP1353	OR221692	MSP1756	OR221346
MSP0213	OR221915	MSP0584	OR222772	MSP0962	OR222434	MSP1354	OR221684	MSP1757	OR221348
MSP0214	OR221917	MSP0586	OR222774	MSP0963	OR222425	MSP1355	OR221683	MSP1760	OR221337
MSP0215	OR221911	MSP0587	OR222768	MSP0965	OR222426	MSP1358	OR221686	MSP1761	OR221339
MSP0216	OR221910	MSP0588	OR222769	MSP0967	OR222428	MSP1359	OR221687	MSP1762	OR221340

MSP0217	OR221913	MSP0589	OR222770	MSP0968	OR222427	MSP1360	OR221679	MSP1763	OR221342
MSP0218	OR221912	MSP0590	OR222771	MSP0969	OR222429	MSP1361	OR221681	MSP1766	OR221341
MSP0219	OR221914	MSP0591	OR222764	MSP0970	OR222421	MSP1362	OR221680	MSP1767	OR221343
MSP0220	OR222709	MSP0592	OR222765	MSP0971	OR222422	MSP1363	OR221682	MSP1768	OR221333
MSP0221	OR222708	MSP0593	OR222766	MSP0972	OR222423	MSP1364	OR221685	MSP1769	OR221332
MSP0222	OR222706	MSP0594	OR222767	MSP0973	OR222424	MSP1365	OR221677	MSP1770	OR221335
MSP0223	OR222707	MSP0595	OR222761	MSP0974	OR222417	MSP1366	OR221674	MSP1771	OR221334
MSP0224	OR222705	MSP0596	OR222762	MSP0975	OR222418	MSP1367	OR221675	MSP1772	OR221336
MSP0225	OR222703	MSP0598	OR222763	MSP0976	OR222419	MSP1368	OR221676	MSP1773	OR221338
MSP0226	OR222704	MSP0599	OR222756	MSP0977	OR222420	MSP1370	OR221678	MSP1774	OR221326
MSP0227	OR222701	MSP0600	OR222759	MSP0978	OR222413	MSP1371	OR221667	MSP1776	OR221327
MSP0228	OR222702	MSP0601	OR222758	MSP0980	OR222412	MSP1372	OR221671	MSP1777	OR221328
MSP0229	OR222699	MSP0602	OR222760	MSP0981	OR222414	MSP1373	OR221669	MSP1778	OR221329
MSP0230	OR222700	MSP0603	OR222754	MSP0982	OR222415	MSP1374	OR221670	MSP1779	OR221330
MSP0232	OR222697	MSP0604	OR222753	MSP0983	OR222416	MSP1375	OR221672	MSP1780	OR221331
MSP0233	OR222698	MSP0605	OR222755	MSP0985	OR222408	MSP1376	OR221673	MSP1781	OR221323
MSP0234	OR222694	MSP0606	OR222757	MSP0986	OR222409	MSP1377	OR221662	MSP1782	OR221322
MSP0235	OR222695	MSP0608	OR222748	MSP0987	OR222410	MSP1378	OR221664	MSP1783	OR221321
MSP0236	OR222696	MSP0609	OR222750	MSP0988	OR222411	MSP1379	OR221666	MSP1784	OR221324
MSP0237	OR222692	MSP0610	OR222751	MSP0989	OR222403	MSP1380	OR221665	MSP1785	OR221325
MSP0238	OR222693	MSP0611	OR222752	MSP0990	OR222405	MSP1382	OR221668	MSP1786	OR221316
MSP0239	OR222689	MSP0612	OR222745	MSP0991	OR222406	MSP1383	OR221658	MSP1787	OR221317
MSP0240	OR222691	MSP0613	OR222746	MSP0992	OR222407	MSP1384	OR221660	MSP1788	OR221318
MSP0241	OR222688	MSP0614	OR222749	MSP0993	OR222401	MSP1387	OR221659	MSP1789	OR221319
MSP0242	OR222687	MSP0615	OR222747	MSP0994	OR222400	MSP1388	OR221661	MSP1790	OR221320
MSP0243	OR222690	MSP0616	OR222744	MSP0995	OR222404	MSP1389	OR221663	MSP1791	OR221311
MSP0244	OR222684	MSP0617	OR222743	MSP0997	OR222402	MSP1390	OR221653	MSP1792	OR221312
MSP0245	OR222686	MSP0618	OR222742	MSP0998	OR222396	MSP1391	OR221654	MSP1793	OR221313
MSP0246	OR222685	MSP0619	OR222736	MSP0999	OR222398	MSP1392	OR221655	MSP1795	OR221314
MSP0247	OR222682	MSP0621	OR222738	MSP1000	OR222397	MSP1393	OR221656	MSP1796	OR221315
MSP0248	OR222683	MSP0623	OR222741	MSP1001	OR222399	MSP1394	OR221657	MSP1797	OR221304
MSP0249	OR222679	MSP0624	OR222740	MSP1003	OR222390	MSP1395	OR221645	MSP1798	OR221306
MSP0250	OR222681	MSP0625	OR222735	MSP1004	OR222392	MSP1397	OR221648	MSP1799	OR221308

MSP0252	OR222680	MSP0626	OR222737	MSP1006	OR222393	MSP1399	OR221649	MSP1800	OR221307
MSP0253	OR222676	MSP0627	OR222739	MSP1007	OR222394	MSP1400	OR221650	MSP1801	OR221310
MSP0254	OR222677	MSP0628	OR222732	MSP1008	OR222395	MSP1401	OR221652	MSP1802	OR221309
MSP0255	OR222678	MSP0629	OR222730	MSP1009	OR222388	MSP1402	OR221651	MSP1803	OR221299
MSP0256	OR222674	MSP0630	OR222733	MSP1010	OR222387	MSP1403	OR221643	MSP1804	OR221300
MSP0257	OR222675	MSP0632	OR222727	MSP1011	OR222389	MSP1404	OR221642	MSP1805	OR221301
MSP0258	OR222671	MSP0633	OR222729	MSP1012	OR222391	MSP1405	OR221644	MSP1806	OR221302
MSP0259	OR222672	MSP0634	OR222734	MSP1013	OR222382	MSP1406	OR221646	MSP1807	OR221303
MSP0260	OR222673	MSP0635	OR222731	MSP1014	OR222384	MSP1408	OR221647	MSP1808	OR221305
MSP0261	OR222669	MSP0636	OR222724	MSP1015	OR222386	MSP1409	OR221637	MSP1809	OR221294
MSP0262	OR222670	MSP0637	OR222728	MSP1016	OR222385	MSP1411	OR221638	MSP1810	OR221295
MSP0263	OR222665	MSP0638	OR222726	MSP1017	OR222377	MSP1412	OR221639	MSP1811	OR221296
MSP0264	OR222668	MSP0639	OR222725	MSP1018	OR222380	MSP1413	OR221640	MSP1812	OR221298
MSP0265	OR222666	MSP0640	OR222720	MSP1019	OR222381	MSP1414	OR221641	MSP1814	OR221297
MSP0266	OR222667	MSP0642	OR222721	MSP1020	OR222383	MSP1415	OR221635	MSP1815	OR221290
MSP0267	OR222661	MSP0643	OR222723	MSP1021	OR222375	MSP1416	OR221633	MSP1816	OR221289
MSP0268	OR222663	MSP0644	OR222722	MSP1022	OR222376	MSP1417	OR221634	MSP1819	OR221291
MSP0269	OR222664	MSP0645	OR222716	MSP1023	OR222379	MSP1418	OR221636	MSP1820	OR221292
MSP0270	OR222660	MSP0646	OR222717	MSP1024	OR222378	MSP1419	OR221627	MSP1821	OR221293
MSP0271	OR222662	MSP0647	OR222718	MSP1025	OR222371	MSP1420	OR221629	MSP1822	OR221283
MSP0272	OR222657	MSP0648	OR222719	MSP1026	OR222373	MSP1421	OR221628	MSP1823	OR221284
MSP0273	OR222658	MSP0650	OR222713	MSP1027	OR222374	MSP1423	OR221630	MSP1824	OR221285
MSP0274	OR222659	MSP0651	OR222712	MSP1028	OR222372	MSP1424	OR221632	MSP1825	OR221286
MSP0275	OR222655	MSP0652	OR222714	MSP1029	OR222367	MSP1425	OR221631	MSP1826	OR221287
MSP0276	OR222656	MSP0653	OR222715	MSP1031	OR222366	MSP1427	OR221621	MSP1827	OR221288
MSP0277	OR222652	MSP0654	OR222710	MSP1032	OR222368	MSP1428	OR221623	MSP1828	OR221276
MSP0278	OR222653	MSP0655	OR222711	MSP1033	OR222369	MSP1429	OR221624	MSP1829	OR221279
MSP0279	OR222654	MSP0656	OR222309	MSP1034	OR222370	MSP1432	OR221625	MSP1830	OR221281
MSP0280	OR222648	MSP0657	OR222306	MSP1035	OR222361	MSP1433	OR221626	MSP1831	OR221280
MSP0281	OR222649	MSP0658	OR222307	MSP1036	OR222363	MSP1434	OR221615	MSP1832	OR221282
MSP0282	OR222651	MSP0659	OR222308	MSP1037	OR222364	MSP1435	OR221617	MSP1833	OR221271
MSP0283	OR222647	MSP0660	OR222302	MSP1038	OR222365	MSP1436	OR221619	MSP1834	OR221272
MSP0284	OR222650	MSP0661	OR222303	MSP1039	OR222357	MSP1438	OR221618	MSP1835	OR221273

MSP0285	OR222644	MSP0662	OR222304	MSP1041	OR222359	MSP1439	OR221620	MSP1836	OR221274
MSP0286	OR222645	MSP0663	OR222305	MSP1042	OR222360	MSP1440	OR221622	MSP1838	OR221275
MSP0287	OR222646	MSP0664	OR222298	MSP1043	OR222362	MSP1441	OR221610	MSP1839	OR221277
MSP0288	OR222642	MSP0665	OR222299	MSP1044	OR222353	MSP1442	OR221612	MSP1840	OR221278
MSP0289	OR222643	MSP0666	OR222301	MSP1045	OR222354	MSP1443	OR221614	MSP1841	OR221266
MSP0290	OR222639	MSP0667	OR222300	MSP1046	OR222355	MSP1444	OR221613	MSP1842	OR221268
MSP0291	OR222640	MSP0668	OR222294	MSP1047	OR222358	MSP1445	OR221616	MSP1843	OR221267
MSP0292	OR222641	MSP0669	OR222295	MSP1048	OR222356	MSP1448	OR221605	MSP1844	OR221269
MSP0293	OR222636	MSP0670	OR222297	MSP1049	OR222349	MSP1449	OR221607	MSP1845	OR221270
MSP0294	OR222637	MSP0672	OR222296	MSP1050	OR222348	MSP1450	OR221608	MSP1846	OR221261
MSP0295	OR222638	MSP0673	OR222290	MSP1051	OR222350	MSP1451	OR221609	MSP1847	OR221260
MSP0296	OR222634	MSP0674	OR222292	MSP1052	OR222352	MSP1453	OR221611	MSP1848	OR221263
MSP0297	OR222635	MSP0675	OR222291	MSP1054	OR222351	MSP1454	OR221600	MSP1849	OR221262
MSP0298	OR222630	MSP0676	OR222293	MSP1055	OR222343	MSP1456	OR221602	MSP1850	OR221264
MSP0299	OR222632	MSP0677	OR222286	MSP1056	OR222345	MSP1457	OR221603	MSP1851	OR221265
MSP0300	OR222633	MSP0678	OR222287	MSP1057	OR222346	MSP1458	OR221604	MSP1854	OR221256
MSP0301	OR222628	MSP0680	OR222288	MSP1058	OR222347	MSP1459	OR221606	MSP1855	OR221258
MSP0302	OR222629	MSP0681	OR222289	MSP1059	OR222338	MSP1460	OR221595	MSP1857	OR221257
MSP0303	OR222631	MSP0682	OR222282	MSP1060	OR222340	MSP1461	OR221597	MSP1858	OR221259
MSP0304	OR222627	MSP0683	OR222283	MSP1061	OR222341	MSP1462	OR221598	MSP1860	OR221251
MSP0305	OR222625	MSP0684	OR222285	MSP1062	OR222342	MSP1463	OR221599	MSP1861	OR221250
MSP0306	OR222623	MSP0685	OR222284	MSP1063	OR222344	MSP1466	OR221601	MSP1862	OR221252
MSP0307	OR222624	MSP0686	OR222278	MSP1064	OR222335	MSP1468	OR221591	MSP1863	OR221253
MSP0308	OR222626	MSP0687	OR222279	MSP1065	OR222336	MSP1469	OR221590	MSP1864	OR221254
MSP0309	OR222620	MSP0688	OR222280	MSP1066	OR222337	MSP1470	OR221592	MSP1865	OR221255
MSP0310	OR222622	MSP0689	OR222281	MSP1067	OR222339	MSP1472	OR221594	MSP1867	OR221242
MSP0311	OR222621	MSP0690	OR222275	MSP1068	OR222330	MSP1473	OR221593	MSP1868	OR221246
MSP0312	OR222616	MSP0691	OR222276	MSP1069	OR222331	MSP1474	OR221596	MSP1869	OR221245
MSP0313	OR222618	MSP0692	OR222277	MSP1070	OR222333	MSP1475	OR221585	MSP1870	OR221247
MSP0314	OR222619	MSP0693	OR222271	MSP1072	OR222332	MSP1476	OR221584	MSP1871	OR221249
MSP0315	OR222617	MSP0694	OR222270	MSP1073	OR222334	MSP1477	OR221586	MSP1872	OR221248
MSP0316	OR222614	MSP0695	OR222272	MSP1074	OR222325	MSP1478	OR221587	MSP1873	OR221239
MSP0317	OR222610	MSP0696	OR222273	MSP1075	OR222326	MSP1479	OR221588	MSP1875	OR221237

MSP0318	OR222613	MSP0697	OR222274	MSP1076	OR222327	MSP1480	OR221589	MSP1876	OR221240
MSP0319	OR222615	MSP0698	OR222267	MSP1077	OR222328	MSP1482	OR221579	MSP1877	OR221241
MSP0320	OR222609	MSP0699	OR222268	MSP1078	OR222329	MSP1483	OR221577	MSP1878	OR221244
MSP0321	OR222611	MSP0700	OR222269	MSP1079	OR222320	MSP1484	OR221581	MSP1879	OR221243
MSP0322	OR222612	MSP0701	OR222263	MSP1080	OR222321	MSP1485	OR221583	MSP1880	OR221231
MSP0323	OR222605	MSP0702	OR222264	MSP1081	OR222322	MSP1487	OR221582	MSP1881	OR221233
MSP0325	OR222607	MSP0703	OR222265	MSP1082	OR222324	MSP1488	OR221575	MSP1882	OR221234
MSP0326	OR222608	MSP0704	OR222266	MSP1083	OR222323	MSP1489	OR221573	MSP1883	OR221235
MSP0327	OR222604	MSP0705	OR222259	MSP1084	OR222315	MSP1490	OR221574	MSP1884	OR221236
MSP0328	OR222606	MSP0706	OR222261	MSP1085	OR222318	MSP1491	OR221576	MSP1885	OR221238
MSP0329	OR222601	MSP0707	OR222260	MSP1086	OR222316	MSP1492	OR221578	MSP1886	OR221227
MSP0330	OR222602	MSP0708	OR222262	MSP1087	OR222319	MSP1493	OR221580	MSP1887	OR221229
MSP0331	OR222603	MSP0709	OR222255	MSP1088	OR222314	MSP1494	OR221567	MSP1889	OR221228
MSP0332	OR222598	MSP0710	OR222257	MSP1089	OR222311	MSP1495	OR221570	MSP1890	OR221230
MSP0333	OR222599	MSP0711	OR222256	MSP1090	OR222310	MSP1496	OR221569	MSP1891	OR221232
MSP0334	OR222600	MSP0712	OR222258	MSP1091	OR222317	MSP1497	OR221571	MSP1892	OR221222
MSP0335	OR222595	MSP0713	OR222251	MSP1093	OR222313	MSP1498	OR221572	MSP1893	OR221221
MSP0336	OR222596	MSP0714	OR222252	MSP1094	OR222312	MSP1500	OR221565	MSP1894	OR221223
MSP0337	OR222597	MSP0715	OR222253	MSP1095	OR221907	MSP1501	OR221563	MSP1895	OR221224
MSP0338	OR222593	MSP0716	OR222254	MSP1096	OR221909	MSP1502	OR221562	MSP1896	OR221225
MSP0340	OR222594	MSP0717	OR222248	MSP1097	OR221908	MSP1503	OR221566	MSP1897	OR221226
MSP0341	OR222590	MSP0718	OR222249	MSP1098	OR221901	MSP1504	OR221568	MSP1898	OR221213
MSP0342	OR222591	MSP0719	OR222250	MSP1099	OR221903	MSP1505	OR221559	MSP1899	OR221215
MSP0343	OR222592	MSP0720	OR222245	MSP1100	OR221904	MSP1506	OR221557	MSP1900	OR221216
MSP0344	OR222587	MSP0721	OR222246	MSP1101	OR221905	MSP1507	OR221558	MSP1901	OR221218
MSP0345	OR222588	MSP0722	OR222247	MSP1102	OR221906	MSP1508	OR221560	MSP1902	OR221217
MSP0346	OR222589	MSP0723	OR222241	MSP1103	OR221896	MSP1509	OR221561	MSP1903	OR221219
MSP0347	OR222584	MSP0724	OR222242	MSP1104	OR221899	MSP1510	OR221564	MSP1904	OR221220
MSP0348	OR222585	MSP0726	OR222244	MSP1105	OR221898	MSP1511	OR221552	MSP1905	OR221209
MSP0349	OR222586	MSP0727	OR222243	MSP1106	OR221900	MSP1512	OR221555	MSP1907	OR221210
MSP0350	OR222581	MSP0728	OR222238	MSP1107	OR221902	MSP1513	OR221554	MSP1908	OR221211
MSP0352	OR222582	MSP0729	OR222237	MSP1108	OR221894	MSP1514	OR221556	MSP1909	OR221212
MSP0353	OR222583	MSP0730	OR222239	MSP1109	OR221892	MSP1515	OR221546	MSP1910	OR221214

MSP0354	OR222578	MSP0731	OR222240	MSP1110	OR221893	MSP1516	OR221548	MSP1911	OR221204
MSP0355	OR222579	MSP0732	OR222232	MSP1111	OR221895	MSP1517	OR221549	MSP1912	OR221203
MSP0356	OR222580	MSP0734	OR222234	MSP1113	OR221897	MSP1521	OR221550	MSP1913	OR221206
MSP0357	OR222575	MSP0735	OR222233	MSP1114	OR221885	MSP1522	OR221551	MSP1914	OR221208
MSP0358	OR222576	MSP0736	OR222235	MSP1115	OR221887	MSP1526	OR221553	MSP1915	OR221207
MSP0359	OR222577	MSP0737	OR222236	MSP1116	OR221889	MSP1527	OR221541	MSP1916	OR221198
MSP0360	OR222571	MSP0738	OR222228	MSP1117	OR221888	MSP1530	OR221543	MSP1917	OR221196
MSP0361	OR222572	MSP0739	OR222229	MSP1118	OR221890	MSP1531	OR221544	MSP1918	OR221197
MSP0362	OR222573	MSP0740	OR222230	MSP1119	OR221891	MSP1532	OR221545	MSP1919	OR221201
MSP0363	OR222574	MSP0742	OR222231	MSP1120	OR221881	MSP1533	OR221547	MSP1920	OR221202
MSP0364	OR222568	MSP0743	OR222224	MSP1121	OR221880	MSP1534	OR221536	MSP1921	OR221205
MSP0365	OR222567	MSP0744	OR222225	MSP1122	OR221882	MSP1535	OR221538	MSP1922	OR221200
MSP0366	OR222570	MSP0745	OR222226	MSP1123	OR221884	MSP1537	OR221539	MSP1923	OR221199
MSP0367	OR222565	MSP0746	OR222227	MSP1124	OR221886	MSP1538	OR221542		
