# Supplementary material for: Publisher Correction: Discovery and description of novel phage genomes from urban microbiomes sampled by the MetaSUB consortium
Source: Sci Rep. 2024 May 10;14:10731. doi: 10.1038/s41598-024-60777-1 (PMC11087461; doi:10.1038/s41598-024-60777-1)
Supplement: Supplementary file 1 — Supplementary Information. [file 41598_2024_60777_MOESM1_ESM.pdf]

These phage sequences are also available from http://www.iq.usp.br/setubal/metasubphages/ppgsequences.fasta

PPG	ACCESSION	PPG	ACCESSION	PPG	ACCESSION	PPG	ACCESSION	PPG	ACCESSION
MSP0001	OR222109	MSP0368	OR222566	MSP0747	OR222221	MSP1125	OR221877	MSP1539	OR221540
MSP0002	OR222107	MSP0369	OR222569	MSP0748	OR222222	MSP1126	OR221879	MSP1540	OR221531
MSP0003	OR222104	MSP0370	OR222562	MSP0749	OR222223	MSP1127	OR221878	MSP1541	OR221533
MSP0004	OR222108	MSP0371	OR222564	MSP0750	OR222217	MSP1128	OR221883	MSP1542	OR221534
MSP0005	OR222105	MSP0372	OR222563	MSP0751	OR222218	MSP1129	OR221872	MSP1543	OR221535
MSP0006	OR222106	MSP0373	OR222559	MSP0753	OR222219	MSP1130	OR221873	MSP1545	OR221537
MSP0007	OR222103	MSP0374	OR222560	MSP0754	OR222220	MSP1131	OR221875	MSP1546	OR221525
MSP0008	OR222102	MSP0375	OR222561	MSP0755	OR222212	MSP1133	OR221874	MSP1547	OR221527
MSP0010	OR222100	MSP0376	OR222556	MSP0756	OR222214	MSP1134	OR221876	MSP1548	OR221529
MSP0011	OR222101	MSP0377	OR222558	MSP0757	OR222215	MSP1135	OR221869	MSP1549	OR221532
MSP0012	OR222099	MSP0378	OR222557	MSP0758	OR222216	MSP1136	OR221868	MSP1551	OR221530
MSP0013	OR222098	MSP0379	OR222552	MSP0759	OR222209	MSP1137	OR221870	MSP1553	OR221523
MSP0014	OR222095	MSP0380	OR222554	MSP0760	OR222210	MSP1138	OR221871	MSP1554	OR221520
MSP0015	OR222096	MSP0381	OR222555	MSP0761	OR222211	MSP1139	OR221865	MSP1556	OR221521
MSP0016	OR222097	MSP0382	OR222551	MSP0762	OR222213	MSP1141	OR221864	MSP1557	OR221524
MSP0017	OR222092	MSP0383	OR222553	MSP0763	OR222205	MSP1142	OR221866	MSP1558	OR221526
MSP0018	OR222094	MSP0384	OR222548	MSP0764	OR222206	MSP1143	OR221867	MSP1559	OR221528
MSP0019	OR222093	MSP0385	OR222547	MSP0765	OR222207	MSP1144	OR221858	MSP1560	OR221516
MSP0020	OR222090	MSP0386	OR222549	MSP0766	OR222208	MSP1145	OR221860	MSP1561	OR221519
MSP0022	OR222091	MSP0387	OR222550	MSP0767	OR222204	MSP1147	OR221861	MSP1562	OR221517
MSP0023	OR222089	MSP0388	OR222545	MSP0768	OR222201	MSP1149	OR221863	MSP1563	OR221518
MSP0024	OR222088	MSP0389	OR222546	MSP0769	OR222202	MSP1151	OR221862	MSP1564	OR221522
MSP0025	OR222087	MSP0391	OR222542	MSP0771	OR222203	MSP1152	OR221854	MSP1565	OR221511
MSP0026	OR222085	MSP0392	OR222544	MSP0772	OR222199	MSP1153	OR221855	MSP1566	OR221514
MSP0027	OR222084	MSP0393	OR222540	MSP0774	OR222197	MSP1154	OR221857	MSP1567	OR221512
MSP0028	OR222082	MSP0396	OR222543	MSP0775	OR222200	MSP1155	OR221856	MSP1568	OR221513
MSP0029	OR222086	MSP0398	OR222541	MSP0776	OR222193	MSP1156	OR221859	MSP1569	OR221515
MSP0030	OR222083	MSP0399	OR222538	MSP0777	OR222194	MSP1157	OR221848	MSP1570	OR221510
MSP0031	OR222081	MSP0400	OR222539	MSP0779	OR222196	MSP1158	OR221850	MSP1571	OR221505
MSP0032	OR222080	MSP0401	OR222535	MSP0781	OR222198	MSP1159	OR221851	MSP1572	OR221506
MSP0033	OR222079	MSP0402	OR222534	MSP0782	OR222190	MSP1160	OR221852	MSP1573	OR221507
MSP0034	OR222078	MSP0403	OR222537	MSP0783	OR222191	MSP1161	OR221853	MSP1574	OR221508

MSP0035	OR222076	MSP0404	OR222536	MSP0784	OR222192	MSP1162	OR221845	MSP1575	OR221509
MSP0036	OR222077	MSP0405	OR222532	MSP0785	OR222195	MSP1163	OR221847	MSP1576	OR221500
MSP0037	OR222074	MSP0406	OR222533	MSP0786	OR222187	MSP1164	OR221846	MSP1577	OR221501
MSP0038	OR222073	MSP0407	OR222530	MSP0787	OR222189	MSP1166	OR221849	MSP1578	OR221502
MSP0040	OR222075	MSP0408	OR222531	MSP0788	OR222188	MSP1167	OR221840	MSP1579	OR221503
MSP0041	OR222072	MSP0409	OR222527	MSP0789	OR222184	MSP1169	OR221841	MSP1580	OR221504
MSP0042	OR222070	MSP0410	OR222525	MSP0790	OR222182	MSP1170	OR221842	MSP1581	OR221495
MSP0043	OR222071	MSP0411	OR222529	MSP0791	OR222185	MSP1171	OR221843	MSP1582	OR221496
MSP0044	OR222069	MSP0412	OR222524	MSP0792	OR222186	MSP1172	OR221844	MSP1583	OR221497
MSP0045	OR222066	MSP0414	OR222528	MSP0793	OR222179	MSP1173	OR221835	MSP1584	OR221498
MSP0046	OR222068	MSP0415	OR222526	MSP0794	OR222180	MSP1174	OR221836	MSP1585	OR221499
MSP0047	OR222065	MSP0417	OR222521	MSP0795	OR222181	MSP1175	OR221837	MSP1586	OR221489
MSP0048	OR222064	MSP0418	OR222523	MSP0796	OR222183	MSP1176	OR221838	MSP1587	OR221490
MSP0049	OR222067	MSP0419	OR222519	MSP0797	OR222174	MSP1178	OR221839	MSP1588	OR221491
MSP0050	OR222063	MSP0420	OR222520	MSP0798	OR222176	MSP1179	OR221831	MSP1589	OR221492
MSP0051	OR222060	MSP0421	OR222522	MSP0799	OR222177	MSP1180	OR221832	MSP1590	OR221493
MSP0052	OR222062	MSP0423	OR222516	MSP0800	OR222178	MSP1181	OR221833	MSP1591	OR221494
MSP0054	OR222061	MSP0428	OR222518	MSP0801	OR222172	MSP1182	OR221834	MSP1592	OR221484
MSP0055	OR222058	MSP0429	OR222517	MSP0803	OR222171	MSP1183	OR221826	MSP1593	OR221485
MSP0056	OR222059	MSP0430	OR222510	MSP0804	OR222173	MSP1184	OR221827	MSP1594	OR221487
MSP0057	OR222056	MSP0431	OR222512	MSP0805	OR222175	MSP1185	OR221828	MSP1595	OR221486
MSP0058	OR222057	MSP0432	OR222515	MSP0806	OR222166	MSP1186	OR221829	MSP1597	OR221488
MSP0059	OR222055	MSP0433	OR222513	MSP0807	OR222168	MSP1187	OR221830	MSP1598	OR221479
MSP0060	OR222053	MSP0434	OR222511	MSP0808	OR222169	MSP1188	OR221820	MSP1599	OR221480
MSP0061	OR222054	MSP0435	OR222514	MSP0809	OR222170	MSP1189	OR221821	MSP1600	OR221482
MSP0062	OR222052	MSP0436	OR222906	MSP0810	OR222163	MSP1190	OR221823	MSP1601	OR221481
MSP0063	OR222050	MSP0437	OR222907	MSP0812	OR222165	MSP1192	OR221824	MSP1602	OR221483
MSP0064	OR222051	MSP0438	OR222908	MSP0813	OR222164	MSP1193	OR221825	MSP1603	OR221474
MSP0065	OR222048	MSP0440	OR222909	MSP0814	OR222167	MSP1194	OR221816	MSP1604	OR221477
MSP0066	OR222049	MSP0441	OR222904	MSP0815	OR222159	MSP1195	OR221817	MSP1605	OR221475
MSP0067	OR222046	MSP0442	OR222905	MSP0817	OR222158	MSP1197	OR221818	MSP1606	OR221476
MSP0068	OR222047	MSP0443	OR222899	MSP0818	OR222160	MSP1198	OR221819	MSP1607	OR221478
MSP0069	OR222045	MSP0444	OR222900	MSP0819	OR222161	MSP1199	OR221822	MSP1608	OR221468

MSP0070	OR222043	MSP0445	OR222902	MSP0820	OR222162	MSP1202	OR221810	MSP1609	OR221471
MSP0071	OR222044	MSP0446	OR222903	MSP0821	OR222155	MSP1204	OR221812	MSP1610	OR221469
MSP0072	OR222042	MSP0448	OR222897	MSP0823	OR222153	MSP1206	OR221813	MSP1611	OR221470
MSP0073	OR222041	MSP0449	OR222898	MSP0824	OR222157	MSP1207	OR221814	MSP1612	OR221472
MSP0074	OR222040	MSP0450	OR222901	MSP0825	OR222156	MSP1208	OR221815	MSP1613	OR221473
MSP0075	OR222038	MSP0451	OR222893	MSP0827	OR222150	MSP1209	OR221805	MSP1614	OR221462
MSP0076	OR222039	MSP0452	OR222894	MSP0828	OR222151	MSP1211	OR221808	MSP1615	OR221465
MSP0077	OR222036	MSP0453	OR222895	MSP0829	OR222154	MSP1213	OR221807	MSP1616	OR221463
MSP0078	OR222037	MSP0454	OR222896	MSP0831	OR222152	MSP1214	OR221811	MSP1617	OR221464
MSP0079	OR222034	MSP0455	OR222889	MSP0832	OR222145	MSP1215	OR221809	MSP1618	OR221466
MSP0081	OR222033	MSP0456	OR222891	MSP0833	OR222146	MSP1216	OR221802	MSP1619	OR221467
MSP0082	OR222032	MSP0457	OR222892	MSP0834	OR222147	MSP1217	OR221803	MSP1620	OR221455
MSP0083	OR222031	MSP0458	OR222887	MSP0835	OR222148	MSP1219	OR221804	MSP1621	OR221458
MSP0084	OR222035	MSP0459	OR222890	MSP0836	OR222149	MSP1220	OR221806	MSP1622	OR221457
MSP0085	OR222029	MSP0460	OR222888	MSP0837	OR222141	MSP1221	OR221797	MSP1623	OR221459
MSP0086	OR222030	MSP0461	OR222884	MSP0838	OR222143	MSP1222	OR221798	MSP1624	OR221460
MSP0088	OR222027	MSP0463	OR222885	MSP0839	OR222142	MSP1223	OR221799	MSP1625	OR221461
MSP0089	OR222028	MSP0465	OR222886	MSP0840	OR222144	MSP1225	OR221801	MSP1626	OR221451
MSP0090	OR222025	MSP0466	OR222879	MSP0841	OR222137	MSP1226	OR221800	MSP1627	OR221453
MSP0091	OR222026	MSP0467	OR222881	MSP0842	OR222138	MSP1227	OR221793	MSP1629	OR221454
MSP0094	OR222023	MSP0468	OR222883	MSP0843	OR222140	MSP1228	OR221794	MSP1630	OR221456
MSP0095	OR222024	MSP0469	OR222882	MSP0844	OR222139	MSP1229	OR221796	MSP1631	OR221446
MSP0097	OR222022	MSP0470	OR222878	MSP0845	OR222133	MSP1230	OR221795	MSP1632	OR221447
MSP0101	OR222021	MSP0471	OR222877	MSP0846	OR222134	MSP1232	OR221787	MSP1633	OR221448
MSP0102	OR222020	MSP0472	OR222880	MSP0847	OR222135	MSP1233	OR221788	MSP1634	OR221449
MSP0103	OR222019	MSP0473	OR222874	MSP0848	OR222136	MSP1234	OR221790	MSP1635	OR221450
MSP0104	OR222018	MSP0474	OR222875	MSP0850	OR222128	MSP1235	OR221789	MSP1636	OR221452
MSP0105	OR222015	MSP0475	OR222876	MSP0851	OR222129	MSP1236	OR221791	MSP1637	OR221441
MSP0106	OR222017	MSP0476	OR222873	MSP0852	OR222130	MSP1237	OR221792	MSP1638	OR221442
MSP0107	OR222014	MSP0477	OR222871	MSP0853	OR222132	MSP1238	OR221783	MSP1639	OR221444
MSP0108	OR222016	MSP0478	OR222872	MSP0854	OR222131	MSP1240	OR221786	MSP1642	OR221443
MSP0109	OR222012	MSP0479	OR222869	MSP0855	OR222125	MSP1241	OR221784	MSP1644	OR221445
MSP0110	OR222013	MSP0481	OR222867	MSP0856	OR222126	MSP1242	OR221785	MSP1645	OR221436

MSP0111	OR222011	MSP0483	OR222868	MSP0858	OR222127	MSP1243	OR221780	MSP1646	OR221439
MSP0112	OR222010	MSP0484	OR222870	MSP0859	OR222121	MSP1244	OR221779	MSP1647	OR221437
MSP0113	OR222009	MSP0485	OR222864	MSP0860	OR222122	MSP1245	OR221782	MSP1649	OR221438
MSP0114	OR222008	MSP0486	OR222865	MSP0861	OR222124	MSP1246	OR221781	MSP1650	OR221440
MSP0115	OR222006	MSP0487	OR222866	MSP0862	OR222123	MSP1247	OR221777	MSP1651	OR221431
MSP0116	OR222007	MSP0488	OR222860	MSP0863	OR222118	MSP1248	OR221775	MSP1652	OR221430
MSP0119	OR222004	MSP0489	OR222861	MSP0865	OR222119	MSP1249	OR221774	MSP1653	OR221432
MSP0120	OR222005	MSP0491	OR222862	MSP0866	OR222116	MSP1250	OR221776	MSP1654	OR221433
MSP0121	OR222002	MSP0492	OR222863	MSP0867	OR222120	MSP1251	OR221778	MSP1657	OR221434
MSP0122	OR222003	MSP0493	OR222857	MSP0868	OR222113	MSP1252	OR221769	MSP1659	OR221435
MSP0123	OR221999	MSP0494	OR222858	MSP0869	OR222112	MSP1253	OR221771	MSP1660	OR221425
MSP0124	OR222000	MSP0495	OR222859	MSP0870	OR222114	MSP1254	OR221772	MSP1661	OR221426
MSP0125	OR221998	MSP0496	OR222854	MSP0871	OR222115	MSP1255	OR221770	MSP1662	OR221427
MSP0127	OR222001	MSP0497	OR222855	MSP0872	OR222117	MSP1256	OR221773	MSP1664	OR221428
MSP0128	OR221997	MSP0498	OR222856	MSP0873	OR222110	MSP1257	OR221763	MSP1666	OR221429
MSP0129	OR221996	MSP0499	OR222851	MSP0874	OR222111	MSP1259	OR221766	MSP1667	OR221421
MSP0130	OR221995	MSP0500	OR222852	MSP0875	OR222509	MSP1260	OR221767	MSP1668	OR221423
MSP0131	OR221993	MSP0501	OR222853	MSP0876	OR222505	MSP1261	OR221765	MSP1669	OR221420
MSP0132	OR221994	MSP0502	OR222847	MSP0877	OR222504	MSP1262	OR221768	MSP1670	OR221422
MSP0133	OR221991	MSP0503	OR222849	MSP0878	OR222508	MSP1263	OR221759	MSP1671	OR221424
MSP0134	OR221992	MSP0504	OR222850	MSP0880	OR222507	MSP1264	OR221762	MSP1672	OR221417
MSP0135	OR221989	MSP0505	OR222844	MSP0881	OR222506	MSP1266	OR221760	MSP1673	OR221415
MSP0136	OR221990	MSP0506	OR222845	MSP0882	OR222500	MSP1268	OR221761	MSP1674	OR221416
MSP0137	OR221987	MSP0507	OR222846	MSP0883	OR222502	MSP1270	OR221764	MSP1676	OR221418
MSP0139	OR221988	MSP0509	OR222848	MSP0884	OR222501	MSP1272	OR221754	MSP1677	OR221419
MSP0140	OR221986	MSP0510	OR222841	MSP0885	OR222503	MSP1273	OR221756	MSP1678	OR221409
MSP0141	OR221985	MSP0511	OR222843	MSP0886	OR222497	MSP1275	OR221755	MSP1680	OR221411
MSP0142	OR221983	MSP0512	OR222842	MSP0887	OR222496	MSP1276	OR221757	MSP1681	OR221410
MSP0143	OR221984	MSP0514	OR222838	MSP0888	OR222498	MSP1277	OR221758	MSP1682	OR221412
MSP0144	OR221982	MSP0515	OR222839	MSP0889	OR222499	MSP1278	OR221748	MSP1683	OR221413
MSP0145	OR221979	MSP0516	OR222840	MSP0890	OR222491	MSP1279	OR221750	MSP1684	OR221414
MSP0146	OR221980	MSP0517	OR222836	MSP0891	OR222493	MSP1280	OR221751	MSP1685	OR221405
MSP0147	OR221981	MSP0518	OR222837	MSP0892	OR222494	MSP1281	OR221752	MSP1686	OR221404

MSP0148	OR221976	MSP0519	OR222835	MSP0893	OR222495	MSP1283	OR221753	MSP1687	OR221406
MSP0149	OR221978	MSP0520	OR222834	MSP0894	OR222487	MSP1285	OR221745	MSP1689	OR221407
MSP0150	OR221975	MSP0521	OR222831	MSP0896	OR222489	MSP1286	OR221744	MSP1691	OR221408
MSP0151	OR221977	MSP0522	OR222832	MSP0898	OR222490	MSP1288	OR221746	MSP1692	OR221398
MSP0152	OR221973	MSP0523	OR222833	MSP0899	OR222492	MSP1289	OR221747	MSP1693	OR221399
MSP0153	OR221974	MSP0524	OR222827	MSP0900	OR222484	MSP1290	OR221749	MSP1694	OR221401
MSP0154	OR221971	MSP0526	OR222828	MSP0901	OR222486	MSP1292	OR221737	MSP1695	OR221400
MSP0155	OR221972	MSP0527	OR222830	MSP0902	OR222488	MSP1293	OR221739	MSP1696	OR221402
MSP0156	OR221968	MSP0528	OR222829	MSP0903	OR222485	MSP1294	OR221741	MSP1697	OR221403
MSP0157	OR221969	MSP0529	OR222822	MSP0904	OR222480	MSP1295	OR221742	MSP1698	OR221393
MSP0158	OR221966	MSP0530	OR222825	MSP0906	OR222482	MSP1296	OR221743	MSP1699	OR221394
MSP0159	OR221967	MSP0531	OR222826	MSP0907	OR222481	MSP1297	OR221733	MSP1700	OR221395
MSP0160	OR221970	MSP0533	OR222824	MSP0908	OR222483	MSP1298	OR221734	MSP1701	OR221397
MSP0161	OR221963	MSP0534	OR222819	MSP0909	OR222475	MSP1299	OR221735	MSP1702	OR221396
MSP0162	OR221965	MSP0535	OR222820	MSP0910	OR222478	MSP1300	OR221740	MSP1703	OR221386
MSP0163	OR221962	MSP0536	OR222821	MSP0911	OR222477	MSP1301	OR221738	MSP1704	OR221388
MSP0164	OR221964	MSP0537	OR222823	MSP0912	OR222479	MSP1302	OR221729	MSP1705	OR221389
MSP0165	OR221958	MSP0538	OR222818	MSP0913	OR222472	MSP1303	OR221730	MSP1706	OR221390
MSP0166	OR221961	MSP0539	OR222815	MSP0914	OR222474	MSP1304	OR221731	MSP1708	OR221392
MSP0167	OR221959	MSP0540	OR222816	MSP0915	OR222476	MSP1305	OR221732	MSP1709	OR221391
MSP0168	OR221960	MSP0541	OR222811	MSP0916	OR222467	MSP1306	OR221736	MSP1710	OR221381
MSP0169	OR221957	MSP0542	OR222814	MSP0917	OR222469	MSP1307	OR221723	MSP1711	OR221383
MSP0170	OR221956	MSP0543	OR222817	MSP0918	OR222471	MSP1308	OR221725	MSP1712	OR221384
MSP0171	OR221954	MSP0544	OR222812	MSP0919	OR222470	MSP1309	OR221726	MSP1713	OR221385
MSP0172	OR221953	MSP0545	OR222809	MSP0920	OR222473	MSP1310	OR221727	MSP1714	OR221387
MSP0173	OR221952	MSP0546	OR222813	MSP0921	OR222464	MSP1312	OR221728	MSP1715	OR221377
MSP0174	OR221955	MSP0547	OR222810	MSP0922	OR222466	MSP1313	OR221719	MSP1716	OR221378
MSP0176	OR221948	MSP0548	OR222806	MSP0923	OR222465	MSP1314	OR221720	MSP1717	OR221379
MSP0177	OR221951	MSP0549	OR222808	MSP0924	OR222468	MSP1315	OR221721	MSP1718	OR221380
MSP0178	OR221950	MSP0550	OR222807	MSP0925	OR222459	MSP1316	OR221722	MSP1719	OR221382
MSP0179	OR221949	MSP0552	OR222802	MSP0926	OR222461	MSP1318	OR221724	MSP1720	OR221371
MSP0180	OR221946	MSP0553	OR222803	MSP0927	OR222462	MSP1319	OR221713	MSP1721	OR221373
MSP0181	OR221947	MSP0554	OR222805	MSP0928	OR222463	MSP1320	OR221716	MSP1722	OR221372

MSP0182	OR221943	MSP0555	OR222804	MSP0929	OR222456	MSP1322	OR221714	MSP1723	OR221376
MSP0183	OR221945	MSP0556	OR222799	MSP0930	OR222457	MSP1323	OR221717	MSP1724	OR221374
MSP0184	OR221942	MSP0557	OR222800	MSP0931	OR222458	MSP1324	OR221718	MSP1725	OR221375
MSP0185	OR221944	MSP0558	OR222801	MSP0934	OR222460	MSP1326	OR221708	MSP1726	OR221367
MSP0186	OR221941	MSP0559	OR222795	MSP0935	OR222451	MSP1327	OR221710	MSP1727	OR221368
MSP0187	OR221940	MSP0560	OR222796	MSP0936	OR222453	MSP1328	OR221709	MSP1728	OR221366
MSP0188	OR221938	MSP0561	OR222797	MSP0938	OR222454	MSP1329	OR221711	MSP1729	OR221370
MSP0189	OR221937	MSP0562	OR222798	MSP0939	OR222455	MSP1330	OR221712	MSP1730	OR221369
MSP0190	OR221939	MSP0563	OR222792	MSP0940	OR222448	MSP1331	OR221715	MSP1731	OR221360
MSP0192	OR221934	MSP0564	OR222793	MSP0941	OR222450	MSP1332	OR221703	MSP1732	OR221362
MSP0193	OR221936	MSP0565	OR222794	MSP0942	OR222449	MSP1333	OR221704	MSP1733	OR221361
MSP0195	OR221933	MSP0566	OR222789	MSP0944	OR222452	MSP1334	OR221705	MSP1734	OR221363
MSP0196	OR221935	MSP0567	OR222790	MSP0945	OR222443	MSP1335	OR221706	MSP1735	OR221364
MSP0197	OR221931	MSP0568	OR222791	MSP0946	OR222445	MSP1337	OR221707	MSP1736	OR221365
MSP0198	OR221932	MSP0569	OR222785	MSP0947	OR222444	MSP1338	OR221698	MSP1737	OR221354
MSP0199	OR221928	MSP0570	OR222786	MSP0948	OR222447	MSP1339	OR221699	MSP1738	OR221355
MSP0200	OR221929	MSP0571	OR222787	MSP0949	OR222446	MSP1340	OR221700	MSP1740	OR221356
MSP0201	OR221930	MSP0572	OR222788	MSP0950	OR222441	MSP1341	OR221701	MSP1742	OR221358
MSP0202	OR221927	MSP0573	OR222782	MSP0951	OR222439	MSP1342	OR221702	MSP1743	OR221357
MSP0203	OR221926	MSP0574	OR222783	MSP0952	OR222442	MSP1344	OR221695	MSP1744	OR221359
MSP0204	OR221923	MSP0575	OR222784	MSP0953	OR222440	MSP1345	OR221693	MSP1746	OR221350
MSP0205	OR221925	MSP0576	OR222778	MSP0954	OR222437	MSP1346	OR221694	MSP1747	OR221349
MSP0206	OR221921	MSP0577	OR222779	MSP0955	OR222435	MSP1347	OR221696	MSP1748	OR221351
MSP0207	OR221922	MSP0578	OR222780	MSP0956	OR222436	MSP1348	OR221697	MSP1749	OR221353
MSP0208	OR221924	MSP0579	OR222781	MSP0957	OR222438	MSP1349	OR221688	MSP1750	OR221352
MSP0209	OR221919	MSP0580	OR222775	MSP0958	OR222430	MSP1350	OR221689	MSP1753	OR221344
MSP0210	OR221920	MSP0581	OR222776	MSP0959	OR222432	MSP1351	OR221690	MSP1754	OR221345
MSP0211	OR221916	MSP0582	OR222777	MSP0960	OR222431	MSP1352	OR221691	MSP1755	OR221347
MSP0212	OR221918	MSP0583	OR222773	MSP0961	OR222433	MSP1353	OR221692	MSP1756	OR221346
MSP0213	OR221915	MSP0584	OR222772	MSP0962	OR222434	MSP1354	OR221684	MSP1757	OR221348
MSP0214	OR221917	MSP0586	OR222774	MSP0963	OR222425	MSP1355	OR221683	MSP1760	OR221337
MSP0215	OR221911	MSP0587	OR222768	MSP0965	OR222426	MSP1358	OR221686	MSP1761	OR221339
MSP0216	OR221910	MSP0588	OR222769	MSP0967	OR222428	MSP1359	OR221687	MSP1762	OR221340

MSP0217	OR221913	MSP0589	OR222770	MSP0968	OR222427	MSP1360	OR221679	MSP1763	OR221342
MSP0218	OR221912	MSP0590	OR222771	MSP0969	OR222429	MSP1361	OR221681	MSP1766	OR221341
MSP0219	OR221914	MSP0591	OR222764	MSP0970	OR222421	MSP1362	OR221680	MSP1767	OR221343
MSP0220	OR222709	MSP0592	OR222765	MSP0971	OR222422	MSP1363	OR221682	MSP1768	OR221333
MSP0221	OR222708	MSP0593	OR222766	MSP0972	OR222423	MSP1364	OR221685	MSP1769	OR221332
MSP0222	OR222706	MSP0594	OR222767	MSP0973	OR222424	MSP1365	OR221677	MSP1770	OR221335
MSP0223	OR222707	MSP0595	OR222761	MSP0974	OR222417	MSP1366	OR221674	MSP1771	OR221334
MSP0224	OR222705	MSP0596	OR222762	MSP0975	OR222418	MSP1367	OR221675	MSP1772	OR221336
MSP0225	OR222703	MSP0598	OR222763	MSP0976	OR222419	MSP1368	OR221676	MSP1773	OR221338
MSP0226	OR222704	MSP0599	OR222756	MSP0977	OR222420	MSP1370	OR221678	MSP1774	OR221326
MSP0227	OR222701	MSP0600	OR222759	MSP0978	OR222413	MSP1371	OR221667	MSP1776	OR221327
MSP0228	OR222702	MSP0601	OR222758	MSP0980	OR222412	MSP1372	OR221671	MSP1777	OR221328
MSP0229	OR222699	MSP0602	OR222760	MSP0981	OR222414	MSP1373	OR221669	MSP1778	OR221329
MSP0230	OR222700	MSP0603	OR222754	MSP0982	OR222415	MSP1374	OR221670	MSP1779	OR221330
MSP0232	OR222697	MSP0604	OR222753	MSP0983	OR222416	MSP1375	OR221672	MSP1780	OR221331
MSP0233	OR222698	MSP0605	OR222755	MSP0985	OR222408	MSP1376	OR221673	MSP1781	OR221323
MSP0234	OR222694	MSP0606	OR222757	MSP0986	OR222409	MSP1377	OR221662	MSP1782	OR221322
MSP0235	OR222695	MSP0608	OR222748	MSP0987	OR222410	MSP1378	OR221664	MSP1783	OR221321
MSP0236	OR222696	MSP0609	OR222750	MSP0988	OR222411	MSP1379	OR221666	MSP1784	OR221324
MSP0237	OR222692	MSP0610	OR222751	MSP0989	OR222403	MSP1380	OR221665	MSP1785	OR221325
MSP0238	OR222693	MSP0611	OR222752	MSP0990	OR222405	MSP1382	OR221668	MSP1786	OR221316
MSP0239	OR222689	MSP0612	OR222745	MSP0991	OR222406	MSP1383	OR221658	MSP1787	OR221317
MSP0240	OR222691	MSP0613	OR222746	MSP0992	OR222407	MSP1384	OR221660	MSP1788	OR221318
MSP0241	OR222688	MSP0614	OR222749	MSP0993	OR222401	MSP1387	OR221659	MSP1789	OR221319
MSP0242	OR222687	MSP0615	OR222747	MSP0994	OR222400	MSP1388	OR221661	MSP1790	OR221320
MSP0243	OR222690	MSP0616	OR222744	MSP0995	OR222404	MSP1389	OR221663	MSP1791	OR221311
MSP0244	OR222684	MSP0617	OR222743	MSP0997	OR222402	MSP1390	OR221653	MSP1792	OR221312
MSP0245	OR222686	MSP0618	OR222742	MSP0998	OR222396	MSP1391	OR221654	MSP1793	OR221313
MSP0246	OR222685	MSP0619	OR222736	MSP0999	OR222398	MSP1392	OR221655	MSP1795	OR221314
MSP0247	OR222682	MSP0621	OR222738	MSP1000	OR222397	MSP1393	OR221656	MSP1796	OR221315
MSP0248	OR222683	MSP0623	OR222741	MSP1001	OR222399	MSP1394	OR221657	MSP1797	OR221304
MSP0249	OR222679	MSP0624	OR222740	MSP1003	OR222390	MSP1395	OR221645	MSP1798	OR221306
MSP0250	OR222681	MSP0625	OR222735	MSP1004	OR222392	MSP1397	OR221648	MSP1799	OR221308

MSP0252	OR222680	MSP0626	OR222737	MSP1006	OR222393	MSP1399	OR221649	MSP1800	OR221307
MSP0253	OR222676	MSP0627	OR222739	MSP1007	OR222394	MSP1400	OR221650	MSP1801	OR221310
MSP0254	OR222677	MSP0628	OR222732	MSP1008	OR222395	MSP1401	OR221652	MSP1802	OR221309
MSP0255	OR222678	MSP0629	OR222730	MSP1009	OR222388	MSP1402	OR221651	MSP1803	OR221299
MSP0256	OR222674	MSP0630	OR222733	MSP1010	OR222387	MSP1403	OR221643	MSP1804	OR221300
MSP0257	OR222675	MSP0632	OR222727	MSP1011	OR222389	MSP1404	OR221642	MSP1805	OR221301
MSP0258	OR222671	MSP0633	OR222729	MSP1012	OR222391	MSP1405	OR221644	MSP1806	OR221302
MSP0259	OR222672	MSP0634	OR222734	MSP1013	OR222382	MSP1406	OR221646	MSP1807	OR221303
MSP0260	OR222673	MSP0635	OR222731	MSP1014	OR222384	MSP1408	OR221647	MSP1808	OR221305
MSP0261	OR222669	MSP0636	OR222724	MSP1015	OR222386	MSP1409	OR221637	MSP1809	OR221294
MSP0262	OR222670	MSP0637	OR222728	MSP1016	OR222385	MSP1411	OR221638	MSP1810	OR221295
MSP0263	OR222665	MSP0638	OR222726	MSP1017	OR222377	MSP1412	OR221639	MSP1811	OR221296
MSP0264	OR222668	MSP0639	OR222725	MSP1018	OR222380	MSP1413	OR221640	MSP1812	OR221298
MSP0265	OR222666	MSP0640	OR222720	MSP1019	OR222381	MSP1414	OR221641	MSP1814	OR221297
MSP0266	OR222667	MSP0642	OR222721	MSP1020	OR222383	MSP1415	OR221635	MSP1815	OR221290
MSP0267	OR222661	MSP0643	OR222723	MSP1021	OR222375	MSP1416	OR221633	MSP1816	OR221289
MSP0268	OR222663	MSP0644	OR222722	MSP1022	OR222376	MSP1417	OR221634	MSP1819	OR221291
MSP0269	OR222664	MSP0645	OR222716	MSP1023	OR222379	MSP1418	OR221636	MSP1820	OR221292
MSP0270	OR222660	MSP0646	OR222717	MSP1024	OR222378	MSP1419	OR221627	MSP1821	OR221293
MSP0271	OR222662	MSP0647	OR222718	MSP1025	OR222371	MSP1420	OR221629	MSP1822	OR221283
MSP0272	OR222657	MSP0648	OR222719	MSP1026	OR222373	MSP1421	OR221628	MSP1823	OR221284
MSP0273	OR222658	MSP0650	OR222713	MSP1027	OR222374	MSP1423	OR221630	MSP1824	OR221285
MSP0274	OR222659	MSP0651	OR222712	MSP1028	OR222372	MSP1424	OR221632	MSP1825	OR221286
MSP0275	OR222655	MSP0652	OR222714	MSP1029	OR222367	MSP1425	OR221631	MSP1826	OR221287
MSP0276	OR222656	MSP0653	OR222715	MSP1031	OR222366	MSP1427	OR221621	MSP1827	OR221288
MSP0277	OR222652	MSP0654	OR222710	MSP1032	OR222368	MSP1428	OR221623	MSP1828	OR221276
MSP0278	OR222653	MSP0655	OR222711	MSP1033	OR222369	MSP1429	OR221624	MSP1829	OR221279
MSP0279	OR222654	MSP0656	OR222309	MSP1034	OR222370	MSP1432	OR221625	MSP1830	OR221281
MSP0280	OR222648	MSP0657	OR222306	MSP1035	OR222361	MSP1433	OR221626	MSP1831	OR221280
MSP0281	OR222649	MSP0658	OR222307	MSP1036	OR222363	MSP1434	OR221615	MSP1832	OR221282
MSP0282	OR222651	MSP0659	OR222308	MSP1037	OR222364	MSP1435	OR221617	MSP1833	OR221271
MSP0283	OR222647	MSP0660	OR222302	MSP1038	OR222365	MSP1436	OR221619	MSP1834	OR221272
MSP0284	OR222650	MSP0661	OR222303	MSP1039	OR222357	MSP1438	OR221618	MSP1835	OR221273

MSP0285	OR222644	MSP0662	OR222304	MSP1041	OR222359	MSP1439	OR221620	MSP1836	OR221274
MSP0286	OR222645	MSP0663	OR222305	MSP1042	OR222360	MSP1440	OR221622	MSP1838	OR221275
MSP0287	OR222646	MSP0664	OR222298	MSP1043	OR222362	MSP1441	OR221610	MSP1839	OR221277
MSP0288	OR222642	MSP0665	OR222299	MSP1044	OR222353	MSP1442	OR221612	MSP1840	OR221278
MSP0289	OR222643	MSP0666	OR222301	MSP1045	OR222354	MSP1443	OR221614	MSP1841	OR221266
MSP0290	OR222639	MSP0667	OR222300	MSP1046	OR222355	MSP1444	OR221613	MSP1842	OR221268
MSP0291	OR222640	MSP0668	OR222294	MSP1047	OR222358	MSP1445	OR221616	MSP1843	OR221267
MSP0292	OR222641	MSP0669	OR222295	MSP1048	OR222356	MSP1448	OR221605	MSP1844	OR221269
MSP0293	OR222636	MSP0670	OR222297	MSP1049	OR222349	MSP1449	OR221607	MSP1845	OR221270
MSP0294	OR222637	MSP0672	OR222296	MSP1050	OR222348	MSP1450	OR221608	MSP1846	OR221261
MSP0295	OR222638	MSP0673	OR222290	MSP1051	OR222350	MSP1451	OR221609	MSP1847	OR221260
MSP0296	OR222634	MSP0674	OR222292	MSP1052	OR222352	MSP1453	OR221611	MSP1848	OR221263
MSP0297	OR222635	MSP0675	OR222291	MSP1054	OR222351	MSP1454	OR221600	MSP1849	OR221262
MSP0298	OR222630	MSP0676	OR222293	MSP1055	OR222343	MSP1456	OR221602	MSP1850	OR221264
MSP0299	OR222632	MSP0677	OR222286	MSP1056	OR222345	MSP1457	OR221603	MSP1851	OR221265
MSP0300	OR222633	MSP0678	OR222287	MSP1057	OR222346	MSP1458	OR221604	MSP1854	OR221256
MSP0301	OR222628	MSP0680	OR222288	MSP1058	OR222347	MSP1459	OR221606	MSP1855	OR221258
MSP0302	OR222629	MSP0681	OR222289	MSP1059	OR222338	MSP1460	OR221595	MSP1857	OR221257
MSP0303	OR222631	MSP0682	OR222282	MSP1060	OR222340	MSP1461	OR221597	MSP1858	OR221259
MSP0304	OR222627	MSP0683	OR222283	MSP1061	OR222341	MSP1462	OR221598	MSP1860	OR221251
MSP0305	OR222625	MSP0684	OR222285	MSP1062	OR222342	MSP1463	OR221599	MSP1861	OR221250
MSP0306	OR222623	MSP0685	OR222284	MSP1063	OR222344	MSP1466	OR221601	MSP1862	OR221252
MSP0307	OR222624	MSP0686	OR222278	MSP1064	OR222335	MSP1468	OR221591	MSP1863	OR221253
MSP0308	OR222626	MSP0687	OR222279	MSP1065	OR222336	MSP1469	OR221590	MSP1864	OR221254
MSP0309	OR222620	MSP0688	OR222280	MSP1066	OR222337	MSP1470	OR221592	MSP1865	OR221255
MSP0310	OR222622	MSP0689	OR222281	MSP1067	OR222339	MSP1472	OR221594	MSP1867	OR221242
MSP0311	OR222621	MSP0690	OR222275	MSP1068	OR222330	MSP1473	OR221593	MSP1868	OR221246
MSP0312	OR222616	MSP0691	OR222276	MSP1069	OR222331	MSP1474	OR221596	MSP1869	OR221245
MSP0313	OR222618	MSP0692	OR222277	MSP1070	OR222333	MSP1475	OR221585	MSP1870	OR221247
MSP0314	OR222619	MSP0693	OR222271	MSP1072	OR222332	MSP1476	OR221584	MSP1871	OR221249
MSP0315	OR222617	MSP0694	OR222270	MSP1073	OR222334	MSP1477	OR221586	MSP1872	OR221248
MSP0316	OR222614	MSP0695	OR222272	MSP1074	OR222325	MSP1478	OR221587	MSP1873	OR221239
MSP0317	OR222610	MSP0696	OR222273	MSP1075	OR222326	MSP1479	OR221588	MSP1875	OR221237

MSP0318	OR222613	MSP0697	OR222274	MSP1076	OR222327	MSP1480	OR221589	MSP1876	OR221240
MSP0319	OR222615	MSP0698	OR222267	MSP1077	OR222328	MSP1482	OR221579	MSP1877	OR221241
MSP0320	OR222609	MSP0699	OR222268	MSP1078	OR222329	MSP1483	OR221577	MSP1878	OR221244
MSP0321	OR222611	MSP0700	OR222269	MSP1079	OR222320	MSP1484	OR221581	MSP1879	OR221243
MSP0322	OR222612	MSP0701	OR222263	MSP1080	OR222321	MSP1485	OR221583	MSP1880	OR221231
MSP0323	OR222605	MSP0702	OR222264	MSP1081	OR222322	MSP1487	OR221582	MSP1881	OR221233
MSP0325	OR222607	MSP0703	OR222265	MSP1082	OR222324	MSP1488	OR221575	MSP1882	OR221234
MSP0326	OR222608	MSP0704	OR222266	MSP1083	OR222323	MSP1489	OR221573	MSP1883	OR221235
MSP0327	OR222604	MSP0705	OR222259	MSP1084	OR222315	MSP1490	OR221574	MSP1884	OR221236
MSP0328	OR222606	MSP0706	OR222261	MSP1085	OR222318	MSP1491	OR221576	MSP1885	OR221238
MSP0329	OR222601	MSP0707	OR222260	MSP1086	OR222316	MSP1492	OR221578	MSP1886	OR221227
MSP0330	OR222602	MSP0708	OR222262	MSP1087	OR222319	MSP1493	OR221580	MSP1887	OR221229
MSP0331	OR222603	MSP0709	OR222255	MSP1088	OR222314	MSP1494	OR221567	MSP1889	OR221228
MSP0332	OR222598	MSP0710	OR222257	MSP1089	OR222311	MSP1495	OR221570	MSP1890	OR221230
MSP0333	OR222599	MSP0711	OR222256	MSP1090	OR222310	MSP1496	OR221569	MSP1891	OR221232
MSP0334	OR222600	MSP0712	OR222258	MSP1091	OR222317	MSP1497	OR221571	MSP1892	OR221222
MSP0335	OR222595	MSP0713	OR222251	MSP1093	OR222313	MSP1498	OR221572	MSP1893	OR221221
MSP0336	OR222596	MSP0714	OR222252	MSP1094	OR222312	MSP1500	OR221565	MSP1894	OR221223
MSP0337	OR222597	MSP0715	OR222253	MSP1095	OR221907	MSP1501	OR221563	MSP1895	OR221224
MSP0338	OR222593	MSP0716	OR222254	MSP1096	OR221909	MSP1502	OR221562	MSP1896	OR221225
MSP0340	OR222594	MSP0717	OR222248	MSP1097	OR221908	MSP1503	OR221566	MSP1897	OR221226
MSP0341	OR222590	MSP0718	OR222249	MSP1098	OR221901	MSP1504	OR221568	MSP1898	OR221213
MSP0342	OR222591	MSP0719	OR222250	MSP1099	OR221903	MSP1505	OR221559	MSP1899	OR221215
MSP0343	OR222592	MSP0720	OR222245	MSP1100	OR221904	MSP1506	OR221557	MSP1900	OR221216
MSP0344	OR222587	MSP0721	OR222246	MSP1101	OR221905	MSP1507	OR221558	MSP1901	OR221218
MSP0345	OR222588	MSP0722	OR222247	MSP1102	OR221906	MSP1508	OR221560	MSP1902	OR221217
MSP0346	OR222589	MSP0723	OR222241	MSP1103	OR221896	MSP1509	OR221561	MSP1903	OR221219
MSP0347	OR222584	MSP0724	OR222242	MSP1104	OR221899	MSP1510	OR221564	MSP1904	OR221220
MSP0348	OR222585	MSP0726	OR222244	MSP1105	OR221898	MSP1511	OR221552	MSP1905	OR221209
MSP0349	OR222586	MSP0727	OR222243	MSP1106	OR221900	MSP1512	OR221555	MSP1907	OR221210
MSP0350	OR222581	MSP0728	OR222238	MSP1107	OR221902	MSP1513	OR221554	MSP1908	OR221211
MSP0352	OR222582	MSP0729	OR222237	MSP1108	OR221894	MSP1514	OR221556	MSP1909	OR221212
MSP0353	OR222583	MSP0730	OR222239	MSP1109	OR221892	MSP1515	OR221546	MSP1910	OR221214

MSP0354	OR222578	MSP0731	OR222240	MSP1110	OR221893	MSP1516	OR221548	MSP1911	OR221204
MSP0355	OR222579	MSP0732	OR222232	MSP1111	OR221895	MSP1517	OR221549	MSP1912	OR221203
MSP0356	OR222580	MSP0734	OR222234	MSP1113	OR221897	MSP1521	OR221550	MSP1913	OR221206
MSP0357	OR222575	MSP0735	OR222233	MSP1114	OR221885	MSP1522	OR221551	MSP1914	OR221208
MSP0358	OR222576	MSP0736	OR222235	MSP1115	OR221887	MSP1526	OR221553	MSP1915	OR221207
MSP0359	OR222577	MSP0737	OR222236	MSP1116	OR221889	MSP1527	OR221541	MSP1916	OR221198
MSP0360	OR222571	MSP0738	OR222228	MSP1117	OR221888	MSP1530	OR221543	MSP1917	OR221196
MSP0361	OR222572	MSP0739	OR222229	MSP1118	OR221890	MSP1531	OR221544	MSP1918	OR221197
MSP0362	OR222573	MSP0740	OR222230	MSP1119	OR221891	MSP1532	OR221545	MSP1919	OR221201
MSP0363	OR222574	MSP0742	OR222231	MSP1120	OR221881	MSP1533	OR221547	MSP1920	OR221202
MSP0364	OR222568	MSP0743	OR222224	MSP1121	OR221880	MSP1534	OR221536	MSP1921	OR221205
MSP0365	OR222567	MSP0744	OR222225	MSP1122	OR221882	MSP1535	OR221538	MSP1922	OR221200
MSP0366	OR222570	MSP0745	OR222226	MSP1123	OR221884	MSP1537	OR221539	MSP1923	OR221199
MSP0367	OR222565	MSP0746	OR222227	MSP1124	OR221886	MSP1538	OR221542		
